# PINGU: PredIction of eNzyme catalytic residues usinG seqUence information

**DOI:** 10.1371/journal.pone.0135122

**Published:** 2015-08-11

**Authors:** Priyadarshini P. Pai, S. S. Shree Ranjani, Sukanta Mondal

**Affiliations:** 1 Department of Biological Sciences, Birla Institute of Technology and Science–Pilani, K.K. Birla Goa Campus, Zuarinagar, Goa, India; 2 Department of Computer Science and Information Systems, Birla Institute of Technology and Science–Pilani, K.K. Birla Goa Campus, Zuarinagar, Goa, India; Indian Institute of Science, INDIA

## Abstract

Identification of catalytic residues can help unveil interesting attributes of enzyme function for various therapeutic and industrial applications. Based on their biochemical roles, the number of catalytic residues and sequence lengths of enzymes vary. This article describes a prediction approach (PINGU) for such a scenario. It uses models trained using physicochemical properties and evolutionary information of 650 non-redundant enzymes (2136 catalytic residues) in a support vector machines architecture. Independent testing on 200 non-redundant enzymes (683 catalytic residues) in predefined prediction settings, i.e., with non-catalytic per catalytic residue ranging from 1 to 30, suggested that the prediction approach was highly sensitive and specific, i.e., 80% or above, over the incremental challenges. To learn more about the discriminatory power of PINGU in real scenarios, where the prediction challenge is variable and susceptible to high false positives, the best model from independent testing was used on 60 diverse enzymes. Results suggested that PINGU was able to identify most catalytic residues and non-catalytic residues properly with 80% or above accuracy, sensitivity and specificity. The effect of false positives on precision was addressed in this study by application of predicted ligand-binding residue information as a post-processing filter. An overall improvement of 20% in F-measure and 0.138 in Correlation Coefficient with 16% enhanced precision could be achieved. On account of its encouraging performance, PINGU is hoped to have eventual applications in boosting enzyme engineering and novel drug discovery.

## Introduction

Enzymes play a key role in catalyzing biochemical reactions important for life. Their function is governed by a small number of amino acids known as catalytic residues. By means of their structure and chemical properties, these residues directly take part in the catalysis process, determining to a certain extent, the chemical properties of the enzyme. Thus, gaining knowledge of the catalytic residues can not only help unravel enzyme functions, but in the long run, boost enzyme engineering and drug design applications [1, 2].

Accurate identification of enzyme catalytic residues using experimental and computational approaches has been widely attempted in biology. The computational approaches because of their time and resource utilization advantages have gained impetus through the years. These approaches fall broadly under similarity transfer based and *ab initio* or *de novo* methods [2]. The similarity transfer based methods identify putative catalytic residues in uncharacterized sequences based on their homology with sequences whose catalytic residues are known. Thus, they rely on templates, alignment and pattern matching for catalytic residue mapping. The *ab initio* methods, on the other hand, predict catalytic residues by exploiting several general properties of enzyme catalytic residues which distinguish them from non-catalytic residues. These methods are useful especially when the catalytic residues of enzymes in question are different from the characterized enzyme catalytic residues to a large extent. By applying sequence and structure information, various computational approaches have been reported over years for developing knowledge bases [3–8], analyzing important biological properties [1,9–14] and catalytic residue prediction [2,15–24]. However, there is ample scope of making these approaches more robust and handy for the users.

This article presents an approach for achieving catalytic residue predictions with improved precision by using physicochemical properties and evolutionary information from enzyme sequences in a support vector machine architecture and post-processing. First, non-redundant datasets with updated information from Catalytic Site Atlas 2.0 [8] were constructed for training and independent testing during the predictor development. Following this, using the polarity index, position specific scoring matrix and sequence conservation information, features were created and used as inputs in three classifiers, *viz*., L1-regularized logistic regression, radial basis function networks and support vector machines. The best classification model was selected upon ten-fold cross-validation and feature selection. This model was used for independent testing and insights into the prediction performance of the developed classification model were obtained. Predefined incrementally challenging prediction settings were used for this purpose, where more than one non-catalytic residue per catalytic residue, were used. Study findings showed the prediction to have above 80% accuracy, sensitivity and specificity, encouraging its investigation in a more challenging and variable situation. Consequently, the discriminative power of the developed predictor (PINGU: PredIcting eNzyme catalytic residues using sequence information) was tested in real scenario on diverse enzymes. The quality of predictions so obtained were analyzed and scope of improvement upon using a post-processing filter such as predicted ligand binding residue information was explored and found to have an advantage in improving the precision. Based on the findings, this approach is suggested to have use in precisely predicting catalytic residues with minimal resources for their eventual applications in industry or medicines.

## Materials and Methods

### 2.1. Datasets

For the construction of suitable training and independent test datasets, enzyme data for the predictor development was collected from the datasets created by Dou et al. [20] and the Catalytic Site Atlas (CSA) 2.0 dataset (updated 14 November 2013)[8]. Using this data, information of their experimentally determined structures available in the Protein Data Bank (PDB) [25] was obtained from the website (http://www.rcsb.org/). A pool of sequence information based on the ATOM record in these PDB structures was generated for the study. From this pool of enzyme sequences, the sequence fragments with lengths less 60 amino acids were filtered out. This will have ensured that the conclusions for various observations to be made in this study would be generally applicable and based on adequate information. Additionally, to limit the possible scope of overestimation and for the inclusion of diversity, remaining enzyme sequences were clustered. Clustering was performed using BLASTClust [26] into groups with ≥30% intra-cluster pair-wise sequence identity over a 60% overlap on both sequences. A total of 850 clusters were returned with 2819 catalytic residues and 312222 non-catalytic residues. From this parent non-redundant dataset, 650 enzymes were randomly allocated into the training and 200 enzymes in the independent test dataset. The training dataset was named as Dset650 and independent test dataset as Dtestset200 and employed in this study for predictor development (Details S1 Dataset).

### 2.2. Features

After building the benchmark dataset, a set of informative features that was created and used to develop the predictor in this study. It included representing the physicochemical property and evolutionary information of amino acids using polarity index, position-specific scoring matrix (PSSM) and sequence conservation score (EntWOP) as described in the following:

#### 2.2.1. Polarity index

The diversity in physicochemical properties of the 20 naturally occurring amino acids such as their polar nature can influence the specificity and diversity of the protein structure and function. This is presented as features using an index known as Polarity index [27]. It basically designates Factor I of the five factors obtained as a result of conducting multivariate statistical analyses of amino acids to solve the sequence metric problem. Factor I is bipolar (large positive and negative factor coefficients). It reflects simultaneous co-variation in portion of exposed residues versus buried residues, non-bonded energy versus free energy, number of hydrogen bond donors, polarity versus non-polarity, and hydrophobicity versus hydrophilicity. For the study, the polarity index with the following values for the 20 naturally occurring amino acids was used: (A: -0.591, C: -1.343, D: 1.050, E: 1.357, F: -1.006, G: -0.384, H: 0.336, I: -1.239, K: 1.831, L: -1.019, M: -0.663, N: 0.945, P: 0.189, Q: 0.931, R: 1.538, S: -0.228, T: -0.032,V: -1.337, W: -0.595, Y: 0.260) [27].

#### 2.2.2. PSSM and EntWOP

Evolutionary information of residues helps in developing a deeper understanding of their conservation patterns and importance in protein functioning [28, 29]. To include evolutionary information in this study, PSSM features were constructed using PSI-BLAST [26], i.e., 20-dimensional Weighted Observed Percentages (WOP) vectors were obtained for each residue. This vector for a given residue represents the log-likelihood of the substitution of 20 amino acids at that sequence position. PSSM values (*x*) for each residue is normalized by 1/(1+exp(-*x*)). Since the WOP vector for a given residue represents a frequency distribution of 20 amino acids at that sequence position, EntWOP [16] was computed using Shannon entropy: $\sum_{i=1}^{20}-p_{i}\text{log}(p_{i}),where\;p_{i}=n_{i}/\sum_{j=1}^{20}n_{i}$. EntWOP ranges between 0 (the most conserved; only one amino acid type has non-zero value at the corresponding position in the WOP vector) and 2.996 (the least conserved; all 20 amino acids have the same non-zero value in the WOP vector).

### 2.3. Feature Selection

F-score is a simple technique which measures the discrimination of two sets of real numbers [30]. Given training vectors *x*_*k*_, *k* = 1,…,*m* if the numbers of positive and negative instances are *n* ^+^ and *n* ^−^, respectively, then the F-score of the *i* ^*th*^ feature is defined as:

$$F_{i}=\frac{{\left({\bar{x}}_{i}^{\left(+\right)}-{\bar{x}}_{i}\right)}^{2}+{\left({\bar{x}}_{i}^{\left(-\right)}-{\bar{x}}_{i}\right)}^{2}}{\frac{1}{n^{+}+1}\sum_{k=1}^{n^{+}}{\left(x_{k,i}^{\left(+\right)}-{\bar{x}}_{i}^{\left(+\right)}\right)}^{2}+\frac{1}{n^{-}-1}\sum_{k=1}^{n^{-}}{\left(x_{k,i}^{\left(-\right)}-{\bar{x}}_{i}^{\left(-\right)}\right)}^{2}}$$ (1)

where ${\bar{x}}_{i},{\bar{x}}_{i}^{\left(+\right)},{\bar{x}}_{i}^{\left(-\right)}$ are the average of the *i* ^*th*^ feature of the whole, positive, and negative data sets, respectively; $x_{k,i}^{\left(+\right)}$ is the *i* ^*th*^ feature of the *k* ^*th*^ positive instance, and $x_{k.i}^{\left(-\right)}$ is the *i* ^*th*^ feature of the *k* ^*th*^ negative instance. The numerator indicates the discrimination between the positive and negative sets, and the denominator indicates the one within each of the two sets. It is more likely that with larger F-scores, the features are more discriminative. This score is used for feature selection in the study.

### 2.4. Classifier

Discrimination of catalytic residues from non-catalytic residues based on their encoded biological properties was attempted using three classifiers in this study. The best model obtained was suggested for use as a predictor. The three classifiers are described below:

#### 2.4.1. Support vector machines (SVM)

SVM is a supervised machine-learning tool based on the structural risk minimization principle of statistics learning theory. It looks for an optimal hyperplane which maximizes the distance between the hyperplane and the nearest samples from each of the two classes. Mathematically, a training vector *x*_*i*_ ∈ *R*_*n*_, and class values *y*_*i*_ ∈ {-1, 1}, *i* = 1,…,*N* are used to solve the problems using the following equation:

$$Minimize(1/2)w^{T}\cdot w+C\sum_{j=1}^{N}\xi_{i}$$ (2)

$$Subject\;to\;y_{i}(w^{T}\cdot x_{i}+b)\geq 1-\xi_{i}\;and\;\xi_{i}\geq 0$$ (3)

where *w* is the normal vector perpendicular to the hyperplane and *ξ*_*i*_ are slake variables for permitting misclassifications. Balancing the trade-off between the margin and the training error is done using C (> 0), the penalty parameter [31]. The user can choose and optimize number of parameters and kernels (*e*.*g*. linear, polynomial, radial basis function and sigmoidal) or any user-defined kernel. In this study, radial basis function kernel was selected and models generated using SVMlight Version 6.02 package which is available at http://svmlight.joachims.org/.

#### 2.4.2. L1-regularized logistic regression (LLR)

LLR is a rapid classifier having innate feature ranking capacity making it advantageous for optimal selection of information from features [20]. In this study, the L1-logreg classifier [32], an interior-point method for large-scale solver for L1-regularized logistic regression problems, was used to develop the prediction approach. The logistic model calculates the conditional probability of *b* ∈ {-1, 1} given *x* ∈ *R*_*n*_,

$$P(b|x)=\text{exp}(b(w^{T}x+v))/(1+\text{exp}(b(w^{T}x+v)))$$ (4)

where *x* denotes a vector of feature variables and *b* denotes the associated binary outcome (class). The model has parameters *w* ∈ *R*_*n*_ (the weight vector) and *v* ∈ *R* (the intercept); *w* ^*T*^ *x* + *v* = 0 defines the neutral hyper-plane in the data vector space.

The classifier locates the optimal model by maximizing the likelihood estimation from the observed examples, i.e. minimizing the average logistic loss:

$$minimize\;(1/m)\sum_{i=1}^{m}\text{log}(1+\text{exp}(-b_{i}(x_{i}^{T}w+v)))+\lambda \sum_{i=1}^{n}\left|w_{i}\right|$$ (5)

where *λ* > 0 is the regularization parameter, which is used to balance the average logistic loss and the size of the weight vector. The software package of L1-logreg classifier available at http://www.stanford.edu/~boyd/l1_logreg/, was used.

#### 2.4.3. Radial Basis Function Network (RBF)

RBF [33] is basically a class of single hidden layer feed forward neural network. The input nodes pass the input to the hidden nodes directly and the first layer connections are not weighted. In this study the QuickRBF package was used to construct RBFN classifiers with all training data as centers.

The general mathematical form of the output nodes in an RBF network is as follows:

$$g_{j}(x)=\sum_{i=1}^{k}w_{ji}\phi (||x-\mu_{i}||;\sigma_{i})$$ (6)

*g*_*j*_(*x*) is the function corresponding to the *j* ^*th*^ output node and is a linear combination of *k* radial basis functions ϕ() with center *μ*_*i*_ and bandwidth *r*_*i*_; The value of *r* can be estimated with data-driven methods. Also, *w*_*ji*_ is the weight associated with the link between the *j* ^*th*^ output node and the *i* ^*th*^ hidden node.

RBF networks exhibit the same properties as back-propagation networks such as generalization ability and robustness, and additionally, have the additional advantage of fast learning and ability to detect outliers during estimation. In this study, the RBF software package, quickRBF, was used with a bandwidth = 5, available at http://www.csie.ntu.edu.tw/~yien/quickrbf/.

### 2.5. Performance assessment

The following six measures were calculated to assess the prediction performance, using counts of true positives (TP; residues correctly predicted as catalytic), false positives (FP; residues incorrectly predicted as catalytic), true negatives (TN; residues correctly predicted as non-catalytic) and false negatives (FN; residues incorrectly predicted as non-catalytic).

Recall, or sensitivity, measures the proportion of the known catalytic residues that are correctly predicted as catalytic residues and is defined as *TP*/(*TP*+*FN*).

Precision measures the proportion of the residues predicted as catalytic that are known catalytic residues and is defined as *TP*/(*TP*+*FP*).

Specificity measures the proportion of the known non-catalytic residues that are correctly predicted as non-catalytic residues and is defined as *TN*/(*TN*+*FP*).

Accuracy is the proportion of the known residues that are correctly predicted in all predictions and is defined as (*TP*+*TN*)/(*TP*+*FN+TN+FP*).

Matthew's Correlation Coefficient (MCC) indicates the degree of the correlation between the actual and predicted classes of the residues. MCC values range between 1, where all the predictions are correct, and −1 where none are correct. MCC is defined as

$$((TP\times TN)-(FP\times FN))/\sqrt{((TP+FP)\times (TP+FN)\times (TN+FP)\times (TN+FP)).}$$

F-measure combines precision and recall into their harmonic mean, and is defined as 2 × (Pr*ecision* × Re*call*)/(Pr*ecision* + Re*call*).

10-fold cross-validation (10CV): The performance of the models trained on Dset650 was assessed using 10CV. The enzymes of training dataset were distributed into ten sets. One set of enzymes was taken out of the ten sets and was used as test dataset, and the remaining sets were used as training datasets. This process was repeated 10 times, and the final performance results were averaged over all the test results. To find the best threshold that can optimally classify each residue as catalytic or non-catalytic, predictions were made for each test data at a given threshold and the averaged performance measures calculated over the 10 iterations. Best models were selected based on best F-measure which is the balance point of sensitivity and specificity.

## Results and Discussion

Non-redundant, updated and diverse set of enzymes were used for training and independent testing using an optimally discriminative feature-classifier combination. The results obtained were analyzed and applied in real scenario. Further scope of improvement was explored using post-processing filtering.

### 3.1. Diversity in enzymes

**Fig 1:**
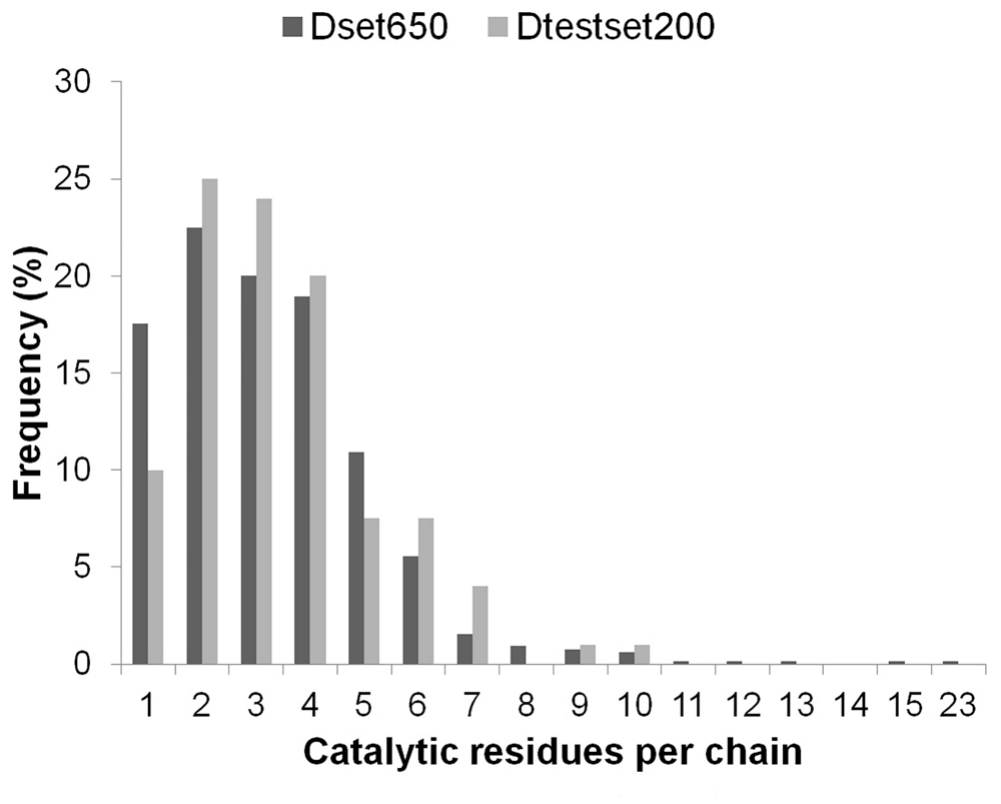
Number of catalytic residues per enzyme (chain) in the datasets used for training (Dset650) and testing (Dtestset200).

**Fig 2:**
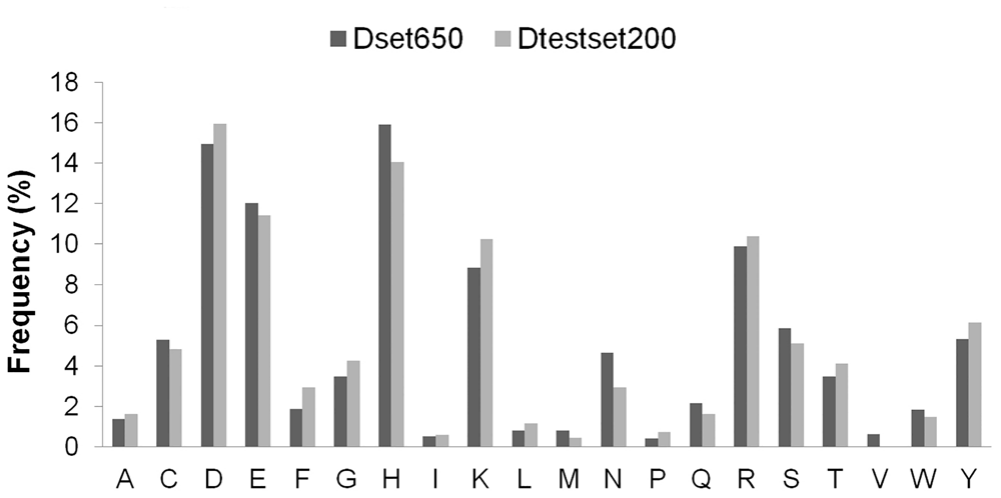
Type of amino acids constituting the catalytic residues of enzymes in Dset650 and Dtestset200.

**Fig 3:**
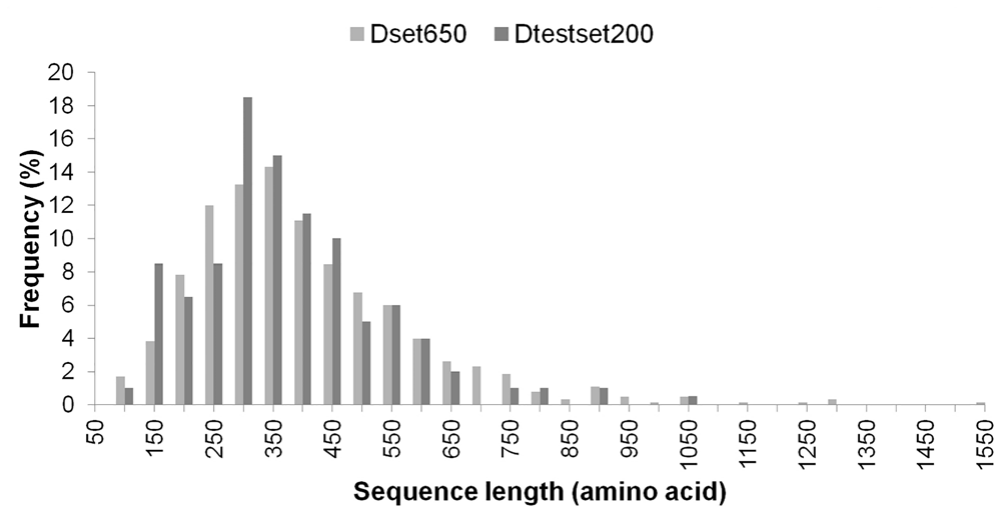
Sequence lengths (number of amino acids in a chain) of enzymes in Dset650 and Dtestset200.

**Fig 4:**
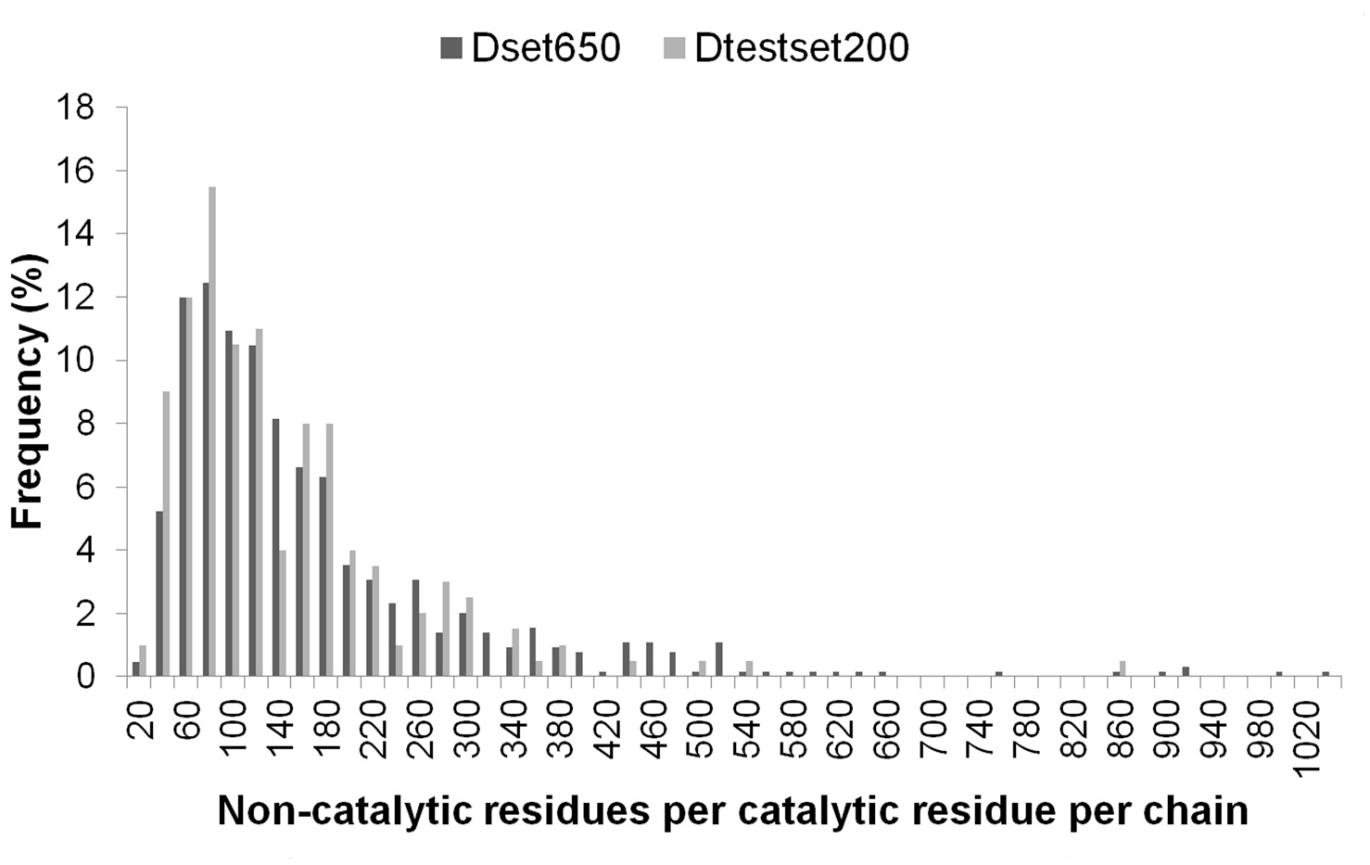
Non-catalytic residues per catalytic residue in enzymes of Dset650 and Dtestset200.

Representing the diversity in enzymes can help in generalizing the prediction approach. For addressing the computational challenge of accurate catalytic residue prediction, variations observed in enzymes such as in the number of catalytic residues per chain, type of constituting amino acids, sequence length and non-catalytic residues to catalytic residues per chain (NnCS) were analyzed. The number of catalytic residues occurring in an enzyme chain ranged from 1 to 23 in the Dset650 (total 2136) and 1 to 10 in the Dtestset200 (total 683), with most chains containing less than 10 catalytic residues as can be seen in Fig 1. Further observations into the amino acid composition of these residues in the datasets showed that there were representations of catalytic residues of different naturally occurring amino acid types. The amino acid distribution in groups of charged (HERKD), polar (QTSNCYW) and hydrophobic (GFLMAIPV) was observed to be 61.6%, 28.6%, and 9.8% for Dataset650 and 62.1%, 26.21%, and 11.7% for Dtestset200. The overall trend is shown in Fig 2, which is similar to amino acid distribution in catalytic residues reported in previous study [1]. A view into the sequence lengths of enzymes in the datasets shown in Fig 3 indicates that Dset650 comprises of enzymes with sequence length as small as 67 amino acids (aa) to as large as 1520 aa. And, Dtestset200 comprises of enzymes with sequence length as small as 62 aa to as large as 1023 aa. Therefore, the study was inclusive of variety in enzyme sequence length as can be seen in Fig 3. These diverse sequence lengths yielded vivid NnCS in datasets with Dset650 having NnCS ranging from over 14 to 1024 residues, with maximum number of enzymes having NnCS in between 80 and 100. In Dtestset200, NnCS ranged from 16 to 848, with a maximum number of enzymes having NnCS in between 80 and 100. This observation suggests the fact that enzyme data shows highly skewed distribution of catalytic residues as also reported in earlier studies (Fig 4).

With this preliminary idea on the diversity in enzyme representation, their biological properties were encoded from sequences. These were then used as an input into various classification models (described below) for obtaining the best possible catalytic residue prediction.

### 3.2. Developing classification models

Catalytic residues have salient physicochemical properties and evolutionary information that discriminate them from non-catalytic residues. These properties were encoded from the enzyme sequence as features of polarity index of amino acids; position specific scoring matrix and entropy information were used for prediction using three classifiers SVM, LLR and RBF as described in the Section 2.4. The so obtained prediction performance upon ten-fold cross validation of the classification models is shown in Table 1. Also shown, is the influence of using imbalanced training on prediction. As can be seen from the table, the models trained with balanced number of catalytic residues and non-catalytic residues show a MCC of 0.652 and F-measure of 83.1% (SVM). This is better than the performance obtained using LLR and RBF. The performance obtained using more than one number of non- catalytic residues per catalytic residues (*NnCS* > 1) while learning showed a dip in sensitivity with greater *NnCS* values. Since catalytic residues are very few among the many non-catalytic residues in the enzyme sequence, it is essential to have as many of them predicted. Although many non-catalytic residues may be falsely predicted along with them, a compromise at this stage in order to obtain better identification of both the classes (*i*.*e*., the catalytic and non-catalytic residues) for obtaining a better overall prediction performance would not be of much help to the end-user eventually. So, the issue of false prediction whatsoever obtained in the prediction process, was addressed separately. Thus, the best SVM models (training *NnCS* = 1 in a sliding window size = 15) were used to discriminate between a catalytic-residue and non-catalytic residue in the independent test-dataset.

**Table 1:** Ten-fold cross-validation results on training dataset Dset650.

*N*_*nCS*_ ^a^	Classifier^b^	RC (%)	PR (%)	SP (%)	AC (%)	MCC	FM (%)
1	LLR	84.7	79.4	80.0	81.4	0.629	81.9
	RBF	85.6	79.8	78.3	81.9	0.641	82.6
	SVM	85.6	80.8	79.5	82.5	0.652	83.1
2	LLR	73.2	76.0	88.4	83.3	0.622	74.6
	RBF	71.9	76.6	89.0	83.3	0.619	74.2
	SVM	75.4	76.7	88.4	84.1	0.642	76.0
3	LLR	58.6	71.7	94.2	87.1	0.571	64.4
	RBF	58.3	75.4	95.4	87.6	0.580	64.4
	SVM	62.0	72.4	94.0	87.6	0.595	66.7
4	LLR	48.6	70.1	96.5	89.7	0.528	57.3
	RBF	45.1	74.2	97.4	90.0	0.528	56.1
	SVM	54.3	71.0	96.3	90.3	0.567	61.5

^**a**^Number of non-catalytic residues per catalytic residue used while training.

^**b**^Models were developed using three classifiers (LLR: L1-regularized logistic regression; RBF: Radial basis function networks; SVM: Support vector machines). Performance assessment parameters described in the main text, RC: Recall, PR: Precision, SP: Specificity, AC: Accuracy, MCC: Mathews Correlation Coefficient, FM: F-measure.

**Fig 5:**
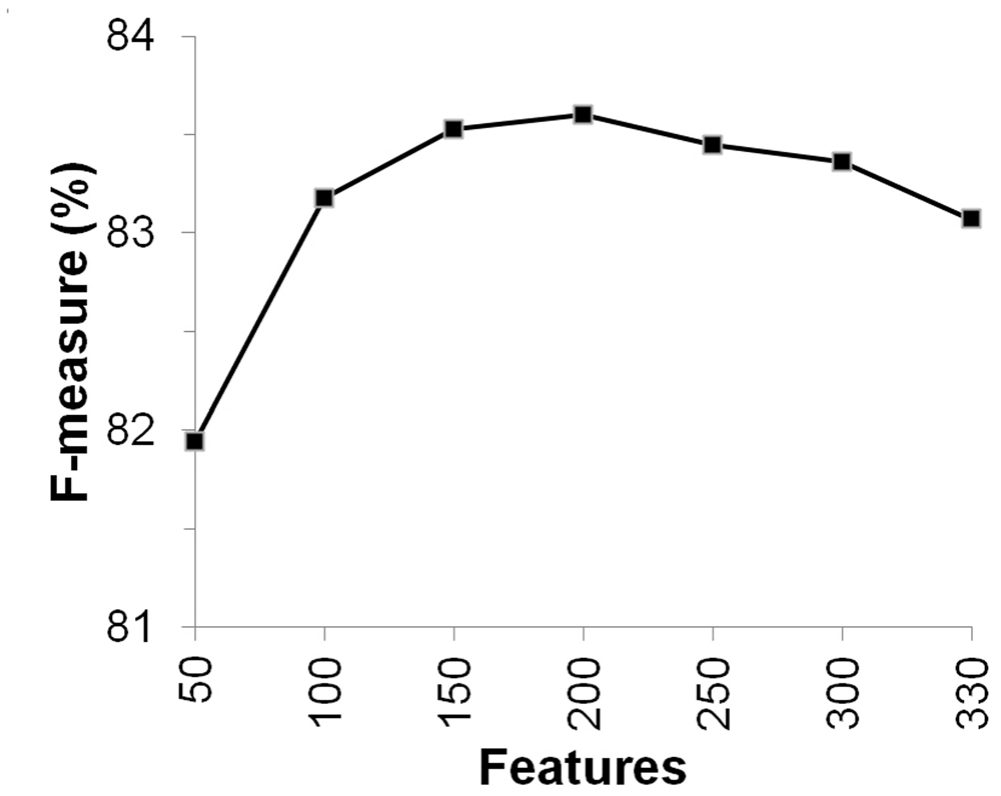
Selected number of features showing the best training performance on Dset650.

But before independent testing, any possible scope of reducing the feature dimension by including only the contributing features was explored. This was done by calculating the F-score as described in Section 2.3. Results suggested that 200 features of the 330 were sufficient to reach the optimal and best prediction performance in this study as shown in Fig 5. Of the 200 optimal features, 10 features were that of Polarity index, 175 of PSSM and 15 from EntWOP, details shown in Table 2. Altogether they helped reach a peak performance, F-measure of 83.6% and MCC of 0.665. The model obtained upon feature selection was used for independent testing as discussed in the next section.

**Table 2:** Summary of the selected features for independent testing on Dtestset200.

Position in window^a^	Feature composition and frequency of occurrence			
	Polarity Index	PSSM	EntWOP	Total
**-7**	0	5	1	6
**-6**	1	7	1	9
**-5**	1	13	1	15
**-4**	1	13	1	15
**-3**	1	14	1	16
**-2**	1	17	1	19
**-1**	1	14	1	16
**0**	1	20	1	22
**+1**	1	15	1	17
**+2**	0	16	1	17
**+3**	1	11	1	13
**+4**	0	8	1	9
**+5**	0	8	1	9
**+6**	0	7	1	8
+7	1	7	1	9

^a^Position in window where i denotes the central residue, i+n/i-n (n ϵ [0, 1, 2, 3, 4, 5, 6]) indicate the residues shifted from i towards the C-/N terminus.

### 3.3. Independent testing in predefined incrementally challenging prediction settings

Prediction of one catalytic residue from one non-catalytic residue in enzymes of the independent test dataset showed a Correlation Coefficient of 0.629 and an F-measure of 81.5%. Encouraged by the discriminative performance, these models were posed with incrementally challenging prediction scenarios. This was done by populating more numbers of non-catalytic residues among the catalytic residues (*NnCS*) in the setting for identification. Results indicated that the developed models were able to identify most of the catalytic residues despite their scanty occurrence in the entire sequence of amino acids. However, in addition to these true catalytic residues, some non-catalytic residues were mistakenly identified as catalytic. This, could also be noted in Table 3 as the decrease in precision from 81.2% (*NnCS* = 1) to 13.4% (*NnCS* = 30). However, as described earlier, our interest in accurately identifying as many catalytic residues is achieved with an overall sensitivity (81.9%) throughout the increments in *NnCS*. The specificity was also high (≥80%) throughout, implying most non-catalytic residues were also correctly identified. Balance assessment parameters of F-measure and MCC over the varied *NnCS* are shown in Table 3. Based on the obtained promising results, the performance of these models was explored in the real scenario (where all the catalytic residues and non-catalytic residues of a chain were included) and is described next.

**Table 3:** PINGU prediction on Independent test-dataset Dtestset200.

*N*_*nCS*_ ^a^	Performance assessment parameters^b^					
	RC (%)	PR (%)	SP (%)	AC (%)	MCC	FM (%)
1	81.9	81.2	81.0	81.5	0.629	81.5
6	81.9	40.1	79.6	79.9	0.473	53.8
12	81.9	26.3	80.9	81.0	0.392	39.8
18	81.9	20.1	81.9	81.9	0.347	32.3
24	81.9	15.9	81.9	81.9	0.309	26.6
30	81.9	13.4	82.1	82.1	0.285	23.0

^a^Number of non-catalytic residues per catalytic residue in the prediction scenario.

^b^Performance assessment parameters described in the main text, RC: Recall, PR: Precision, SP: Specificity, AC: Accuracy, MCC: Mathews Correlation Coefficient, FM: F-measure.

### 3.4. PINGU predictions in the real scenario

**Fig 6:**
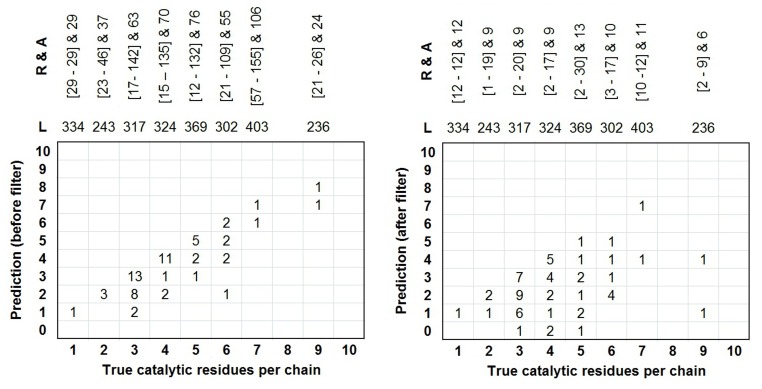
Insights into the prediction performance of PINGU in real scenario on 60 diverse enzymes. Abbreviations L corresponds to the average length of enzymes with a particular number of catalytic residues, R stands for the range of false positives identified in those enzymes and A is the average number of false positives.

**Fig 7:**
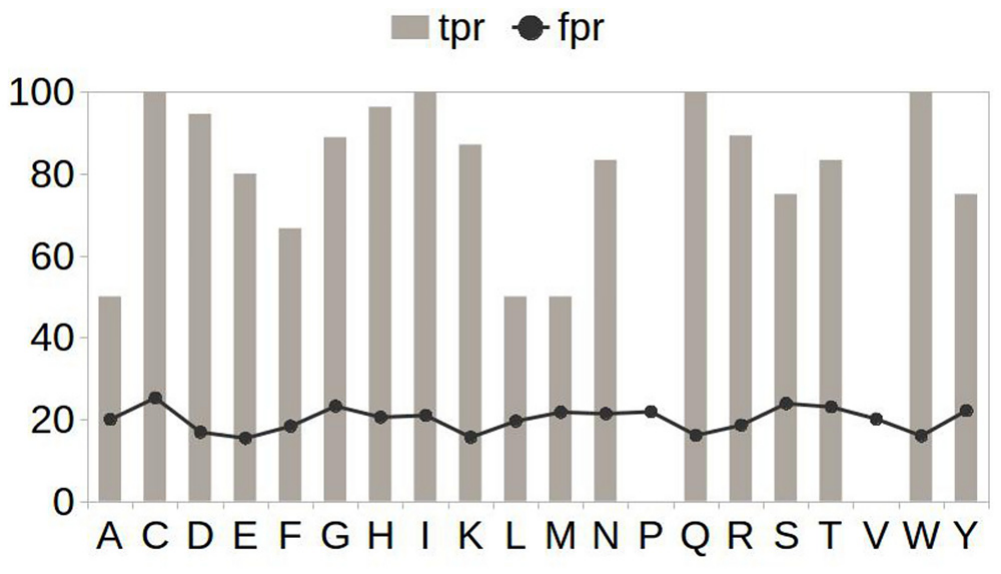
Exploring preference of amino acids during PINGU predictions in real scenario. Abbreviations tpr (Sensitivity): true positive rate; fpr (1—Specificity): false positive rate.

The best model obtained during the independent testing with (c = 50.0 and g = 0.08) was named PINGU: PredIction of eNzyme catalytic residues usinG seqUence information. Its discrimination power was tested on a pool of 60 enzymes with varied sequence lengths, number of non-catalytic residues per catalytic residue and enzyme class information. Performance assessment measures provided in Fig 6 show that PINGU on an average is 86.4% sensitive, 80.9% specific with a Correlation Coefficient of 0.203 and F-measure of 12.6%. The idea of having most catalytic residues predicted is preserved (detailed performance results are shown in S1 Table). Upon analysis, it was observed that some of these residues occurred in the enzyme subunit interface in homodimeric proteins with PDB ID 1BD0, 1DQR and 1Q6L (based on CSA database [8] records). For residue prediction, one each of their chains was included in this study and the residues that occurred on the subunit interface from the other chain were mapped on it. Based on the prediction obtained for the said enzymes, of the residues occurring on the subunit interface, the following were missed: 1DQR_A position 388 (Histidine) and 1Q6L_B positions 68 (Alanine) and139 (Arginine), with numbering based on PDB [25]. Further, whether the residues missed were solvent exposed (surface) or buried could provide an interesting understanding of the trend in prediction by PINGU. This was also explored using GETAREA web server available at http://curie.utmb.edu/getarea.html. It was found that nine of the 60 protein chains (1AOP_A, 1BD3_A, 1BIB_A, 1DJL_B, 1DQR_A, 1F6D_A, 1G99_A, 1KEZ_B, 2TDT_A) have one of their catalytic residues exposed and five others (1CVR_A, 1JHF_A, 1NSF_A, 1SES_B, 2A86_B) have two. PINGU was able to predict 15 out of 19 solvent exposed catalytic residues from above-mentioned 14 protein chains. Details of the performance of PINGU for these proteins are given in S1 Table. However, no specific bias or prediction trend was observed with regard to residues present on the subunit interface or those occurring on the surface of the enzymes. The false positives causing dip in predictor precision (Fig 7) is addressed separately by application of predicted ligand-binding information. This is described in the following.

### 3.5. Post-processing filter

Biological functions are broadly inclusive of specific biochemical activities and therefore may offer informative perspectives for relevant applications [2]. This idea is applied for investigating the scope of post-processing filtering [34] false PINGU predictions and enhancing its performance further. As catalytic residues are also ligand-binding residues in a broader perspective, predictions of a template-recognition based ligand binding site predictor, S-SITE [35], was integrated with PINGU hoping to improve its precision. So, only those predictions of PINGU that were also present in the set of predicted ligand binding residues obtained from S-SITE, were taken into consideration as catalytic and the remaining was filtered out and regarded as non-catalytic. Fig 6 shows the predictor performance before and after application of this post-processing filter. On an average, without much compromise in sensitivity, specificity or accuracy of the predictor, an overall improvement of 16% was observed in precision. This reflected in the balanced performance assessment parameters also, where an achievement of 20% rise in F-measure and 0.138 in MCC was marked (Details in S2 Table). The results of this attempt suggest that application of post-processing filter such as the one used in this study can be useful in obtaining accurate prediction of catalytic residues in real scenarios, with minimal false positives, which has been a challenge in biology.

### 3.6. Case study

**Fig 8:**
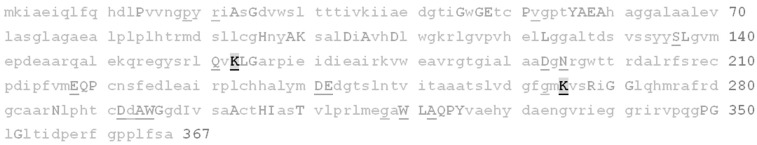
Prediction performance of PINGU on 4-hydroxyproline betaine 2-epimerase. The alphabets in upper case indicate PINGU predictions; alphabets underlined are predicted ligand binding residues; and those highlighted are catalytic residues.

PINGU predictions were so far generalized for a pool of enzymes. An insight into its working on an individual enzyme, 4-hydroxyproline betaine 2-epimerase [36], is provided here. The enzyme reportedly takes part in multiple biochemical reactions resulting in different biologically relevant functions in the catabolic pathway depending upon osmotic stress. The residues that directly take part in the catalytic activity, *i*.*e*., the catalytic residues, occur at position 163 (Lysine) and 265 (Lysine) in a sequence length of 367 residues. Prediction performance of PINGU is shown in Fig 8. As can be noted, among 353 residues (residues analysed excluding termini), the two catalytic residues were correctly predicted. Along with them, 52 non-catalytic residues were also predicted as catalytic. So, on an average, for every catalytic residue experimental validation for 26 other residues had to be done, if at all. To diminish the false positive rate, predicted ligand binding residue information for this enzyme was used. Consequently, it was observed that, of the 52 falsely predicted residues, 40 residues could be assigned as non-catalytic and for every catalytic residue, only 6 other residues needed scanning or validation, if at all, for experimental studies. These results suggest that PINGU with post-processing filtering can boost enzyme applications further.

## Conclusions

Accurate catalytic residues prediction has been a challenge because of their scanty occurrence along the enzyme length. Computational approaches with several developments reported over years could aid faster and accurate prediction of catalytic sites. But, unless the prediction is precise, experimental determination of the computationally identified putative catalytic residues can get highly resource intensive. To optimize the precision of prediction, this study attempts discrimination of catalytic residues with polarity index, PSSM and sequence conservation score in a support vector machine architecture. Further, it suggests use of post-processing filter such as ligand binding residue information for obtaining precise predictions which could be of help to the end-users. The supporting information and software are available at http://dx.doi.org/10.6084/m9.figshare.1492931. Overall, on account of its encouraging performance in prediction scenarios, PINGU is hoped to have eventual applications in boosting enzyme engineering and novel drug discovery process.

## Supporting Information

S1 Dataset Analyzed training (Dset650) and independent (Dtestset200) datasets. (PDF)

S1 Table PINGU predictions in real scenario on 60 diverse enzymes. (PDF)

S2 Table Prediction performance upon application of post-processing filter in real scenario on 60 diverse enzymes. (PDF)
