# Supplementary material for: PINGU: PredIction of eNzyme catalytic residues usinG seqUence information
Source: PLoS One. 2015 Aug 11;10(8):e0135122. doi: 10.1371/journal.pone.0135122 (PMC4532418; doi:10.1371/journal.pone.0135122)
Supplement: S1 Dataset — (PDF) [file pone.0135122.s001.pdf]

```
#Format:
#>PDB_Chain
#Sequence information with catalytic residues indicated in upper case


//Dset650//
>2C49_B
gkmekitcvghtaldyifnvekfpepntsiqipsarkyyggaaantavgikklgvnsellscvgydfknsgye
rylknldinisklyyseeeetpkawiftdkdnnqitfflwgaakhykelnppnfnteivhiatgdpefnlkca
kkaygnnlvsfdpgqdlpqyskemlleiiehtnflfmnkheferasnllnfeiddylervdalivtkgskgsv
iytkdkkieipcikagkvidptGAGDsyragflsayvkgydlekcgligaatasfvveakgcqtnlptwdkvv
erlekh
>1G79_A
msdndelqqiahlrreytkgglrrrdlpadpltlferwlsqaceakladptamvvatvdehgqpyqrivllkh
ydekgmvfytnlgsrkahqiennprvsllfpwhtlerqvmvigkaerlstlevmkyfhsrprdsqigawvskq
ssrisargileskflelkqkfqqgevplpsfwggfrvsleqiefwqggehRlhdrflyqrendawkidrlap
>1IDT_A
mdiisvalkrhstKafdaskkltpeqaeqiktllqyspsstnsqpwhfivasteegkarvaksaagnyvfner
Kmldashvvvfcaktamddvwlklvvdqedadgrfatpeakaandkgrkffadmhrkdlhddaewmakqvyln
vgnfllgvaalgldavpiegfdaaildaefglkekgytslvvvpvghhsvedfnatlpksrlpqnitltev
>1B5Q_A
atvgprvivvgagmsgisaakrlseagitdllileatdhiggrmhktnfaginvelganwvEgvnggkmnpiw
pivnstlklrnfrsdfdylaqnvykedggvydedyvqkrieladsveemgeklsatlhasgrddmsilamqrl
nehqpngpatpvdmvvdyykfdyefaepprvtslqntvplatfsdfgddvyfvadqrgyeavvyylagqylkt
ddksgkivdprlqlnkvvreikyspggvtvktednsvysadyvmvsaslgvlqsdliqfkpklptwkvraiyq
fdmavytkiflkfprkfwpegkgrefflyassrrgyygvwqefekqypdanvllvtvtdeesrrieqqsdeqt
kaeimqvlrkmfpgkdvpdatdilvprwwsdrfykgtfsnwpvgvnryeydqlrapvgrvyftgehtsehyng
yvhgaylsgidsaeilincaqkkmckyhvqgkyd
>1NLN_A
mgsseqelkaivkdlgcgpyflgtydkrfpgfvsphklacaivntagretggvHwmafawnprsktcylfEpf
gfsdqrlkqvyqfeyesllrrsaiasspdrcitlekstqsvQgpnsaaCglfccmflhafanwpqtpmdhnpt
mnlitgvpnsmlnspqvqptlrrnqeqlysflerhspyfrshsaqirsatsfchlknm
>1EB6_A
tevtdckgdaessltalsnaaklanqaaeaaesgdeskfeeyfkttdqqtrttvaerlravakeagstsggs
ttyhcndpygycepnvlaytlpskneiancdiYyselpplaqkchaqdqatttlhEfthapgvyqpgtedlgy
gydaatqlsaqdalnnadsyalyanaielkc
>1LUC_A
mkfgnflltyqppelsqtevmkrlvnlgkasegcgfdtvwlleHHftefgllgnpyvaaahllgatetlnvgt
aaivlptahpvrqaedvnlldqmskgrfrfgicrglydkdfrvfgtdmdnsralmdcwydlmkegfnegyiaa
dnehikfpkiqlnpsaytqggapvyvvaesasttewaaerglpmilswiinthekkaqldlynevatehgydv
tkidhclsyitsvdhdsnrakdicrnflghwydsyvnatkiFddsdqtkgydfnkgqwrdfvlkghkdtnrri
dysyeinpvgtpeeciaiiqqdidatgidniccgfeangseeeiiasmklfqsdvmpylkekq
>1YVE_L
attfdfdssvfkkekvtlsghdeyivrggrnlfpllpdafkgikqigvigwgsqapaqaqnlkdslteaksdv
vvkiglrkgsnsfaearaagfseengtlgdmwetisgsdlvlllisdsaqadnyekvfshmkpnsilglshgf
llghlqslgqdfpknisviavcpkgmgpsvrrlyvqgkevngaginssfavhqdvdgratdvalgwsialgsp
ftfattleqeyksdifgergillgavhgiveclfrrytesgmsedlaykntvecitgvisktistkgmlalyn
slseegkkdfqaaysasyypsmdilyecyedvasgseirsvvlagrrfyekeglpafpmgkidqtrmwkvgek
vrsvrpagdlgplypftagvyvalmmaqieilrkkghsyseiinesviEavdslnpfmhargvsfmvdncstt
arlgsrkwaprfdyilsqqalvavdngapinqdlisnflsdpvheaigvcaqlrpsvdisvtadadfvrpelr
qa
>1K30_A
mashsrkfldvrseeellscikketeagklppnvaagmeelyqnyrnaviesgnpkadeivlsnmtvaldril
ldvedpfvfsshhkairepfdyyifgqnyirplidfgnsfvgnlslfkdieeklqqghnvvlisnHqteaDpa
```

```
iislllektnpyiaentifvagdrvladplckpfsigrnlicvyskkhmfdipeltetkrkantrslkemall
lrggsqliwiapsggrdrpdpstgewypapfdassvdnmrrliqhsdvpghlfplallchdimpppsqveiei
gekrviafngaglsvapeisfeeiaathknpeevreayskalfdsvamqynvlktaisgkqglgastadvsls
qpw
>1AKM_A
sgfyhkhflklldftpaelnsllqlaaklkadkksgkeeakltgknialifekdstrtrcsfevaaydqgarv
tylgpsgsqighkesikdtarvlgrmydgiqyRgygqeivetlaeyasvpvwngltnefHptQlladlltmqe
hlpgkafnemtlvyagdarnnmgnsmleaaaltgldlrlvapqacwpeaalvtecralaqqnggnitltedva
kgvegadfiytDvwvsmgeakekwaeriallreyqvnskmmqltgnpevkflhClpafhddqttlgkkmaeef
glhggmevtdevfesaasivfdqaenRmhtikavmvatlsk
>1FO5_A
mskvkielftspmCPHCpaakrvveevanempdaveveyinvmenpqkameygimavptivingdvefigapt
kealveaikkrl
>1PZ3_A
matkkatmiiekdfkiaeidkriygsfiehlgravyggiyepghpqadengfrqdvielvkelqvpiirypgg
nfvsgynwedgvgpkeqrprrldlawksvetneiglnefmdwakmvgaevnmavnlgtrgidaarnlveycnh
psgsyysdlriahgykephkiktwclgnEmdgpwqighktaveygriaceaakvmkwvdptielvvcgssnrn
mptfaeweatvldhtydhvdyislhqyygnrdndtanylalslemddfirsvvaiadyvkakkrskktihlsf
dewnvwyhsneadkliepwtvapplledіynfedallvgcmlitlmkhadrvkiaclaqlvnviapimtekng
pawkqtiyypfmhasvygrgvalhpvisspkydskdftdvpylesiavyneekeevtifavnrdmedalllec
dvrsfedyrviehivlehdnvkqtnsaqsspvvphrngdaqlsdrkvsatlpklswnvirlgkr
>1CV2_A
mslgakpfgekkfieikgrrmayidegtgdpilfqhgnptssylwrnimphcaglgrliacdligmgdsdkld
psgperyayaehrdyldalwealdlgdrvvlvvhDWgsalgfdwarrhrervqgiaymEaiampiewadfpeq
drdlfqafrsqageelvlqdnvfveqvlpglilrplseaemaayrepflaagearrptlswprqipiagtpad
vvaiardyagwlsespipklfinaepgalttgrmrdfcrtwpnqteitvagaHfiqedspdeigaaiaafvrr
lrpa
>1DPG_A
vseiktlvtffggtgdlakrklypsvfnlykkgylqkhfaivgtarqalnddefkqlvrdcikdftddqaqae
afiehfsyrahdvtdaasyavlkeaieeaadkfdidgnrifymsvaprffgtiakylkseglladtgynrlmi
ekpfgtsydtaaelqndlenafddnqlfriDhylgkemvqniaalrfgnpifdaawnkdyiknvqvtlsevlg
veeragyydtagalldmiqnHtmqivgwlamekpesftdkdiraaknaafnalkiydeaevnkyfvraqygag
dsadfkpyleeldvpadsknntfiagelqfdlprwegvpfyvrsgkrlaakqtrvdivfkagtfnfgseqeaq
eavlsiiidpkgaielklnaksvedafntrtidlgwtvsdedkkntpepyermihdtmngdgsnfadwngvsi
awkfvdaisavytadkapletyksgsmgpeasdkllaangdawvfkg
>1GP5_A
mvaverveslaksgiisipkeyirpkeelesindvfleekkedgpqvptidlkniesddekirencieelkka
sldwgvmhlinhgipadlmervkkageeffslsveekekyandqatgkiqgygsklannasgqlewedyffhl
aypeekrdlsiwpktpsdyieatseyakclrllatkvfkalsvglglepdrlekevggleelllqmKinyypk
cpqpelalgveahtdvsaltfilhnmvpglqlfyegkwvtakcvpdsivmhigdtleilsngkyksilhrglv
nkekvriswavfceppkdkivlkplpemvsvespakfpprtfaqhiehklfgkeqeelvseknd
>1OQZ_A
eptstpqapiaaykprsneilwdgygvphiygvdapsafygygwaqarshgdnilrlygeargkgaeywgpdy
eqttvwlltngvperaqqwyaqqspdfranldafaaginayaqqnpddispdvrqvlpvsgadvvahahrlmn
flyvaspgrtlgegdppdladqgSnswavapgktangnalllqnpHlswttdyftyyeahlvtpdfeiygatq
iglpvirfafnqrmgitntVngmvgatnyrltlqdggylydgqvrpferpqasyrlrqadgttvdkpleirss
vhgpvferadgtavavrvagldrpgmleqyfdmitadsfddyeaalarmqvptfnivyadregtinysfngva
pkraegdiafwqglvpgdssrylwtethplddlprvtnppggfvqnsNdppwtptwpvtytpkdfpsylapqt
phslraqqsvrlmsenddltlerfmalqlshravmadrtlpdlipaalidpdpevqaaarllaawdreftsds
raallfeewarlfagqnfagqagfatpwsldkpvstpygvrdpkaavdqlrtaiantkrkygaidrpfgdasr
milndvnvpgaagygnlgsfrvftwsdpdengvrtpvhgEtwvamiefstpvrayglmsygnsrqpgtthysd
qiervsradfrellllrreqveaavqertpfnfkphhhhhh
>2ZAF_A
```

vdfklspsqlearrhaqafantvltkasaeystqkdqlsrfqatrpfyreavrhglikaqvpiplggtmeslv
hesiileelfavepatsitivatalglmpvilcdspslqekflkpfisgegeplaslmHSepngtanwlqkgg
pglqttarkvgnewvisgeklwpsnsggwdykgadlacvvcrvsddpskpqdpnvdpatqiavllvtretian
nkkdayqilgepelaghittsgphtrftefhvphenllctpglkaqglvetafamSaalvgamaigtaraafe
ealvfaksdtrggskhiiehqsvadklidckirletsrllvwkavttledealewkvklemamqtkiyttdva
vecvidamkavgmksyakdmsfprllnevmcyplfDggniglkrrqmqrvmaledyepwaatygs
>1JFL_A
mktigilggmgplataelfrriviktpakrdqehpkviifnnpqipdrtayilgkgedprpqliwtakrleec
gadfiimpCntahafvedirkaikipiismieetakkvkelgfkkagllattgtivsgvyekefskygveimt
ptedeqkdvmrgiyegvkagnlklgrelllktakileergaeciiagCtevsvvlkqddlkvplidpmdviae
vavkvalek
>1AMY_A
qvlfqgfnweswkhnggwynflmgkvddiaaagithvwlppasqsvaeqgympgrlydldaskygnkaqlksl
igalhgkgvkaiadivinhrtaehkdgrgiycifeggtpdarldwgphmicrddrpyadgtgnpdtgadfgaa
pdidhlnlrvqkelvewlnwlkadigfdgwrfDfakgysadvakiyidrsepsfavaEiwtslayggdgkpnl
nqdqhrqelvnwvdkvggkgpattfdfttkgilnvavegelwrlrgtdgkapgmigwwpakavtfvdnhdtgs
tqhmwpfpsdrvmqgyayilthpgtpcifydhffdwglkeeidrlvsvrtrhgihnesklqiieadadlylae
idgkvivklgprydvgnlipggfkvaahgndyavweki
>1MHY_D
maislatkaatnrapvgvepqevhkwlqsfnwdfkenrtkyptkyhmanetkeqfkviakeyarmeaakderq
fgtlldgltrlgagnkvhprwgetmkvisnflevgeynaiaasamlwdsataaeqkngylaqvldeirhthqC
afinhyyskhyhdpaghndarrtraigplwkgmkrvfadgfisgdavecsvnlqlvgeacfTnplivavtewa
sangdeitptvflsvetdelrhmangyqtvvsiandpasakflntdlnnafwtqqkyftpvlgylfeygskfk
vepwvktwnrwvsedwggiwigrlgkygvesprslrdakrdaywahhdlalaayamwplgfarlalpdeedqa
wfeanypgwadhygkifnewkklgyedpksgfipyqwllanghdvyidrvsqvpfipslakgtgslrvhefng
kkhsltddwgerqwlieperyechnvfeqyegrelseviaeghgvrsdgktliaqphtrgdnlwtledikrag
cvfpdplakf
>3QXS_A
fqghmlfisatntnagKttcarllaqycnacgvktillKpieTgvndainhssdahlflqdnrlldrsltlkd
isfyryhkvsapliaqqeedpnapidtdnltqrlhnftktydlvivegagglcvpitleenmldfalklkakm
llishdnlglindcllndfllkshqldykiainlkgnntafhsislpyielfntrsnnpivifqqslkvlmsf
alkgs
>1OJ4_A
mrtqwpspaKlnlflyitgqradgyhtlqtlfqfldygdtisielrddgdirlltpvegvehednlivraarl
lmktaadsgrlptgsganisidkrlpmggglgggssnaatvlvalnhlwqcglsmdelaemgltlgaDvpvfv
rghaafaegvgeiltpvdppekwylvahpgvsiptpvifkdpelprntpkrsietllkcefsndceviarkrf
revdavlswlleyapsrltgtgacvfaefdtesearqvleqapewlngfvakgvnlsplhraml
>3D3L_B
wtlkagalemalkrvytllsswncledfdqifwgqksalaekvrqcwqddelfsyqflnganpmllrrstslp
srlvlpsgmeelraqlekelqngslfeadfilldgipaqylaaplvmlkmepngklqpmviqiqppnpssptp
tlflpsdpplawllakswvrnsdfqlheiqyhllnthlvaeviavatmrclpglhpifkfliphigghvqllr
raaaqltycslcppddladrgllglpgalyahdalrlweiiaryvegivhlfyqrddivkgdpelqawcreit
evglcqaqdrgfpvsfqsqslchfltmcvftctaqhaaiNqgqldwyawvpnapctmrmppttkedvtmat
vmgslpdvrqaclqmaiswhlsrmvplghhkekyfsgpkpkavlnqfrtdleklekeitarneqldwpyeylk
psciensvt
>1D4A_A
vgrralivlahsertsfnyamkeaaaaalkkkgwevvesdlyamnfnpiisrkditgklkdpanfqypaesvl
aykeghlspdivaeqkkleaadlvifqfplqwfgvpailkgwfervfigefaytyaamydkgpfrskkavlsi
ttGgsgsmYslqgiHgdmnvilwpiqsgilhfcgfqvlepqltysightpadariqilegwkkrleniwdetp
lyfapsslfdlnfqagflmkkevqdeeknkkfglsvghhlgksiptdnqikark
>1C0K_A
lmmhsqkrvvvlgsgviglssalilarkgysvhilardlpedvssqtfaspwaganwtpfmtltdgprqakwe
estfkkwvelvptghamwlkgtrrfaqnedgllghwykditpnyrplpssecppgaigvtydtlsvhapkycq
ylarelqklgatferrtvtsleqafdgadlvvnatglgaksiagiddqaaepirgqtvlvkspckrctmdssd

paspayiiprpggevicggtygvgdwdlsvnpetvqrilkhclrldptissdgtiegievlrhnvglrparrg
gprveaerivlpldrtksplslgrgsaraakekevtlvhaygfsSagyqqswgaaedvaqlvdeafqryhg
>1FNB_A
qiasdveapppapakvekhskkmeegitvnkfkpktpyvgrcllntkitgddapgetwhmvfshegeipyreg
qsvgvipdgedkngkphklrlySiassalgdfgdaksvslcvkrliytndagetikgvcsnflcdlkpgaevk
ltgpvgkemlmpkdpnatiimlgtgtgiapfrsflwkmffekhddykfnglawlflgvptssslly keefekm
kekapdnfrldfavsreqtnekgekmyiqtrmaqyavelwemlkkdntyvymCglkgmekgiddimvslaaae
gidwieykrqlkkaeqwnvEvy
>1QDL_A
amevhpisefaspfevfkcierdfkvagllesiggpqykarysviawstngylkihddpvnilngylkdlkla
dipglfkggmigyisydavrfwekirdlkpaaedwpyaefftpdniiiydhnegkvyvnadlssvggcgdige
fkvsfydeslnknsyerivsesleyirsgyifqvvlsrfyryifsgdplriyynlrrinpspymfylkfdeky
ligsspellfrvqdnivetypiagtrprgadqeedlklelelmnsekdkaehlmlvdlarndlgkvcvpgtvk
vpelmyvekyshvqHivskvigtlkkkynalnvlsatfpagtvsgapkpmamniietleeykrgpyagavgfi
sadgnaefaiairtaflnkellrihagagivydsnpeseyfetehklkalktaigvr
>1QDL_B
mdltliidnydsfvyniaqivgelgsypivirndeisikgieridpdrliispgpgtpekredigvsldviky
lgkrtpilgvClghqaigyafgakirrarkvfhgkisniilvnnsplslyygiakefkatryhslvvdevhrp
livdaisaedneimaihheeypiygvqfHpEsvgtslgykilynflnrv
>1V04_A
makltaltllgmglalfdrqkssfqtrfnvhrevtpvelpncnlvkgidngsedleilpnglafissglkypg
imsfdpdksgkillmdlnekepavseleiigntldissfnpHgistfidddntvyllvvnHpgssstvevfkf
qeeeksllhlktirhkllpsvndivavgpehfyatndhyfidpylkswemhlglawsfvtyyspndvrvvaeg
fdfanginispdgkyvyiaellahkihvyekhanwtltplrvlsfdtlvdnisvdpvtgdlwvgchpngmrif
fydaenppgsevlriqdilseepkvtvvyaengtvlqgstvaavykgklligtvfhkalycdl
>1KZH_A
mntslfkqerqkyipklpnilkkdfnnislvygenteaiqdrqalkeffkntyglpiisftegesslsfskal
nigiilsgGpapgghnvisgvfdaikkfnpnsklfgfkggplgllendkielteslinsyrntggfdivssgR
tkieteehynkalfvakennlnaiiiiggddsntnaailaeyfkkngeniqvigvpKTiDadlrndhieisfg
fdsatkiyselignlcrdamstkkywhfvklmgrsashvalecalkthpnicivseevlakkktlseiidemv
svilkrslngdnfgvvivpegliefipevkslmlelcdifdknegefkglniekmkeifvaklsdymkgvyls
lplfiqfeliksilerdphgnfnvsrvpteklfiemiqsrlndmkkrgeykgsftpvdhffgyegrsafpsnf
dsdycyslgynavvlilngltgymsciknlnlkptdwiaggvpltmlmnmeerygekkpvikkalvdlegrpf
kefvknrdkwalnnlylypgpvqyfgsseivdeitetlklelfk
>1D6O_A
gvqvetispgdgrtfpkrgqtcvvhytgmledgkkfDssrdrnkpfkfmlgkqevIrgweegvaqmsvgqrak
ltispdyaYgatghpgiipphatlvfdvellkle
>1FRO_A
aepqppsggltdeaalsccsdadpstkdfllqqtmlrvkdpkksldfytrvlgmtliqkcdfpimkfslyfla
yedkndipkekdekiawalsrkatlelthnwgteddetqsyhngnsdprgfghigiavpdvysackrfeelgv
kfvkkpddgkmkglafiqdpdgywiEilnpnkmatlm
>1L7D_A
mkiaipkerrpgedrvaispevvkklvglgfeviveqgagvgasitddaltaagatiastaaqalsqadvvwk
vqrpmtaeegtdevalikegavlmchlgaltnrpvvealtkrkitayamelmpRisraQsmDilSsqsnlagy
ravidgayefarafpmmmtaagtvpparvlvfgvgvaglqaiatakrlgavvmatdvraatkeqveslggkfi
tvddeamktaetaggyakemgeefrkkqaeavlkelvktdiaittalipgkpapvliteemvtkmkpgsviid
laveaggncplsepgkivvkhgvkivghtnvpsrvaadasplfaknllnfltphvdkdtktlvmkledetvsg
tcvtrdgaivhpaltgqga
>3KVL_A
fyaehlmptlqglldpesahrlavrftslgllprarfqdsdmlevrvlghkfrnpvgiaagfdkhgeavdgly
kmgfgfveiGsvtpkpqegnprprvfrlpedqavinrygFnshglsvvehrlrarqqkqakltedglplgvnl
gknktsvdaaedyaegvrvlgpladylvvnvsSpnTaglrslqgkaelrrlltkvlqerdglrrvhrpavlvK
iapdltsqdkediasvvkelgidglivtnttvsrpaglqgalrsetgglsgkplrdlstqtiremyaltqgrv
piigvggvssgqdalekiragaslvqlytaltfwgppvvgkvkreleallkeqgfggvtdaigadhr

>1VAO_A
msktqefrpltlppklslsdfnefiqdiirivgsenvevisskdqivdgsymkpththdphhvmdqdyflasa
ivaprnvadvqsivglankfsfplwpisigrnsgYggaaprvsgsvvldmgknmnrvlevnvegaycvvepgv
tyhdlhnyleannlrdklwldvpDlgggsvlgnavergvgytpygdhwmmhsgmevvlangellrtgmgalpd
pkrpetmglkpedqpwskiahlfpygfgpyidglfsqsnmgivtkigiwlmpnprgyqsylitlpkdgdlkqa
vdiirplrlgmalqnvptirhilldaavlgdkrsyssrteplsdeeldkiakqlnlgrwnfygalygpepirr
vlwetikdafsaipgvkfyfpedtpensvlrvrdktmqgiptydelkwidwlpngaHlffspiakvsgedamm
qyavtkkrcqeagldfigtftvgmremhhivcivfnkkdliqkrkvqwlmrtliddcaangwgeYRthlafmd
qimetynwnnssflrfnevlknavdpngiiapgksgvwpsqyshvtwkl
>1QFN_A
mqtvifgRsGcpYsvraKdlaeklsnerddfqyqyvdiraegitkedlqqkagkpvetvpqifvdqqhiggYt
dfaawvkenlda
>1PEO_A
etmdyhalnamlnlydkaghiqfdkdqqaidaffathvrphsvtfasqherlgtlvregyyddavlarydraf
vlrlfehahasgfrfqtflgawkfytsytlktfdgkrylehfedrvtmvaltlaqgdetlatqltdemlsgrf
qpatptflncgkqqrgelvsCfllriednmesigravnsalqlskrgggvafllsnlreagapikrienqssg
vipvmkmledafsyanqlgarqgagavylhahhpdilrfldtkrriktlslgvvipditfrlakenaqmalfs
pydiqrrygkpfgdiaiserydeliadphvrktyinardffqtlaeiqfesgypyimfedtvnranpiagrin
msNlCsEilqvnsasryddnldythighdisCnlgslniahvmdspdigrtvetairgltavsdmshirsvps
iaagnaashaiglgqmnlhgylaregiaygspealdftnlyfytitwhavhtsmrlarergktfagfaqsrya
sgdyftqylqddwqpktakvralfarsgitlptremwlklrddvmrygiynqnlqavpptgsisyinhatssi
hpivakieirkegktgrvyypapfmtnenldmyqdaydigpekiidtyaeatrhvdqglsltlffpdtattrd
inkaqiyawrkgikslyyirlrql
>1COY_A
apsrtladgdrvpalvigsgyggavaalrltqagiptqivemgrswdtpgsdgkifcgmlnpdkrsmwladkt
dqpvsnfmgfginksidryvgvldserfsgikvyqgrgvgggslvnggmavtpkrnyfeeilpsvdsnemynk
yfprantglgvnnidqawfestewykfartgrktaqrsgfttafvpnvydfeymkkeaagqvtksglggeviy
gnnagkksldktylaqaaatgkltittlhrvtkvapatgsgysvtmeqideqgnvvatkvvtadrvffaagsv
gtskllvsmkaqghlpnlssqvgegwgnngnimvgranhmwdatgskqatiptmgidnwadptapifaEiapl
pagletyvslylaitknperarfqfnsgtgkvdltwaqsqnqkgidmakkvfdkinqkegtiyrtdlfgvyyk
twgddftyHplggvllnkatdnfgrlpeypglyvvdgslvpgnvgvNpfvtitalaernmdkiissdiq
>1P3D_A
mkhsheeirkiipemrrvqqihfigiggagmsgiaeillnegyqisgsdiadgvvtqrlaqagakiyighaee
hiegasvvvvssaikddnpelvtskqkripviqraqmlaeimrfrhgiavagthgKttttamismiytqakld
ptfvngglvksagknahlgasryliaeadesdasflhlqpmvsvvtnmepdhmdtyegdfekmkatyvkflhn
lpfyglavmcaddpvlmelvpkvgrqvitygfseqadyriedyeqtgfqghytvicpnnerinvllnvpgkhn
alnataalavakeegianeailealadfqgagrrfdqlgefirpngkvrlvddyghhptevgvtikaaregwg
dkrivmifqphrysrtrdlfddfvqvlsqvdalimldvyaageapivgadskslcrsirnlgkvdpilvsdts
qlgdvldqiiqdgdlilaqggagsvskisrglaeswkn
>1UAG_A
adyqgknvviiglgltglscvdfflargvtprvmdtrmtppgldklpeaverhtgslndewlmaadlivaspg
ialahpslsaaadagieivgdielfcreaqapivaitgsngKstvttlvgemakaagvnvgvggNiglpalml
lddecelyvlelssfqlettsslqavaatilnvtedHmdrypfglqqyraaklriyenakvcvvnaddaltmp
irgadercvsfgvnmgdyhlnhqqgetwlrvkgekvlnvkemklsgqhnytnalaalalaladaaglprasslka
lttftglphrfevvlehngvrwindskatnvgsteaalnglhvdgtlhlllggdgksadfsplarylngdnvr
lycfgrdgaqlaalrpevaeqtetmeqamrllaprvqpgdmvllspacasldqfknfeqrgnefarlakelg
>1MHT_A
mieikdkqltglrfidlfaglggfrlalescgaecvysnewdkyaqevyemnfgekpegditqvnektipdhd
ilcagfpCqafsisgkqkgfedsrgtlffdiarivrekkpkvvfmEnvknfashdngntlevvkntmneldys
fhakvlnaldygipqkreriymicfrndlniqnfqfpkpfelntfvkdlllpdsevehlvidrkdlvmtnqei
eqttpktvrlgivgkggqgeriystrgiaitlsayggifaktggylvngktrklhprecarvmgypdsykvh
pstsqaykqfgnsvvinvlqyiaynigsslnfkpy
>1ULA_A

mengytyedykntaewllshtkhrpqvaiicgsglggltdkltqaqifdyseipnfprstvpghagrlvfgfl
ngracvmmqgrfHmyEgyplwkvtfpvrvfhllgvdtlvvtnaagglnpkfevgdimlirdhinlpgfsgqnp
lrgpnderfgdrfpamsdaydrtmrqralstwkqmgeqrelqegtyvmvagpsfetvaecrvlqklgadavgm
stvpevivarhcglrvfgfslitNkvimdyeslekanheevlaagkqaaqkleqfvsilmasiplpdkas
>1JVZ_A
qapiaaykprsneilwdgygvphiygvdapsafygygwaqarshgdnilrlygeargkgaeywgpdyeqttvw
lltngvperaqqwyaqqspdfranldafaaginayaqqnpddispevrqvlpvsgadvvaHahrlnflyvasp
grtlg
>1S76_D
mntiniakndfsdielaaipfntladhygerlareqlalehesyemgearfrkmferqlkagevadnaaakpl
ittllpkmiarindwfeevkakrgkrptafqflqeikpeavayitikttlacltsadnttvqavasaigraie
dearfgrirdleakhfkknveeqlnkrvghvykkafmqvveadmlskgllggeawsswhkedsihvgvrciem
liestgmvslhrqnagvvgqdsetielapeyaeaiatragalagispmfqpcvvppkpwtgitgggywangrr
plalvrthskkalmryedvympevykainiaqntawkinkkvlavanvitkwkhcpvedipaiereelpmkpe
didmnpealtawkraaaavyrkdkarksrrislefmleqankfanhkaiwfpynmdwrgrvyavsmfnpqgnd
mtkglltlakgkpigkegyywlkihgancagvdkvpfperikfieenhenimacaksplentwwaeqdspfcf
lafcfeyagvqhhglsyncslplafdgscsgiqhfsamlrdevggravnllpsetvqdiygivakkvneilqa
daingtdnevvtvtdentgeisekvklgtkalagqwlaygvtrsvtKrsvmtlaygskefgfrqqvledtiqp
aidsgkglmftqpnqaagymakliwesvsvtvvaaveamnwlksaakllaaevkdkktgeilrkrcavhwvtp
dgfpvwqeykkpiqtrlnlmflgqfrlqptintnkdseidahkqesgiapnfvhsqdgshlrktvvwahekyg
iesfalihdsfgtipadaanlfkavretmvdtyescdvladfydqfadqlhesqldkmpalpakgnlnlrdil
esdfafa
>1AJS_A
appsvfaevpqaqpvlvfkliadfredpdprkvnlgvgayrtddcqpwvlpvvrkveqriannsslnheylpi
lglaefrtcasrlalgddspalqekrvggvqslggtgalrigaeflarwyngtnnkdtpvyvssptWenhngv
fttagfkdirsyrywdtekrgldlqgflsdlenapefsifvlhacahnptgtdptpeqwkqiasvmkrrflfp
ffDsayqgfasgnlekdawairyfvsegfelfcaqsfsKnfglynervgnltvvakepdsilrvlsqmqkivr
vtwsnppaqgarivartlsdpelfhewtgnvktmadrilsmrselrarlealktpgtwnhitdqigmfsftgl
npkqveylinqkhiyllpsgrinmcglttknldyvatsiheavtkiq
>2XFN_A
nkcdvvvvgggisgmaaakllhdsglnvvvleardrvggrtytlrnqkvkyvdlggsyvgptqnrilrlakel
gletykvneverlihhvkgksypfrgpfppvwnpityldhnnfwrtmddmgreipsdapwkaplaeewdnmtm
kelldklcwtesakqlatlfvnlcvtaethevsalwflwyvkQcggttriisttnggqerkfvggsgqvseri
mdllgdrvklerpviyidqtrenvlvetlnhemyeakyvisaipptlgmkihfnpplpmmrnqmitrvplgsv
iKcivyykepfwrkkdycgtmiidgeeapvaytlddtkpegnyaaimgfilahkarklarltkeerlkklcel
yakvlgslealepvhyeeknwceeqysggcyttyfppgiltqygrvlrqpvdriyfagtetathwsgymegav
eageraareilhamgkipedeiwqsepesvdvpaqpitttflerhlpsvpgllrligltti
>1FLG_A
kdvtwedianddkttgdvlqygmgthaqrwsplkqvnadnvfkltpawsysfgdekqrgqesqaivsdgviyv
tasysrlfaldaktgkrlwtynhrlpddirpccdvvnrgaaiygdkvffgtldasvvalnkntgkvvwkkkfa
dhgagytmtgaptivkdgktgkvllihgssgdefgvvgrlfardpdtgeeiwmrpfveghmgrlngkdstvtg
dvkapswpddrnsptgkveswshgggapwqsasfdaetntiivgagnpgpwntwartakggnphdydslytsg
qvgvdpssgevkwfyqhtpndawDfsgnnelvlfdykakdgkivkatahadrngffyvvdrsngklqnafpfv
dnitwashidlktgrpveregqrpplpepgqkhgkavevsppflggknwnpmaysqdtglfyvpanhwkedyw
teevsytkgsaylgmgfrikrmyddhvgslramdpvsgkvvwehkehlplwagvlatagnlvftgtgdgyfka
fdaksgkelwkfqtgsgivsppitweqdgeqylgvtvgyggavplwggdmadltrpvaqggsfwvfklpsw
>2PIA_A
ttpqedgflrlkiaskekiardiwsfeltdpqgaplppfeagaNltvavpngsrrtySlcndsqernryviav
krdsngrggsisfiddtsegdavevslprnefpldkraksfilvaggigitpmlsmarqlraeglrsfrlyyl
trdpegtaffdeltsdewrsdvkihhdhgdptkafdfwsvfekskpaqhvycCgpqalmdtvrdmtghwpsgt
vhfEsfgatntnarentpftvrlsrsgtsfeipanrsilevlrdanvrvpsscesgtcgscktalcsgeadhr
dmvlrddekgtqimvcvsraksaelvldl
>5COX_A

```
anpccsnpcqnrgecmstgfdqykcdctrtgfygencttpefltriklllkptpntvhyilthfkgvwnivnn
ipflrslimkyvltsrsylidspptynvhygyksweafsnlsyytralppvaddcptpmgvkgnkelpdskev
lekvllrrefipdpqgsnmmfaffaQhftHqffktdhkrgpgftrglghgvdlnhiygetldrqhklrlfkdg
klkyqviggevypptvkdtqvemiypphipenlqfavgqeVfglvpglmmyatiwlrehqrvcdilkqehpew
gdeqlfqtskliligetikiviedyvqhlsgyhfklkfdpellfnqqfqyqnriasefntlYhwhpllpdtfn
iedqeysfkqflynnsillehgltqfvesftrqiagrvaggrnvpiavqavakasidqsremkyqslneyrkr
fslkpytsfeeltgekemaaelkalysdidvmelypallvekprpdaifgetmvelgapfslkglmgnpicsp
qywkpstfggevgfkiintasiqslicnnvkgcpftsfnvqdpqptktatinasashsrlddinptvlikrrs
tel
>3MZ0_A
slrigvigtgaigkehinritnklsgaeivavtdvnqeaaqkvveqyqlnatvypnddslladenvdavlvts
wgpahessvlkaikaqkyvfceKplattaegcriveeeikvgkrlvqvgfrrydsgyvqlkealdnhvigepl
ihcahrnptvgdnyttdavvdtlvheidvlhwlvnddyesvqviypkksknalphlkdpqivvietkggivin
aeiyvnckygydiqceivgedgiiklpepssislrkegrfstdildwqrrfvaaydveiqdfidsiqkkgevs
gptawdgyiaavttdacvkaqesgqkekvelkekpefyq
>1GEQ_A
mfkdgslipyltagdpdkqstlnfllaldeyagaiElgipfsdpiaDgktiqeshyralkngfklreafwivk
efrrhsstpivlmtyynpiyragvrnflaeakasgvdgilvvdlpvfhakefteiareegiktvflaapntpd
erlkviddmttgfvYlvslygttgareeipktaydllrrakricrnkvavgfgvskrehvvsllkegangvvv
gsalvkiigekgreateflkkkveellgi
>1LXA_A
midksafvhptaiveegasiganahigpfcivgphveigegtvlkshvvvnghtkigrdneiyqfasigevnq
dlkyageptrveigdrnriresvtihrgtvqgggltkvgsdnllminahiaHdctvgnrcilannatlaghvs
vddfaiiggmtavhqfciigahvmvggcsgvaqdvppyviaqgnhatpfgvnieglkrrgfsreaitairnay
kliyrsgktldevkpeiaelaetypevkaftdffarstrglir
>1PYA_B
ftgvqgrvigydilrspevdkakplftetqwdgselpiydakplqdalveyfgteqdrrhypapgsfivcank
gvtaerpkndadmkpgqgygvwsaiaisfakdptkdssmFvEdagvwetpnedelleylegrrkamaksiaec
gqdahasfesswigfaytmmepgqignaitvapyvslpidsipggsiltpdkdmeimenltmpewlekmgyks
lsannalky
>1PYA_C
seldaklnklgvdriaispykqwtrgymepgnigngyvtglkvdagvrdksdddvldgivsYdraetknayig
qinmttaS
>2PTQ_B
melssltavspvdgrygdkvsalrgifseygllkfrvqvevrwlqklaahaaikevpafaadaigyldaivas
fseedaariktierttnhdvkaveyflkekvaeipelhavsefihfactsedinnlshalmlktardevilpy
wrqlidglkdlavqyrdipllsrtngqpatpstigkemanvayrmerqyrqlnqveilgkingavgnynahia
aypevdwhqfseefvtslgiqwnpyttqiephdyiaelfdcvarfntilidfdrdvwgyialnhfkqktiage
igsstmphKvnpidfEnsegnlglsnavlqhlasklpvsrwqrdltdstvlrnlgvgigyaliayqstlkgvs
klevnrdhlldeldhnwevlaepiqtvmrrygiekpyeklkeltrgkrvdaegmkqfidglalpeeekarlka
mtpanyigraitmvdelkhh
>1QGN_A
makavdaaaaaaaaiapvdttvvnedvalvenetcndqnvqfdslpsmkyasflnsdgsvaihagerlgrgiv
tdaittpvvntsayffnktselidfkekrrasfeygrygnpttvvleekisalegaestllmasgmcastvml
lalvpagghivtttdcYrktrifietilpkmgitatvidpadvgalelalnqkkvnlfftesptnpflrcvdi
elvsklchekgalvciDgtfatplnqkalalgadlvlhsatKflgghndvlagcisgplklvseirnlhhilg
galnpnaayliirgmktlhlrvqqqnstalrmaeileahpkvrhvyypglqshpehhiakkqmtgfggavsfe
vdgdllttakfvdalkipyiapsfggcesivdqpaimsywdlsqsdrakygimdnlvrfsfgvedfddlkadi
lqaldsi
>1UAQ_A
mvtggmaskwdqkgmdiayeeaalgykeggvpiggclinnkdgsvlgrghnmrfqkgsatlhgEistlencgr
legkvykdttlyttlSpCdmctgaiimygiprcvvgenvnfkskgekylqtrghevvvvdderckkimkqfid
erpqdwfedige
>1PWH_D
```

```
maaqeslhvktplrdsmalskvagtsvflkmdssqpsgsfKirgighlckmkakqgckhfvcssagnagmata
yaarrlglpativvpsttpaltierlknegatvevvgemldeaiqlakaleknnpgwvyispfddpliweght
slvkelketlsakpgaivlsvggggllcgvvqglrevgwedvpiiametfgahsfhaavkegklvtlpkitsv
akalgvntvgaqtlklfyehpifsevisdqeavtaiekfvddekilvepacgaalaavysgvvcrlqaearlq
tplaslvvivcggsnislaqlqalkaqlglnellk
>1E6E_A
stqeqtpqicvvgsgpagfytaqhllkhhsrahvdiyekqlvpfglvrfgvapdHpevknvintftqtarsdr
cafygnvevgrdvtvqelqdayhavvlsygaedhqaldipgeelpgvfsarafvgwynglpenrelapdlscd
tavilgqgnvalDvarilltppdhlektditeaalgalrqsrvktvwivgrrgplqvaftikelremiqlpgt
rpmldpadflglqdrikeaarprkrlmelllrtatekpgveeaarrasasrawglrffrspqqvlpspdgrra
agirlavtrlegigeatravptgdvedlpcglvlssigyksrpidpsvpfdpklgvvpnmegrvvdvpglycs
gwvkrgptgvITttmtdsfltgqillqdlkaghlpsgprpgsafikalldsrgvwpvsfsdwekldaeevsrg
qasgkprekllldpqemlrllgh
>1ZYM_A
misgilaspgiafgkalllkedeividrkkisadqvdqeverflsgrakasaqletiktkagetfgeekeaif
eghimlledeeleqeiialikdkhmtadaaaheviegqasaleelddeylkeraadvrdigkrllrnilglki
idlsaiqdevilvaadltpseTaqlnlkkvlgfitdaggrtsHtsimarslelpaivgtgsvtsqvknddyli
ldavnnqvyvnptnevidkmravqeqvasekaelaklkd
>3ECA_A
lpnitilatggTiagggdsatksnYtvgkvgvenlvnavpqlkdianvkgeqvvnigsqdmndnvwltlakki
ntdcdktdgfvithgTDtmeetayfldltvkcdkpvvmvgamrpstsmsadgpfnlynavvtaadkasanrgv
lvvmndtvldgrdvtKtnttdvatfksvnygplgyihngkidyqrtparkhtsdtpfdvsklnelpkvgivyn
yanasdlpakalvdagydgivsagvgngnlyksvfdtlataaktgtavvrssrvptgattqdaevddakygfv
asgtlnpqkarvllqlaltqtkdpqqiqqifnqy
>1Z48_B
arklftpitikdtlknrivspCysshekdgkltpfhahyisraigqvgliiveasavnpqgritdqdlgiwsd
ehiegfaklteqvkeqgskigiqlahagrkaelegdifapsaiafdeqsatpvesaekvketvqefkqaaara
keagfdvieiHaaHgYliheflsplsnhrtdeyggspenryrflreiidevkqvwdgplfvRvsasdytdkgl
diadhigfakwkeqgvdlidcssgalvhadinvfpgyqvsfaekireqadatgavgitdgsaeeilqngradl
ifigrellrdpffartaakqlnteipapvqyergw
>1FSG_A
gshmaskpiedygkgkgriepmyipdntfynaddflvpphckpyidkillpgglvkdrveklaydihrtyfge
elhiicilkgsrgffnllidylatiqkysgressvppffehyvrlksyqndnstgqltvlsddlsifrdkhvl
ivEDivDtgftltefgerlkavgpksmriatlveKrtdRsnslkgdfvgfsiedvwivgccydfnemfrdfdh
vavlsdaarkkfek
>1PUD_A
mveataqetdrprfsfsiaaregkartgtiemkrgvirtpafmpvgtaatvkalkpetvratgadiilgntyh
lmlrpgaeriaklgglhsfmgwdrpiltDsggyqvmslssltkqseegvtfkshldgsrhmlspersieiqhl
lgsdivmafdectpypatpsraassmersmrwakrsrdafdsrkeqaenaalfgiqqgsvfenlrqqsadala
eigfdgyavgglavgegqdemfrvldfsvpmlpddkphylmgvgkpddivgavergidmfdcvlptrsgrngq
aftwdgpinirnarfsedlkpldsechcavcqkwsrayihhlirageilgamlmtehniafyqqlmqkirdsi
segrfsqfaqdfraryfarns
>1DJ0_A
ppvykialgieydgskyygwqrqnevrsvqeklekalsqvanepitvfcagrtDagvhgtgqvvhfettalrk
daawtlgvnanlpgdiavrwvktvpddfharfsatarryryiiynhrlrpavlskgvthfyepldaermhraa
qcllgendftsfravqcqsrtpwrnvmhinvtrhgpyvvvdikanafvhhmvrnivgslmevgahnqpeswia
ellaakdrtlaaatakaeglylvavdypdrydlpkppmgplflad
>1AKO_A
mkfvsfNinglrarphqleaivekhqpdviglqetkvhddmfpleevaklgynvfyhgqkghygvalltketp
iavrrgfpgddeeaqrriimaeipsllgnvtvingyfpqgesrdhpikfpakaqfyqnlqnyletelkrdnpv
limgDmNisptdldigigeenrkrwlrtgkcsflpeerewmdrlmswglvdtfrhanpqtadrfswfdyrskg
fddnrglriDlllasqplaeccvetgidyeirsmekpsdHapvwatfrr
>1MOK_A
```

```
mkvwnarndhltinqwatrideileapdggeviynvdendpreydaifigggaagrfgsaylramggrqlivd
rwpfLggsCphnaCvphhlfsdcaaelmlartfsgqywfpdmtekvvgikevvdlfragrngpHgimnfqske
qlnleyilncpakvidnhtveaagkvfkaknlilavgagpgtldvpgvnakgvfdhatlveeldyepgstvvv
vggsktaveygcffnatgrrtvmlvrteplklikdnetrayvldrmkeqgmeiisgsnvtrieedangrvqav
vamtpngemrietdfvflglgeqprsaelakilgldlgpkgevlvneylqtsvpnvyavgdliggpmemfkar
ksgcyaarnvmgekisytpknypdflhthyevsflgmgeeearaagheivtikmppdtenglnvalpasdrtm
lyafgkgtahmsgfqkividaktrkvlgahhvgygakdafqylnvlikqgltvdelgdmdelFlnpthfiqls
rlragsknlvsl
>7ODC_A
mssftkdefdchildegftakdildqkinevsssddkdafyvadlgdilkkhlrwlkalprvtpfyavKcnds
raivstlaaigtgfdcaskteiqlvqglgvpaerviyanpckqvsqikyaasngvqmmtfdseielmkvarah
pkaklvlriatddskavcrlsvkfgatlktsrllllerakelnidvigvsfHvgsgctdpdtfvqavsdarcvf
dmatevgfsmhlldigggfpgsedtklkfeeitsvinpaldkyfpsdsgvriiaEpgryyvasaftlavniia
kktvwkeqpgsddedesneqtfmyyvndgvygsfncilydhahvkallqkrpkpdekyysssiwgptcdgldr
ivercnlpemhvgdwmlfenmgaytvaaastfngfqrpniyyvmsrpmwqlmkqiqshg
>1FWK_D
mkvrvkapctsanlgvgfdvfglclkepydvieveaiddkeiiievddkniptdpdknvagivakkmiddfni
gkgvkitikkgvkagsglgssaassagtayainelfklnldklklvdyasygelassgakhadnvapaifggf
tmvtnyeplevlhipidfkldiliaipnisinTkeareilpkavglkdlvnnvgkacgmvyalynkdkslfgr
ymmsdkviepvrgklipnyfkikeevkdkvygitisgsgpsiiafpkeefidevenilrdyyentirtevgkg
vevv
>1RBL_A
saagykagvkdykltyytpdytpkdtdllaafrfspqpgvpadeagaaiaaesstgtwttvwtdlltdmdryk
gkcyhiepvageensyfafiaypldlfeegsvtniltsivgnvfgfkairslrledirfpvalvktfqgpphg
iqverdllnkygrpmlgctiKpKlglsaknygravyeclrggldftKdDeninsqpfqrwrdrflfvadaihk
sqaetgeikghylnvtaptceemmkraefakelgmpiimhdfltagftanttlakwcrdngvllhiHramhav
idrqrnhgihfrvlakclrlsggdhlHsgtvvgklegdkastlgfvdlmredhieadrsrgvfftqdwasmpg
vlpvasggihvwhmpalveifgddsvlqfgggtlghpwgnapgatanrvaleacvqarnegrdlyreggdilr
eagkwspelaaaldlwkeikfefetmdkl
>2WTB_A
gktvmevggdgvavitlinppvnslsfdvlynlksnyeealsrndvkaivitgakgrfsggFdiepkagYisi
DiitdlleaarkpsvaaidglalgGglElamacharisapaaqlglpElqlgviPGfggtqrlprlvgltkal
emiltskpvkaeeghslglidavvppaelvttarrwaldivgrrkpwvssvsktdklpplgeareiltfakaq
tlkrapnmkhplmcldaievgivsgpraglekeaevasqvvkldttkglihvffsqrgtakvpgvtdrglvpr
kikkvaiigggdmgsgiatalilsnypvilkevnekfleagigrvkanlqsrvrsqekfektmsllkgsldye
sfrdvdmvieavienislkqqifadlekycpqhcilasntStidlnkigertksqdrivgahffspahimpll
EivrtnhtsaqvivdlldvgkkikktpvvvgnctgfavNrmffpytqaamflvecgadpylidraiskfgmpm
gpfrlcdlvgfgvaiatatqfienfsertyksmiiplmqedkdpelkkyiekarsisgvkldpklanlsekdi
iemtffpvvneacrvfaegiavkaadldiagimgmgfppyrggimfwadsigskyiysrldewskaygeffkp
caflaergskgvllsapvk
>2DOR_A
mlnttfanakfanpfmnasgvhcmtiedleelkasqagayitKsstlekregnplpryvdlelgsinsmglpn
lgfdyyldyvlknqkenaqegpiffsiagmsaaeniamlkkiqesdfsgitelnlsCpnvpgkpqlaydfeat
ekllkevftfftkplgvklppyfdlvhfdimaeilnqfpltyvnsvnsignglfidpeaesvvikpkdgfggi
ggayikptalanvrafytrlkpeiqiigtggietgqdafehllcgatmlqigtalhkegpaifdriikeleei
mnqkgyqsiadfhgklksl
>1AB8_A
slkneelyhqsydcvcvmfasipdfkefytesdvnkegleclrllneiiadfddllskpkfsgvekiktigst
ymaatglsaipsqehaqeperqymhigtmvefayalvgkldainkhsfndfklrvginhgpviagvigaqkpq
ydiwgntvnvasRmdstgvldkiqvteetslilqtlgytctcrgiinvkgkgdlktyfvntemsrslsqsnla
s
>1R4Z_A
```

aehnpvvmvhgIggasfnfagiksylvsqgwsrdklyavdfwdktgtnynngpvlsrfvqkvldetgakkvdi
vahSMggantlyyiknldggnkvanvvtlgganrlttgkalpgtdpnqkilytsiyssaDmivmnylsrldga
rnvqihgvgHigllyssqvnslikeglngggqntn
>1BS9_A
scpaihvfgareTtaspgygssstvvngvlsaypgstaeainypacggqsscggasysssvaqgiaavasavn
sfnsqcpstkivlvgySQggeimdvalcgggdpnqgytntavqlsssavnmvkaaifmgdpmfraglsyevgt
caaggfdqrpagfscpsaakiksycdasDpyccngsnaatHqgygseygsqalafvksklg
>1EMD_A
mkvavlgaaggigqalalllktqlpsgselslydiapvtpgvavdlshiptavkikgfsgedatpalegadvv
lisagvrrkpgmdrsdlfnvnagivknlvqqvaktcpkacigiitnpvnttvaiaaevlkkagvydknklfgv
ttlDiirsntfvaelkgkqpgevevpviggHsgvtilpllsqvpgvsfteqevadltkriqnagtevveakag
ggsatlsmgqaaarfglslvralqgeqgvvecayvegdgqyarffsqplllgkngveerksigtlsafeqnal
egmldtlkkdialgqefvnk
>1XQD_A
masgapsfpfsrasgpeppaefaklratnpvsqvklfdgslawlvtkhkdvcfvatseklskvrtrqgfpelg
aggkqaakakptfvdmdppehmhqrsmveptftpeavknlqpyiqrtvddlleqmkqkgcangpvdlvkefal
pvpsyiiytllgvpfndleyltqqnairtngsstarqasaanqelldylailveqrlvepkddiisklcteqv
kpgnidksdavqiaflllvagnatmvnmialgvatlaqhpdqlaqlkanpslapqfveelcryhtaSalaikr
takedvmigdklvranegiiasnqsanrdeevfenpdefnmnrkwppqdplgfgfgdhrciaehlakaelttv
fstlyqkfpdlkvavplgkinytplnrDvgivdlpvif
>1R16_B
ivptrelenvflgrckdyeitryldilprvrsdcsalwkdffkafsfknpcdldlgsykdfftsaqqqlpknk
vmfwsgvydeahdyantgrkyitlEdtlpgymlnslvwcgqranpgfnekvcpdfktcpvqaresfwgmasss
yahsaegevtymvdgsnpkvpayrpdsffgkyElpnltnkvtrvkvivlhrlgekiiekcgagslldleklvk
akhfafdcvenpravlfllcsdnpnarecrla
>2BX4_A
sgfrkmafpsgkvegcmvqvtcgtttlnglwlddtvycprHvictaedmlnpnyedllirksnhsflvqagnv
qlrvighsmqncllrlkvdtsnpktpkykfvriqpgqtfsvlacyngspsgvyqcamrpnhtikgsflngsCg
svgfnidydcvsfcymhhmelptgvhagtdlegkfygpfvdrqtaqaagtdttitlnvlawlyaavingdrwf
lnrftttlndfnlvamkynyepltqdhvdilgplsaqtgiavldmcaalkellqngmngrtilgstiledeft
pfdvvrqcsgvtfq
>1LTQ_A
mkkiiltigcpgsgKstwarefiaknpgfyninrDdyrqsimaheerdeykytkkkegivtgmqfdtaksily
ggdsvkgviisdtnlnperrlawetfakeygwkvehkvfdvpwtelvkrnskRgtkavpidvlrsmyksmrey
lglpvyngtpgkpkavifDvdgtlakmngrgpydlekcdtdvinpmvvelskmyalmgyqivvvsgresgtke
dptkyyrmtrkwvediagvplvmqcqreqgdtrkddvvkeeifwkhiaphfdvklaiddrtqvvemwrrigve
cwqvasgdf
>1I78_A
stetlsftpdninadislgtlsgktkervylaeeggrkvsqldwkfnnaaiikgainwdlmpqisigaagwtt
lgsrggnmvDqDwmdssnpgtwtdeArhpdtqlnyanefdlnikgwllnepnyrlglmagyqesrysftargg
syiysseegfrddigsfpngeraigykqrfkmpyigltgsyryedfelggtfkysgwvessdnDeHydpkgri
tyrskvkdqnyysvavnagyyvtpnakvyvegawnrvtnkkgntslydhnnntsdyskngagienynfittag
lkytf
>1E5Q_H
atksvlmlgsgfvtrptldvltdsgikvtvacrtlesakklsagvqhstpisldvnddaaldaevakhdlvis
lipytfhatviksairqkkhvvttsyvspammeldqaakdagitvmneiglDpgidhlyaiktieevhaaggk
iktflsycgglpapessdnplgykfswssrgvllalrnaasfykdgkvtnvagpelmatakpyfiypgfafva
ypnrdstpykeryqipeadnivrgtlryqgfpqfikvlvdigflsdeeqpflkeaipwkeatqkivkassase
qdivstivsnatfesteeqkrivaglkwlgifsdkkitprgnaldtlcatleekmqfeegerdlvmlqhkfei
enkdgsretrtsslceygapigsggysamaklvgvpcavavkfvldgtisdrgvlapmnskindplmkelkek
ygieckekvva
>1V25_A
megermnafpstmmdeelnlwdfleraaalfgrkevvsrlhtgevhrttyaevyqrarrlmgglralgvgvgd
rvatlgfnhfrhleayfavpgmgavlhtanprlspkeiayilnhaedkvllfdpnllplveairgelktvqhf

vvmdekapegylayeealgeeadpvrvperaacgmayttgttglpkgvvyshralvlhslaaslvdgtalsek
dvvlpvvpmfhvnawclpyaatlvgakqvlpgprldpaslvelfdgegvtftagvptvwlaladylestghrl
ktlrrlvvggsaaprsliarfermgvevrqgygltetspvvvqnfvkshleslseeekltlkaktglpiplvr
lrvadeegrpvpkdgkalgevqlkgpwitggyygneeatrsaltpdgffrtgdiavwdeegyveikdrlkdli
KsggeWissvdlenalmghpkvkeaavvaiphpkwqerplavvvprgekptpeelnehllkagfakwqlpday
vfaeeiprtsagkflkralreqyknyygga
>1AL6_A
asstnlkdvlaslipkeqariktfrqqhgntavgqitvdmsyggmrgmkgliyetsvldpdegirfrgfsipe
cqkllpkagggeeplpeglfwllvtgqiptpeqvswvskewakraalpshvvtmldnfptnlhpmsqlsaait
alnsesnfarayaeginrtkywefvyedamdliaklpcvaakiyrnlyragssigaidskldwshnftnmlgy
tdpqftelmrlyltihsdheggnvSahtshlvgsalsdpylsfaaamnglagplHglanqevllwlsqlqkdl
gadasdeklrdyiwntlnsgrvvpgygHavlrktdprytcqrefalkhlpsdpmfklvaqlykivpnvlleqg
kaknpwpnvDahsgvllqyygmtemnyytvlfgvsralgvlaqliwsralgfplerpksmstagleklsagg
>1MEK_A
dapeeedhvlvlrksnfaealaahkyllvefyapwCGHCkalapeyakaagklkaegseirlakvdateesdl
aqqygvrgyptikffrngdtaspkeytagreaddivnwlkkrtgpaa
>1DW9_A
miqsqinrnirldladaillskakkdlsfaeiadgtglaeafvtaallgqqalpadaarlvgakldldedsil
llqmiplrgciddriptdptmyRfyEmlqvygttlkalvhekfgdgiisainfkldvkkvadpeggeravitl
dgkylptkpf
>1HFE_M
srtvmerieyemhtpdpkadpdklhfvqideakcigcdtcsqycptaaifgemgephsiphieacincgqclt
hcpenaiyeaqswvpevekklkdgkvkciampapavryalgdafgmpvgsvttgkmlaalqklgfahcwdtef
tadvtiweegsefverltkksdmplpqftsCcpgwqkyaetyypellphfstckspigmngalaktygaermk
ydpkqvytvsimpciaKkyeglrpelkssgmrdidatlttrelaymikkagidfaklpdgkrdslmgestgga
tifgvtggvmeaalrfayeavtgkkpdswdfkavrgldgikeatvnvggtdvkvavvhgakrfkqvcddvkag
kspyhfieymacpggcvcgggqpvmpgvlea
>3FY4_C
sgsliwfrkglrvhdnpaleyaskgsefmypvfvidphymesdpsafspgssragvnrirflleslkdldssl
kklgsrllvfkgepgevlvrclqewkvkrlcfeydtdpyyqaldvkvkdyasstgvevfspvshtlfnpahii
ekngykpplsyqsflkvagepscakselvmsysslppigdignlgisevpsleelgykddeqadwtpfrgges
ealkrltksisdkawvanfekpkgdpsaflkpattvmspylkfgclssryfyqclqniykdvkkhtsppvsll
gQllWreffyttafgtpnfdkmkgnrickqipwnedhamlaawrdgktgypwidaimvqllkwgwmhHlarhc
vacfltrgdlfihweqgrdvferllidsdwainngnwmwlscssffyqfnriyspisfgkkydpdgkyirhfl
pvlkdmpkqyiyepwtaplsvqtkancivgkdypkpmvlhdsaskeckrkmgeayalnkkmdgkvdeenlrdl
rrklqkdeh
>3O32_B
ntrvielfdeftdlirdfivrheittpeyetimqymisvgeagewplwldaffettvdsvsygkgnwtssaiq
gpffkegaplltgkpatlpmradepgdrmrftgsvrdtsgtpitgavidvwhstndgnysffspalpdqyllr
grvvpaedgsiefhsirpvpYeipkagptgqlmnsylgrhswRpahihiritadgyrplitqlyfegdpylds
dscsavkselvlpvnkididgetwqlvdfnfilqhn
>1JXA_A
cgivgaiaqrdvaeilleglrrleyrgydsaglavvdaeghmtrlrrlgkvqmlaqaaeehplhggtgiahtr
wathgepsevnahphvsehivvvhngiienheplreelkargytfvsetdteviahlvnwelkqggtlreavl
raipqlrgaygtvimdsrhpdtllaarsgsplviglgmgenfiasdqlallpvtrrfifleegdiaeitrrsv
nifdktgaevkrqdiesnlqydagdkgiyrhymqkeiyeqpnaikntltgrishgqvdlselgpnadellskv
ehiqilacgtsynsgmvsrywfeslagipcdveiasefryrksavrrnslmitlsqsgetadtlaglrlskel
gylgslaicnvpgsslvresdlalmtnagteigvastkafttqltvllmlvaklsklkgldasiehdivhglq
alpsrieqmlsqdkriealaedfsdkhhalflgrgdqypialEgalKlkEisyihaeayaagelkHgplalid
admpvivvapnnelleklksnieevrarggqlyvfadqdagfvssdnmhiiemphveeviapifytvplqlla
yhvalikgtdvdqprnlaKsvtve
>1DGS_A
mtreearrrinelrdliryhnyryyvladpeisdaeydrllrelkeleerfpefkspdspteqvgarpleptf
rpvrhptrmysldnaftyeevlafeerlereaeapslytvehKvDglsvlyyeegvwstgsgdgevgeevtqn

lltiptiprrlkgvpdrlevrgevympieaflrlneeleergekvfknpRnaaagslrqkdprvtakrglrat
fyalglglgleesglksqyelllwlkekgfpvehcyekalgaegveevyrrglaqrhalpfeadgvvlklddl
tlwgelgytaraprfalayKfpaeeketrlldvvfqvgrtgrvtpvgvlepvfiegsevsrvtlhnesyieel
dirigdwvlvhkaggvipevlrvlkerrtgkerpirwpeacpecghrlvkegkvhrcpnplcpakrfeairhy
asrkamdieglgeklierllekglvrdvadlyhlrkedllglermgeksaqnllrqieeskhrglerllyalg
lpgvgevlarnlarrfgtmdrlleasleelieveevgeltarailetlkdpafrdlvrrlkeagvsmeskeev
sdllsgltfvltgelsrpreevkallgrlgakvtdsvsrktsylvvgenpgsklekaralgvavlteeefwrf
lkekgapvpa
>1SMN_A
dtlesidncavgcptggssnvsivrhaytlnnnsttkfanwvayhitkdtpasgktrnwktdpalnpadtlap
adytganaalkvdRgHqaplaslagvsdweslnylsnitpqksdlNqgawarledqerklidradissvytvt
gplyerdmgklpgtqkahtipsaywkvifinnspavnhyaaflfdqntpkgadfcqfrvtvdeiekrtgliiw
aglpddvqaslkskpgvlpelmgckn
>1IEC_A
avapvyvggflarydqspdeaelllprdvvehwlalplninHddtavvghvaamqsvrdglfclgcvtsprfl
eivrrasekselvsrgpvsplqpdkvveflsgsyagllSsrfkAvalcsvgRRrgtlavygrdpewvtqrfpd
ltaadrdglraqwqgdpfrsdsygllgnsvdalyirerlpklrydkqlvgvteresyvka
>3QKV_B
qkargaatrarqkqrasletmdkavqrfrlqnpdldsealltlpllqlvqklqsgelspeavfftylgkawev
nkgtncvtsyltdcetqlsqaprqgllygvpvslKecfsykghdstlglslnegmpsesdcvvvqvlklqgav
pfvhtnvpqsmlsfdcsnplfgqtmnpwkssskspggSSggegaligsggsplglgtdIGGSirfpsafcgicg
lkptgnrlsksglkgcvygqtavqlslgpmardveslalclkallcehlftldptvpplpfreevyrssrplr
vgyyetdnytmpspamrralietkqrleaaghtlipflpnnipyalevlsagglfsdggrsflqnfkgdfvdp
clgdlililrlpswfkrllsllklpflfprlaaflnsmrprsaeklwklqheiemyrqsviaqwkamnldvllt
pmlgpaldlntpgratgaisytvlyncldfpagvvpvttvtaeddaqmelykgyfgdiwdiilkkamknsvgl
pvavqcvalpwqeelclrfmreveqlmtpqkq
>1XNY_A
msepeeqqpdihttagkladlrrrieeathagsaravekqhakgkltareridlllldegsfveldefarhrst
nfgldanrpygdgvvtgygtvdgrpvavfsqdftvfggalgevygqkivkvmdfalktgcpvvgindsggari
qegvaslgaygeifrrnthasgvipqislvvgpcaGGavyspaitdftvmvdqtshmfitgpdviktvtgedv
gfeelggarthnstsgvahhmagdekdaveyvkqllsylpsnnlseppafpeeadlavtdedaeldtivpdsa
nqpydmhsviehvlddaeffetqplfapniltgfgrvegrpvgivanqpmqfagclditasekaarfvrtcda
fnvpvltfvdvpgflpgvdqehdgiirrgaklifayaeatvplitvitrkafgGAydvmgskhlgadlnlawp
taqiavmgaqgavnilhrrtiadagddaeatrarliqeyedallnpytaaergyvdavimpsdtrrhivrglr
qlrtkreslppkkhgnipl
>3FPC_A
mkgfamlsigkvgwiekekpapgpfdaivrplavapcTSdiHtvfegaigerhnmilgheavgevvevgsevk
dfkpgdrvvvpaitpdwrtsevqrgyhqhsggmlagwkfsnvkdgvfgeffhvndadmnlahlpkeipleaav
mipdmmttgfhgaelaniklgdtvcvigigpvglmsvaganhlgagrifavgsrkhccdialeygatdiinyk
ngdiveqilkatdgkgvdkvviaggdvhtfaqavkmikpgsdignvnylgegdnidiprsewgvgmghkhihg
glcpggrlrmerlidlvfykrvdpsklvthvfrgfdniekafmlmkdkpkdlikpvvila
>2J5M_A
epgsgigypydnntlpyvapgptdsrapCpalnalanhgyiphdgraisretlqnaflnhmgiansvielalt
nafvvceyvtgsdcgdslvnltllaephafeHDhsfsrkdykqgvansndfidnrnfdaetfqtsldvvagkt
hfdyadmneirlqreslsneldfpgwfteskpiqnvEsgfifalvsdfnlpdndenplvridwwkywftnesf
pyhlgwhppspareiefvtsassavlaasvtstpsslpsgaigpgaeavplsfastmtpfllatnapyyaqdp
tlgpnd
>3CYN_B
nlyfqsminsfyafevkdakgrtvslekykgkvslvvnvasdcqltdrnylglkelhkefgpshfsvlafpcn
qfgeSeprpskevesfarknygvtfpifhkikilgsegepafrflvdsskkeprWnfwkylvnpegqvvkfwr
peepievirpdiaalvrqviikkkedl
>1NF9_A

msgipeitayplptaqqlpanlarwsleprravllvhDmqryflrplpeslraglvanaarlrrwcveqgvqi
aytaqpgsmteeqrgllkdfwgpgmraspadrevveelapgpddwlltkwrysaffhsdllqrmraagrdqlv
lcgvyahvgvlistvdaysndiqpflvadaiadfseahhrmaleyaasrcamvvttdevle
>1PMI_A
ssekl friqcgyqnydwgkigsssavaqfvhnsdpsitidetkpyaelwmgthpsvpskaidlnnqtlrdlvt
akpqeylgesiitkfgsskelpflfkvlsiekvlsiQahpdkklgaqlhaadpknypddnhkpemaiavtdfe
gfcgfkpldqlaktlatvpelneiigqelvdefisgiklpaevgsqddvnnrkllqkvfgklmntdddvikqq
takllertdrepqvfkdidsrlpeliqrlnkqfpndiglfcgclllnhvglnkgeamflqakdphayisgdii
EcmaasdnvvRagftpkfkdvknlvemltysyesvekqkmplqefprskgdavksvlydppiaefsvlqtifd
kskggkqvieglngpsiviatngkgtiqitgddstkqkidtgyvffvapgssieltadsanqdqdfttyrafv
ea
>1F75_A
mfpikkrkaiknnninaaqipkhiaiimdgngRwakqkkmpRikghyegmqtvrkitryasdlgvkyltlyaf
stenwsrpkdevnylmklpgdflntflpelieknvkvetigfiddlpdhtkkavleakektkhntgltlvfal
nyggrkeiisavqliaeryksgeisldeisethfneylftanmpdpelliRtsgeeRlsnfliwqcsysefvf
idefwpdfneeslaqcisiyqnrhrrfggl
>1N29_A
alvnfhrmiklttgkeaalsygfygchcGvggrgspkdatdrccvtHdccykrlekrgcgtkflsykfsnsgs
ritcakqdscrsqlcecDkaaatcfarnkttynkkyqyysnkhcrgstprc
>1KRA_C
msnisrqayadmfgptvgdkvrladtelwieveddlttygeevkfgggkvirdgmgqgqmlaadcvdlvltna
livdhwgivkadigvkdgrifaigkagnpdiqpnvtipigaateviaaegkivtaggidthihwicpqqaeea
lvsgvttmvgggtgpaagthattctpgpwyisrmlqaadslpvnigllgkgnvsqpdalreqvaagviglkiH
eDwgatpaaidcaltvademdiqvalhsdtlnesgfvedtlaaiggrtihtfhtegaggghapdiitacahpn
ilpsstnptlpytlntidehldmlmvcHhldpdiaedvafaesRirretiaaedvlhdlgafsltssdsqamg
rvgevilrtwqvahrmkvqrgalaeetgdndnfrvkryiakytinpalthgiahevgsievgkladlvvwspa
ffgvkpatvikggmiaiapmgdinasiptpqpvhyrpmfgalgsarhhcrltflsqaaaangvaerlnlrsai
avvkgcrtvqkadmvhnslqpnitvdaqtyevrvdgelitsepadvlpmaqryflf
>3A5C_A
miqgviqkiagpaviakgmlgarmydickvgeeglvgeiirldgdtafvqvyedtsglkvgepvvstglplav
elgpgmlngiydgiqrplvhaldrekkwawtpmvkpgdevrggmvlgtvpefgfthkilvppdvrgrvkevkp
ageytveepvvvledgtelkmyhtwpvrrarpvqrkldpntpfltgmrildvlfpvamggtaaipgpfgsgkt
vtqqslakwsnadvvvyvgcgergnemtdvlvefpeltdpktggplmhrtvliantsnmpvaareasiyvgvt
iaeyfrdqgfsvalmadstsrwaealreissrleempaeegyppylaarlaafyeragkvitlggeegavtiv
gavsppggdmsepvtqstlrivgafwrldaslafrrhfpainwngsysLftsaldpwyrenvaedypelrdai
sellqreaglqeivqlvgpdalqdaerlvievgriiredflqqnayhevdaycsmkkaygimkmilafykeae
aaikrgvsideilqlpvlerigraryvseeefpayfeeamkeiqgafkal
>1EVY_A
mstkqhsakdellylnkavvfgsgafgtalamvlskkcrevcvwhmneeevrlvnekrenvlflkgvqlasni
tftsdvekayngaeiilfviptqflrgffeksggnliayakekqvpvlvctkgierstlkfpaeiigeflpsp
llsvlagpsfaievatgvftcvsiasadinvarrlqrimstgdrsfvcwattdtvgcevasavKnvlaigsgv
anglgmglnaraalimrglleirdltaalggdgsavfglaglgdlqlTcsselsrnftvgkklgkglpieeiq
rtskavaegvatadplmrlakqlkvkmplchqiyeivykkknprdaladllscglqdeglpplfkrsastpsk
l
>1FY2_A
mellllsnstlpgkawlehalplianqlngrrsavfipfagvtqtwdeytdktaevlaplgvnvtgihrvadp
laaiekaeiiivggGntfqllkesrergllapmadrvkrgalyigwSAganlacptirttndmpivdpngfda
ldlfplqinpHftnalpeghkgetreqrirellvvapeltviglpEgnwiqvsngqavlggpnttwvfkagee
avaleaghrf
>3N3I_A
pqvtlwqrplvtikiggqlkealldtgaddtvleemslpgrwkpkmivgiggfikvrqydqilieicghkaig
tvlvgptpvniigrnlltqigftlnfpqvtlwqrplvtikiggqlkeallDtGaddtvleemslpgrwkpkmi
vgiggfikvrqydqilieicghkaigtvlvgptpvniigrnlltqigftlnf
>1JDW_A

mlrvrclrggsrgaeavhyigsrlgrtltgwvqrtfqstqaatassrnscaaddkateplpkdcpvssynewd
pleevivgraenacvppftievkantyekywpfyqkqgghyfpkdhlkkavaeieemcnilktegvtvrrpdp
idwslkyktpdfestglysamprdilivvgneiieapmawrsrffeyrayrsiikdyfhrgakwttapkptma
delynqdypihsvedrhklaaqgkfvttefepcfDaadfiragrdifaqrsqvtnylgiewmrrhlapdyrvh
iisfkdpnpmHidatfniigpgivlsnpdrpchqidlfkkagwtiitpptpiipddhplwmsskwlsmnvlml
dekrvmvdanevpiqkmfeklgittikvnirnanslgggfhCwtcdvrrrgtlqsyld
>1F2D_A
agvakfakypltfgpspisnlnrlsqhlgskvnvyakredcnsglafggnKlrkleyivpdivegdythlvsi
ggrqSnqTrmvaalaaklgkkcvliqedwvpipeaekdvynrvgnielsrimgadvrviedgfdigmrksfan
alqeledaghkpypipagcsehkygglgfvgfadevinqevelgikfdkivvccvtgsttagilagmaqygrq
ddviaidasftsektkeqtlrianntakligvehefkdftldtrfaypcygvpnegtieairtcaeqegvltd
pvYEgksmqglialikedyfkpganvlyvhlggapalsayssffptkta
>1RK2_A
mqnagslvvlgsinadhilnlqsfptpgetvtgnhyqvafggkganqavaagrsganiafiactgddsigesv
rqqlatdniditpvsvikgestgvalifvngegenvigihaganaalspalveaqrerianasallmqlespl
esvmaaakiahqnktivalnpaparelpdellalvdiitpneteaekltgirvendedaakaaqvlhekgirt
vlitlgsrgvwasvngegqrvpgfrvqavdtiAAGDtfngalitalleekplpeairfahaaaaiavtrkgaq
psvpwreeidafldrqr
>1AHJ_B
mdgvhdlagvqgfgkvphtvdadigptfhaewehlpyslmfagvaelgafsvdevRyvvermeprhymmtpyy
eryvigvatlmvekgiltqdeleslaggpfplsrpsesegrpapvetttfevgqrvrvrdeyvpghirmpayc
rgrvgtishrttekwpfpdaighgrndageeptyhvkfaaeelfgsdtdggsvvvdlfegylepaa
>3TNE_B
dsislslinegpsyaskvsvgsnkqqqtviiDTgSsdfwvvdsnaqcgkgvdckssgtftpsssssyknlgaa
ftirygdgstsqgtwgkdtvtingvsitgqqiadvtqtsvdqgilgigytsneavydtsgrqttpnydnvpvt
lkkqgkirtnayslylnspsaetgtiifggvdnakysgklvaeqvtssqaltislasvnlkgssfsfgdgall
DsgTtltyfpsdfaaqladkagarlvqvardqylyfidcntdtsgttvfnfgngakitvpnteyvyqngdgtc
lwgiqpsddtilgdnflrhayllynldantisiaqvkyttdssisav
>3NOS_B
pkfprvknwevgsitydtlsaqaqqdgpctprrclgslvfprklqgrpspgppapeqllsqardfinqyyssi
krsgsqaheqrlqeveaevaatgtyqlreselvfgakqawrnaprCvgRiqwgklqvfdardcrsaqemftyi
cnhikyatnrgnlrsaitvfpqrcpgrgdfriwnsqlvryagyrqqdgsvrgdpanveitelciqhgwtpgng
rfdvlplllqapdeppelfllppelvlevplehptlewfaalglrwyalpavsnmlleigglefpaapfsgWy
mstEigtrnlcdphryniledvavcmdldtrttsslwkdkaaveinvavlhsyqlakvtivdhhaatasfmkh
leneqkarggcpadwawivppisgsltpvfhqemvnyflspafryqpdpwkgsaakgtgitr
>1LXT_B
vkivtvktkaypdqkpgtsglrkrvkvfqsstnyaenfiqsiistvepaqrqeatlvvggdgrfymkeaiqli
vriaaangigrlvigqngilstpavsciirkikaiggiiltaSHnpggpngdfgikfNisnggpapeaitdki
fqisktieeyaicpdlkvdlgvlgkqqfdlenkfkpftveivdsveayatmlrnifdfnalkellsgpnrlki
ridamhgvvgpyvkkilceelgapansavncvpledfgghhpdpnltyaadlvetmksgehdfgaafdgdgdR
nmilgkhgffvnpsdsvaviaanifsipyfqqtgvrgfarsmptsgaldrvanatkialyetptgwkffgnlm
dasklslcgeesfgtgsdHirekdglwavlawlsilatrkqsvedilkdhwhkfgrnfftrydyeeveaegat
kmmkdlealmfdrsfvgkqfsandkvytvekadnfeyhdpvdgsvsknqglrlifadgsriifrlsgtgsaga
tirlyidsyekdnakinqdpqvmlaplisialkvsqlqertgrtaptvit
>1T7D_B
mivrsfiyepfqipSgSmmptlligdfilvekfaygikdpiyqktlietghpkrgdivvfkypedpkldyiKr
avglpgdkvtydpvskeltiqpgcssgqacenalpvtysnvepsdfvqtfsrrnggeatsgffevpknetken
girlserketlgdvthriltvpiaqdqvgmyyqqpgqqlatwivppgqyfmmgdnrdnsadsrywgfvpeanl
vgrataiwmsfdkqegewptglrlsriggih
>1BOU_B
marvttgitsshipalgaaiqtgtsdndywgpvfkgyqpirdwikqpgnmpdvvilvyndhasafdmniiptf
aigcaetfkpadegwgprpvpdvkghpdlawhiaqslildefdmtimnqmdvdhgctvplsmifgepeewpck
vipfpvnvvtypppsgkrcfalgdsiraavesfpedlnvhvwgtggmsHqlqgpraglinkefdlnfidklis

dpeelskmphiqylresgsegvelvmwlimrgalpekvrdlytfyhipasntalgamilqpeetagtpleprk
vmsghslaqa
>1FUQ_A
mntvrsekdsmgaidvpadklwgaqtqrslehfristekmptslihalaltkraaakvnedlgllseekasai
rqaadevlagqhddefplaiwqtgsgtqsnmnmnevlanrasellggvrgmerkvhpnddvnksqssndvfpt
amhvaallalrkqlipqlktltqtlneksrafadivkigrTHlqdatpltlgqeisgwvamlehnlkhieysl
phvaelalggtavgtglnthpeyarrvadelavitcapfvtapnkfealatcdalvqahgalkglaaslmkia
ndvrwlasgprcgigeisipenepgssimpgKvnptqcEaltmlccqvmgndvainmggasgnfelnvfrpmv
ihnflqsvrlladgmesfnkhcavgiepnrerinqllneslmlvtalnthigydkaaeiakkahkegltlkaa
alalgylseaefdswvrpeqmvgsmkagrhhhhh
>1E7L_A
mlltgklykeekqkfydaqngkclicqrelnpdvqanhldHdHelngpkagkvrgllcnlcdaaEgqmkhkfn
rsglkgqgvdylewlenlltylksdytqnniHpnfvgdkskefsrlgkeemmaemlqrgfeynesdtktqlia
sfkkqlrkslk
>1XS1_A
mrlcdrdieawldegrlsinprppveringatvdvrlgnkfrtfrghtaafidlsgpkdevsaaldrvmsdei
vldegeafylhpgelalavtlesvtlpadlvgwldgrSslaRlglmvhvtAhRidpgwsgcivlEfynsgklp
lalrpgmligalsfeplsgpavrpynrredakyrnqqgavasridkd
>1DEK_A
mkliflsgvkrsgkdttadfimsnysavkyqlagpikdalayawgvfaantdypcltrkefegidydRetnln
ltklevitimeqafcylngkspikgvfvfddegkesvnfvafnkitdvinniedqwsvrrlmqalgtdlivnn
fdrmywvklfaldyldkfnsgydyyivpdtrqdhemdaaramgatvihvvrpgqksndtHiteaglpirdgdl
vitndgsleelfskikntlkvl
>2VEP_A
msklellpavDvrdgqavrlvhgesgtetsygspleaalawqrsgaewlhlvdldaafgtgdnraliaevaqa
mdikvelsggirdddtlaaalatgctrvnlgtaaletpewvakviaehgdkiavglDvrgttlrgrgwtrdgg
dlyetldrlnkegcaryvvtdiakdgtlqgpnlellknvcaatdrpvvasggvsslddlraiaglvpagvega
ivgkalyakaftleealeats
>1C17_M
hgkskliaplaltifvwvflmnlmdllpidllpyiaehvlglpalrvvpsadvnvtlsmalgvfililfysik
mkgiggftkeltlqpfnhwafipvnlilegvsllskpvSlglRlfgNmyagelifiliagllpwwsqwilnvp
waifhiliitlqafifmvltivylsmaseeh
>1C17_L
menlnmdllymaaavmmglaaigaaigigilggkflegaarqpdlipllrtqffivmglvDaipmiavglgly
vmfava
>1UK7_A
manleigksilaagvltnyhdvgegqpvilihgsgpgvsayanwrltipalskfyrviapdmvgfgftdrpen
ynyskdswvdhiigimdaleiekahivgnAfggglaiatalryservdrmvlmgaagtrfdvteglnavwgyt
psienmrnlldifaydrslvtdelarlryeasiqpgfqesfssmfpeprqrwidalassdediktlpnetlii
hgreDqvvplssslrlgelidraqlhvfgrcgHwtqieqtdrfnrlvveffneantpklvgrp
>1HIA_A
iiggreceknshpwqvaiyhyssfqcggvlvnpkwvltaaHckndnyevwlgrhnlfenentaqffgvtadfp
hpgfnls
>2DHN_A
mqdtiflkgmrfygyhgalsaEneigqifkvdvtlkvdlseagrtdnvidtvhygevfeevksimegkavnll
ehlaerianrinsqynrvmetkvritKenppipghydgvgieivrenk
>1D2H_A
vdsvyrtrslgvaaegipdqyadgeaarvwqlyigdtrsrtaeykawllgllrqhgchrvldvacgtgvdsim
lveegfsvtsvdasdkmlkyalkerwnrrkepafdkwvieeanwltldkdvpagdgfdaviclGnsfahlpds
kgdqsehrlalkniasmvrpggllvidhKnydyilstgcappgkniyyksdltkdittsvltvnnkahmvtld
ytvqvpgagrdgapgfskfrlsYyphclasftelvqeafggrcqhsvlgdfkpyrpgqayvpcyfihvlkktg
>1C4Z_A
npylrlkvrRdhiiddalvrlemiamenpadlkkqlyvefegEqgvdeggvskEffqlvveeifnpdigmfty
destklfwfnpssfetegqftligivlglaiynncilDvhfpmvvyrklmgkkgtfrdlgdshpvlyqslkdl

leyegnveddmmitfqisqtdlfgnpmmydlkengdkipitnenrkefvnlysdyilnksvekqfkafrrgfh
mvtnesplkylfrpeeiellicgsrnldfqaleetteydggytrdsvlirefweivhsftdeqkrlflqfttg
tdrapvgglgklkmiiakngpdterlptsHtCfnvlllpeysskeklkerllkaitya
>1OPM_A
neclgtigpvtpldasdfaldirmpgvtpkesdtyfcmsmrlpvdeeafvidfkprasmdtvhHmllfgcnmp
sstgsywfcdegtctdkanilyawarnapptrlpkgvgfrvggetgskyfvlQvhygdisafrdnhkdcsgvs
vhltrvpqpliagmylmmsvdtvippgekvvnadiscqykmypmhvfayrvHthhlgkvvsgyrvrngqwtli
grqnpqlpqafypvehpvdvtfgdilaarcvftgegrteathiggtssdemcnlyimyymeakyalsfmtctk
nvapdmfrtipaeanipi
>1FVA_B
ivspqealpgrkeplvvaakhhvngnrtvepfpegtqmavfgmgcfwgaerkfwtlkgvystqvgfaggytpn
ptykevcsgktghaEvvrvvfqpehisfeellkvfwenhdptqgmrqgndhgsqyrsaiyptsaehvgaalks
kedyqkvlsehgfglittdiregqtfyyaedyhqqylskdpdgycglggtgvscp
>1C3C_A
veryslspmkdlwteeakyrrwlevelavtrayeelgmipkgvterirnnakidvelfkkieektnhdvvafv
egigsmigedsrffhygltssdvldtanslalveagkilleslkefcdvlwevanrykhtptigrTHgvhaep
tsfglkvlgwysemkrnvqrleraieevsygkisgavgnyanvppeveekalsylglkpepvstqvvprdrha
fylstlaivaagieriaveirhlqrtevleveepfrkgqrgssamphKknpitcErltglsrmmrayvdpsle
nialwherdishssveryvfpdatqtlyymivtatnvvrnmkvneermkknidltkglvfsqrvllkliekgl
trkeaydivqrnalktwnsekhfleylledeevkklvtkeeleelfdisyylkhvdhiferfek
>1ELS_A
avskvyarsvydsrgnptveveltteкgvfrsivpsgastgvhealemrdgdkskwmgkgvlhavknvndvia
pafvkanidvsdqkavddflisldgtanksklganailgvslaasraaaaeknvplykhladlsksktspyvl
pvpflnvlnggshaggalalqEfmiaptgaktfaealrigsevyhnlksltkkrygasagnvgdEggvapniq
taeealdlivdaikaaghdgkvkigldcasseffkdgkydldfknpnsdkskwltgpqladlyhslmkrypiv
siedpfaeddweawshffktagiqivaddltvtnpkriataiekkaadalllkvnqigtlsesikaaqdsfaa
gwgvmvsHrsgetedtfiadlvvglrtgqiKtgaparserlaklnqllrieeelgdnavfagenfhhgdkl
>1CUD_B
rttrddlingnsascadvifiyargStetgnlgtlgpsiasnlesafgkdgvwiqgvggayratlgdnalprg
tssaairemlglfqqantkcpdatliaggySQgaalaaasiedldsairdkiagtvlfgytknlqnrgripny
padrtkvfcktgDlvctgslivaapHlaygpdaegpapefliekvravrgs
>1DFO_A
mlkremniadydaelwqameqekvrqeehieliasenytsprvmqaqgsqltnkyaEgypgkryyggceyvdi
veqlaidrakelfgadyanvqphsgsqanfavytallepgdtvlgmnlahgghlthgspvnfsgklynivpyg
idatghidyadlekqakehkpkmiiggfsaysgvvdwakmreiadsigaylfvdmahvaglvaagvypnpvph
ahvvttTthKtlagprgglilakggseelykklnsavfpggqggplmhviagkavalkeamepefktyqqqva
knakamvevflergykvvsggtdnhlflvdlvdknltgkeadaalgranitvnknsvpndpkspfvtsgirvg
tpaitrrgfkeaeakelagwmcdvldsindeavierikgkvldicarypvya
>1BOL_A
sscsstalscsnsansdtccspeyglvvlnmqwapgygpdnaftlHglwpdkcsgayapsggcdsnrasssia
svikskdsslynsmltywpsnqgnnnvfwshEwskHgtcvstydpdcydnyeegedivdyfqkamdlrsqynv
ykafssngitpggtytatemqsaiesyfgakakidcssgtlsdvalyfyvrgrdtyvitdalstgscsgdvey
ptk
>1XTC_A
nddklyRadsrppdeikqsgglmprgqseyfdrgtqmninlydhargtqtgfvrhddgyvStsislrsahlvg
qtilsghstyylyvlatapnmfnvndvlgaysphpdEqEvsalggipysqiygwyrvhfgvldeqlhrnrgyr
dryysnldiapaadgyglagfppehrawreepwihhappgcgnaprss
>1BOB_A
msandfkpetwtssanealrvsivgenavqfsplftypiygdsekiygykdliihlafdsvtfkpyvnvkysa
klgddnivdvekkllsflpkddvivrdeakwvdcfaeerkthnlsdvfekvseyslngeefvvyksslvddfa
rrmhrrvqifsllfieaanyidetdpswqiywllnkktkeligfvttykywhylgaksfdedidkkfrakisq
flifppyqnkghgsclyeaiiqswledksiteitvEdpneafddlrdrndiqrlrklgydavfqkhsdlsdef
lessrkslkleerqfnrlvemllllnns
>1P5D_X

```
mstakaptlpasifraydiRgvvgdtltaetaywigraigseslargepcvavgrdgrlsgpelvkqliqglv
dcgcqvsdvgmvptpvlyyaanvlegksgvmltgsHnppdyngfKivvagetlaneqiqalrerieknldlasg
vgsveqvdilpryfkqirddiamakpmkvvvdcgngvagviapqliealgcsviplycevdgnfpnhhpdpgk
penlkdliakvkaenadlglafdgdgdRvgvvtntgtiiypdrllmlfakdvvsrnpgadiifdvkctrrlia
lisgyggrpvmwktghslikkkmketgallagemsgHvffkerwfgfddgiysaarlleilsqdqrdsehvfs
afpsdistpeinitvtedskfaiiealqrdaqwgegnittldgvrvdypkgwglvrasnttpvlvlrfeadte
eeleriktvfrnqlkavdsslpvpf
>1LWD_A
adqrikvakpvvemdgdemtriiwqfikeklilphvdvqlkyfdlglpnrdqtndqvtidsalatqkysvavk
catitpdearveefklkkmwkspngtirnilggtvfrepiickniprlvpgwtkpitigrhahgdqykatdfv
vdragtfkiVftpkdgssakqwevynfpaggvgmgmyntdesisgfahscfqyaiqkkwplymstKntilkay
dgrfkdifqeifekhyktdfdkykiwyehrliDdmvaqvlkssggfvwacknydgdvqsdilaqgfgslglmt
svlvcpdgktieaeaahgtvtrhyrehqkgrptstnpiasifawtrglehrgkldgnqdlirfaqtlekvcve
tvesgamtkdlagcihglsnvklnehflntsdfldtiksnldralgrq
>1CI8_A
mtaasldptafsldaaslaarldavfdqalrerrlvgavaivarhgeilyrraqgladreagrpmredtlfrl
aSvtKpivalavlrlvargelaldapvtrwlpefrprladgseplvtihhllthtsglgywllegagsvydrl
gisdgidlrdfdldenlrrlasaplsfapgsgwqYslaldvlgavveratgqplaaavdalvaqplgmrdcgf
vsaeperfavpyhdgqpepvrmrdgievplpeghgaavrfapsrvfepgaypsggagmygsaddvlraleair
anpgflpetladaarrdqagvgaetrgpgwgfgylsavlddpaaagtpqhagtlqWggVyghswfvdralgls
vllltntayegmsgpltialrdavyar
>1BMT_A
qaewrswevnkrleyslvkgitefieqdteearqqatrpieviegplmdgmnvvgdlfgegkmflpqvvksar
vmkqavaylepfieaskeqgktngkmviatvkgDvHdigknivgvvLqcnnyeivdlgvmvpaekilrtakev
nadliglsglitpSldemvnvakemerqgftiplliggattskahtavkieqnysgptvyvqnasrtvgvvaa
llsdtqrddfvartrkeyetvriqhgr
>1LCI_A
medaknikkgpapfypledgtageqlhkamkryalvpgtiaftdahievnityaeyfemsvrlaeamkrygln
tnhrivvcsenslqffmpvlgalfigvavapandiynerellnsmnisqptvvfvskkglqkilnvqkklpii
qkiiimdsktdyqgfqsmytfvtshlppgfneydfvpesfdrdktialimnssgstglpkgvalphrtacvRf
shardpifgnqiipdtailsvvpfhHgfgmfttlgylicgfrvvlmyrfeeelflrslqdykiqsallvptlf
sffakstlidkydlsnlheiasggaplskevgeavakrfhlpgirqgyglTettsailitpegddkpgavgkv
vpffeakvvdldtgktlgvnqrgelcvrgpmimsgyvnnpeatnalidkdgwlhsgdiaywdedehffivdrl
kslikykgyqvapaelesillqhpnifdagvaglpdddagelpaavvvlehgktmtekeivdyvasqvttakk
lrggvvfvdevpkgltgKldarkireilikakkggkskl
>2Z5U_A
sgvegaafqsrlphdrmtsqeaacfpdiisgpqqtqkvflfirnrtlqlwldnpkiqltfeatlqqleapyns
dtvlvhrvhsylerhglinfgiykrikplptkktgkviiigsgvsglaaarqlqsfgmdvtlleardrvggrv
atfrkgnyvadlgamvvTglggnpmavvskqvnmelakikqkcplyeangqavpkekdemveqefnrlleats
ylshqldfnvlnnkpvslgqalevviqlqekhvkdeqiehwkkivktqeelkellnkmvnlkekikelhqqyk
eitaeflvkskhrdltalckeydelaetqgkleeklqeleanppsdvylssrdrqildwhfanlefanatpls
tlslkHwdqdddfeftgshltvrngyscvpvalaegldiklntavrqvrytasgceviavntrstsqtfiykc
davlctlplgvlkqqppavqfvpplpewktsavqrmgfgnlnKvvlcfdrvfwdpsvnlfghvgsttasrgel
flfwnlykapillalvageaagimenisddvivgrclailkgifgssavpqpketvvsrwradpwargsysyv
aagssgndydlmaqpitppiprlffagehtirnypatvhgallsglreagriadqflg
>3EBH_A
pkihyrkdykpsgfiinqvtlninihdqetivrsvldmdiskhnvgedlvfdgvglkineisinnkklvegee
ytydnefltifskfvpkskfafsseviihpetnyaltglykskniivsqceatgfrritffidrpdmmakydv
tvtadkekypvllsngdkvnefeipggrhgarfndpplkpcylfavvagdlkhlsatyitkytkkkvelyvfs
eekyvsklqwaleclkksmafdedyfgleydlsrlnlvavsdfnvgamEnkglnifnansllaskknsidfsy
ariltvvgheyfhqytgnrvtlrdwfqltlkegltvhrenlfseemtktvttrlshvdllrsvqfledsspls
hpirpesyvsmEnfytttvydkgsevmrmyltilgeeyykkgfdiyikkndgntatcedfnyameqaykmkka
dnsanlnqyllwfsqsgtphvsfkynydaekkqysihvnqytkpdenqkekkplfipisvglinpengkemis
qttleltkesdtfvfnniavkpipslfrgfsapvyiedqltdeerilllkydsdafvrynsctniymkqilmn
```

yneflkakneklesfqltpvnaqfidaikylledphadagfksyivslpqdryiinfvsnldtdvladtkeyi
ykqigdklndvyykmfksleakaddltyfndeshvdfdqmnmrtlrntllsllskaqypnilneiiehskspy
psnwltslsvsayfdkyfelydktyklskddelllqewlktvsrsdrkdiyeilkklenevlkdsknpndira
vylpftnnlrrfhdisgkgykliaevitktdkfnpmvatqlcepfklwnkldtkrqelmlnemntmlqepqis
nnlkeyllrltnk
>1M21_B
fpyaetdvadlqarmtageldsttltqaylqriaaldrtgprlravielnpdalkeaaerdrerrdgrlrgpl
hgiplllKdninaapmatsagslalqgfrpddaylvrrlrdagavvlgktnlsewanfrgndsisgwsarggq
trnpyrishspcgSssgsavavaanlasvaigtetdgSivcpaaingvvglkptvglvsrdgiipisfsqdta
gpmarsvadaaavltaiagrddadpatatmpgravydytarldpqglrgkrigllqtpllkyrgmpplieqaa
telrragavvvpvelpnqgawaeaertlllyefkagleryfnthraplrsladliafnqahskqelglfgqel
lveadatagladpayirarsdarrlagpegidaalaahqldalvapttgvawpirsegddfpgesysaaavag
ypsltvpmgqidglpvgllfmgtawsepkliemayayeqrtrarrpphf
>1AT1_A
anplyqkhiisindlsrddlnlvlataaklkanpqpellkhkviascffeastRTrlsfqtsmhrlgasvvgf
sdsantslgkkgetladtisvistyvdaivmRhpqegaarlatefsgnvpvlnagdgsnqHptqtlldlftiq
qtegrldnlhvamvgdlkygrtvhsltqalakfdgnrfyfiapdalampeyildmldekgiawslhssieevm
aevdilymtrvqkerldpseyanvkaqfvlrasdlhnakanmkvlhplprvdeiatdvdktphawyfqqagng
ifarqallalvlnrdlvl
>1J53_A
mstaitrqivldtEttgmnqigahyeghkiieigavevvnrrltgnnfhvylkpdrlvdpEafgvhgiadefl
ldkptfaevadefmdyirgaelvihnaafdigfmdyefsllkrdipktntfckvtdslavarkmfpgkrnsld
alcaryeidnskrtlHgalldaqilaevylamtggqtsma
>1QLH_A
stagkvikckaavlweekkpfsieevevappkahevrikmvatgicrSddHvvsgtlvtplpviagheaagiv
esigegvttvrpgdkviplftpqcgkcrvckhpegnfclkndlsmprgtmqdgtsrftcrgkpihhflgtstf
sqytvvdeisvakidaasplekvcligcgfstgygsavkvakvtqgstcavfglggvglsvimgckaagaari
igvdinkdkfakakevgatecvnpqdykkpiqevltemsnggvdfsfevigrldtmvtalsccqeaygvsviv
avtpdsqnlsmnpmlllsgrtwkgaifggfkskdsvpklvadfmakkfaldplithvlpfekinegfdllrsg
esirtiltf
>1QCN_A
sfipvaedsdfpiqnlpygvfstqsnpkprigvaigdqildlsvikhlftgpalskhqhvfdettlnnfglgq
aawkearaslqnllsasqarlrddkelrqraftsqasathlpatigdytdfyssrqHatnvgifrgkenallp
nwlhlpvgyhgrassivvsgtpirrpgqrpdnskppvygacrlldEleaffvgpgnrfgepipiskahehifg
vlndwsaRdiQqweyvplgpflgKsfgttispwvvpdalpfvvpnpkqdpkplpylchsqpytfdinlsvslk
gegsqaaticrsnfkhywtlqqlthhsvngcnlrpgdllasgtisgsdpesfgslElswkgtkaidvgqgqtr
tflldgdeviitghcqgdgyrvgfgqcagkvlpal
>1P4R_B
qlalfsvsdktglvefarnltalglnlvasggtakalrdaglavrdvseltgfpemlggrvKtlhpavhagil
arnipednadmarldfnlirvvacnlYpfvktvaspgvtveeaveqidIGgvtllraaaKnharvtvvceped
yvvvstemqsseskdtsletrrqlalkafthtaqydeaisdyfrkqyskgvsqmplrygmnphqtpaqlytlq
pklpitvlngapgfinlcdalnawqlvkelkealgipaaasfKHvspagaavgiplsedeakvcmvydlyktl
tpisaayarargadrmssfgdfvalsdvcdvptakiisrevsdgiiapgyeeealtilskkkngnycvlqmdq
sykpdenevrtlfglhlsqkrnngvvdkslfsnvvtknkdlpesalrdlivatiavkytqsNsvcyakngqvi
gigagqqsrihctrlagdkanywwlrhhpqvlsmkfktgvkraeisnaidqyvtgtigededlikwkalfeev
pellteaekkewvekltevsissdaffpfrdnvdrakrsgvayiaapsgsaadkvvieacdelgiilahtnlr
lfhH
>1B5T_A
gqinvsfEffpprtsemeqtlwnsidrlsslkpkfvsvtygansgerdrthsiikgikdrtgleaaphltcid
atpdelrtiardywnngirhivalrgDlppgsgkpemyasdlvtllkevadfdisvaaypevhpeaksaqadl
lnlkrkvdaganraitqfffdvesylrfrdrcvsagidveiipgilpvsnfkqakkfadmtnvripawmaqmf
dglddd aetrklvganiamdmvkilsregvkdfhfytlnraemsyaichtlgvrpa
>1GPR_A

```
miaeplqneigeevfvspitgeihpitdvpdqvfsgkmmgdgfailpsegivvspvrgkilnvfpTkHaiglq
sdggreiliHfGidtvslkgegftsfvsegdrvepgqkllevdldavkpnvpslmtpivftnlaegetvsika
sgsvnreqedivkiek
>1D2T_A
lalvatgndtttkpdlyylknseainslallppppavgsiaflndqamyeqgrllrntergklaaedanlssg
gvanafsgafgspitekdapalhklltnmiedagdlatrsakdhymrirpfafygvstcntteqdklskngsy
psgHtsigwatalvlaeinpqrqneilkrgyelgqsRvicgyHwqsDvdaarvvgsavvatlhtnpafqqqlq
kakaefaqhqkk
>1H7X_A
mapvlskdvadiesilalnprtqshaalhstlakkldkkhwkrnpdkncfhceklennfddikhttlgergal
reamrclkcadapcqkscpthldiksfitsisnknyygaakmifsdnplgltcgmvcptsdlcvggcnlyate
egsinigglqqfasevfkamnipqirnpclpsqekmpeaysakiallgagpasiscasflarlgysditifek
qeyvgglstseipqfrlpydvvnfeielmkdlgvkiicgkslseneitlntlkeegykaafigiglpepktdd
ifqgltqdqgfytskdflplvaksskagmcachsplpsirgavivlgagdtafdcatsalrcgarrvflvfrk
gfvniravpeevelakeekceflpflsprkvivkggrivavqfvrteqdetgkwnededqivhlkadvvisaf
gsvlrdpkvkealspikfnrwdlpevdpetmqtsepwvfaggdivgmanttvesvndgkqaswyihkyiqaqy
gasvsakpelplfytpvdlvdisvemaglkfinpfglasaapttsssmirrafeagwgfaltktfsldkdivt
nvsprivrgttsgpmygpgqssflnielisektaaywcqsvtelkadfpdniviasimcsynkndwmelsrka
easgadalelnlsAphgmergmglacgqdpelvrnicrwvrqavqipffakltpnvtdivsiaraakeggad
gvtatntvsglmglkadgtpwpavgagkrttyggvsgtairpialravttiaralpgfpilatggidsaesgl
qflhsgasvlqvcsavqnqdftviqdyctglkallylksieelqgwdgqspgteshqkgkpvpriaelmgkkl
pnfgpyleqrkkiiaeekmrlkeqnaafpplerkpfipkkpipaikdvigkalqylgtfgelsnieqvvavid
eemcincgkcymtcndsgyqaiqfdpethlptvtdtctgctlclsvcpiidcirmvsrttpyepkrglplavn
pvc
>1CQJ_B
mnlheyqakqlfaryglpapvgyacttpreaeeaaskigagpwvvkcqvhaggrgkaggvkvvnskedirafa
enwlgkrlvtyqtdangqpvnqilveaatdiakelYlgavvdrssrrvvfmasteggveiekvaeetphlihk
valdpltgpmpyqgrelafklglegklvqqftkifmglatiflerdlaliEinplvitkqgdlicldgklgad
gnalfrqpdlremrdqsqedpreaqaaqwelnyvaldgnigcmvngaglamgtmdivklhggepanfldvggg
atkervteafkiilsddkvkavlvnifggivrcdliadgiigavaevgvnvpvvvrlegnnaelgakkladsg
lniiaakgltdaaqqvvaav
>1FUI_A
mkkislpkigirpvidgrrmgvresleeqtmnmakataallteklrhacgaavecvisdtciagmaeaaacee
kfssqnvgltitvtpcwcygsetidmdptrpkaiwgfngterpgavylaaalaahsqkgipafsiyghdvqda
ddtsipadveekllrfaraglavasmkgksylslggvsmgiagsivdhnffeswlgmkvqavdmtelrrridq
kiydeaelemalawadknfrygedennkqyqrnaeqsravlresllmamcirdmmqgnskladigrveeslgy
naiaagfqgqrhwtdqypngdtaeailnssfdwngvrepfvvatEndslngvamlmghqltgtaqvfaDvrty
wspeaiervtghkldglaehgiihlinsgsaaldgsckqrdsegnptmkphweisqqeadaclaatewcpaih
eyfrgggyssrflteggvpftmtrvniikglgpvlqiaegwsvelpkdvhdilnkrtnstwpttwfaprltgk
gpftdvysvmanwganhgvltighvgadfitlasmlripvcmhnveetkvyrpsawaahgmdiegqdyracqn
ygplykr
>1ESC_A
apadpvptvffgdSytanfgiapvtnqdsergwcfqakenypavatrsladkgitldvqadvscggalihhfw
ekqelpfgagelppqqdalkqdtqltvgslggntlgfnrilkqcsdelrkpsllpgdpvdgdepaakcgeffg
tgdgkqwlddqfervgaeleelldrigyfapdakrvlvgyprlvpedttkcltaapgqtqlpfadipqdalpv
ldqiqkrlndamkkaaadggadfvdlyagtgantacdgadrgigglledsqlellgtkipWyaHpndkgrdiq
akqvadkieeilnr
>1LWS_A
cfakgtnvlmadgsiecienievgnkvmgkdgrpreviklprgretmysvvqksqhrahksdssrevpellkf
tcnathelvvrtprsvrrlsrtikgveyfevitfemgqkkapdgrivelvkevsksypisegperanelvesy
rkasnkayfewtieardlsllgshvrkatyqtyapilyendhffdymqkskfhltiegpkvlayllglwigdg
lsdratfsvdsrdtslmervteyaeklnlcaeykdrkepqvaktvnlyskvvrgngirnnlntenplwdaivg
lgflkdgvKnipsflstdnigtretflaglidsdgyvtdehgikatiktihtsvrdglvslarslglvvsvna
```

epakvdmngtkhkisyaiymsggdvllnvlskcagskKfrpapaaafarecrgfyfelqelkeddyygitlsd
dsdhqfllanqvvvhn
>1D0S_A
mqtlhallrdipapdaeamartqqhidgllkppgslgrletlavqlagmpglngtpqvgekavlvmcadhgvw
degvavspkivtaiqaanmtrgttgvcvlaaqagakvhvidvgidaepipgvvnmrvargcgniavgpamsrl
qaealllevsryacdlaqrgvtlfgvgelgmanttpaaamvsvftgsdakevvgiganlppsridnkvdvvrr
aiainqpnprdgidvlskvggfdlvgmtgvmlgaarcglpvlldgflsysaalaacqiapavrpylipshfsa
ekgarialahlsmepylhmamrlgEgsgaalampiveaacamfhnmgelaasnivlpegnanat
>1A05_A
mkkiaifagdgigpeivaaarqvldavdqaahlglrcteglvggaaldasddplpaaslqlamaadavilgav
ggprwdayppakrpeqgllrlrkgldlyanlrpaqifpqlldasplrpelvrdvdilvvreltgdiYfgqprg
levidgkrrgfntmvydedeirriahvafraaqgrrkqlcsvdKanvlettrlwrevvtevardypdvrlshm
yvDnaamqlirapaqfdvlltgnmfgdilsdeasqltgsigmlpsaslgegramyepihgsapdiagqdkanp
latilsvammlrhslnaepwaqrveaavqrvldqglrtadiaapgtpvigtkamgaavvnalnlkd
>1S20_A
mkvtfeqlkaafnrvlisrgvdsetadacaemfarttesgvysHgvnrfprfiqqlengdiipdaqpkritsl
gaieqwdaqrsignltakkmmdraielaadhgiglvalrnanhwmrggsygwqaaekgyigicwtnsiavmpp
wgakecrigtnplivaipstpitmvdmsmsmfsygmlevnrlagrqlpvdggfddegnltkepgvieknrril
pmgywkgsgmsivldmiatllsdgasvaevtqdnsdeygisqifiaievdklidgptrdaklqrimdyvtsae
radenqairlpghefttllaenrrngitvddsvwakiqallehhhhhh
>1EYI_A
tdqaafdtnivtltrfvmeegrkargtgemtqllnslctavkaistavrkagiahlygiagstnvtgDqvkkl
DvlsndlvinvlkssfatcvlvseEdknaiivepekrgkyvvcfdpldgssnidclvsigtifgiyrknstde
psekdalqpgrnlvaagyalygsatmlvlamvngvncfmldpaigefilvdrdvkikkkgsiysinegyakef
dpaiteyiqrkkfppdnsapygaryvgsmvadvhrtlvyggifmypankkspkgklrllyecnpmayvmekag
glattgkeavldivptdihqrapiilgspedvtelleiyqkhaak
>1G8F_A
mpaphggilqdliardalkknellseaqssdilvwnltprqlcdielilnggfspltgflnendyssvvtdsr
ladgtlwtipitldvdeafanqikpdtrialfqddeipiailtvqdvykpnktieaervfrgdpehpaisylf
nvagdyyvggsleaiqlpqhydypglrktpaqlrlefqsrqwdrvvafqtrnpmhrahreltvraareanakv
lihpvvgltkpgdidhhtrvrvyqeiikrypngiaflsllplamrmsgdreavwhaiirknygashfivgRdh
agpgknskgvdfygpydaqelvesykheldievvpfrmvtylpdedryapidqidttktrtlnisgtelrrrl
rvggeipewfsypevvkilresnpprpkqgfsivlgnsltvsreqlsiallstflqfgggryykifehnnkte
llsliqdfigsgsgliipdqweddkdsvvgkqnvylldtsssadiqlesadepishivqkvvlfledngffvf
>1IM5_A
mpeealivvDmqrdfmpggalpvpegdkiipkvneyirkfkekgalivatrdwhpenhisfrerggpwprhcv
qntpgaefvvdlpedaviisKatepdkeaysgfegtdlakilrgngvkrvyicgvAteyCvrataldalkhgf
evyllrdavkgikpedeeraleemksrgikivqf
>1NW5_A
gttrhvydvcdcldtlaklpddsvqliicDPpynimladwddhmdyigwakrwlaeaervlsptgsiaifggl
qyqgeagsgdlisiishmrqnskmllanliiwnypngmsaqrffanrheeiawfaktkkyffdldavrepyde
etkaaymkdkrlnpesvekgrnptnvwrmsrlngnslervghptqkpaavierlvralshpgstvldffagsg
vtarvaiqegrnsictdaapvfkeyyqkqltflrsyeivegaanfgaalqr
>1K55_D
sitentswnkefsaeavngvfvlckssskscatndlaraskeylpaStfKipnaiigletgviknehqvfkwd
gkpramkqwerdltlrgaiqvSavpvFqqiarevgevrmqkylkkfsygnqnisggidkfWlegqlrisavnq
vefleslylnklsaskenqlivkealvteaapeylvhsktgFsgvgtesnpgvawwvgwveketevyffafnm
didnesklplrksiptkimesegiigg
>1QAZ_A
gshpfdqavvkdptasyvdvkarrtflqsgqlddrlkaalpkeydctteatpnpqqgemviprrylsgnhgpv
npdyepvvtlyrdfekisatlgnlyvatgkpvyatcllnmldkwakadallnydpksqswyqvewsaataafa
lstmmaepnvdtaqrervvkwlnrvarhqtsfpggdtsccnNhsywrgqeatiigviskddelfrwglgryvq
amglinedgsfvhemtRheqslhYqnyamlpltmiaetasrqgidlyaykengrdihsarkfvfaavknpdli
kkyasepqdtrafkpgrgdlnwieyqrarfgfadelgfmtvpifdprtggsatllaykp

>1PKN_A
skshseagsafiqtqqlhaamadtflehmcrldidsapitarntgiictigpasrsvetlkemiksgmnvaRm
nfshgtheyhaetiknvrtatesfasdpilyrpvavaldtkgpeiRtglikgsgtaevelkkgatlkitldna
ymaacdenilwldyknickvvevgskvyvddglislqvkqkgpdflvtevenggflgskkgvnlpgaavdlpa
vsekdiqdlkfgvdedvdmvfasfirkaadvhevrkilgekgknikiisKienhegvrrfdeileasdgimva
rgdlgieipaekvflaqkmiigrcnragkpvicaTqmlesmikkprptraegsdvanavldgadcimlSgEta
kgdypleavrmqhliareaeaamfhrklfeelarssshstdlmeamamgsveasykclaaalivltesgrsah
qvaryrprapiiavtrnhqtarqahlyrgifpvvckdpvqeawaedvdlrvnlamnvgkaagffkkgdvvivl
tgwrpgsgftntmrvvpvp
>1PJH_A
msqeirqnekisyriegpffiihlinpdnlnalegedyiylgelleladrnrdvyftiiqssgrffssgAdfk
giakaqgddtnkypsetskwvsnfvarnvyvtdafikhskvlicclngpaigLsaalvalcdivysindkvyl
lypfanlglitEggttvslplkfgtnttyeclmfnkpfkydimcengfisknfnmpssnaeafnakvleelre
kvkglylpsclgmkkllksnhidafnkansvevneslkywvdgeplkrfrqlgskqrkhrl
>1AK0_A
wgalghatvayvaqhyvspeaaswaqgilgsssssylasiaswadeyRltsagkwsaslhfidaednpptncn
vdyerdcgssgcsisaianytqrvsdsslssenhaealrflvhfigdmtqplhdeayavggnkinvtfdgyhd
nlhsdwdtympqkligghalsdaeswaktlvqniesgnytaqaigwikgdnisepittatrwasdanalvctv
vmphgaaalqtgdlyptyydsvidtielqiakggyrlanwineihgseiak
>1O9I_A
mfkhtrklqynakpdrsdpimarrlqeslggqwgettgmmsflsqgwastgaekykdllldtgteemahvemi
stmigylledapfgpedlkrdpslattmagmdpehslvhglnaslnnpngaawnagyvtssgnlvadmrfnvv
researlqvsrlysmtedegvrdmlkfllarEtqhqlqfmkaqeeleekygiivpgdmkeiehsefshvlmnf
sdgdgskafegqvakdgekftyqenpeamggiphikpgdprlhnhqg
>1G6S_A
mesltlqpiarvdgtinlpgsKsvsnralllaalahgktvltnlldsddvrhmlnaltalgvsytlsadrtrc
eiignggplhaegalelflgnagtamrplaaalclgsndivltgeprmkerpighlvdalrlggakityleqe
nypplrlqggftggnvdvdgsvssqfltallmtaplapedtvirikgdlvskpyiditlnlmktfgveienqh
yqqfvvkggqsyqspgtylvegdassasyflaaaaikggtvkvtgigrnsmqgdirfadvlekmgaticwgdd
yisctrgelnaidmdmnhipDaamtiataalfakgtttlrniynwrvkEtdrlfamatelrkvgaeveeghdy
iritppeklnfaeiatyndHrmamcfslvalsdtpvtildpkctaKtfpdyfeqlarisqaa
>3MO2_C
verivsrdiargyeripipcvnavdsepcpsnykyvsqncvtspmnidrnithlqycvciddcsssncmcgql
smrcwydkdgrllpefnmaepplifecnhacscwrncrnrvvqnglrarlqlyrtrdmgwgvrslqdippgtf
vceYvgelisdseadvreedsylfdldgevycidarfygnvsrfinhhcepnlvpvrvfmahqdlrfpriaff
strlieageqlgfdYgerfwdikgklfscrcgspkcrhs
>3L5Q_1
mdiilgirvqdsvilasskavtrgisvlkdsddKtrqlsphtlmsfaGeagdtvqfaeyiqaniqlysiredy
elspqavssfvrqelaksirsrrpyqvnvliggydkkknkpelyqidylgtkvelpygahgYsgfytfslldh
hyrpdmtteegldllklcvqelekrmpmdfkgvivkivdkdgirqvddfqaq
>3AR6_A
meaahsksteeclayfgvsettgltpdqvkrhlekyghnelpaeegkslwelvieqfedllvrilllaacisf
vlawfeegeetitafvepfvillilianaivgvwqernaenaiealkeyepemgkvyradrksvqrikardiv
pgdivevavgdkvpadirilsiksttlrvdqsiltgesvsvikhtepvpdpravnqdkknmlfsgtniaagka
lgivattgvsteigkirdqmaateqdktplqqkldefgeqlskvislicvavwlinighfndpvhggswirga
iyyfkiavalavaaipeglpavittclalgtrrmakknaivrslpsvetlgctsvicsDktgtlttnqmsvck
mfiidkvdgdfcslnefsitgstyapegevlkndkpirsgqfdglvelaticalcndssldfnetkgvyekvg
eatetalttlvekmnvfntevrnlskveranacnsvirqlmkkeftlefsrdrksmsvycspakssraavgnk
mfvkgapegvidrcnyvrvgttrvpmtgpvkekilsvikewgtgrdtlrclalatrdtppkreemvlddssrf
meyetdltfvgvvgmldpprkevmgsiqlcrdagirvimitgdnkgtaiaicrrigifgeneevadraytgre
fddlplaeqreacrraccfarvepshkskiveylqsydeitamtgdgvndapalkkaeigiamgsgtavakta
semvladdnfstivaaveegraiynnmkqfirylissnvgevvcifltaalglpealipvqllwvnlvtdglp
atalgfnppdldimdrpprspkeplisgwlffrymaiggyvgaatvgaaawwfmyaedgpgvtyhqlthfmqc

```
tedhphfegldceifeapepmtmalsvlvtiemcnalnslsenqslmrmppwvniwllgsiclsmslhflily
vdplpmifklkaldltqwlmvlkislpvigldeilkfiarnyleg
>1QMH_A
mvkrmialdgaqgegggqilrsalslsmitgqpftitsiragrakpgllrqhltavkaateicgatvegaelg
sqrllfrpgtvrggdyrfaigsagsctlvlqtvlpalwfadgpsrvevsggtdnpsappadfirrvlepllak
igihqqttllrhgfypagggvvatevspvasfntlqlgergnivqmrgevllagvprhvaereiatlagsfsl
heqnihnlprdqgpgntvsleveseniterffvvgekrvsaevvaaqlvkevkrylastaavgeyladqlvlp
malagageftvahpscHlltniavverflpvrfslietdgvtrvsiegshhhhhh
>13PK_A
ekksinecdlkgkkvlirvdfnvpvkngkitndyRirsalptlkkvlteggscvlmshlgrpkgipmaqagki
rstggvpgfqqkatlkpvakrlselllrpvtfapdclnaadvvskmspgdvvllenvrfykeegskkakdrea
makilasygdvyisdafgtahrdsatmtgipkilgngaagylmekeisyfakvlgnpprplvaivggaKvsdk
iqlldnmlqridylliggamaytflkaqgysigkskceesklefarsllkkaedrkvqvilpidhvchtefka
vdsplitedqnipeghmaldigpktiekyvqtigkcksaiwngpmgvfemvpyskgtfaiakamgrgthehgl
msiiggGdsasaaelsgeakrmshvstggGaslellegktlpgvtvlddk
>1DTW_B
vahftfqpdpepreygqtqkmnlfqsvtsaldnslakdptavifgedvafggvfrctvglrdkygkdrvfntp
lcEqgivgfgigiavtgataiaeiqfadyifpafdqivneaakyryrsgdlfncgsltirspwgcvghgalyh
sqspeaffahcpgikvviprspfqakglllsciedknpciffepkilyraaaeevpiepyniplsqaeviqeg
sdvtlvawgtqvhvirevasmakeklgvscevidlrtiipwdvdticksviktgrllisheapltggfaseis
stvqeecflnleapisrvcgydtpfphifepfyipdkwkcydalrkminy
>1QF6_A
mpvitlpdgsqrhydhavspmdvaldigpglakaciagrvngelvdacdliendaqlsiitakdeegleiirh
scahllghaikqlwphtkmaigpvidngfyydvdldrtltqedvealekrmhelaeknydvikkkvswheare
tfanrgesykvsildeniahddkpglyfheeyvdmcrgphvpnmrfchhfklmktagaywrgdsnnkmlqriy
gtawadkkalnaylqrleeaakrdhrkigkqldlyhmqeeapgmvfwhndgwtifrelevfvrsklkeyqyqe
vkgpfmmdrvlwektghwdnykdamfttssenreycikpmncpghvqifnqglksyrdlplrmaefgschRne
psgslhglmrvrgftqddahifcteeqirdevngcirlvydmystfgfekivvklstrpekrigsdemwdrae
adlavaleennipfeyqlgegafygpkieftlydcldrawqcgtvqldfslpsrlsasyvgednerkvpvmih
railgsmerfigilteefagffptwlapvqvvimnitdsqseyvneltqklsnagirvkadlrnekigfkire
htlrrvpymlvcgdkevesgkvavrtrrgkdlgsmdvnevieklqqeirsrslkqlee
>2GQN_B
kkldtqlvnagrskkytlgavnsviqrasslvfdsveakkhatrnrangelfygRrgtlthfslqqamceleg
gagcvlfpcgaaavansilafieqgdhvlmtntaYepsqdfcskilsklgvttswfdpligadivkhlqpntk
ivflespgsitmevhdvpaivaavrsvvpdaiimiDntwaagvlfkaldfgidvsiqaatKylvghsdamigt
avcnarcweqlrenaylmgqmvdadtayitsrglrtlgvrlrqhhesslkvaewlaehpqvarvnhpalpgsk
ghefwkrdftgssglfsfvlkkklnneelanyldnfslfsmayswggyeslilanqpehiaairpqgeidfsg
tlirlhigledvddliadldagfariv
>1AUI_A
msepkaidpklsttdrvvkavpfppshrltakevfdndgkprvdilkahlmkegrleesvalriitegasilr
qeknlldidapvtvcgdihgqffdlmklfevggspantrylflgdyvDrgyfsiecvlylwalkilypktlfl
lrgnHecrhlteyftfkqeckikyservydacmdafdclplaalmnqqflcvhgglspeintlddirkldrfk
eppaygpmcdilwsdpledfgnektqehfthntvrgcsyfysypavceflqhnnllsilraheaqdagyrmyr
ksqttgfpslitifsapnyldvynnkaavlkyennvmnirqfncsphpywlpnfmdvftwslpfvgekvteml
vnvlnicsddelgseedgfdgataaarkevirnkiraigkmarvfsvlreesesvltlkgltptgmlpsgvls
ggkqtlqsatveaieadeaikgfspqhkitsfeeakgldrinermpprrdampsdanlnsinkaltsetngtd
sngsnssniq
>1S2K_A
tvesnwggailigsdfdtvsatanvpsasggssaagtawvgidgdtcqtailQtgfdwygdgtydawyewype
vsddfsgitisegdsiqmsvtatsdtsgsatlenlttgqkvsksfsnessgslcrtnaefiiEdfeecnsngs
dcefvpfasfspaveftdcsvtsdgesvslddaqitqviinnqdvtdcsvsgttvscsyv
>1H7O_A
mhtaefletepteissvlaggynhpllrqwqserqltknmlifplfisdnpddfteidslpninrigvnrlkd
ylkplvakglrsvilfgvplipgtkdpvgtaaddpagpviqgikfireyfpelyiicDvclceytshghcgvl
```

yddgtinrersvsrlaavavnyakagahcvapSdmidgrirdikrglinanlahktfvlsyaaKfsgnlygpf
rdaacsapsngdrkcyqlppagrglarralerdmsegadgiivKpstfyldimrdaseickdlpicayhvsge
yamlhaaaekgvvdlktiafeshqgflragarliitylapefldwldee
>1RDD_A
mlkqveiftdgsclgnpgpggygailryrgrektfsagytrttnnrmelmaaivalealkehcevilstdsqy
vrqgitqwihnwkkrgwktadkkpvknvdlwqrldaalgqhqikwewvkgHaghpenercdelaraaamnptl
edtgyqvev
>1XRS_B
mssglysmekkefdkvldlervkpygdtmndgkvqlsftlplknnersaeaakqialkmgleepsvvmqqsld
eeftffvvygnfvqsvnyneihveavnseilsmeetdeyikenigrkivvvgastgtdahtvgidaimnmKgy
aghygleryemidaynlgsqvanedfikkaveleadvllvsqtvtqknvhiqnmthlielleaeglrdrfvll
cggprinneiakelgydagfgpgrfaddvatfavktlndrmns
>1AQ0_A
igvcygmsannlpaastvvsmfksngiksmrlyapnqaalqavggtginvvvgapndvlsnlaaspaaaaswv
ksniqaypkvsfryvcvgnevaggatrnlvpamknvhgalvaaglghikvttsvsqailgvfsppsagsftge
aaafmgpvvqflartnaplmaniypylawaynpsamdmgyalfnasgtvvrdgaygyqnlfdttvdafytamg
khggssvklvvsEsgwpsgggtaatpanarfynqhlinhvgrgtprhpgaietyifamfnEnqKdsgvEqnwg
lfypnmqhvypinf
>1BHE_A
sdsrtvsepktpsscttlkadsstatstiqkalnncdqgkavrlsagstsvflsgplslpsgvsllidkgvtl
ravnnaksfenapsscgvvdkngkgcdafitavsttnsgiygpgtidgqggvklqdkkvswwelaadakvkkl
kqntprliqinksknftlynvslinspnfhvvfsdgdgftawkttiktpstarntDgidpmssknitiaysni
atgDDnvaikaykgraetrnisilhndfgtgHgmsigsetmgvynvtvddlkmngttnglriksdksaagvvn
gvrysnvvmknvakpividtvyekkegsnvpdwsditfkdvtsetkgvvvlngenakkpievtmknvkltsds
twqiknvnvkk
>1BOO_A
mlnfgkkpayttsngsmyigdslellesfpeesislvmtSPpfalqrkkeygnleqheyvdwflsfakvvnkk
lkpdgsfvvDfggaymkgvparsiynfrvlirmidevgfflaedfywfnpsklpspiewvnkrkirvkdavnt
vwwfsktewpksditkvlapysdrmkkliedpdkfytpktrpsghdigksfskdnggsippnllqisnsesng
qylancklmgikahparfpaklpeffirmltepddlvvdifggsnttglvaeresrkwisfemkpeyvaasaf
rfldnniseekitdiynrilngesldlnsii
>1TOX_A
gaddvvdssksfvmenfssyhgtkpgyvdsiqkgiqkpdddwkgfystdnkydaagysvdnenplsgkaggvv
kvtypgltkvlalkvdnaetikkelglslteplmeqvgteefikrfgdgasrvvlslpfaegsssvEyinnwe
qakalsveleinfetrgkrgqdamyeymaqacascinldwdvirdktktkieslkehgpiknkmsespnktvs
eekakqyleefhqtalehpelselktvtgtnpvfaganyaawavnvaqvidsetadnlekttaalsilpgigs
vmgiadgavhhnteeivaqsialsslmvaqaiplvgelvdigfaaynfvesiinlfqvvhnsynrpayspghk
tqpflhdgyavswntvedsiirtgfqgesghdikitaentplpiagvllptipgkldvnkskthisvngrkir
mrcraidgdvtfcrpkspvyvgngvhanlhvafhrsssekihsneissdsigvlgyqktvdhtkvnsklslff
eiks
>1QS0_A
neyaplrlhvpeptgrpgcqtdfsylrlndagqarkppvdvdaadtadlsyslvrvldeqgdaqgpwaedidp
qilrqgmramlktrifdsrmvvaqrqkkmsfymqslgeeaigsgqalalnrtdmcfptyrqqsilmardvslv
emicqllsnerdplkgrqlpimysvreagfftisgnlatqfvqavgwamasaikgdtkiasawigdgataesd
fhtaltfahvyrapvilnvvnnqwaistfqaiaggesttfagrgvgcgiaslrvdgndfvavyaasrwaaera
rrglgpsliewvtyragpHstsddpskyrpaddwshfplgdpiarlkqhlikighwseeehqattaefeaavi
aaqkeaeqygtlanghipsaasmfedvykempdhlrrqrqel
>1LWH_B
migyqiyvrsfrdgnldgvgdfrglknavsylkelgidfvwlmpvfssisfhgydvvdfysfkaeygserefk
emieafhdsgikvvldlpiHhtgflhtwfqkalkgdphyrdyyvwanketdlderrewdgekiwhpledgrfy
rglfgpfspdlnydnpqvfdemkrlvlhlldmgvdgfRfDaakhmrdtieqnvrfwkyflsdlkGiflaEIWa
earmvdehgrifgymlnfdtshcikeavwkentrvliesieraviakdylpvnfTsnHDmsrlasfeggfske
kiklsisilftlpgvplvfygdelgmkgvyqkpntevvldpfpwnesmcvegqtfwkwpayngpfsgisveyq

```
krdpdsilshtlgwtrfrkenqwidrakleflckedkflvyrlyddqhslkvfhnlsgeevvfegvkmkpykt
evv
>1T0U_A
msksdvfHlgltkndlqgatlaivpgdpdrvekiaalmdkpvklashrefttwraeldgkpvivcstgiggps
tsiaveElaqlgirtflrigttgaiqphinvgdvlvttasvrldgaslhfaplefpavadfecttalveaaks
igatthvgvtassdtfypgqerydtysgrvvrhfkgsmeewqamgvmnyemesatlltmcasqglragmvagv
ivnRtqqeipnaetmkqteshavkivveaarrll
>1C9U_A
dvpltpsqfakaksenfdkkvilsnlnkphallwgpdnqiwlteratgkilrvnpesgsvktvfqvpeivnda
dgqngllgfafhpdfknnpyiyisgtfknpkstdkelpnqtiirrytynkstdtlekpvdllaglpsskdHqs
grlvigpdqkiyytigDqgrnqlaylflpnqaqhtptqqelngkdyhtymgkvlrlnldgsipkdnpsfngvv
shiytlghrnpqglaftpngkllqseqgpnsddeinlivkggnygwpnvagykddsgyayanysaaanksikd
laqngvkvaagvpvtkesewtgknfvpplktlytvqdtynyndptcgemtyicwptvapssayvykggkkait
gwentllvpslkrgvifrikldptysttyddavpmfksnnryrdviaspdgnvlyvltdtagnvqkddgsvtn
tlenpgslikftykak
>2HI7_B
mlrflnqasqgrgawllmaftalaleltalwfqhvmllkpCvlCiyeRvalfgvlgaaligaiapktplryva
mviwlysafrgvqltyehtmlqlypspfatCdfmvrfpewlpldkwvpqvfvasgdsaerqwdflglempqwl
lgifiaylivavlvvisqpfkakkrdlfgr
>1CQQ_A
gpeeefgmslikhnscvittengkftglgvydrfvvvptHadpgkeiqvdgittkvidsydlynkngiklEit
vlkldrnekfrdirryipnneddypncnlallanqpeptiinvgdvvsygnillsgnqtarmlkysyptksGy
Cggvlykigqvlgihvggngrdgfsamllrsyft
>1E19_A
mgkrvvialggnalqqrgqkgsyeemmdnvrktarqiaeiiargyevvithgngpqvgslllhmdagqatygi
paqpmdvagamsqgwigymiqqalknelrkrgmekkvvtiitqtivdkndpafqnptKpvgpfydeetakrla
rekgwivkedsgrgwrrvvpspdpkghveaetikklvergviviasggggvpviledgeikgveavidKdlag
eklaeevnadifmiltdvngaalyygtekeqwlrevkveelrkyyeeghfkagsmgpKvlaairfiewggera
iiahlekavealegktgtqvlp
>1ORD_A
ssslkiastqearqyfdtdrvvvdavgsdftdvgaviamdyetdvidaadatkfgipvfavtkdaqaisadel
kkifhiidlenkfdatvnareietavnnyedsilppffkslkeyvsrgliqfdcpghqggqyyrkhpagrefy
dffgetvfradlcnadvalgdllihegpavaaekhaarvynadktyfvlggssnanntvtsalvsngdlvlfd
rnnHksvynsalamaggrpvylqtnrnpygfiggiydsdfdekkirelaakvdperakwkrpfrlaviqlgty
dgtiynahevvkrighlcdyiefDsawvgyeqfipmmrnsspllidd lgpedpgiivvqsvhKqqagfsqtsq
ihkkdshikgqlrycdhkhfnnsfnlfmstspfypmyaaldvnaamqegeagrklwhdllittiearkklika
gsmfrpfvppvvngkkwedgdtedmannidywrfekgakwhayegygdnqyyvdpnkfmlttpginpetgdye
dfgvpativanylrdhgiipeksdlnsilflmtpaetpakmnnlitqllqlqrlieedaplkqvlpsiyaane
eryngytirelcqelhdfyknnntftyqkrlflreffpeqgmlpyearqefirnhnklvplnkiegeialega
lpyppgvfcvapgekwsetavkyftilqdginnfpgfapeiqgvyfkqegdkvvaygevydaevaknddrynn
>2F61_A
arpcipksfgyssvvcvcnatycdsfdpptfpalgtfsryestrsgrrmelsmgpiqanhtgtglll tlqpeq
kfqkvkgfggamtdaaalnilalsppaqnlllksyfseegigyniirvpmascdfsirtytyadtpddfqlhn
fslpeedtklkiplihralqlaqrpvsllaspwtsptwlktngavngkgslkgqpgdiyhqtwaryfvkflda
yaehklqfwavtaenEpsagllsgypfqclgftpehqrdfiardlgptlansthhnvrllmlddqrlllphwa
kvvltdpeaakyvhgiavhwyldflapakatlgethrlfpntmlfasEaCvgskfweqsvrlgswdrgmqysh
siitnllyhvvgwtdwnlalnpeggpnwvrnfvdspiivditkdtfykqpmfyhlghfskfipegsqrvglva
sqkndldavalmhpdgsavvvvlnrsskdvpltikdpavgfletispgysihtylwhrq
>1A4S_A
aqlvdsmpsastgsvvvtddlnywggrrikskdgattepvfepatgrvlcqmvpcgaeevdqavqsaqaaylk
wskmagiersrvmleaariirerrdniaklevinngktiteaeydidaawqcieyyaglaptlsgqhiqlpgg
afaytrreplgvcagilawNypfmiaawkcapalacgnavvfkpspmtpvtgvilaeifheagvpvglvnvvq
ggaetgsllchhpnvakvsftgsvptgkkvmemsaktvkhvtlElggkspllifkdcelenavrgalmanflt
qgqvCtngtrvfvqreimpqfleevvkrtkaivvgdplltetrmggliskpqldkvlgfvaqakkegarvlcg
```

```
gepltpsdpklkngyfmspcvldncrddmtcvkeeifgpvmsvlpfdteeevlqranntfglasgvftrdis
rahrvaanleagtcyintysispvevpfggykmsgfgrengqatvdyysqlktvivemgdvdslf
>1YQS_A
lpapddtglqavlhtalsqgapgamvrvddngtihqlsegvadratgraitttdrfrvgSvtKsfsavvllql
vdegkldldasvntylpgllpddritvrqvmshrsglydytndmfaqtvpgfesvrnkvfsyqdlitlslkhg
vtnapgaaysYsNtnfvvagmliekltghsvateyqnriftplnltdtfyvhpdtvipgthangyltpdeagg
alvdsteqtvswaqsagavisstqdldtffsalmsgqlmsaaqlaqmqqwttvnstqgyglglrrrdlscgis
vygHtgtvqgYytyafaskdgkrsvtalantsnnvnvlntmartlesafcgkp
>1KEW_B
mkilitggagfigsavvrhiikntqdtvvnidkltyagnleslsdisesnrynfehadicdsaeitrifeqyq
pdavmhlaaeshvdrsitgpaafietnivgtyallevarkywsalgedkknnfrfhhisTDEvygdlphpdev
ensvtlplftettayapsSpYSasKassdhlvrawrrtyglptivtncsnnygpyhfpeKliplvilnalegk
plpiygkgdqirdwlyvedharalhmvvtegkagetynigghnekknldvvfticdlldeivpkatsyreqit
yvadrpghdrryaidagkisrelgwkpletfesgirktvewylantqwvnnvksgayqswieqnyegrq
>2CCD_A
mkypvegggnqdwwpnrlnlkvlhqnpavadpmgaafdyaaevatidvdaltrdieevmttsqpwwpadyghy
gplfiRmawHaagtyrihdgrggagggmqrfaplnswpDnasldkarrllwpvkkkygkklswadlivfagnc
alesmgfktfgfgfgrvdqwepdevywgkeatwlgderysgkrdlenplaavqmgliyvnpegpngnpdpmaa
avdiretfrrmamndvetaalivgghtfgkthgagpadlvgpepeaapleqmglgwkssygtgtgkdaittgi
evvWtntptkwdnsfleilygyeweltkspagawqytakdgagagtipdpfggpgrsptmlatdlslrvdpiy
eritrrwlehpeeladefakawyklihrdmgpvarylgplvpkqtllwqdpvpavshdlvgeaeiaslksqir
asgltvsqlvstawaaassfrgsdkrgganggrirlqpqvgwevndpdgdlrkvirtleeiqesfnsaapgni
kvsfadlvvlggcaaiekaakaaghnitvpftpgrtdasqeqtdvesfavlepkadgfrnylgkgnplpaeym
lldkanlltlsapemtvlvgglrvlganykrlplgvfteasesltndffvnlldmgitwepspaddgtyqgkd
gsgkvkwtgsrvdlvfgsnselralvevygaddaqpkfvqdfvaawdkvmnldrfdvr
>1DAA_A
gytlwndqivkdeevkidkedrgyqfgdgvyevvkvyngemftvnehidrlyasaekiritipytkdkfhqll
helveknelntghiyfqvtrgtsprahqfpentvkpviigytkenprplenlekgvkatfvedirwlrcdiKs
lnllgavlakqeahekgcyeailhrnntvtEgsssnvfgikdgilythpannmiLkgitrdvviacaneinmp
vkeipftthealkmdelfvtsttseitpvieidgklirdgkvgewtrklqkqfetkipkplhi
>4H2B_A
pweltilhtndvhsrleqtsedsskcvdasrcmggvarlftkvqqirraepnvllldagdqyqgtiwftvykg
aevahfmnalrydamalgNHefDngvegliepllkeakfpilsanisasgplasqisglylpykvlpvgdevv
givgytsketpflsnpgtnlvfedeitalqpevdklktlnvnkiialghsgfemdkliaqkvrgvdvvvgghs
ntflytgnppskevpagkypfivtsddgrkvpvvqayafgkylgylkiefdergnvisshgnpilldssiped
psikadinkwriklddystqelgktivyldgsSqscRfrecnmgnlicdaminnnlrhademfwnhvsmciln
gggiRspiderndgtitwenlaavlpfggtfdlvqlkgstlkkafehsvhrygqstgeflqvggihvvydlsr
kpgdrvvkldvlctscrvpsydplkmdevykvilpnflanggdgfqmikdellrhdsgdqdinvvstyiskmk
viypavegrikfs
>1H19_A
mpeivdtcslaspasvcrtkhlhlrcsvdftrrtltgtaaltvqsqednlrslvldtkdltiekvvingqevk
yalgerqsykgspmeislpialsknqeivieisfetspkssalqwltpeqtsgkehpylfsqcqaihcrailp
cqdtpsvkltytaevsvpkelvalmsairdgetpdpedpsrkiykfiqkvpipcylialvvgalesrqigprt
lvwsekeqveksayefsetesmlkiaedlggpyvwgqydllvlppsfpyggmQnpcltfvtptllagdkslsn
viahEishswtgnlvtnktwdhfwlneghtvylerhicgrlfgekfrhfnalggwgelqnsvktfgethpftk
lvvdltdidpdvayssvpYekgfallfyleqllggpeiflgflkayvekfsyksittddwkdflysyfkdkvd
vlnqvdwnawlyspglppikpnydmtltnacialsqrwitakeddlnsfnatdlkdlsshqlneflaqtlqra
plplghikrmqevynfnainnseirfrwlrlciqskwedaiplalkmateqgrmkftrplfkdlaafdkshdq
avrtyqehkasmhpvtamlvgkdlkvd
>1TML_A
ndspfyvnpnmssaewvrnnpndprtpvirdriasvpqgtwfahhnpgqitgqvdalmsaaqaagkipilvvy
napgrdcgnhssggapshsayrswidefaaglknrpayiivepDlislmsscmqhvqqevletmayagkalka
gssqariyfdaghsawhspaqmaswlqqadisnsahgiatntsnyrwtadevayakavlsaignpslravidt
srngngpagnewcdpsgraigtpsttntgdpmidaflwiklpgeaDgciagagqfvpqaayemaiaa
```

>1QAM_A
mneknikhsqnfitskhnidkimtnirlnehdnifeiGsgkghftlelvqrcnfvtaiEidhklckttenklv
dhdnfqvlnkdilqfkfpknqsykifgNipynistdiirkivfdsiadeiyliveygfakrllntkrslalfl
maevdisilsmvpreyfhpkpkvnsslirlnrkksrishkdkqkynyfvmkwvnkeykkiftknqfnnslkha
giddlnnisfeqflslfnsyklfnk
>1JJI_A
mldmpidpvyyqlaeyfdslpkfdqfssareyreainriyeernrqlsqhervervedrtikgrngdirvrvy
qqkpdspvlvyyhgggfvicsieshdalcrriarlsnstvvsvdyrlapehkfpaavydcydatkwvaenaee
lridpskifvggdSaggnlaaavsimardsgedfikhqiliypvvnfvaptpsllefgeglwildqkimswfs
eqyfsreedkfnplasvifadlenlppaliitaeyDplrdegevfgqmlrragveasivryrgvlHgfinyyp
vlkaardainqiaallvfd
>1OAS_A
skiyednsltightplvrlnrigngrilakvesrnpsfsvKcriganmiwdaekrgvlkpgvelveptngntg
ialayvaaargykltltmpetmsierrkllkalganlvltegakgmkgaiqkaeeivasdpqkylllqqfsnp
anpeihekttgpeiwedtdgqvdvfisgvgtggtltgvtryikgtkgktdlitvaveptdspviaqalageei
kpgphkiqgigagfipgnldlklidkvvgitneeaistarrlmeeegilagiSsgaavaaalklqedesftnk
nivvilpssgerylstalfadlftekelqq
>1W2N_A
mgsshhhhhhssglvprgshmdttvptfslaelqqglhqdefrrclrdkglfyltdcgltdtelksakdlvid
ffehgseaekravtspvptmRrgftglesestaqitntgsysdysmcysmgtadnlfpsgdferiwtqyfdrq
ytasravarevlratgtepdggveafldcepllRfrYfpqvpehrsaeeqplrMaphydlsmvtliqqtpcan
gfvslqaevggaftdlpyrpdavlvfcgaiatlvtggqvkaprhhvaaprrdqiagssrtssvfflrpnadft
fsvplarecgfdvsldgetatfqdwiggnyvnirrtska
>1HKA_A
tvayiaigsnlaspleqvnaalkalgdipeshiltvssfyrtpplgpqdqpdylnaavaletslapeellnht
qrielqqgRvrkaerwgpRtldldimlfgnevinterltvphydmknrgfmlwplfeiapelvfpdgemlrqi
lhtrafdklnkw
>1LYA_A
gpipevlknymdaqyygeigigtppqcftvvfDtgssnlwvpsihcklldiacwihhkynsdksstyvkngts
fdihygsgslsgylsqdtvsvpcq
>1JH6_A
meevkkdvysvwalpdeeseprfkklmealrseftgprfvpHvtvavsayltadeakkmfesacdglkaytat
vdrvstgtfffqcvflllqttpevmeagehcknhfncstttpyMpHlSllYaelteeekknaqekaytldssl
dglsfrlnrlalcktdtedktletwetvavcnlnpgshhhhhh
>1E5T_A
mlsfqypdvyrdetaiqdyhghkvcdpyawledpdseqtkafveaqnkitvpfleqcpirglykermtelydy
pkyschfkkgkryfyfyntglqnqrvlyvqdslegearvfldpnilsddgtvalrgyafsedgeyfayglsas
gsdwvtikfmkvdgakelpdvlervkfscmawthdgkgmfynaypqqdgksdgtetstnlhqklyyhvlgtdq
sedilcaefpdepkwmggaelsddgryvllsiregtdpvnrlwycdlqqesngitgilkwvklidnfegeydy
vtnegtvftfktnrhspnyrlinidftdpeeskwkvlvpehekdvlewvacvrsnflvlcylhdvkntlqlhd
latgallkifplevgsvvgysgqkkdteifycftsflspgiiyhcdltkeeleprvfrevtvkgidasdyqtv
qifypskdgtkipmfivhkkgikldgshpaflygyggfnisitpnysvsrlifvrhmggvlavanirgggeyg
etwhkggilankqncfddfqcaaeylikegytspkrltinggSngglllvatcanqrpdlfgcviaqvgvmdml
kfhkytighawttdygcsdskqhfewlikysplhnvklpeaddiqypsmllltadhDdrvvplhslkfiatlq
yivgrsrkqnnpllihvdtkagHgagmptakvieevsdmfafiarclnidwip
>1KQF_C
mskskmivrtkfidrachwtvvicfflvalsgisfffptlqwltqtfgtpqmgrilhpffgiaifvalmfmfv
rfvhhnipdkkdipwllnivevlkgnehkvadvgkynagqkmmfwsimsmifvllvtgviiwrpyfaqyfpmq
vvrysllihaaagiilihailiHmymafwvkgsikgmiegkvsrrwakkhhprwyreiekaeakkeseegi
>3M0C_B
sipwnleritppryrggslvevyllDtsiqsdhreiegrvmvtdfenvpeedaskcdsHgthlagvvsgrdag
vakgasmrslrvlncqgkgtvsgtliglefirksqlvqpvgplvvllplaggysrvlnaacqrlaragvvlvt
aagNfrddaclyspasapevitvgatnaqdqpvtlgtlgtnfgrcvdlfapgediigassycstcfvsqsgtS
qaaahvagiaammlsaepeltlaelrqrlihfsakdvineawfpedqrvltpnlvaalppsthgwqlfcrtvw

sahsgptrmataiarcapdeellscssfsrsgkrrgermeaqggklvcrahnafggegvyaiarccllpqanc
svhtappaeasmgtrvhchqqghvltgcsshwevedlqpnqcvghreasihascchapgleckvkehgipqeq
vtvaceegwtltgcsalpshvlgayavdntcvvrsravtavaiccrsr
>2PLC_A
vttkqwmsalpdttnlaalsipgtHDtmsyngditwtltkplaqtqtmslyqqleagiryidiRakdnlniyH
gpiflnaslsgvletitqflkknpketiimrlkdeqnsndsfdyriqpliniykdyfyttprtdtsnkiptlk
dvrgkilllsenhtkkplvinsrkfgmqfgapnqviqddyngpsvktkfkeivqtayqaskadnklflnhisa
tsltftprqyaaalnnkveqfvlnltsekvrglgilimDfpekqtikniiknnkf
>1GT7_P
mqnitqswfvqgmikattdawlkgwdernggnltlrlddadiapyhdnfhqqpryiplsqpmpllantpfivt
gsgkffrnvqldpaanlgivkvdsdgagyhilwgltneavptsElpahflshcerikatngkdrvimhchatn
lialtyvlendtavftrqlwegstEclvvfpdgvgilpwmvpgtdeigqataqemqkhslvlwpfhgvfgsgp
tldetfglidtaeksaqvlvkvysmggmkqtisreelialgkrfgvtplasalal
>1DAE_A
skryfvtgtdTevgKtvascallqaakaagyrtagyKpvaSgsektpeglrnsdalalqrnsslqldyatvnp
ytfaeptsphiisaqegrpieslvmsaglraleqqadwvlvegaggwftplsdtftfadwvtqeqlpvilvvg
vklgcinhamltaqviqhagltlagwvandvtppgkrhaeymttltrmipapllgeipwlaenpenaatgkyi
nlall
>1LJL_A
mdkktiyfiCtGNScRSqmaegwgkeilgegwnvysagiethgvnpkaieamkevdidisnhtsdlidndilk
qsdlvvtlCsdadnnCpilppnvkkehwgfdDpagkewsefqrvrdeiklaiekfklr
>1NML_A
dnlmeransmfepipkyppvidgneltqakvelgkmeffeprlssshliscntchnvglggddelptsighgw
qkgprnsptvfnavfnaaqfWdgraadlaeqakgpvqagvEmsstpdrvvatlksmpeyierfedafpgqenp
vtfdnmavaieayeatlitpeapfdkylrgdtsalnesekeglalfmdrgctachsgvnlggqnyypfglvak
pgaeilpegdkgrfsvtetasdeyvfrasplrnieltapyfhsgavwsleeavavmgtaqlgtelnndevksi
vaflktltgnvpevtypvlppstantpkpvdmip
>1AUG_A
mekkvlltgfdpfggetvnpsweavkrlngaaegpasivseqvptvfykslavlreaikkhqpdiiicvgqag
grmqitpErvainlneaRipdnegnqpvgedisqggpaaywtglpikriveeikkegipaavsytagtfvCnh
lfyglmdeisrhhphirggfiHipyipeqtlqksapslsldhitkalkiaavtaavheddietgggelh
>1AFW_A
knsllekrpedvvivaanrsaigkgfkgafkdvntdyllynflnefigrfpeplradlnlieevacgnvlnvg
agatehraaclasgipystpfvalnrqCssgltavndiankikvgqidiglalgvesmtnnyknvnplgmiss
eelqknreakkclipmgitnenvaanfkisrkdqdefaansyqkaykakneglfedeilpiklpdgsicqsde
gprpnvtaeslssirpafikdrgtttagnasqvsdgvagvllarrsvanqlnlpvlgryidfqtvgvppeimg
vgpayaipkvleatglqvqdidifeineafaaqalycihklgidlnkvnprggaialgHplgctgarqvatil
relkkdqigvvsmCiGtgmgaaaifike
>1TDJ_A
madsqplsgapegaeylravlrapvyeaaqvtplqkmeklssrldnvilvkredrqpvhsfKlrgayammagl
teeqkahgvitasagnhaqgvafssarlgvkalivmptatadikvdavrgfggevllhganfdeakakaiels
qqqgftwvppfdhpmviagqgtlalellqqdahldrvfvpvggglaagvavlikqlmpqikviaveaedsac
lkaaldaghpvdlprvglfaegvavkrigdetfrlcqeylddiitvdsdaicaamkdlfedvravaepsgala
lagmkkyialhnirgerlahilSganvnfhglryvsercelgeqreallavtipeekgsflkfcqllggrsvt
efnyrfadaknacifvgvrlsrgleerkeilqmlndggysvvdlsddemaklhvrymvggrpshplqerlysf
efpespgallrflntlgtywnislfhyrshgtdygrvlaafelgdhepdfetrlnelgydchdetnnpafrff
lag
>1F8R_A
addrnplaecfqendyeefleiarnglkatsnpkhvvivgagmaglsaayvlagaghqvtvleaserpggrvr
tyrneeagwyanlgpmrlpekhrivreyirkfdlrlnefsqendnawyfiknirkkvgevkkdpgllkypvkp
seagksagqlyeeslgkvveelkrtncsyilnkydtystkeylikegdlspgavdmigdllnedsgyyvsfie
slkHddifayekrfdeivdgmdklptamyrdiqdkvhfnaqvikiqqndqkvtvvyetlsketpsvtadyviv
cttsravrlikfnppllpkkahalrsvhyrsgtKifltcttkfweddgihggksttdlpsrfiyypnhnftng

```
vgviiaygigddanffqaldfkdcadivfndlslihqlpkkdiqsfcypsviqkwsldkyamggittftpyqf
qhfsdpltasqgriyfageytaqahgwidstiksglraardvnlasenpsgihlsndnel
>1YSC_A
kikdpkilgidpnvtqytgyldvededkhfffwtfesrndpakdpvilwlnggpgcssltglffelgpssigp
dlkpignpyswnsnatvifldqpvnvgfsysgssgvsntvaagkdvynflelffdqfpeyvnkgqdfhiageS
yaghyipvfaseilshkdrnfnltsvligngltdpltqynyyepmacgeggepsvlpseecsamedslerclg
liescydsqsvwscvpatiycnnaqlapyqrtgrnvydirkdceggnlcyptlqdiddylnqdyvkeavgaev
dhyescnfdinrnflfagdwmkpyhtavtdllnqdlpilvyagdkDficnwlgnkawtdvlpwkydeefasqk
vrnwtasitdevagevksykhftylrvfnggHmvpfdvpenalsmvnewihggfsl
>1TYF_M
srgersfdiysrllkervifltgqvedhmanlivaqmlfleaenpekdiylyinspgGvitagmsiydtmqfi
kpdvsticmgqaaSMgaflltagakgkrfclpnsrvmiHqplggyqgqatdieihareilkvkgrmnelmalh
tgqsleqierdterDrflsapeaveyglvdsilthrn
>1G64_A
msderyqqrqqkvkdrvdarvaqaqeergiiivftgngkgKtTaafgtaaravghgknvgvvqfikgtwpnge
rnllephgvefqvmatgftwetqnreadtaacmavwqhgkrmladplldmvvldeltymvaydylpleevisa
lnarpghqtviitgrgchrdildladtvselrpvkhafdagvkaqmgidy
>1NZY_A
myeaighrvedgvaeitiklprhrnalsvkamqevtdalnraeeddsvgavmitgaedafcagFylreipldk
gvagvrdhfriaalwwHqmihkiirvkrpvlaaingvaagGglgislasdmaicadsakfvcaWhtigignDt
atsyslarivgmrramelmltnrtlypeeakdwglvsrvvypkdefrevawkvarelaaapthlqvmakerfha
gwmqpveectefeiqnviasvthphfmpcltrfldghradrpqvelpagv
>1UW8_A
mkkqndipqpirgdkgatvkiprnierdrqnpdmlvppetdhgtvsnmkfsfsdthnrlekggyarevtvrel
pisenlasvnmrlkpgaiRelhwhkeaewaymiygsarvtivdekgrsfiddvgegdlwyfpsglphsiqale
egaefllvfddgsfsEnstfqltdwlahtpkeviaanfgvtkeeisnlpgkekyifenqlpgslkddivegpn
gevpypftyrlleqepieseggkvyiadstnfkvsktiasalvtvepgamrelhwhpnthewqyyisgkarmt
vfasdghartfnyqagdvgyvpfamghyvenigdeplvfleifkddhyadvslnqwlamlpetfvqahldlgk
dftdvlskekhpvvkkkcsk
>4E7D_A
mdkfrvqgptrlqgevtisgakNaalpilfaallaeepveiqnvpklkdidttmklltqlgtkvergsvwida
snvnnfsapydlvktmrasiwalgplvarfgqgqvslpggCaigarpvdlhifgleklgaeikleegyvkasv
ngrlkgahivmdkvsvgatvtimsaatlaegttiienaarepeivdtanflvalgakisgqgtdritiegver
lgggvyrvlpdrietgtflvaaaisggkivcrnaqpdtldavlaklreagadietgedwisldmhgkrpkavt
vrtaphpafptDmqaqftllnlvaegtgvitetifenrfmhvpelirmgahaeiesntvichgveklsgaqvm
atdLrasaslvlagciaegttvvdriyhidRgyeriedklralganiervkge
>1VLB_A
miqkvitvngieqnlfvdaeallsdvlrqqlgltgvkvgceqgqcgacsvildgkvvracvtkmkrvadgaqi
ttiegvgqpenlhplqkawvlhggaqcgfcspgfivsakglldtnadpsredvrdwfqkhrnacrctgykplv
davmdaaavingkkpetdlefkmpadgriwgskyprptavakvtgtldygadlglkmpagtlhlamvqakvsh
anikgidtsealtmpgvhsvithkdvkgknritglitfptnkgdgwdrpilcdekvfqygdcialvcadsean
araaaekvkvdleelpaymsgpaaaaedaieihpgtpnvyfeqpivkgedtgpifasadvtvegdfyvgrqph
mpiepdvafaymgddgkcyihsksigvhlhlymiapgvglepdqlvlvanpmggtfgykfsptsealvavaam
atgrpvhlrynyqqqqytgkrspwemnvkfaakkdgtllamesdwlvdhgpysefgdlltlrgaqfigagyn
ipnirglgrtvatnhvwgsafrgygapqsmfaseclmdmlaeklgmdplelryknayrpgdtnptgqepevfs
lpdmidqlrpkyqaalekaqkestathkkgvgisigvygsgldgpdaseawaelnadgtitvhtawedhgqga
digcvgtahealrpmgvapekikftwpntattpnsgpsggsrqqvmtgnairvacenllkacekpgggyytyd
elkaadkptkitgnwtasgathcdavtglgkpfvvymygvfmaevtvdvatgqttvdgmtlmadlgslcnqla
tdgqiygglaqgiglalsedfedikkhatlvgagfpfikqipdkldivyvnhprpdgpfgasgvgElpltsph
aaiinaiksatgvriyrlpaypekvlealka
>1BHG_A
lglqggmlypqespsreckeldglwsfradfsdnrrrgfeeqwyrrplwesgptvdmpvpssfndisqdwrlr
hfvgwvwyerevilperwtqdlrtrvvlrigsahsyaivwvngvdtleheggylpfeadisnlvqvgplpsrl
ritiainntltpttlppgtiqyltdtskypkgyfvqntyfdffnyaglqrsvllyttpttyidditvttsveq
```

dsglvnyqisvkgsnlfklevrlldaenkvvangtgtqgqlkvpgvslwwpylmherpaylyslevqltaqts
lgpvsdfytlpvgirtvavtksqflingkpfyfhgvnkhedadirgkgfdwpllvkdfnllrwlganafrtsh
ypyaeevmqmcdrygivvidecpgvglalpqffnnvslhhhmqvmeevvrrdknhpavvmwsvanEpashles
agyylkmviahtksldpsrpvtfvsnsnyaadkgapyvdviclnsYyswyhdyghleliqlqlatqfenwykk
yqkpiiqsEygaetiagfhqdpplmfteeyqkslleqyhlgldqkrrkyvvgeliwnfadfmteqsptrvlgn
kkgiftrqrqpksaafllrerywkianet
>1EYP_A
maasitaitvenleypavvtspvtgksyflggagergltiegnfikfTaigvylediavaslaakwkgkssee
lletldfyrdiisgpfeklirgskirelsgpeYsrkvmeNcvahlksvgtygdaeaeamqkfaeafkpvnfpp
gasvfyrqspdgilglsfspdtsipekeaalienkavssavleTmigehavspdlkrclaarlpallnegafk
ign
>2VK8_D
seitlgkylferlkqvnvntvfglpgDfNlslldkiyevegmrwagnanElnaayaadgyarikgmsciittF
gvgelsalngiagsyaehvgvlhvvgvpsisaqakqlllHHtlgngdftvfhrmsanisettamitdiatapa
eidrcirttyvtqrpvylglpanlvdlnvpakllqtpidmslkpndaesekevidtilvldkdaknpvilada
ccsrhdvkaetkklidltqfpafvtpmgkgsideqhpryggvyvgtlskpevkeavesadlilsvgallsdfn
tgsfsysyktknivefhsdhmkirnatfpgvqmkfvlqklltiadaakgykpvavpartpanaavpastplk
qewmwnqlgnflqegdvviaeTgtsafginqttfpnntygisqvlwGsigftTgatlgaafaaeeidpkkrvi
lfigdgslqltvqeistmirwglkpylfvlnndgytiqklihgpkaqyneiqgwdhlsllptfgakdyethrv
attgewdkltqdksfndnskirmievmlpvfdapqnlveqakltaatnakq
>1FJS_A
ivggqeckdgecpwqallineenegfcggtilsefyiltaaHclyqakrfkvrvgdrnteqeeggeavhevev
vikhnrftketydfDiavlrlktpitfrmnvapaclperdwaestlmtqktgivsgfgrthekgrqstrlkml
evpyvdrnscklsssfiitqnmfcagydtkqedacqGdSGgphvtrfkdtyfvtgivSwgegcarkgkygiyt
kvtaflkwidrsmkt
>1ECF_A
Cgivgiagvmpvnqsiydaltvlqhrgqdaagiitidanncfrlrkanglvsdvfearhmqrlqgnmgighvr
yptagsssaseaqpfyvnspygitlahNGnltnahelrkklfeekrrhinttsdseillnifaseldnfrhyp
leadnifaaiaatnrlirgayacvamiighgmvafrdpngirplvlgkrdidenrteymvasesvaldtlgfd
flrdvapgeaiyiteegqlftrqcadnpvsnpclfeyvyfarpdsfidkisvysarvnmgtklgekiarewed
ldidvvipipetscdialeiarilgkpyrqgfvknryvgrtfimpgqqlrrksvrrklnanraefrdknvllv
ddsivrgttseqiiemareagakkvylasaapeirfpnvygidmpsateliahgrevdeirqiigadglifqd
lndlidavraenpdiqqfecsvfngvyvtkdvdqgyldfldtlrnddakavqrqnevenlemhneg
>1I8D_A
mFtgivqgtaklvsidekpnfrthvvelpdhmldgletgaSvahngcCltvteingnhvsfdlMketlritnl
gdlkvgdwvnveraakfsdeigghlmsgHimttaevakiltsennrqiwfkvqdsqlmkyilykgfigidgis
ltvgevtptrfcvhlipetlerttlgkkklgarvnieidpqtqavvdtvervlaarenamnqpgtea
>1A2T_A
atstkklhkepatlikaidgdtcklmykgqpmtfRlllvdtpetkhpkkgvekygpeasaftkkmvenakkie
vefdkgqrtdkygRglayiyadgkmvnealvrqglakvayvykpnntheqhlrkseaqakkeklniwsednad
sgq
>1POX_A
tnilagaavikvleawgvdhlygipgGsiNsImdalsaerdrihyiqvrhEevgamaaaadakltgkigvcFg
sagpggthlmnglydaredhvpvlaligqfgttgmnmdtFQemnenpiyadvadynvtavnaatlphvideai
rrayahqgvavvqipvdlpwqqisaedwyasannyqtpllpepdvqavtrltqtllaaerpliyygigarkag
keleqlsktlkiplmstypakgivadrypaylgsanrvaqkpanealaqadvvlfvgnnypfaevskafkntr
yflqididpaklgkrhktdiavladaqktlaailaqvserestpwwqanlanvknwraylasledkqegplqa
yqvlravnkiaepdaiysidVgdinlnanrhlkltpsnrhitsnlfAtMgvgipgaiaaklnyperqvfnlag
dggasmtmqdlvtqvqyhlpvinvvftncqygFIkdeqedtnqndfigvefndidfskiadgvhmqafrvnki
eqlpdvfeqakaiaqhepvlidavitgdrplpaeklrldsamssaadieafkqryeaqdlqplstylkqfgld
d
>1RO7_A
mkkviiagngpslkeidysrlpndfdvfrcnqfyfedkyylgkkckavfynpslffeqyytlkhliqnqeyet
elimcsnynqahlenenfvktfydyfpdahlgydffkqlkdfnayfkfheiyfnqRitsgvymcavaialgyk

eiylsgidfYqngssYafdtkqknllklapnfkndnshyigHskntdikaleflektykiklyclcpnsllan
fielapnlnsnfiiqeknnytkdilipsseaygkfsknin
>1G24_A
aysntyqeftnidqakawgnaqykkyglsksekeaivsytksaseingklrqnkgvingfpsnlikqvelldk
sfnkmktpenimlfrgddpaylgtefqntllnsngtinktafekakakflnkdrleygyistslmnvsqfagr
piitkfkvakgskagyidpisafagqlEmllprhstyhiddmrlssdgkqiiitatmmgtainpk
>1OCX_A
stekekmiagelyrsadetlsrdrlrarqlihrynhslaeehtlrqqiladlfgqvteayieptfrcdygyni
flgnnffanfdcvmldvcpirigdncmlapgvhiyTathpidpvarnsgaelgkpvtignnvwiggravinpg
vtigdnvvvasgavvtkdvpdnvvvggnpariikkl
>1PII_A
mqtvlakivadkaiwvearkqqqplasfqnevqpstrhfydalqgartafilEcKkaspskgvirddfdpari
aaiykhyasaisvltdekyfqgsfnflpivsqiapqpilcKdfiidpyqiylaryyqadacllmlsvldddqy
rqlaavahslemgvltEvsneeeqeraialgakvvgiNnrdlrdlsidlnrtrelapklghnvtviseSgint
yaqvrelshfangfligsalmahddlhaavrrvllgenkvcgltrgqdakaaydagaiygglifvatsprcvn
veqaqevmaaaplqyvgvfrnhdiadvvdkakvlslaavqlhgneeqlyidtlrealpahvaiwkalsvgetl
parefqhvdkyvldngqggsgqrfdwsllngqslgnvllagglgadncveaaqtgcagldfnsavesqpgikd
arllasvfqtlray
>1GAL_A
sngieaslltdpkdvsgrtvdyiiaggg ltglttaarltenpnisvlviesgsyesdrgpiiedlnaygdifg
ssvdhayetvelatnnqtalirsgnglggstlvnggtwtrphkaqvdswetvfgnegwnwdnvaayslqaera
rapnakqiaaghyfnaschgvngtvhagprdtgddyspivkalmsavedrgvptkkdfgcgdphgvsmfpntl
hedqvrsdaarewllpnyqrpnlqvltgqyvgkvllsqngttpravgvefgthkgnthnvyakhevllaagsa
vsptileysgigmksileplgidtvvdlpvglnlqdqttatvrsritsagagqgqaawfatfnetfgdyseka
hellntkleqwaeeavarggfhnttalliqyenyrdwivnhnvaysElfldtagvasfdvwdllpftrgyvhi
ldkdpylhhfaydpqyflneldllgqaaatqlarnisnsgamqtyfagetipgdnlaydadlsawteyipyhf
rpnyHgvgtcsmmpkemggvvdnaarvygvqglrvidgsipptqmssHvmtvfyamalkisdailedyasmq
>4B73_A
gsvvivgriilsgsgsitaysqqtrgllgciitsltgrdknqvegevqvvstatqsflatcvngvcwtvyHga
gsktlagpkgpitqmytnvdqDlvgwqappgarsltpctcgssdlylvtrhadvipvrrrgdsrgsllsprpv
sylkGsSggpllcpsghavgifraavctrgvakavdfvpvesmettmrspvftdnssppavpqsfqvahlhap
tgsgkstkvpaayaaqgykvlvlnpsvaatlgfgaymskahgidpnirtgvrtittgapvtystygkfladgg
csggaydiiicdechstdsttilgigtvldqaetagarlvvlatatppgsvtvphpnieevalsntgeipfyg
kaipieairggrhlifchskkkcdelaaklsglginavayyrgldvsviptigdvvvvatdalmtgytgdfds
vidcntcvtqtvdfsldptftietttvpqdavsrsqrrgrtgrgrrgiyrfvtpgerpsgmfdssvlcecyda
gcawyeltpaetsvrlraylntpglpvcqdhlefwesvftglthidahflsqtkqagdnfpylvayqatvcar
aqapppswdqmwkclirlkptlhgptpllyrlgavqnevtlthpitkyimacmsadlevvt
>1QZ9_A
mttrndclaldaqdslaplrqqfalpegviyldgnslgarpvaalaraqaviaeewgnglirswnsagwrdls
erlgnrlatligardgevvvtdttsinlfkvlsaalrvqatrsperrvivtetsnFptdlyiaegladmlqqg
ytlrlvdspeelpqaidqdtavvmlthvnyktgymhdmqaltalshecgalaiwDlahsagavpvdlhqagad
yaigctyKylnggpgsqafvwvspqlcdlvpqplsgwfghsrqfamepryepsngiarylcgtqpitslamve
cgldvfaqtdmaslrrkslaltdlfielveqrcaaheltlvtprehakrgshvsfehpegyaviqalidrgvi
gdyreprimrfgftplyttftevwdavqilgeildrktwaqaqfqvrhsvt
>1KC7_A
akwvykfeegnasmrnllggKgcnlaemtilgmpipqgftvtteacteyynsgkqitqeiqdqifeaitwlee
lngkkfgdtedpllvsvrsgarasmpGmMdtilnlglndvavegfakktgnprfaydsyrrfiqmysdvvmev
pkshfekiidamkeekgvhfdtdltaddlkelaekfkavykeamngeefpqepkdqlmgavkavfrswdnpra
ivyrrmndipgdwgtavnvqtmvfgnkgetsgtgvaftrnpstgekgiygeylinaqgedvvagvrtpqpitq
lendmpdcykqfmdlamklekhfrdmqdmeftieegklyflqtRngkrtapaalqiacdlvdegmiteeeavv
rieaksldqllhptfnpaalkagevigsalpaspgaaagkvyftadeakaahekgervilvrletspediegm
haaegiltvrggmtsHaavvargmgtccvsgcgeikineeaktfelgghtfaegdyisldgstgkiykgdiet
qeasvsgsferimvwadkfrtlkvrtnadtpedtlnavklgaegiglcrtehmffeadrimkirkmilsdsve
areealnelipfqkgdfkamykalegrpmtvryldpplhefvphteeeqaelaknmgltlaevkakvdelhef

```
npmmghrgcrlavtypeiakmqtravmeaaievkeetgidivpeimiplvgekkelkfvkdvvvevaeqvkke
kgsdmqyhigtmieipraaltadaiaeeaeffSfgtndltqmtfgfsrddagkfldsyykakiyesdpfarld
qtgvgqlvemavkkgrqtrpglkcgiCgehggdpssvefchkvglnYvscspfrvpiarlaaaqaalnn
>1NIR_B
kaaeqyqgaasavdpahvvrtngapdmsesefneakqiyfqrcagchgvlrkgatgkpltpditqqrgqqyle
alitygtplgmpnwgssgelskeqitlmakyiqhtppqppewgmpemreswkvlvkpedrpkkqlndldlpnl
fsvtlrdagqialvdgdskkivkvidtgyavhisrmsasgryllvigrdaridmidlwakeptkvaeikigie
arsvesskfkgyedrytiagaywppqfaimdgetlepkqivstrgmtvdtqtyhpeprvaaiiashehpefiv
nvketgkvllvnykdidnltvtsigaapflHdggwdsshryfmtaannsnkvavidskdrrlsalvdvgktpH
pgrganfvhpkygpvwstshlgdgsisligtdpknhpqyawkkvaelqgqgggslfikthpksshlyvdttfn
pdarisqsvavfdlknldakyqvlpiaewadlgegakrvvqpeynkrgdevwfsvwngkndssalvvvddktl
klkavvkdprlitptgkfnvyntqhdvy
>1G4P_A
hgirmtrisremmkellsvyfimgsnntkadpvtvvqkalkggatlyqfrekggdaltgearikfaekaqaac
reagvpfivnddvelalnlkadgihigqedanakevraaigdmilgvAahtmsevkqaeedgadyvglgpiyp
tetkkdtravqgvslieavrrqgisipivgiggitidnaapviqagadgvsmisaisqaedpesaarkfreei
qtyktgr
>1PNT_A
aeqvtksvlfvClgniCRspiaeavfrklvtdqnisdnwvidsgavsdwnvgrspdpravsclrnhgintahk
arqvtkedfvtfdyilcmdesnlrdlnrksnqvkncrakiellgsydpqkqliieDpyygndadfetvyqqcv
rccraflekvr
>1IU4_A
dsddrvtppaepldrmpdpyrpsygraetvvnnyirkwqqvyshrdgrkqqmteeqrewlsygCvgvtwvnsg
qyptnrlafasfdedrfknelkngrprsgetraefegrvakesfdeekgfqrarevasvmnralenahdesay
ldnlkkelangndalrnedarspfysalrntpsfkernggnhdpsrmkaviyskhfwsgqdrsssadkrkygd
pdafrpapgtglvdmsrdrniprsptspgegfvnfDygwfgaqteadadKtvWtHgnhyhapngslgamhvye
skfrnwsegysdfdrgayvitfipkswntapdkvkqgwp
>1IVH_A
hsllpvddainglseeqrqlrqtmakflqehlapkaqeidrsnefknlrefwkqlgnlgvlgitapvqyggsg
lgylehvlvmeeisrasgavglsygahsnlcinqlvrngneaqkekylpklisgeyigalamsepnagsdvvs
mklkaekkgnhyilngnkfwitngpdadvlivyaktdlaavpasrgitafivekgmpgfstskkldklgmrgs
ntcelifedckipaanilghenkgvyvlmsgldlErlvlaggplglmqavldhtipylhvreafgqkighfql
mqgkmadmytrlmacrqyvynvakacdeghctakdcagvilysaecatqvaldgiqcfggngyindfpmgrfl
rdaklyeigagtsevrrlvigrafnadfh
>1FFT_A
mfgklsldavpfhepivmvtiagiilgglalvglityfgkwtylwkewltsvdhkrlgimYiivaivmllRgf
aDaimMrsqqalasageagflpphhydqiFtahgvimiffvampfviglmNlvvplqigarDvafpflNnlSf
wfTvvgvilvnvslgvgefaqtgwlaypplsgieyspgvgvdywiwslqlsgigTtlTginffvTilkmrapg
mtmfkmpvftwaslcanvliiasfpiltvtvalltldrylgthfftndmggnmmmyinliwawgHpEvYilil
pvfgvfSeiaatfsrkrlfgytSlvwatvcitvlsfivwlhhfftmgaganvnaffgittmiiaipTgvKifn
wlftmyqgrivfhsamlwtigfivtfsvggmtgvllavpgadfvlhnslfliaHFHnviiggvvfgcfagmty
wwpkafgfklnetwgkrafwfwiigffvafmplyalgfmgmtrrlsqqidpqfhtmlmiaasgavlialgilc
lviqmyvsirdrdqnrdltgdpwggrtlewatsspppfynfavvphvherdafwemkekgeaykkpdhyeeih
mpknsgagiviaafstifgfamiwhiwwlaivgfagmiitwivksfdedvdyyvpvaeieklenqhfdeitka
glkngn
>1STC_E
gnaaaakkgseqesvkeflakakedflkkwenpaqntahldqferiktlgtgsfgrvmlvkhmetgnhyamki
ldkqkvvklkqiehtlnekrilqavnfpflvklefsfkdnsnlymvmeyvpggemfshlrrigrfsepharfy
aaqivltfeylhsldliyrDlKpenllidqqgyiqvtdfgfakrvkgrtwtlcgTpeylapeiilskgynkav
dwwalgvliyemaagyppffadqpiqiyekivsgkvrfpshfssdlkdllrnllqvdltkrfgnlkngvndik
nhkwfattdwiaiyqrkveapfipkfkgpgdtsnfddyeeeeirvsinekcgkefsef
>1K4V_B
klklsdwfnpfkrpevvtmtkwkapvvwegtynravldnyyakqkitvgltvfavgryiehyleefltsankh
fmvghpvifyimvddvsrmplielgplrsfkvfkikpekrwqdismmrmktigehivahiqhevdflfcmdvd
```

qvfqdkfgvetlgesvaqlQawwykadpndftyerrkesaayipfgegdfyyHaaifggtptqvlnitqecfk
gilkdkkndieaqWhdEshlnkyfllnkptkilspeycwdyhiglpadiklvkmsWqtkeynvvRnnv
>1BXR_B
miksallvledgtqfhgraigatgsavgevvfntsmtgyqeiltdpsysrqivtltyphignvgtndadeess
qvhaqglvirdlpliasnfrntedlssylkrhnivaiadidtrkltrllrekgaqngciiagdnpdaalalek
arafpglngmdlakevttaeayswtqgswtltgglpqakkedelpfhvvaydfgakrnilrmlvdrgcrltiv
paqtsaedvlkmnpdgiflsngpgdpapcdyaitaiqkfletdipvfgiClghqllalasgaktvkmkfghhg
gnhpvkdveknvvmitaqnhgfavdeatlpanlrvthkslfdgtlqgihrtdkpafsfqgHpeaspgphdaap
lfdhfielieqyrktak
>1YBQ_A
midytaagftllqgahlyapedrgicdvlvangkiiavasnipsdivpnctvvdlsgqilcpgfidqhvhlig
gggeagpttrtpevalsrlteagvtsvvgllgtdsisrhpesllaktralneegisawmltgayhvpsrtitg
svekdvaiidrvigvkcaisdhrsaapdvyhlanmaaesrvggllggkpgvtvfhmgdskkalqpiydllenc
dvpiskllpthvnrnvplfeqalefarkggtiditssidepvapaegiaravqagiplarvtlssNgngsqpf
fddegnlthigvagfetlletvqvlvkdydfsisdalrpltssvagflnltgkgeilpgndadllvmtpelri
eqvyargklmvkdgkacvkgtfeta
>1OYG_A
asmketnqkpyketygishitrhdmlqipeqqknekyqvpefdsstiknissakgldvwDswplqnadgtvan
yhgyhivfalagdpknaddtsiymfyqkvgetsidswknagrvfkdsdkfdandsilkdqtqewsgsatftsd
gkirlfytdfsgkhygkqtlttaqvnvsasdsslningvedyksifdgdgktyqnvqqfidegnyssgdnhtl
rDphyvedkghkylvfeantgtedgyqgeeslfnkayygkstsffrqesqkllqsdkkrtaelangalgmiel
nddytlkkvmkpliasntvtdeiEranvfkmngkwylftdsrgskmtidgitsndiymlgyvsnsltgpykpl
nktglvlkmdldpndvtftyshfavpqakgnnvvitsymtnrgfyadkqstfapsfllnikgkktsvvkdsil
eqgqltvnk
>1DGK_N
miaaqllayyftelkddqvkkidkylyamrlsdetlidimtrfrkemknglsrdfnptatvkmlptfvrsipd
gsekgdfialdlggssfrilrvqvnheknqnvhmesevydtpenivhgsgsqlfdhvaeclgdfmekrkikdk
klpvgftfsfpcqqskideailitwtkrfkasgvegadvvkllnkaikkrgdydanivavvndtvgtmmtcgy
ddqhcevgliigtgtnacymeelrhidlvegdegrmcintewgafgddgsledirtefdraidayslnpgkql
fekmvsgmylgelvrlilvkmakegllfegritpelltrgkfntsdvsaieknkeglhnakeiltrlgvepsd
ddcvsvqhvctivsfrsanlvaatlgailnrlrdnkgtprlrttvgvdgslykthpqysrrfhktlrrlvpds
dvrfllsesgsgkgaamvtavayrlaeqhrqieetlahfhltkdmllevkkrmraemelglrkqthnnavvkm
lpsfvrrtpdgtengdflaldlgganfrvllvkirsgkkrtvemhnkiyaipieimqgtgeelfdhivscisd
fldymgikgprmplgftfsfpcqqtsldagilitwtkgfkatdcvghdvvtllrdaikrreefdldvvavvnD
tvgtmmtcayeeptcevglivgtgsnacymeemknvemvegdqgqmcinmewgafgdngclddirthydrlvd
eyslnagkqryekmisgmylgeivrnilidftkkgflfrgqisetlktrgifetkflsqiesdrlallqvrai
lqqlglnstcddsilvktvcgvvsrraaqlcgagmaavvdkirenrgldrlnvtvgvdgtlyklhphfsrimh
qtvkelspkcnvsfllsedgsgkgaalitavgvrlrteass
>1PJQ_A
mdhlpifcqlrdrdclivgggdvaerkarllleagarltvnaltfipqftvwanegmltlvegpfdetlldsc
wlaiaatdddtvnqrvsdaaesrrifcnvvdapkaasfimpsiidrsplmvavssggtspvlarllreklesl
lpqhlgqvaryagqlrarvkkqfatmgerrrfwekffvndrlaqslanadekavnatterlfsepldhrgevv
lvgagpgdaglltlkglqqiqqadivvyDrlvsddimnlvrrdadrvfvgKragyhcvpqeeinqillreaqk
gkrvvrlkggdpfifgrggeeletlchagipfsvvpgitaasgcsaysgipltfhrdyaqsvrlvtghlktgge
ldwenlaaekqtlvfyMglnqaatiqekliafgmqadmpvalvengtsvkqrvvhgvltqlgelaqqvespal
iivgrvvalrdklnwfsnh
>1UI7_B
aspfrlasageisevqgilrtagllgpekriaylgvldpargagseaedrrfrvfihdvsgarpqevtvsvtn
gtvisaveldtaatgelpvleeefevveqllatderwlkalaarnldvskvrvaplsagvfeyaeergrrilr
glafvqdfpedsawahpvdglvayvdvvskevtrvidtgvfpvpaehgnytdpeltgplrttqkpisitqpeg
psftvtggnhiewekwsldvgfdvregvvlhniafrdgdrlrpiinrasiaemvvpygdpspirswqnyfDtg
eylvgqyanslelgcdclgditylspvisdafgnpreirngicmheedwgilakhsdlwsginytrrnrrmvi
sfftttignydygfywylyldgtiefeakatgvvftsafpeggsdnisqlapglgapfhqaifsarldmaidgf
tnrveeedvvrqtmgpgnergnafsrkrtvltreseavreadartgrtwiisnpesknrlnepvgyklhahnq

ptlladpgssiarraafatkdlwvtryadderyptgdfvnqhsggaglpsyiaqdrdidgqdivvwhtfglth
fprvedwpimpvdtvgfklrpegffdrspvldvpan
>1GPM_A
mtenihkhrilildfgsqytqlvarrvrelgvycelwawdvteaqirdfnpsgiilsgGpestteensprapq
yvfeagvpvfgvCYgmqtmamqlgghveasnerefgyaqvevvndsalvrgiedaltadgkplldvwmshgdk
vtaipsdfitvastescpfaimaneekrfygvqfHpEvthtrqgmrmlerfvrdicqcealwtpakiiddava
rireqvgddkvilglsggvDssvtamllhraigknltcvfvdngllrlneaeqvldmfgdhfglnivhvpaed
rflsalagendpeakrkiigrvfvevfdeealkledvkwlaqgtiypdviesaasatgkahvikshhnvgglp
kemkmglveplkelfkdevrkiglelglpydmlyrhpfpgpglgvrvlgevkkeycdllrradaifieelrka
dlydkvsqaftvflpvrsvgvmgdgrkydwvvslravetidfmtahwahlpydflgrvsnriinevngisrvv
ydisgkppatiewe
>1GS5_A
mmnpliiKlgGvlldseealerlfsalvnyreshqrplvivhggGcvvdelmkglnlpvkkknglrvtpadqi
diitgalagtanktllawakkhqiaavglflgdgdsvkvtqldeelghvglaqpgspklinsllengylpvvs
sigvtdegqlmnvnadqaatalaatlgadlillsdvsgildgkgqriaemtaakaeqlieqgiitdgmivKvn
aaldaartlgrpvdiaswrhaeqlpalfngmpmgtrila
>1BF2_A
ainsmslgasydaqqanitfrvyssqatrivlylysagygvqesatytlspagsgvwavtvpvssikaagitg
avyygyrawgpnwpyasnwgkgsqagfvsdvdangdrfnpnklllldpyaqevsqdplnpsnqngnvfasgasy
rttdsgiyapkgvvlvpstqstgtkptraqkddviyevhvrgfteqdtsipaqyrgtyygaglkasylaslgv
taveflpvqetqndandvvpnsdanqnywgymtenyfspdrryaynkaaggptaefqamvqafhnagikvymd
vvynhtaeggtwtssdpttatiyswrgldnatyyeltsgnqyfydntgiganfntyntvaqnlivdslaywan
tmgvdgfrfDlasvlgnsclngaytasapncpnggynfdaadsnvainrilreftvrpaaggsgldlfaEpwa
iggnsyqlggfpqgwsewnglfrdslrqaqnelgsmtiyvtqdandfsgssnlfqssgrspwnsinfidvhDg
mtlkdvyscngannsqawpygpsdggtstnyswdqgmsagtgaavdqrraartgmafemlsagtplmqggdey
lrtlqcnnnaynldssanwltyswttdqsnfytfaqrliafrkahpalrpsswysgsqltwyqpsgavadsny
wnntsnyaiayaingpslgdsnsiyvayngwsssvtftlpappsgtqwyrvtdtcdwndgastfvapgsetli
ggagttygqcgqsllllisk
>1ECL_A
mgkalvivEspakaktinkylgsdyvvkssvghirdlptsgsaakksadststktakkpkkdergalvnrmgv
dpwhnweahyevlpgkekvvselkqlaekadhiylatDldregeaiawhlreviggddarysrvvfneitkna
irqafnkpgelnidrvnaqqarrfmdrvvgymvspllwkkiarglsagrvqsvavrlvverereikafvpeef
wevdastttpsgealalqvthqndkpfrpvnkeqtqaavsllekarysvleredkpttskpgapfitstlqqa
astrlgfgvkktmmmaqrlyeagyitYmrtdstnlsqdavnmvrgyisdnfgkkylpespnqyaskgnsqeaH
eairpsdvnvmaeslkdmeadaqklyqliwrqfvacqmtpakydsttltvgagdfrlkargrilrfdgwtkvm
palrkgdedrilpavnkgdaltlveltpaqhftkpparfseaslvkelekrgigrpstyasiistiqdrgyvr
venrrfyaekmgeivtdrleenfrelmnydftaqmensldqvanheaewkavldhffsdftqqldkaekdpee
ggmrpnqmvltsi
>1D8D_A
maategvgesapggepgqpeqpppppppppaqqpqeeemaaeageaaaspmddgflsldsptyvlyrdraewa
didpvpqndgpspvvqiiysekfrdvydyfravlqrderserafkltrdaielnaanytvwhfrrvllrslqk
dlqeemnyiiaiieeqpKnyqvwhhrrvlvewlkdpsqelefiadilnqdaknyhawqhrqwviqefrlwdne
lqyvdqllkedvrnnsvwnqrhfvisnttgysdravlerevqytlemiklvphnesawnylkgilqdrglsry
pnllnqlldlqpshsspyliaflvdiyedmlenqcdnkedilnkalelceilakekdtirkeywryigrslqs
khsresdipasv
>1D8D_B
massssftyycppssspvwseplyslrpeharerlqddsvetvtsieqakveekiqevfssykfnhlvprlvl
qrekhfhylkrglrqltdayecldasrpwlcywilhslelldepipqivatdvcqflelcqspdggfgggpgq
yphlaptyaavnalciigteeaynvinrekllqylyslkqpdgsflmhvggevdvrsaycaasvasltniitp
dlfegtaewiarcqnweggiggvpgmeahggytfcglaalvilkkerslnlksllqwvtsrqmrfeggfqgrc
nklvdgcYsfwqagllpllhralhaqgdpalsmshwmfhqqalqeyilmccqcpaggllldkpgksrdfyhtcy
clsglsiaqhfgsgamlhdvvmgvpenvlqpthpvynigpdkviqatthflqkpvpgfeecedavtsdpatd
>1K4T_A

kkpknkdkdkkvpepdnkkkkpkkeeeqkwkwweeerypegikwkflehkgpvfappyeplpenvkfyydgkv
mklspkaeevatffakmldheyttkeifrknffkdwrkemtneekniitnlskcdftqmsqyfkaqtearkqm
skeeklkikeeeneklllkeygfcimdnhkerianfkieppglfrgrgnhpkmgmlkrrimpediiincskdakv
pspppghkwkevrhdnkvtwlvswteniqgsikyimlnpssrikgekdwqkyetarrlkkcvdkirnqyredw
kskemkvrqravalyfidklalRagnekeegetadtvgccslrvehinlhpeldgqeyvvefdflgkdsiryy
nkvpvekrvfknlqlfmenkqpeddlfdrlntgilnkhlqdlmegltakvfRtynasitlqqqlkeltapden
ipakilsynranravailcnHqrappktfeksmmnlqtkidakkeqladarrdlksakadakvmkdaktkkvv
eskkkavqrleeqlmklevqatdreenkqialgtsklnyldpritvawckkwgvpiekiynktqrekfawaid
madedyef
>1UQR_J
mkkilllngpNlnmlgkRephiYgsqtlsdieqhlqqsaqaqgyeldyfqangeeslinrihqafqntdfiii
npgafthtsvairdallavsipfiEvHlsnvhaRepfrhhsylsdvakgvicglgakgydyaldfaiselqki
>1SNZ_B
masvtravfgelpsgggtvekfqlqsdllrvdiiswgctitalevkdrqgrasdvvlgfaelegylqkqpyfg
avigrvanriakgtfkvdgkeyhlainkepnslhggvrgfdkvlwtprvlsngvqfsrispdgeegypgelkv
wvtytldggelivnyraqasqatpvnltnHsyfnlagqaspnindhevtieadtylpvdetliptgevapvqg
tafdlrkpvelgkhlqdfhlngfdhnfclkgskekhfcarvhhaasgrvlevyttqpgvqfytgnfldgtlkg
kngavypkhsgfclEtqnwpdavnqprfppvllrpgeeydhttwfkfsva
>7ATJ_A
qltptfydnscpnvsnivrdtivnelrsdpriaasilRlhfHdcfvngcdasilldnttsfrtekdafgNans
argfpvidrmkaavesacprtvscadlltiaaqqsvtlaggpswrvplgrrdslqafldlananlpapfftlp
qlkdsfrnvglnrssdlvalsgghtfgknqcrfimdrlynfsntglpdptlnttylqtlrglcplngnlsalv
dfdlrtptifdnkyyvnleeqkgliqsdqelfsspnatdtiplvrsfanstqtffnafveamdrmgnitpltg
tqgqirlncrvvnsns
>2NMT_A
amkdhkfwrtqpvkdfdekvveegpidkpktpedisdkplpllssfewcsidvdnkkqledvfvllnenyved
rdagfrfnytkeffnwalkspgwkkdwhigvrvketqklvafisaipvtlgvrgkqvpsveiNFLcvhkqlrs
krltpvlikeitrrvnkcdiwhalytagivlpapvstcrythrplnwkklyevdftglpdghteedmiaenal
paktktaglrklkkedidqvfelfkryqsrfeliqiftkeefehnfigeeslpldkqvifsyvveqpdgkitd
ffsfyslpftilnntkykdlgigylyyyatdadfqfkdrfdpkatkalktrlceliydacilaknanmdvfna
ltsqdntlflddlkfgpgdgflnfylfnyrakpitgglnpdnsndikrrsnvgvvmL
>1GPA_D
rkqivrglagvenvtelkknfnrhlhftlvkdrnvatprdyyfalahtvrdhlvgrwirtqqhyyekdpkriy
ylslefymgrtlqntmvnlalenacdeatyqlgldmeeleeieedaglgngglgrlaacfldsmatlglaayg
ygiryefgifnqkicggwqmeeaddwlrygnpwekarpeftlpvhfygrvehtsqgakwvdtqvvlampydtp
vpgyrnnvvntmrlwsakapndfnlkdfnvggyiqavldrnlaenisrvlypndnffegkelrlkqeyfvvaa
tlqdiirrfksskfgcrdpvrtnfdafpdkvaiqlndthpslaipelmrvlvdlerldwdkawevtvktcayt
nhtvipealerwpvhlletllprhlqiiyeinqrflnrvaaafpgdvdrlrrmslveegavkrinmahlciag
shavngvarihseilkktifkdfyelephkfqnktngitprrwlvlcnpglaeiiaerigeeyisdldqlrkl
lsyvddeafirdvakvkqenklkfaaylereykvhinpnslfdvqvKRiheyKrqllnclhvitlynrikkep
nkfvvprtvmiggkaapgyhmakmiiklitaigdvvnhdpvvgdrlrviflenyrvslaekvipaadlseqis
tagteasgTgnmkfmlngaltigtmdganvemaeeageenffifgmrvedvdrldqrgynaqeyydripelrq
iieqlssgffspkqpdlfkdivnmlmhhdrfkvfadyeeyvkcqervsalyknprewtrmvirniatsgkfss
drtiaqyareiwgvepsrqrlpap
>1WOS_A
mkrtplfekhvelgakmvdfagwemplyytsifeevmavrksvgmfdvshmgeflvkgpeavsfidflitndf
sslpdgkaiysvmcnenggiidDlvvykvspdealmvvnaaniekdfnwikshsknfdvevsnisdttaliaf
qgpkaqetlqelvedgleeiayysfrksivagvetlvsrtgytgedgfelmleaknapkvwdalmnllrkidg
rpaglgardvcrleatyllygqdmdentnpfevglswvvklnkdfvgkeallkakekverklvalelsgkria
rkgyevlkngervgeitsgnfsptlgksialalvsksvkigdqlgvvfpggklvealvvkkpfyrgsvr
>2BKR_A
mdpvvlsymdsllrqsdvslldppsWlndhiigfafeyfansqfhdssdhvsfispevtqfikctsnpaeiam
flepldlpnkrvvflaindnsnqaaggsHWsllvylqdknsffhyDshsrsnsvhakqvaekleaflgrkgdk
lafveekapaqqnsydCgmyvicntealcqnffrqqtesllqlltpayitkkrgewkdliatlakk

```
>1NMW_A
geparvrcsHllvkhsqsrrpsswrqekitrtkeealelingyiqkiksgeedfeslasqfsdCssakargdl
gafsrgqmqkpfedasfalrtgemsgpvftdsgiHiilrte
>1FR2_B
meskRnkpgkatgkgkpvgdkwlddagkdsgapipdriadklrdkefksfddfrkavweevskdpelsknlnp
snkssvskgyspftpknqqvggrkvyElHHdkpisqggevydmdnirvttpkrhidihrgk
>1SOX_A
apsypeytreevgrhrspeervwvthgtdvfdvtdfvelhpggpdkillaaggalepfwalyavhgephvlel
lqqykvgelspdeapaapdaqdpfagdpprhpglrvnsqkpfnaeppaellaerfltpnelfftRnhlpvpav
epssyrlrvdgpgggtlslslaelrsrfpkhevtatlqcagnrrsemsrvrpvkglpwdigaistarwggarl
rdvllhagfpeelqgewhvcfegldadpggapygasipygralspaadvllayemngtelprdhgfpvrvvvp
gvvgarsvkwlrrvavspdespshwqqndYkgfspcvdwdtvdyrtapaiqelpvqsavtqprpgaavppgel
tvkgyawsgggrevvrvdvsldggrtwkvarlmgdkappgrawawalweltvpveagteleivckavdssynv
qpdsvapiwnlrgvlstawhrvrvsvqd
>1XK7_C
shdhlppkfgplaglrvvfsgiEiagpfagqfaewgaeviwienvawadtirvqpnypqlsrrnlhalslnif
kdegreaflklettdifieaskgpafarrgitdevlwqhnpklviahlsgfgqygteeytnlpayNtiaqafs
gyliqngdvdqppafpytaDyfsgltattaalaalhkvretgkgesidiayevlrgqyfdyfnggecprskgk
dpyyagcglykcadgyivelvgitqieecfkdiglahllgtpeipegtqlihriecpygplveekldawlath
tiaevkerfaelniacakvltvpelesnpqyvaresitqwqtdgrtckgpnipkfknnpgqiwrgpshgdtaa
ilknigysendiqelvskglakved
>1MRO_B
akfedkvdlyddrgnlveeqvplealsplrnpaiksivqgikrtvavnlegienalktakvggpackimgrel
dldivgnaesiaaaakemiqvtedddtnvellgggkralvqvpsarfdvaaeysaaplvtatafvqaiinefd
vsmydanmvkaavlgrypqsveymganiatmldipqklegpgyalrnimvnhvvaatlkntlqaaalstileq
tamfemgdavgafermhllglayqgmnadnlvfdlvkangkegtvgsviadlveraledgvikvekeltdykv
ygtddlamwnayaaaglmaatmvnqgaaraaqgvsstllyyndliefetglpsvdfgkvegtavgfsffshsi
Ygggpgifngnhivtrhskgfaipcvaaamaldagtqmfspeatsglikevfsqvdefreplkyvveaaaei
knei
>1B3R_D
lpykvadiglaawgrkaldiaenempglmrmremysaskplkgariagclHmtvetavlietlvalgaevrws
scnifstqdhaaaaiakagipvfawkgetdeeylwcieqtlhfkdgplnmildDggdltnlihtkhpqllsgi
rgiseetttgvhnlykmmangilkvpainvndsvtKskfDNlygCreslidgikratdvmiagkvavvagygd
vgkgcaqalrgfgarviiteidpinalqaamegyevttmdeackegnifvtttgcvdiilgrhfeqmkddaiv
cnigHfdveidvkwlnenavekvnikpqvdryllknghriillaegrlvnlgcamghpsfvmsnsftnqvmaq
ielwthpdkypvgvhflpkkldeavaeahlgklnvkltkltekqaqylgmpingpfkpdhyry
>3GRV_A
qcflidknfvnkavesanltkddvvleiGlgkgilteelaknakkvyviEidkslepyanklkelynnieiiw
gdalkvdlnkldfnkvvaNlpyqisspitfklikrgfdlavlmyqyefakrmvaaagtkdygrlsvavqsrad
veivakvppsafypkpkvysaivkikpnkgkyhienenffddflraifqhrnksvrkalidsskelnynkdem
kkiledflntnseiknlinekvfklsvkdivnlsnefyrflqn
>1L1L_A
mseeislsaefidrvkasvkphwgklgwvtykrtyarwlpekgrsenwdetvkrvvegninldprlqdspsle
lkqslteeaerlykliyglgatpsgrnlwisgtdyqrrtgdslnnCwfvairpqkygdskivpsylgkqekav
smpfsflfdelmkgggvgfsvarsnisqiprvdfaidlqlvvdetsesydasvkvgavgknelvqdadsiyyr
lpdtregwvlanallidlhfaqtnpdrkqklildlsdirpygaeihgfggtasgpmplismlldvnevlnnka
ggrltavdaadicnligkavvagnvrrsaelalgsnddqdfismkqdqeklmhhrwasnnsvavdsafsgyqp
iaagirengepgivnldlsknygrivdgyqagidgdvegtNpCgEislangepCnlfevfpliaeeqgwdlqe
vfalaaryakrvtfspydweisreiiqknrrigismsgiqdwlltrlgnrvvtgfkddfdpetheaikvpvyd
kraikmvdqlykavvkadqdysktlgcnesikhttvkpsgtvaklagasegmhfhygayliqrirfqdsdpll
palkacgyrteadiytenttcvefpikavgadnpnfasagtvsiaeqfatqaflqtywsdnavsctitfqdse
gdqvesllrqyrfitkstsllpyfggslqqapkepidketyekrsqeitgnveevfsqlnsdvkdlelvdqtd
ceggacpik
>1JMS_A
```

nsspspvpgsqnvpapavkkisqyacqrrttlnnynqlftdaldilaendelrenegsclafmrassvlkslp
fpitsmkdtegipclgdkvksiiegiiedgesseakavlnderyksfklftsvfgvglktaekwfrmgfrtls
kiqsdkslrftqmqkagflyyedlvscvnrpeaeavsmlvkeavvtflpdalvtmtggfrrgkmtghdvdfli
tspeatedeeqqllhkvtdfwkqqglllycdilestfekfkqpsrkvdaldhfqkcflilkldhgrvhseksg
qqegkgwkairvDlvmcpydrrafallgwtgsrqferdlrryatherkmmldnhalydrtkrvfleaeseeei
fahlgldyiepwerna
>1W0G_A
ygthshglfkklgipgptplpflgnilsyhkgfcmfdmechkkygkvwgfydgqqpvlaitdpdmiktvlvke
cysvftnrrpfgpvgfmksaisiaedeewkrlrsllsptftsgklkemvpiiaqygdvlvrnlrreaetgkpv
tlkdvfgaysmdvitstsfgvnidslnnpqdpfventkkllrfdfldpfflsitvfpflipilevlnicvfpr
evtnflrksvkrmkesrledflqlmialsdlelvaqsiifiaagyETTssvlsfimyelathpdvqqklqeei
davlpnkapptydtvlqmeyldmvvnetlrlfpiamrlervckkdveingmfipkgvvvmipsyalhrdpkyw
tepekflperfskknkdnidpyiytpFgsgprnCIGmrfalmNmklalirvlqnfsfkpcketqiplklslgg
llqpekpvvlkvesrdg
>1CWY_A
melprafglllhptslpgpygvgvlgreardflrflkeaggrywqvlplgptgygdspyqsfsafagnpylid
lrplaergyvrledpgfpqgrvdygllyawkwpalkeafrgfkekaspeereafaafrereawwledyalfma
lkgahgglpwnrwplplrkreekalreaksalaeevafhaftqwlffrqwgalkaeaealgiriigdmpifva
edsaevwahpewfhldeegrptvvagvppdyfsetgqrwgnplyrwdvleregfsfwirrlekalelfhlvri
DhfrgfeayweipascptavegrwvkapgeklfqkiqevfgevpvlaEdlgvitpevealrdrfglpgmkvlq
fafddgmenpflphnypahgrvvvytgthDndttlgwyrtatphekafmaryladwgitfreeeevpwalmhl
gmksvarlavypvqdvlalgsearmnypgrpsgnwawrllpgelspehgarlramaeaterl
>1A79_B
kitglldgdrvivfdkngisklsarhygnvegnflslslvealylinlgwlevkykdnkplsfeelyeyarnv
eerlclkylvykdlrtrgyivktglkygadfrlYerganidkeHsvylvkvfpedssfllseltgfvrvahsv
rKklliaivdadgdivyynmtyvkp
>2C7V_A
eapaavvtgaakRigraiavklhqtgyrvvihyhnsaeaavsladelnkersntavvqadltnsnvlpascee
iinscfrafgrcdvlvnnasafyptplvqgktvetqvaeligtnaiapflltmsfaqrqkgssnlsivnlcDa
mvdqpmafslYnmgkhalvgltqsaalelapygirvngvapgvsllpvamgeeekdkwrrkvplgrreasaeq
iadaviflvsgsaqyitgsiikvdgglslvha
>1J79_A
tapsqvlkirrpddwhlhlrdgdmlktvvpytseiygraivmpnlappvttveaavayrqrildavpaphdft
plmtcyltdsldpnelergfnegvftaaklypanattnsshgvtsvdaimpvlermekigmpllvhgevthad
idifdrearfiesvmeplrqrltalkvvfehittkdaadyvrdgnerlaatitpqhlmfnrnhmlvggvrphl
yclpilkrnihqqalrelvasgfqrvflgtDsapharhrkesscgcagcfnaptalgsyatvfeemnalqhfe
afcsvngpqfyglpvndtfielvreeqqvaesialtddtlvpflagetvrwsvkq
>1LIJ_A
mavdssnsatgpmrvfaignpildlvaevpssfldefflkrgdatlatpeqmriystldqfnptslpggsaln
svrvvqkllrkpgsagymgaigddprgqvlkelcdkeglatrfmvapgqstgtcavlinekeRtlcthlgacg
sfripenwttfasgalifyataytltatpknalevagyahgipnaiftlnlsapfcvelykdamqslllhtni
lfgneeefahlakvhnlvaaekvalsvankehavevctgalrlltagqntgatklvvmtrghnpviaaeqtad
gtvvvhevgvpvvaaekivdtngagDafvggflyglsqgktvkqcimcgnacaqdviqhvgfslsftslpc
>1Y9M_A
fnydqpyrgqyhfspqknwmnDpngllyhngtyhlffqynpggiewgniswghaisedlthweekpvallarg
fgsdvtemyfsgsavadvnntsgfgkdgktplvamytsyypvaqtlpsgqtvqedqqsqsiaysldggltwtt
ydaanpvipnppspyeaeyqnfrdpfvfwhdesqkwvvvtsiaelhklaiytsdnlkdwklvsefgpynaqgg
vwEcpglvklpldsgnstkwvitsglnpggppgtvgsgtqyfvgefdgttftpdadtvypgnstanwmdwgpd
fyaaagynglslndhvhigwmnnwqyganiptypwrsamaiprhmalktigskatlvqqpqeawssisnkrpi
ysrtfktlsegstnttttgetfkvdlsfsakskastfaialrasanfteqtlvgydfakqqifldrthsgdvs
fdetfasvyhgpltpdstgvvklsifvdrssvevfggqgettltaqifpssdavharlastggttedvradiy
kiastw
>3AN1_A

adelvffvngkkvveknadpettllvylrrklglcgtklgcgeggcgactvmiskydrlqnkivhfsvnacla
picslhhvavttvegigntqklhpvqeriarshgsqcgfctpgivmsmytllrnqpeptveeienafqgnlcr
ctgyrpilqgfrtfakdgspslfnpedfkpldptqepifppellrlkdtpqkklrfegervtwiqastmeell
dlkaqhpdaklvvgnteigiemkfknmlfplivcpawipelnsvvhgpegisfgascplslvesvlaeeiakl
peqktevfrgvmeqlrwfagkqvksvasiggniitaspisdlnpvfmasgakltlvsrgtrrtvrmdhtffpg
yrktllrpeeillsieipyskegeffsafkqasrreadiakvtsgmrvlfkpgtievqelslcfggmadrtis
alkttpkqlskswneellqsvcaglaeelqlapdapggmvefrrtltlsfffkfyltvlqklgradledmcgk
ldptfasatllfqkdppanvqlfqevpkdqseedmvgrplphlaanmqasgeavycddipryenelslrlvts
trahakitsidtseakkvpgfvcfltaedvpnsnatglfndetvfakdevtcvghiigavvadtpehaqraar
gvkityedlpaiitiqdainnnsfygseikiekgdlkkgfseadnvvsgelyiggqehfyletnctiavpkge
agemelfvstqntmktqsfvakmlgvpdnrivvrvkrmgggfggkEtrstvvstalalaahktgrpvrcmldr
dedmlitggrhpflakykvgfmktgtvvalevahfsnggntedlsrsimeRalfhmdnaykipnirgtgrick
tnlpsntafrgfggpqgmliaeywmsevaitcglpaeevrrknmykegdlthfnqklegftlprcwdeciass
qylarkrevekfnrencwkkrglciiptkfgisftlpflnqggalvhvytdgsvllthggtemgqglhtkmvq
vasralkiptskihisetstntvpntsptaasasadlngqgvyeacqtilkrlepfkkkkptgpweawvmday
tsavslsatgfyktpnlgysfetnsgnpfhyfsygvacseveidcltgdhknlrtdivmdvgsslnpaidigq
vegafvqglglftmeelhyspegslhtrgpstykipafgsipiefrvsllrdcpnkraiyaskavgEpplfla
ssiffaikdairaaraqhgdnakqlfqldspatpekirnacvdqfttlcvckswsvri
>1PS9_A
sypslfapldlgfttlknrvlmgsmhtgleeypdgaerlaafyaerarhgvalivsggiapdltgvgmeggam
lndasqiphhrtiteavhqeggkialqilhtgrysyqphlvapsalqapinrfvphelsheeilqlidnfarc
aqlareagydgvevmgsegYlinefltlrtnqrsdqwggdyrnrmrfavevvravrervgndfiiiyrlsmld
lvedggtfaetvelaqaieaagatiintgigwHeariptiatpvprgafswvtrklkghvslplvttnrindp
qvaddilsrgdadmvsmarpfladaellskaqsgradeintcigcnqacldqifvgkvtsclvnprachetkm
pilpavqkknlavvgagpaglafainaaarghqvtlfdahseiggqfniakqipgkeefyetlryyrrmievt
gvtlklnhtvtadqlqafdetilasgivprtppidgidhpkvlsyldvlrdkapvgnkvaiigcggigfdtam
ylsqpgestsqniagfcnewgidsslqqagglspqgmqiprsprqivmlqrkaskpgqglgkttgwihrttll
srgvkmipgvsyqkidddglhvvingetqvlavdnvvicagqepnralaqplidsgktvhliggcdvamelda
rraiaqgtrlalei
>1RHC_A
mktqigyfasleqyrpmdaleqairaekvgfdsvwvddHfhpWyhdnaqsaqawawmgaalqatkkvfistci
tcpimrynpaivaqtfatlrqmypgrvgvavgagEamnevpvtgewpsvpvrqdmtveavkvmrmlwesdkpv
tfkgdyftldkaflytkpddevplyfsgmgpkgaklagmygdhlmtvaaapstlknvtipkfeegareagkdp
skmehamliwysvdpdydkavealrfwagclvpsmfkykvydpkevqlhanlvhcdtikenymcatdaeemik
eierfkeaginhfclgnsspdvnfgidifkevipavrd
>1DKI_D
dqnfarnekeakdsaitfiqksaaikagarsaedikldkvnlggelsgsnmyvynistggfvivsgdkrspei
lgystsgsfdvngkeniasfmesyveqikenkkldstyagtaeikqpvvkslldskgihynqgnpynlltpvi
ekvkpgeqsfvgqhaatgSvatataqimkyhnypnkglkdytytlssnnpyfnhpknlfaaistrqynwnnil
ptysgresnvqkmaiselmadvgisvdmdygpssgsagssrvqralkenfgynqsvhqinrgdfskqdweaqi
dkelsqnqpvyyqgvgkvggHafvidgadgrnfyhvnwgwggvsdgffrldalnpsalgtgggaggfngyqsa
vvgikp
>1USH_A
mkllqrgvalallttftlasetalayeqdktykitvlhtndhhghfwrneygeyglaaqktlvdgirkevaae
ggsvlllsggdintgvpesdlqdaepdfrgmnlvgydamaigNHefDnpltvlrqqekwakfpllsaniyqks
tgerlfkpwalfkrqdlkiavigltddtakignpeyftdiefrkpadeaklviqelqqtekpdiiiaathmg
hydngehgsnapgdvemaralpagslamivgghsqdpvcmaaenkkqvdyvpgtpckpdqqngiwivqahewg
kyvgradfefrngemkmvnyqlipvnlkkkvtwedgkservlytpeiaenqqmisllspfqnkgkaqlevkig
etngrlegdRdkvRfvqtnmgrlilaaqmdrtgadfavmsgggiRdsieagdisyknvlkvqpfgnvvvyadm
tgkevidyltavaqmkpdsgaypqfanvsfvakdgklndlkikgepvdpaktyrmatlnfnatggdgyprldn
kpgyvntgfidaevlkayiqksspldvsvyepkgevswq
>1R44_B

meigftfldeivhgvrwdakyatwdnftgkpvdgyevnrivgtyelaesllkakelaatqgyglllwdgyRpk
ravncfmqwaaqpennltkesyypnidrtemiskgyvasksshsrgsaidltlyrldtgelvpmgsrfdfmde
rshhaangiscneaqnrrrlrsimensgfeayslEwwhyvlrdepypnsyfdfpvk
>1L6P_A
glfdapgrsqfvpadqafafdfqqnqhdlnltwqikdgyylYrkqiritpehakiadvqlpqgvwheDeFYgk
seiyrdrltlpvtinqasagatltvtyqgCadagfCyppetktvplsevvan
>1REQ_A
stlprfdsvdlgnapvpadaarrfeelaakagtgeawetaeqipvgtlfnedvykdmdwldtyagippfvhgp
yatmyafrpwtirqYagfstakesnafyrrnlaagqkglsvafdlpthrgydsdnprvagdvgmagvaidsiy
dmrelfagipldqmsvsmtmngavlpilalyvvtaeeqgvkpeqlagtiqndilkefmvrntyiyppqpsmri
iseifaytsanmpkwnsisisgyHmqeagatadiemaytladgvdyiragesvglnvdqfaprlsffwgigmn
ffmevaklraarmlwaklvhqfgpknpksmslrthsqtsgwsltaqdvynnvvrtcieamaatqghtqslhtn
sldeaialptdfsariarntqlflqqesgttrvidpwsgsayveeltwdlarkawghiqevekvggmakaiek
gipkmrieeaaartqaridsgrqpligvnkyrlehepplddvlkvdnstvlaeqkaklvklraerdpekvkaal
dkitwaagnpddkdpdrnllklcidagramatvgemsdalekvfgrytaqirtisgvyskevkntpeveeare
lveefeqaegrrprillaKmgqDgHdrgqkviataYadlgfdvdvgplfqtpeetarqaveadvhvvgvssla
ggHltlvpalrkeldklgrpdilitvggvipeqdfdelrkdgaveiytpgtvipesaislvkklraslda
>1XGM_A
mdteklmkageiakkvrekaiklarpgmlllelaesiekmimelggkpafpvnlsineiaahytpykgdttvl
kegdylkidvgvhidgfiadtavtvrvgmeedelmeaakealnaaisvaragveikelgkaieneirkrgfkp
ivnlsghkieryklhagisipniyrphdnyvlkegdvfaiEpfatigagqvievpptliymyvrdvpvrvaqa
rfllakikreygtlpfayrwlqndmpegqlklalktlekagaiygypvlkeirngivaqfehtiivekdsviv
tte
>3KBK_A
vppslrlpvieaafprqlhpywpklqettrtwllekrlmpadkveeyadglcytdlmagyylgapdevlqaia
dysawFFvwddrhdrdivhgragawrrlrgllhtaldspgdhlhhedtlvagfadsvrrlyaflpatwnarfa
rhfhtvieaydrefhnrtrgivpgeeylelrrltfahwiwtdllepssgcelpdavrkhpayrraallsqefa
awyNdlcslpkeiatleeaigevrrrveeciteflaverdalrfadeladgtvrgkelsgavranvgnmrnWF
ssvywfhhesgry
>1AST_A
aailgdeylwsggvipytfagvsgadqsailsgmqeleektcirfvprttesdyveiftsgsgcwsyvgrisg
aqqvslqangcvyhgtiihElmhaigfyhehtrmdrdnyvtinyqnvdpsmtsnfdidtysryvgedyqyysi
mhYgkysfsiqwgvletivplqngidltdpydkahmlqtdanqinnlytnecsl
>1PJA_A
mlglwgqrlpaawvllllpflplllllaapaphrasykpvivvhgLfdssysfrhlleyinethpgtvvtvldl
fdgreslrplweqvqgfreavvpimakapqgvhlicySQgglvcrallsvmddhnvdsfislsspqmgqygdt
dylkwlfptsmrsnlyricyspwgqefsicnywhdphhddlylnassflalingerdhpnatvwrknflrvgh
lvliggpdDgvitpwqssffgfydanetvlemeeqlvylrdsfglktllargaivrcpmagisHtawhsnrtl
yetciepwls
>1U5U_B
wknfgfeifgekygqeelekrikdehtpppdspvfgglklklkkekfktlftlgttlkgfrraTHtvgtggig
eitivndpkfpehefftagrtfparlrhanlkypddagadarsfsikfadsdsdgpldivmNtgeanifwnsp
sledfvpveegdaaeeyvyknpyyyynlvealrrapdtfahlyyYsqvtmpfkakdgkvrycryralpgdvdi
keedesgrlteeeqrkiwifsrhenekrpddylrkeyverlqkgpvnyrlqiqiheaspddtatifhagilwd
kethpwfdlakvsiktplspdvlektafnianqpaslglleakspedynsigelrvavytwvqhlrklkigsl
v
>1CBX_A
arstntfnyatyhtldeiydfmdllvaehpqlvsklqigrsyegrpiyvlkfstggsnrpaiwidlgihsrew
itqatgvwfakkftenygqnpsftaildsmdifleivtnpngfafthsenrlwRktrsvtssslcvgvdanrn
wdagfgkagassspcsetyhgkyansevevksivdfvknhgnfkaflsihsysqlllypygyttqsipdktel
nqvaksavaalkslygtsykygsiittiyqasggsidwsynqgikysftfElrdtgrygfllpasqiiptaqe
twlgvltimehtvnn
>1APX_A

```
gksyptvspdyqkaiekakrklrgfiaekkcaplilRlawHsagtfdsktktggpfgtikhqaelahgaNngl
diavrllepikeqfpivsyadfyqlagvvaveitggpevpfhpgredkpepppegrlpdatkgsdhlrdvfgk
amglsdqdivalsgghtigaahkersgfegpwtsnplifdnsyftelltgekdgllqlpsdkalltdsvfrpl
vekyaadedvffadyaeahlklselgfaea
>1FX2_A
nnnrapkeptdpvtliftdiesstalwaahpdlmpdavaahhrmvrsligrykcyevktvgdsfmiaskspfa
avqlaqelqlcflhhdwgtnalddsyrefeeqraegeceytpptahmdpevysrlwnglrvrvgihtglcdir
hdevtkgydyygrtpnmaaRtesvanggqvlmthaaymslsaedrkqidvtalgdvalrgvsdpvkmyqlntv
psrnfaalrldreyfd
>1DCT_A
mnlislfsgaggldlgfqkagfriicaneydksiwktyesnhsaklikgdiskissdefpkcdgiiggppCqs
wseggslrgiddprgklfyeyirilkqkkpifflaEnvkgmmaqrhnkavqefiqefdnagydvhiillnand
ygvaqdrkrvfyigfrkelninylppiphlikptfkdviwdlkdnpipaldknktngnkciypnheyfigsys
tifmsrnrvrqwnepaftvqasgrqcqlhpqapvmlkvsknlnkfvegkehlyrrltvrecarvqgfpddfif
hyeslndgykmignavpvnlayeiaktiksal
>1GCB_A
msssidiskinswnkefqsdlthqlattvlknynaddallnktrlqkqdnrvfntvvstdstpvtnQkssgrC
wlfaatnqlrlnvlselnlkefelsqaylffydklekanyfldqivssadqdidsrlvqyllaaptedggqys
mflnlvkkyglipkdlygdlpysttasrkwnslltt klrefaetlrtalkersaddsiivtlreqmqreifrl
mslfmdippvqpneqftweyvdkdkkihtikstplefaskyakldpstpvslindprhpygklikidrlgnvl
ggdaviylnvdnetlsklvvkrlqnnkavffgshtpkfmdkktgvmdielwnypaigynlpqqkasriryhes
lmtHamlitgchvdetsklplryrveNswgkdsgkdglyvmtqkyfeeycfqivvdinelpkelaskftsgke
epivlpiwdpmgalak
>1BG6_A
miesktyavlglgngghafaaylalkgqsvlawdidaqrikeiqdrgaiiaegpglagtahpdlltsdiglav
kdadvilivvpaihhasiaaniasyisegqliilnpgatggalefrkilrengapevtigetssmlftcrser
pgqvtvnaikgamdfaclpaakagwaleqigsvlpqyvavenvlhtsltnvnavmHplptllnaarcesgtpf
qyylegitpsvgslaekvdaeriaiakafdlnvpsvcewykesygqspatiyeavqgnpayrgiagpinlntr
yffeDvstglvplselgravnvptplidavldlisslidtdfrkegrtleklglsgltaagirsave
>1DL2_A
gagemrdriesmfleswrdyskhgwgydvygpiehtshnmprgnqplgwiivdsvdtlmlmynsstlyksefe
aeiqrsehwindvldfdidaevnvfEttiRmlggllsayhlsdvlevgnktvylnkaidlgdrlalaflstqt
gipyssinlhsgqavknhadggasstaefttlqmefkylayltgnrtywelvervyeplyknndllntydglv
piytfpdtgkfgastirfgsrgDsfyeyllkqyllthetlyydlyrksmegmkkhllaqskpsslwyigereq
glhgqlspkmdhlvcfmggllasgsteglsihearrrpffsksdwdlakgitdtcyqmykqsssglapeivvf
ndgnikqdgwwrssvgdffvkpldrhnlqrpEtvesimfmyhlshdhkyrewgaeiatsffentcvdcndpkl
rrftslsdcitlptkksnnmesfwlaetlkylyilfldefdltkvvfnteahpfpvldeeilksqslttgwsl
>1JOA_A
mkvivlgssHggyeaveellnlhpdaeiqwyekgdfisflscgmqlylegkvkdvnsvrymtgekmesrgvnv
fsnteitaiqpkehqvtvkdlvsgeervenydkliispgavpfeldipgkdldniylmrgrqwaiklkqktvd
pevnnvvvigsgyigieaaeafakagkkvtvidildrplgvyldkeftdvlteemeannitiatgetveryeg
dgrvqkvvtdknaydadlvvvavgvrpntawlkgtlelhpngliktdeymrtsepdvfavgdatlikynpadt
evnialatnaRkqgrfavknleepvkpfpgvqgssglavfdykfastginevmaqklgketkavtvvedylmd
fnpdkqkawfklvydpettqilgaqlmskadltaninaislaiqakmtiedlayadfffqpafdkpwniinta
aleavkqer
>1K3P_A
adtkakltlngdtaveldvlkgtlgqdvidirtlgskgvftfdpgftstasceskitfidgdegillhrgfpi
dqlatdsnylevcyillngekptqeqydefkttvtrhtmiheqitrlfhafrrdshpmavmcgitgalaafyh
dsldvnnprhreiaafrllskmptmaamcykysigqpfvyprndlsyagnflnmmfstpcepyevnpileram
drililhadheqnaStstvrtagssganpfaciaagiaslwgpaHgganeaalkmleeissvkhipeffrrak
dkndsfrlmgfgHrvyknydpratvmretchevlkelgtkddllevamelenialndpyfiekklypnvDfys
giilkamgipssmftvifamartvgwiahwsemhsdgmkiarprqlytgyekrdfksdikr
>1IVD_A
```

veyrnwskpqcqitgfapfskdnsirlsaggdiwvtrepyvscdpvkcyqfalgqgttldnkhsndtvhDrip
hrtllmnelgvpfhlgtrqvciawssssschdgkawlhvcitgddknatasfiydgrlvdsigswsQnilrtqe
secvcingtctvvmtdgsasgradtrilfieegkivhisplagsaqhveEcscyprypgvrcicrdnwkgsnr
pvvdinmedysidssyvcsglvgdtprnddrssnsncrdpnnergtqgvkgwafdngndlwmgrtiskdlRsg
yetfkviggwstpnsksqinrqvivdsdnrsgysgifsvegkscinrcfyvelirgrkqetrvwwtsnsivvf
cgtsgtygtgswpdganinfmpi
>2WK8_B
lpdfiqnkidhyienyfdinkngkhlvlgkqaspddiilqsndylalanhplikarlakslleiekrlakftg
fdecllsqsgwnanvgllqticqpntnvyidffaHmslwegaryanaqahpfmhnncdhlrmliqrhgpgiiv
vDsiystlgtiaplaelvniskefgcallvDesHslgthgpngagllaelgltrevhfmtaslaKtfayraga
iwcnnevnrcvpfisypaifsstllpyeaaglettleiiesadnrrqhldrmarklriglsqlgltirsesqi
igletgderntekvrdylesngvfgsvfcrpatsknkniirlslnsdvndeqiakiievcsdavnygdfyfr
>2HDH_A
kiivkhvtvigggglgagiaqvaaatghtvvlvdqtedilakskkgieeslrkvakkkfaenpkagdefvaktl
stiatstdaasvvhstdlvveaivenlkvknelfkrldkraaehtifasntSslqitsianattrqdrfaglH
ffnpvpvklvEviktptsqktfeslvdfskalgkhpvsckdtpgfivNrllvpyleairlyergdaskedidt
aklgagypgpfelldyvgldttkfivdgwhedaenplhqpspslnklvaenkfgkktgegfykykaa
>1NLU_A
aagtakghnptefptiydassaptaanttvgiitiggvsqtlqdlqqftsanglasvntqtiqtgssngdysd
dqqgqgEwdlDsqsivgsaggavqqllfymadqsasgntgltqafnqavsdnvakvinvslgwceadanadgt
lqaedrifataaaqgqtfsvssgDegvyecnnrgypdgstysvswpasspnviavggttlyttsagaysnetv
wnegldsngklwatgggysvyeskpswqsvvsgtpgrrllpdisfdaaqgtgaliynygqlqqiggtSlaspi
fvglwarlqsansnslgfpaasfysaisstpslvhdvksgnngyggygynagtgwdyptgwgsldiaklsayi
rsngf
>1ITQ_A
dffrdeaerimrdspvidghndlpwqlldmfnnrlqderanlttlagthtnipklragfvggqfwsvytpcdt
qnkdavrrtleqmdvvhrmcrmypetflyvtssagirqafregkvasligvegghsidsslgvlralyqlgmr
yltltHscntpwadnwlvdtgdsepqsqglspfgqrvvkelnrlgvlidlahvsvatmkatlqlsrapvifsh
ssaysvcasrrnvpddvlrlvkqtdslvmvnfynnyisctnkanlsqvadhldhikevagaravgfggDfdgv
prvpegledvskypdliaellrrnwteaevkgaladnllrvfeaveqasnltqapeeepipldqlggscrthy
gyss
>1MLA_A
mtqfafvfpgqgsqtvgmladmaasypiveetfaeasaalgydlwaltqqgpaeelnktwqtqpalltasval
yrvwqqqggkapammaghSlgeysalvcagvidfadavrlvemrgkfmqeavpegtgamaaiiglddasiaka
ceeaaegqvvspvnfnspgqvviaghkeaveragaackaagakralplpvsvpsHcalmkpaadklavelaki
tfnaptvpvvnnvdvkcetngdairdalvrQlynpvqwtksveymaaqgvehlyevgpgkvltgltkrivdtl
tasalnepsamaaalel
>1THF_D
mlakriiaclDvkdgrvvkgsnfenlrdsgdpvelgkfyseigidelvflditasvekrktmlelvekvaeqi
dipftvgggihdfetaselilrgadkvsintaavenpslitqiaqtfgsqavvvaiDakrvdgefmvftysgk
kntgillrdwvvevekrgageilltsidrdgtksgydtemirfvrplttlpiiasggagkmehfleaflagad
aalaasvfhfreidvrelkeylkkhgvnvrlegl
>1CEV_B
mkpisiigvpmdlgqtrrgvdmgpsamryagvierlerlhydiedlgdipigkaerlheqgdsrlrnlkavae
aneklaaavdqvvqrgrfplvlggdhsiaigtlagvakhyerlgviwydahgDvntaetspsgnihgmplaas
lgfghpaltqiggyspkikpehvvligvrsldegekkfirekgikiytmhevdrlgmtrvmeetiaylkertd
gvhlsldldgldpsdapgvgtpvigglt yreshlamemlaeaqiitsaefvEvnpildernktasvavalmgs
lfgeklm
>1AFR_A
mpprevhvqvthsmppqkieifksldnwaeenilvhlkpvekcWqpqdflpdpasdgfdeqvrelrerakeip
ddyfvvlvgdmiteealptyqtmlntldgvrdetgasptswaiwtrawtaeenrHgdllnkylylsgrvdmrq
iektiqyligsgmdprtenspylgfiytsfqeraTfishgntarqakehgdiklaqicgtiaaDekrhetayt
kiveklfeidpdgtvlafadmmrkkismpahlmydgrddnlfdhfsavaqrlgvytakdyadileflvgrwkv
dkltglsaegqkaqdyvcrlpprirrleeraqgrakeaptmpfswifdrqvkl

```
>1FOH_A
tkysesycdvlivgagpaglmaarvlseyvrqkpdlkvriidkrstkvyngqaDglqcrtleslknlgladki
lseandmstialynpdenghirrtdripdtlpgisryhqvvlhqgrierhildsiaeisdtrikverplipek
meidsskaedpeaypvtmtlrymsdhestplqfghktenslfhsnlqtqeeedanyrlpegkeageietvhck
yvigcdgghswvrrtlgfemigeqtdyiwgvldavpasnfpdirsrcaihsaesgsimiipRennlvrfYvql
qaraekggrvdrtkftpevvianakkifhpytfdvqqldwftayhigqrvtekfskdervfiagdachthspk
agqgmntsmmdtynlgwklglvltgrakrdilktyeeerhafaqalidfdhqfsrlfsgrpakdvademgvsm
dvfkeafvkgnefasgtainydenlvtdkksskqelakncvvgtrfksqpvvrhseglwmhfgdrlvtdgrfr
iivfagkatdatqmsrikkfsayldsensvislytpkvsdrnsridvitihschrddiemhdfpapalhpkwq
ydfiyadcdswhhphpksyqawgvdetkgavvvvrpdgytslvtdlegtaeidryfsgilvepkeksgaqtea
dwtksta
>1KP2_A
ttilkhlpvgqrigiafsggldtsaallwmrqkgavpyaytanlgqpdeedydaiprrameygaenarlidcr
kqlvaegiaaiqcgafhnttggltyfnttplgRavtgtmlvaamkedgvniwgdgstykgnDierfyrygllt
naelqiykpwldtdfidelggrhemsefmiacgfdykmsvekaystdsnmlgatheakdleylnssvkivnpi
mgvkfwdesvkipaeevtvrfeqghpvalngktfsddvemmleanriggrhglgmsdqienriieaksrgiye
apgmallhiayerlltgihnedtieqyhahgrqlgrllyqgrwfdsqalmlrdslqrwvasqitgevtlelrr
gndysilntvsenltykperltmekgdsvfspddrigqltmrnlditdtreklfgyaktgllsssaasgvpqv
enlenkgqsvehhhhhh
>1EZ1_B
tllgtalrpaatrvmllgsgelgkevaiecqrlgveviavdryadapamhvahrshvinmldgdalrrvvele
kphyivpeieaiatdmliqleeeglnvvpcaratkltmnregirrlaaeelqlptstyrfadseslfreavad
igypcivkpvmsSsGkgqtfirsaeqlaqawkyaqqggragrvivegvvkfdfeitlltvsavdgvhfcapvg
hrqedgdyreswqpqqmsplaleraqeiarkvvlalggyglfgvelfvcgdevifsevsprphDTgmvtlisq
dlsefalhvraflglpvggirqygpaasavilpqltsqnvtfdnvqnavgadlqirlfgkpeidgsRrlgval
ataesvvdaierakhaagqvkvqg
>1ZOI_B
syvttkdgvqifykdwgprdapvihfhhgWplsaddwdaqllfflahgyrvvahdrrghgrssqvwdghdmdh
yaddvaavvahlgiqgavhvghSTgggevvrymarhpedkvakavliAavpplmvqtpgnpgglpksvfdgfq
aqvasnraqfyrdvpagpfygynrpgveasegiignwwrqgmigsakahydgivafsqtdftedlkgiqqpvl
vmhgddDqivpyensgvlsakllpngalktykgypHgmptthadvinadllafir
>1DCO_A
magkahrlsaeerdqllpnlravgwnelegrdaifkqfhfkdfnrafgfmtrvalqaekldHHpewfnvynkv
hitlstHecaglserDinlasfieqvavsmt
>1D8T_A
skekfertkphvnvgtighvDhgkttltaaittvlaktyggaarafdqidnapeekargitintshveydtpt
rhyahvdcpghadyvknmitgaaqmdgailvvaatdgpmpqtrehillgrqvgvpyiivflnkcdmvddeell
elvemevrellsqydfpgddtpivrgsalkalegdaeweakilelagfldsyipeperaidkpfllpiedvfs
isgrgtvvtgrvergiikvgeeveivgiketqkstctgvemfrklldegragenvgvllrgikreeiergqvl
akpgtikphtkfesevyilskdeggrhtpffkgyrpqfyfrttdvtgtielpegvemvmpgdnikmvvtlihp
iamddglrfaireggrtvgagvvakvlg
>1MLW_A
svpwfpkkisdldhcanrvlmygseldadhpgfkdnvyrkrrkyfadlamnykhgdpipkvefteeeiktwgt
vfrelnklypthacreylknlpllskycgyrednipqledvsnflkertgfsirpvagylsprdflsglafrv
fhctqyvrhssdpfytpepdtcHellghvpllaepsfaqfsqeiglaslgaseeavqklatcyfftvefglck
qdgqlrvfgagllSsiselkhalsghakvkpfdpkitckqeclittfqdvyfvsesfedakekmreftkti
>1QD1_A
sqlvecvpnfsegknqevidaisravaqtpgcvlldvdsgpstnrtvytfvgrpedvvegalnaaraayqlid
msrhhgeHprmgaldvcpfipvrgvtmdecvrcaqafgqrlaeelgvpvylygeaartagrqslpalrageye
alpeklkqaewapdfgpsafvpswgatvagarkfllafninllstreqahrialdlreqggrlkkvqaigwyl
deknlaqvstnlldfevtglhtvfeetcreaqelslpvvgsqlvglvplkalldaaafycekenlfllqdehr
irlvvnrlgldslapfkpkeriieylv
>1CDG_A
```

apdtsvsnkqnfstdviyqiftdrfsdgnpannptgaafdgtctnlrlycggdwqgiinkindgyltgmgvta
iwisqpveniysiinysgvnntayhgywardfkktnpaygtiadfqnliaaahaknikviidfapnhtspass
dqpsfaengrlydngtllggytndtqnlfhhnggtdfsttengiyknlydladlnhnnstvdvylkdaikmwl
dlgidgiRmDavkhmpfgwqksfmaavnnykpvftfgEwflgvnevspenhkfanesgmslldfrfaqkvrqv
frdntdnmyglkamlegsaadyaqvddqvtfidnHDmerfhasnanrrkleqalaftltsrgvpaiyygteqy
msggtdpdnraripsfststtayqviqklaplrkcnpaiaygstqerwinndvliyerkfgsnvavvavnrnl
napasisglvtslpqgsyndvlggllngntlsvgsggaasnftlaaggtavwqytaatatptighvgpmmakp
gvtitidgrgfgsskgtvyfgttavsgaditswedtqikvkipavaggnynikvanaagtasnvydnfevlsg
dqvsvrfvvnnattalgqnvyltgsvselgnwdpakaigpmynqvvyqypnwyydvsvpagktiefkflkkqg
stvtweggsnhtftapssgtatinvnwqp
>1HY3_A
mnseldyyekfeevhgilmykdfvkywdnveafqarpddlviatypKsgttwvseivymiykegdvekckedv
ifnripflecrkenlmngvkqldemnsprivktHlppellpasfwekdckiiylcrnakdvavSfyyfflmva
ghpnpgsfpefvekfmqgqvpygswykhvkswwekgksprvlflfyedlkedirkeviklihflerkpseelv
driihhtsfqemknnpstnyttlpdeimnqklspfmrkgitgdwknhftealnekfdkhyeqqmkestlkfrt
ei
>1NDO_A
mnynnkilvsesglsqkhlihgdeelfqhelktifarnwlflthdslipapgdyvtakmgidevivsrqndgs
iraflnvcrhrgktlvsveagnakgfvcsyHgwgfgsngelqsvpfekdlygeslnkkclglkevarvesfhg
fiygcfdqeapplmdylgdaawylepmfkhsgglelvgppgkvvikanwkapaenfvgDayHvgwthasslrs
gesifsslagnaalppegaglqmtskygsgmgvlwdgysgvhsadlvpelmafggakqerlnkeigdvrariy
rshlnctvfpnnsmltcsgvfkvwnpidanttevwtyaivekdmpedlkrrladsvqrtfgpagfwesddndn
metasqngkkyqsrdsdllsnlgfgedvygdavypgvvgksaigetsyrgfyrayqahvsssnwaefehasst
whteltkttdr
>1BE1_A
mekktivlgvigsDcHavgnkildhsftnagfnvvnigvlssqedfinaaietkadlicvsslygqgeidckg
lrekcdeaglkgiklfvggnivvgkqnwpdveqrfkamgfdrvyppgtspettiadmkevlgve
>1F07_B
mkfgiefvpnepiekivklvklaedvgfeyawitdHynnknvyetlaliaegtetiklgpgvtnpyvrspait
asaiatldelsngratlgigpgDkatfdalgiewvkpvstirdaiammrtllagektesgaqlmgvkavqeki
piymgaqgpmmlktageisdgalinasnpkdfeaavplikegaeaagksiadidvaaytccsidedaaaaana
akivvafiaagspppvferhglpadtgkkfgellgkgdfggaigavddalmeafsvvgtpdefipkiealgem
gvtqyvagspigpdkeksikllgeviasf
>1BRM_A
mknvgfigwrgmvgsvlmqrmveerdfdairpvffstsqlgqaapsfggttgtlqdafdlealkaldiivtcq
ggdytneiypklresgwqgywidaasslrmkddaiiildpvnqdvitdglnngirtfvggnCtvslmlmslgg
lfandlvdwvsvatyQaasgggarhmrelltqmghlyghvadelatpssaildierkvttltrsgelpvdnfg
vplagslipwidkqldngqsreewkgqaetnkilntssvipvdglcvrvgalrcHsqaftiklkkdvsiptve
ellaahnpwakvvpndreitmreltpaavtgtlttpvgrlrklnmgpeflsaftvgdqllwgaaeplrrmlrq
la
>1BWL_A
sfvkdfkpqalgdtnlfkpikignnellhravipplTrmralhpgnipnrdwaveyytqraqrpgtmiitega
fispqaggydnapgvwseeqmvewtkifnaihekksfvwvqlwvlgwaafpdnlardglrydsasdnvfmdae
qeakakkannpqhsltkdeikqyikeyvqaaknsiaagadgveiNsaHgYllnqfldphsntrtdeyggsien
rarftlevvdalveaighekvglrlspygvfnsmsggaetgivaqyayvagelekrakagkrlafvhlveprv
tnpfltegegeyeggsndfvysiwkgpviragnfalhpevvreevkdkrtligygrffisnpdlvdrlekglp
lnkydrdtfyqmsahgyidyptyeealklgwdks
>2GGE_A
lvkivrietfplfhrlekpygdangfkryrtcyliriitesgidgwgecvdwlpalhvgftkriipfllgkqa
gsrlslvrtiqkwhqraasavsmalteiaakaadcsvcelwggryreeipvyasfqsysdspqwisrsvsnve
aqlkkgfeqiKvKiggtsfkedvrhinalqhtagssitmildaNqsydaaaafkweryfsewtnigwleeplp
fdqpqdyamlrsrlsvpvaggenmkgpaqyvpllsqrcldiiqpDvmhvngidefrdclqlaryfgvRasaHa
yDgslsrlyalfaqaclpPwsKMkndhiepiewdvmenpftdlvslqpskgmvhipkgkgigteinmeivnry
kwdgsaye

```
>1ADN_A
mkkatcltddqrwqsvlardpnadgefvfavrttgifcrpscrarhalrenvsfyanasealaagfrpCkrcq
pdkanprqhrldkithacr
>1THT_A
mnnqcktiahvlrvnngqelhvwetppkenvpfknntiliasgfarrmdhfaglaeylstngfhvfrydslhh
vglssgsideftmttgknslctvyhwlqtkgtqnigliaaSlsarvayevisdlelsflitavgvvnlrdtle
kalgfdylslpidelpndldfeghklgsevfvrdcfehhwdtldstldkvantsvpliaftannddwvkqeev
ydmlahirtghcklysllgssHdlgenlvvlrnfyqsvtkaaiamdggsleidvdfiepdfeqltiatvnerr
lkaeienrtpema
>1N2C_G
amrqcaiygKggigKstttqnlvaalaemgkkvmivgcdpKadstrlilhskaqntimemaaeagtvedlele
dvlkagyggvkcvesggpepgvgcagrgvitainfleeegayeddldfvfydvlgDvvcggfampirenkaqe
iyivcsgemmamyaanniskgivkyansgsvrlgglicnsrntdredeliialanklgtqmihfvprdnvvqr
aeirrmtvieydpkakqadeyralarkvvdnkllvipnpitmdeleellmefgim
>1N2C_D
sqqvdkikasyplfldqdykdmlakkrdgfeekypqdkidevfqwtttkeyqelnfqrealtvnpakacqplg
avlcalgfektmpyvhgsqgcvayfrsyfnrhfrepvscvsdsmtedaavfggqqnmkdglqnckatykpdmi
avsttCmaeVigddlnafinnskkegfipdefpvpfahtpsfvgshvtgwdnmfegiaryftlksmddkvvgs
nkkinivpgfetylgnfrvikrmlsemgvgysllsdpeevldtpadgqfrmyaggttqeemkdapnalntvll
qpwhlektkkfvegtwkhevpklnipmgldwtdeflmkvseisgqpipasltkergrlvdmmtdshtwlhgkr
falwgdpdfvmglvkfllelgcepvhilchngnkrwkkavdailaaspygknatvyigkdlwhlrslvftdkp
dfmignsygkfiqrdtlhkgkefevplirigfpifdrhhlhrsttlgyegamqilttlvnsilerldeetrgm
qatdynhdlvr
>1FCQ_A
tpdnnktvrefnvywnvptfmchkyglrfeevsekygilqnwmdkfrgeeiailydpgmfpallkdpngnvva
rnggvpqlgnltkhlqvfrdhlinqipdksfpgvgviDfEswrpifrqnwaslqpykklsvevvrrehpfwdd
qrveqeakrrfekygqlfmeetlkaakrmrpaanwgyYaypycynltpnqpsaqceattmqendkmswlfese
dvllpsvYlrwnltsgervglvggrvkealriarqmttsrkkvlpyywykyqdrrdtdlsradleatlrkitd
lgadgfiiWgssddintkakclqfreylnnelgpavkrialnnnandrltvdvsvdqv
>2DCY_E
astdywqnwtdgggivnavngsggnysvnwsntgnfvvgkgwttgspfrtinynagvwapngngyltlygwtr
spliEyyvvdswgtyrptgtykgtvksdggtydiytttrynapsidgdrttftqywsvrqskrptgsnatitf
snhvnawkshgmnlgsnwayqvmatEgyqssgssnvtvw
>1GQG_A
dtsslivedapdhvrpyvirhysharavtvdtqlyrfyvtgpssgyaftlmgtnaphsdalgvlphihqkhyE
nfycnkgsfqlwaqsgnetqqtrvlssgdygsvprnvthtfqiqdpdtemtgvivpggfedlfyylgtnatdt
thtpyipsssdssttgpdsstistlqsfdvyaelsftprtdtvngtapantvwhtganalastagdpyfian
gwgpkylnsqygyqivapfvtatqaqdtnytlstismsttpstvtvptwsfpgacafqvqegrvvvqigdyaa
telgsgdvafipggvefkyyseayfskvlfvssgsdgldqnlvnggeewssvsfpadw
>1RPT_A
kelkfvtlvfRHgdRgpietfpndpikesswpqgfgqltkwgmgqhyelgsyirrrygrflnnsykhdqvyir
stdvdRtlmsamtnlaalfppegnsiwnprllwqpipvhtvslsedrllylpfrdcprfqelksetlkseefl
krlqpyksfidtlpslsgfedqdlfeiwsrlydplycesvhnftlptwatedamtklkelselsllslygihk
qkeksrlqggvlvneilknmklatqpqkarklimysaHDttvsglqmaldvyngllppyaschimelyqdngg
hfvemyyrnetqnepypltlpgcthscplekfaelldpvipqdwatecmg
>4DDV_B
avnskyhhscincgglntdernerglpcevclpedspsdiyrallerktlkeyrfyhefwneyedfrsffkkk
fgkdltgyqrlwakrivqgksftmvaptgvgkttfgmmtalwlarkgkksalvfptvtlvkqtlerlqklade
kvkifgfyssmkkeekekfeksfeeddyhilvfstqfvsknreklsqkrfdfvfvddvdavlkasrnidtllm
mvgipeeiirkafstikqgkiyerpknlkpgilvvssatakprgirpllfrdllnftvgrlvsvarnithvri
ssrskeklvelleifrdgilifaqteeegkelyeylkrfkfnvgetwsefeknfedfkvgkiniligvqayyg
kltrgvdlperikyvifwgtpsmrfsleldkaprfvlarvlkemglikaqenpdveelrkiakehltqkefve
kvkemfrgvvvkdedleliipdvytyiqasgrssrilngvlvkgvsvifeedeeifeslktrllliaeeeiie
eaeanwkelvheveesrrrsereltdtsrslliivEsptkaetlsrflgrassrkerniivheavtgegvilf
```

tatrghvydlvtkggihgveeengkfvpvynslkrcrdcgyqftedrdecpvcsskniddktetlralreisl
eadeilvatdpdvegekiswdvtqyllpstrslrriemheitrygfkkaresvrfvdfnlvkaqivrrvqdrw
igfelsgklqkrfgrsnlsagrvqstvlgwivereeeykksekdftllvlengvnlevegkiaddvvtvvelq
eaeeeknplppyttssalseisqklrlgvqevmdilqdlfekgfitYhRtdstrislegqnvartylrkigke
difmgrswstegaheairpvkpidareleemieegliadltkkhlrvyelifnrflasqsaavkvkkqivtvd
vdgkrmgieqiveilrdgwnlfvpltvsprfehrtykikekkfykkhtvplftqasiveemkkrgigrpstya
kivevlfrrgyvyedkykrvrptrfgvmvysylkeryekyvteettrrleeimdkvergeedyqatlrllyee
ikslmee
>1FR8_B
gsnltsapvpstttrsltacpeespllvgpmliefnipvdlklveqqnpkvklggrytpmdcisphkvaiiip
frnrqehlkywlyylhpilqrqqldygiyvinqagesmfnrakllnvgfkealkdydyncfvfsdvdlipmnd
hntyrcfsqprhisvamdkfgfslpyvqyfggvsalskqqflsingfpnnywgwggEdDdiynrlafrgmsvs
rpnavigkcrmirhsrdkknepnpqRfdriahtketmlsdglnsltymvlevqryplytkitvdigtps
>1F48_A
mqflqnippylfftgkgGvgKtsiscatairlaeqgkrvllvstdpasnvgqvfsqtigntiqaiasvpglsa
leidpqaaaqqyrarivdpikgvlpddvvssineqlsgactteiaafdeftglltdaslltrfdhiifdtapt
ghtirllqlpgawssfidsnpegasclgpmaglekqreqyayavealsdpkrtrlvlvarlqkstlqevarth
lelaaiglknqylvingvlpkteaandtlaaaiwereqealanlpadlaglptdtlflqpvnmvgvsalsrll
stqpvaspssdeylqqrpdipslsalvddiarnehglimlmgkggvgkttmaaaiavrladmgfdvhlttsdp
aahlsmtlngslnnlqvsridpheeteryrqhvletkgkeldeagkrlleedlrspcteeiavfqafsrvire
agkrfvvmdtaptghtlllldatgayhreiakkmgekghfttpmmllqdpertkvllvtlpettpvleaanlq
adleragihpwgwiinnslsiadtrspllrmraqqelpqiesvkrqhasrvalvpvlaseptgidklkqlagh
hhhhh
>1A4I_A
mapaeilngkeisaqirarlknqvtqlkeqvpgftprlailqvgnrddsnlyinvKlkaaeeigikathiklp
rtttesevmkyitslnedstvhgflvqlpldsensinteevinaiapekdvdgltsinagrlargdlndcfip
ctpkgcleliketgvpiagrhavvvgrskivgapmhdlllwnnatvttchsktahldeevnkgdilvvatgqp
emvkgewikpgaividcginyvpddkkpngrkvvgdvaydeakerasfitpvpggvgpmtvamlmqstvesak
rflekfkpg
>1K82_A
PElpevetsrrgiephlvgatilhavvrngrlrwpvseeiyrlsdqpvlsvqrraKylllelpegwiiihlgm
sgslrilpeelppekhdhvdlvmsngkvlrytdprrfgawlwtkeleghnvlthlgpeplsddfngeylhqkc
akkktaikpwlmdnklvvgvgniyaseslfaagihpdrlasslslaecellarvikavllrsieqggttlkdf
lqsdgkpgyfaqelqvygrkgepcrvcgtpivatkhaqRatfycrqcqk
>1F7U_A
mastanmisqlkklsiaepavakdshpdvnivdlmrnyisqelskisgvdsslifpalewtntmergdllipi
prlrikganpkdlavqwaekfpcgdflekveangpfiqfffnpqflaklvipdiltrkedygscklvenkkvi
iefsspniaKpfHagHlrstiiggflanlyeklgwevirmnylgdwgkqfgllavgferygneealvkdpihh
lfdvyvrinkdieeegdsipleqstngkareyfkrmedgdeealkiwkrfrefsiekyidtyarlnikydvys
gesqvskesmlkaidlfkekglthedkgavlidltkfnkklgkaivqksdgttlyltrdvgaamdryekyhfd
kmiyviasqqdlhaaqffeilkqmgfewakdlqhvnfgmvqgmstrkgtvvfldnileetkekmhevmkknen
kyaqiehpeevadlvgisavmiqdmqgkrinnyefkwermlsfegdtgpylqyahsrlrsvernasgitqekw
inadfsllkepaakllirllgqypdvlrnaikthepttvvtylfklthqvsscydvlwvagqteelatarlal
ygaarqvlyngmrllgltpverm
>1TDE_A
gttkhskllilgsgpagytaavyaaranlqpvlitgmekggqlttttevenwpgdpndltgpllmermhehat
kfeteiifdhinkvdlqnrpfrlngdngeytcdaliiatgasarylglpseeafkgrgvsaCatCDgffyrnq
kvaviggntaveealylsniasevhlihrrdgfraekilikrlmdkvengniilhtnrtleevtgdqmgvtg
vrlrdtqnsdniesldvaglfvaighspntaifegqlelengyikvqsgihgnatqtsipgvfaagdvmdhiy
rqaitsagtgcmaaldaeryldgl
>1PKU_C
rmeqsfimiKpdgvqrgligdiisrfekkgfylrgmkfmnversfaqqhYadlsdkpffpglveyiisgpvva
mvwegkdvvatgrriigatrpweaapgtiradyavevgrNviHgsdsvdngkkEialwfpeglaewrsnlhpw
iye

>1DL5_A
mreklfwilkkygvsdhiakafleipreefltksyplsyvyedivlvsyddgeeystsSqpslmalfmewvgl
dkgmrvleigggtgynaavmsrvvgekglvvsveysrkiceiakrnverlgienvifvcgdgyygvpefspyd
vifvtvgvdevpetwftqlkeggrvivpinlklsrrqpaflfkkkdpylvgnykletrfitaggnlgnllern
rkllrefpfnreillvrshifvelvdlltrrlteidgtfyyagpngvvefld drmriygdapeienlltqwes
cgyrsfeylmlhvgynafshiscsi
>1I19_B
stgpvaplptppnfpndialfqqayqnwskeimldatwvcspktpqdvvrlanwahehdykirprgamhgwtp
ltvekganvekviladtmthlngitvntggpvatvtagagasieaivtelqkhdlgwanlpapgvlsiggala
vnahgaalpavgqttlpghtygslsnlvteltavvwngttyaletyqrndpritplltnlgrcfltsvtmqag
pnfrqrcqsytdipwrelfapkgadgrtfekfvaesggaEaiwypftekpwmkvwtvsptkpdssnevgslgs
agslvgkppqarevsgpynyifsdnlpepitdmigainagnpgiaplfgpamyeitklglaatnandiwgwsk
dvqfyikattlrltegggavvtsraniatvindftewfheriefyrakgefplngpvEiRccgldqaadvkvp
svgpptisatrprpdhpdwdvaiwlnvlgvpgtpgmfefyremeqwmrshynnddatfrpewskgwafgpdpy
tdndivtnkmratyiegvpttenwdtararynqidphrvftngfmdkllp
>2HGS_A
matnwgsllqdkqqleelarqavdralaegvllrtsqeptssevvsyapftlfpslvpsalleqayavqmdfn
llvdavsqnaafleqtlsstikqddftarlfdihkqvlkegiaqtvflglnRsdymfqrsadgspalkqiein
tisaSfgglasrtpavhrhvlsvlsktkeagkilsnnpskglalgiakawelygspnalvlliaqekernifd
qraienellarnihvirrtfedisekgsldqdrrlfvdgqeiavvyfrdgymprqyslqnwearlllershaa
kcpdiatqlagtkkvqqelsrpgmlemllpgqpeavarlratfaglysldvgeegdqaiaealaapsrfvlkp
qreGggnnlygeemvqalkqlkdseerasyilmekiepepfencllrpgsparvvqciselgifgvyvrqekt
lvmnkhvghllRtkaiehadggvaagvavldnpypv
>1YON_A
mkitvlgcgalgqlwltalckqghevqgwlrvpqpycsvnlvetdgsifnesltandpdflatsdlllvtlka
wqvsdavkslastlpvttpillihngmgtieelqniqqpllmgttthaarrdgnviihvangithigparqqd
gdysyladilqtvlpdvawhnniraelwrKlavncvinpltaiwncpngelrhhpqeimqiceevaaviereg
hhtsaedlrdyvmqvidataenissmlqdiralrhteidyingfllrrarahgiavpentrlfemvkrkesey
erigtglprpw
>1JOF_E
plhhligtwtppgaiftvqfddekltcklikrteipqdepiswtfdherkniygaakkwssfavkspteivhe
ashpigghprandadtntraifllaakqppyavyanpfykfagygnvfsvsetgkleknvqnyeyqentgiHg
vfdptetylysadltanklwthrklasgevelvgsvdapdpgdhpRwvahptgnylyalEagnriceyvidpa
thpvythhsfplippgipdrdpetgkglyradvcaltfsgkyfassRankfelqgyiagfklrdcgsiekqlf
lsptptsgghsnavspcpwsdewaitddqegwleiyrwkdeflhrvarvripepgfgnaiwyd
>1H54_A
mkrifevqpwnvithtfdpkdkrlqesmtslgngymgmrgdfeegysgdslqgiylggvwypdktrvgwwkng
ypkyfgkvvnavnfiklpieingepvdlakdkisdftldldmhqgvlnrsfvvergavrvalnfqrflsvaqp
elsvqkvtvknlsdaevdvtlkpsidadvmneeanyderfwdvlatdqqadrgsivakttpnpfgtprftsgm
emrlvtdlknvaitqpnekevttaytgklapqasaelekrvivvtsrdydtqesltaamhqlsdkvaqssyed
llnahtaiwaqrweksdvvikgddesqqgirfnlfqlfstyygedarlnigpkgftgekyggatywdteafaf
pvylgitdpkvtrnllmyrykqldgayinaqeqglkgalfpmvtfdgiechneweitfeeihrngdiafaiyn
ytrytgddsyvlhegakvlteisrfwadrvhfskrnnqymihgvtgadEyennvdnnwdtnmlaqwtlkytle
ilgkvdqdtakqldvsdeektkwqdivdrmylpydkdlnifvqhdgfldkdiepvssipadqrpinqnwswdk
ilrspyikqgdvlqgiwdfiddytpeqkkanfdfyepltvhesslspaihsvlaadlhyedkavelysrtarl
dldnynndttdglhitsmtgawiavvqgfagmrvrdgqlhyapflpktwtsytfrqvfrdrlievsvhadgph
fkllsgepltidvagaaaaaaaaa
>1CNS_A
svssivsraqfdrmllhrndgacqakgfytydafvaaaaafsgfgttgsadvqkrevaaflaqtshEttggwa
tapdgafawgycfkqErgassdyctpsaqwpcapgkryygrgpiqlShnynygpagraigvdllanpdlvatd
atvsfktamwfwmtaqppkpsshavivgqwspsgadraagrvpgfgvitniinggiecghgqdsrvadrigfy
krycdilgvgygnnldcysqrpfa
>1NVM_A

mtfnpskklyisdvtlrdgsHairhqytlddvraiaraldkakvdsievahgdglqgssfnygfgrhtdleyi
eavageishaqiatlllpgigsvhdlknayqagarvvrvathcteadvskqhieyarnlgmdtvgflmmshmi
paeklaeqgklmesygatciymadsggamsmndirdrmrafkavlkpetqvgmhahhnlslgvansivaveeg
cdrvdaslagmgagagnaplevfiavaerlgwnhgtdlytlmdaaddivrplqdrpvrvdretlglgyagvYs
sflrhaeiaaakynlktldilvelghrrmvggqedmivdvaldllaahkenra
>1KFU_L
agiaaklakdreaaeglgsheraikylnqdyealrnecleagtlfqdpsfpaipsalgfkelgpyssktrgmr
wkrpteicadpqfiiggatrtdicQgalgdCwllaaiasltlneeilarvvplnqsfqenyagifhfqfwqyg
ewvevvvddrlptkdgellfvhsaegsefwsallekayakingcyealsggattegfedftggiaewyelkkp
ppnlfkiiqkalqkgsllgcsiditsaadseaitfqklvkgHaysvtgaeevesngslqklirirNpWgevew
tgrwndncpswntidpeererltrrhedgefwmsfsdflrhysrleicnltpdtltsdtykkwkltkmdgnwr
rgstaggcrnypntfwmnpqylikleeededeedgesgctflvgliqkhrrrqrkmgedmhtigfgiyevpee
lsgqtnihlsknffltnrarersdtfinlrevlnrfklppgeyilvpstfepnkdgdfcirvfsekkadyqav
ddeieanleefdiseddiddgvrrlfaqlagedaeisafelqtilrrvlakrqdiksdgfsietckimvdmld
sdgsgklglkefyilwtkiqkyqkiyreidvdrsgtmnsyemrkaleeagfkmpcqlhqvivarfaddqliid
fdnfvrclvrletlfkifkqldpentgtieldliswlcfsvl
>1BS0_A
mswqekinaaldarraadalrrrypvaqgagrwlvaddrqylnfssndylglshhpqiirawqqgaeqfgigs
ggsghvsgysvvhqaleeelaewlgysrallfisgfaanqaviaammakedriaadrlsHaslleaaslspsq
lrrfahndvthlarllaspcpgqqmvvtEgvfsmdgdsaplaeiqqvtqqhngwlmvDdahgtgvigeqgrgs
cwlqkvkpellvvtfgKgfgvsgaavlcsstvadyllqfarhliystsmppaqaqalraslavirsdegdarr
eklaalitrfragvqdlpftladscsaiqplivgdnsralqlaeklrqqgcwvtairpptvpagtarlrltlt
aahemqdidrllevlhgng
>3UON_A
tfevvfivlvagslslvtiignilvmvsikvnrhlqtvnnyflfslacadliigvfsmnlytlytvigywplg
pvvcdlwlaldyvvsnasvmnlliisfdryfcvtkpltypvkrttkmagmmiaaawvlsfilwapailfwqfi
vgvrtvedgecyiqffsnaavtfgtaiaafylpviimtvlywhisrasksrinifemlridEglrlkiykDte
gyytigighlltkspslnaakseldkaigrntngvitkdeaeklfnqdvdaavrgilrnaklkpvydsldavr
raalinmvfqmgetgvagftnslrmlqqkrwdeaavnlaksrwynqtpnrakrvittfrtgtwdaypppsrek
kvtrtilaillafiitwapynvmvlintfcapcipntvwtigywlcyinstinpacyalcnatfkktfkhllm
>1J09_A
mvvtriapsptgdphvgtayialfnyawarrnggrfivriedtdraryvpgaeerilaalkwlglsydegpdv
ggphgpyrqserlplyqkyaeellkrgwayrafetpeeleqirkekggydgrarnippeeaeerarrgephvi
rlkvprpgttevkdelrgvvvydnqeipdvvllksdgyptyhlanvvddhlmgvtdviraeewlvstpihvll
yrafgweaprfyhmpllrnpdktkisKrkshtsldwykaegflpealrnylclmgfsmpdgreiftleefiqa
ftwervslggpvfdleklrwmngkyirevlsleevaervkpflreaglsweseaylrravelmrprfdtlkef
pekarylftedypvsekaqrkleeglpllkelyprlraqeewteaaleallrgfaaekgvklgqvaqplraal
tgsletpglfeilallgkeralrrlerala
>1QHF_A
pklvlvrHgqsewneknlftgwvdvklsakgqqeaaragellkekkvypdvlytsklsRaiqtanialekadr
lwipvnrswrlnErhygdlqgkdkaetlkkfgeekfntyrrsfdvppppidasspfsqkgderykyvdpnvlp
eteslalvidrllpywqdviakdllsgktvmiaaHgnslrglvkhlegisdadiaklniptgiplvfeldenl
kpskpsyyldpeaaaagaaav
>1HPM_A
mskgpavgidlgttyscvgvfqhgkveiiandqgnrttpsyvaftdterligdaaknqvamnptntvfdaKrl
igrrfddavvqsdmkhwpfmvvndagrpkvqveykgetksfypeevssmvltkmkeiaeaylgktvtnavvtv
payfndsqrqatkdagtiaglnvlriineptaaaiaygldkkvgaernvlifdlgggtfdvsiltiedgifev
kstagdthlggedfdnrmvnhfiaefkrkhkkdisenkravrrlrtacerakrtlssstqasieidslyegid
fytsitrarfeelnadlfrgtldpvekalrdakldksqihdivlvggstripkiqkllqdffngkelnksinp
deavaygaavqaailsgdkse
>1MPY_A
mnkgvmrpghvqlrvldmskalehyvellgliemdrddqgrvylkawtevdkfslvlreadepgmdfmgfkvv
dedalrqlerdlmaygcaveqlpagelnscgrrvrfqapsghhfelyadkeytgkwglndvnpeawprdlkgm
aavrfdhalmygdelpatydlftkvlgfylaeqvldengtrvaqflslstkaHdvafihhpekgrlhhvsfhl

etwedllraadlismtdtsidigptrHglthgktiYffdpsgnrnevfcggdynypdhkpvtwttdqlgkaif
yhdrilnerfmtvlt
>1VNC_A
mgsvtpiplpkidepeeyntnyilfwnhvglelnrvthtvggpltgpplsaralgmlhlaihdayfsicpptd
fttflspdtenaayrlpspngandarqavagaalkmlsslymkpveqpnpnpganisdnayaqlglvldrsvl
eapggvdresasfmfgedvadvffallndprgasqegyhptpgrykfddepthpvvlipvdpnnpngpkmpfr
qyhapfygkttkrfatqsehfladppglrsnadetaeyddavrvaiamggaqalnstkrspwqtaqglywayd
gsnligtpprfynqivrriavtykkeedlansevnnadfarlfalvdvactdagifswkeKwefefwrplsgv
rddgrpdhgdpfwltlgapatntndipfkppfpaypsgHatfggavfqmvrryyngrvgtwkddepdniaidm
miseelngvnrdlrqpydptapiedqpgivrtrivrhfdsawelmfenaisriflgvhwrfdaaaardilipt
ttkdvyavdnngatvfqnvedirytttrgtredeeglfpiggvplgieiadeifnnglkptppeiqpmpqetpv
qkpvgqqpvkgmweeeqapvvkeap
>1PFQ_B
tadsrktytltdylkntyrlklyslrwisdheylykqennilvfnaeygnssvflenghsindysispdgqfi
lleynyvkqwrhsytasydiydlnkrqliteeripnntqwvtwspvghklayvwnndiyvkiepnlpsyritw
tgkediiyngitdwvyeeevfsaysalwwspngtflayaqfndtevplieysfysdeslqypktvrvpypkag
avnptvkffvvntdslssvtnatsiqitapasmligdhylcdvtwatqerislqwlrriqnysvmdicdydes
sgrwnclvarqhiemsttgwvgrfrpsephftldgnsfykiisneegyrhicyfqidkkdctfitkgtwevig
iealtsdylyyisneykgmpggrnlykiqlsdytkvtclscelnpercqyysvsfskeakyyqlrcsgpglpl
ytlhssvndkglrvlednsaldkmlqnvqmpskkldfiilnetkfwyqmilpphfdkskkyplllldvyagpcs
qkadtvfrlnwatylasteniivasfdgrgsgyqgdkimhainrrlgtfevedqieaarqfskmgfvdnkria
iwgwSyggyvtsmvlgsgsgvfkcgiavapvsrweyydsvyterymglptpednldhyrnstvmsraenfkqv
eyllihgtaDdnvhfqqsaqiskalvdvgvdfqamwytdedHgiasstahqhiythmshfikqcfslp
>1PMA_N
tvfspdgrlfqveyareavkkgStalgmkfangvllisdkkvrsrlieqnsieKiqliddyvaavtsGlvada
rvlvdfarisaqqekvtygslvnienlvkrvadqmqqytqyggvrpygvslifagidqigprlfdcdpagtin
eykataigSgkdavvsflereykenlpekeavtlgikalkssleegeelkapeiasitvgnkyriydqeevkk
fl
>1OHH_F
aaqaspspkagattgrivavigavvdvqfdeglppilnalevqgretrlvlevaqhlgestvrtiamdgtegl
vrgqkvldsgapiripvgpetlgrimnvigepidergpiktkqfaaihaeapefvemsveqeilvtgikvvdl
lapyakggkiglfggagvgKtvlimelinnvakahggysvfagvgERtregndlyhemiesgvinlkdatskv
alvygqmneppgararvaltgltvaeyfrdqegqdvllfidnifrftqagsevsallgripsavgyqptlatd
mgtmqeritttkkgsitsvqaiyvpaddltdpapattfahldattvlsraiaelgiypavdpldstsRimdpn
ivgsehydvargvqkilqdykslqdiiailgmdelseedkltvsrarkiqrflsqpfqvaevftghlgklvpl
ketikgfqqilageydhlpeqafymvgpieeavakadklaeehs
>1VAS_A
TrinltlvseladqhlmaeyRQlpRvfgavrkhvangkrvrdfkisptfilgaghvtffydkleflrkrqiel
iaeclkrgfnikdttvqdisdipqefrgdyipheasiaisqarldekiaqrptwykyygkaiya
>1OHH_C
adtsvdleetgrvlsigdgiarvhglrnvqaeemvefssglkgmslnlepdnvgvvvfgndklikegdivkrt
gaivdvpvgeellgrvvdalgnaidgkgpigskarrrvglkapgiiprisvrepmqtgikavdslvpigrgqr
eliigdrqtgKtsiaidtiinqkrfndgtdekkklyciyvaigQKrstvaqlvkrltdadamkytivvsatas
daaplqylapysgcsmgeyfrdngkhaliiyddlskqavayrqmslllrrppgreaypgdvfylhsrlleraa
kmndafgggsltalpvietqagdvsayiptnvisitdgqifletelfykgirpainvglsvsRvgsaaqtram
kqvagtmklelaqyrevaafadldaatqqllsrgvrltellkqgqyspmaieeqvaviyagvrgyldklepsk
itkfenaflshvisqhqallgkirtdgkiseesdaklkeivtnflagfea
>1GK9_B
SnmwvigkskaqdakaimvngpqfgwyapaytygiglhgagydvtgntpfaypglvfghngviswgstAgfgd
dvdifaerlsaekpgyylhngkwvkmlsreetitvkngqaetftvwrtvhgnilqtdqttqtayaksrawdgk
evasllawthqmkaknwqewtqqaakqaltinwyyadvngnigyvhtgaypdrqsghdprlpvpgtgkwdwkg
llpfemnpkvynpqsgyianwNnspqkdypasdlfaflwggadrvteidrlleqkprltadqawdvirqtsrq
dlnlrlflptlqaatsgltqsdprrqlvetltrwdginllnddgktwqqpgsailnvwltsmlkrtvvaavpm
pfdkwysasgyettqdgptgslnisvgakilyeavqgdkspipqavdlfagkpqqevvlaaledtwetlskry

```
gnnvsnwktpamaltfrannffgvpqaaaeetrhqaeyqnrgtendmivfspttsdrpvlawdvvapgqsgfi
apdgtvdkhyedqlkmyenfgrkslwltkqdveahkesqevlhvqr
>3R1R_A
mnqnllvtkrdgsterinldkihrvldwaaeglhnvsisqvelrshiqfydgiktsdihetiikaaadlisrd
apdyqylaarlaifhlrkkaygqfeppalydhvvkmvemgkydnhlledyteeefkqmdtfidhdrdmtfsya
avkqlegkylvqnrvtgeiyesaqflyilvaaclfsnypretrlqyvkrfydavstfkislptpimsgvrtpt
rqfssCvliecgdsldsinatssaivkyvsqragiginagriralgspirggeafhtgcipfykhfqtavksc
sqggvrggaatlfypmwhlevesllvlknnrgvegnrvrhmdygvqinklmytrllkgeditlfspsdvpgly
daffadqeeferlytkyekddsirkqrvkavelfslmmqerastgriyiqnvdhcnthspfdpaiapvrqsNl
ClEialptkplndvndengeialCtlsafnlgainnldeleelailavraldalldyqdypipaakrgamgrr
tlgigvinfayylakhgkrysdgsannlthktfeaiqyyllkasnelakeqgacpwfnettyakgilpidtyk
kdldtianeplhydwealresikthglrnstlsalmpsetssqisnatngiepprgyvsikaskdgilrqvvp
dyehlhdayellwempgndgylqlvgimqkfidqsisantnydpsrfpsgkvpmqqllkdlltaykfgvktly
yqntrdgaedaqddlvpsiqddgcesgacki
>1FDY_A
matnlrgvmaalltpfdqqqaldkaslrrlvqfniqqgidglyvggSTgeafvqslsereqvleivaeegkgk
ikliahvgcvttaesqqlaasakrygfdavsavtpfyypfsfeehcdhyraiidsadglpmvvynipalsgvk
ltldqintlvtlpgvgalKqtsgdlyqmeqirrehpdlvlyngydeifasgllagadggigstynimgwryqg
ivkalkegdiqtaqklqtecnkvidlliktgvfrglktvlhymdvvsvplcrkpfgpvdekylpelkalaqql
mqerg
>4EMW_B
pkivvvgavAggatcasqirrldkesdiiifekdrdmsfaNcalpyvigevvedrryalaytpekfydrkqit
vktyheviainderqtvsvlnrktneqfeesydklilspgasanslgfesditftlrnledTdaIDqfikanq
vdkvlvvgagYvslEvlenlyerglhptlihrsdkinklmdadmnqpildeldkreipyrlneeinaingnei
tfksgkvehydmiiegvgthpnskfiessnikldrkgfipvndkfetnvpniyaigdiatshyrhvdlpasvp
lawgaHraasivaeqiagndtiefkgflgnnivkffdytfasvgvkpnelkqfdykmvevtqgahanyypgns
plhlrvyydtsnrqilraaavgkegadkridvlsmammnqltvdeltefevaYappyshpkdlinmigyka
>3J36_F
mrhyeivfmvhpDqseqvpgmierytaaitgaegkihrledwgrrqlaypinklhkahyvlmnveapqevide
lettfrfndavirsmvmrtkhavteaspmvkakderrerrddfanetaddaeagdseeeeee
>1TJV_D
tdpimeklnssiaydqrlsevdiqgsmayakalekagiltktelekilsglekiseewskgvfvvkqsdedih
tanerrlkeligdiagklhtgrsrndqvvtdlklfmknslsiisthllqliktlveraaieidvilpgydHlq
kaqpirwsqfllshavaltrdserlgevkkrinvlplgsgalagnpldidremlrselefasislnsmdaise
rdfvveflsfatllmihlskmaedliiystsefgfltlsdafstgsslmpqKknpdslElirskagrvfgrla
silmvlkglpstynkdlqedkeavfdvvdtltavlqvatgvistlqiskenmekaltpemlatdlalylvrkg
vpfrqahtasgkavhlaetkgitinklsledlksispqfssdvsqvfnfvnsveqytalagtakssvttqieq
lrelmkkqkeqa
>3CSM_A
mdftkpetvlnlqniRdelvrmedsiifkfiershfatcpsvyeanhpgleipnfkgsfldwalsnleiahsr
irrfespdetpffpdkiqksflpsinypqilapyapevnyndkikkvyiekiipliskrdgddknnfgsvatr
dieclqslsrRihfgkfvaeaKfqsdiplytklikskdvegimknitnsaveekilerltkkaevygvdpter
rierritpeylvkiykeivipitkevEveyllrrlee
>1I1I_P
mtlgkelasplqamssytaagrnvlrwdlspeqiktrteqliaqtkqvydtvgtialkevtyenclqvladie
vtyivertmldfpqhvssdrevraasteadkklsrfdiemsmredvfqrivhlqetcdlekikpearryleks
ikmgkrnglhlsehirneiksmkkrmselcidfnknlneddtslvfskaelgalpddfidslektdedkykvt
lkyphyfpvmkkccvpetrrkmemafhtrckqentailqqllplraqvakllgynthadfvlelntakstsrv
aaflddlsqklkplgeaerefilslkkkeceergfeydgkinawdlhyymtqteelkysvdqeslkeyfpiev
vtegllsiyqellglsfeqvpdahvwnksvslytvkdkatgevlgqfyldlypregkynhaacfglqpgcllp
dgsrmmsvaalvvnfsqpvagrpsllrhdevrtyfhefghvmhqicaqtdfarfsgtnvetdfvEvpsqmlen
wvwdvdslrklskhykdghpitdelleklvasrlvntglltlrqivlskvdqslhtnatldaaseyakyctei
lgvaatpgtnmpatfghlaggydgqyygYlwsevfsmdmfhscfkkegimnpevgmkyrnlilkpggsldgmd
mlqnflqrepnqkaflmsrglngs
```

>1IPH_A
msqhneknphqhqsplhdsseakpgmdslapedgshrpaaeptppgaqptapgslkapdtrneklnsledvrk
gsenyalttnqgvriaddqnslragsrgptlledfilrekithfdheriperivHargsaahgyfqpykslsd
itkadflsdpnkitpvfvrfStvqggagsadtvrdirgfatkfyteegifdlvgNntpiffiqdahkfpdfvh
avkpephwaipqgqsahdtfwdyvslqpetlhnvmwamsdrgiprsyrtmegfgihtfrlinaegkatfvrfh
wkplagkaslvwdeaqkltgrdpdfhrrelweaieagdfpeyelgfqlipeedefkfdfdlldptklipeelv
pvqrvgkmvlnrnpdnffaeneqaafhpghivpgldftndpllqgrlfsytdtqisrlggpnfheipinrptc
pyhnfqrdgmhrmgidtnpanyepnsindnwpretppgpkrggfesyqervegnkvrerspsfgeyyshprlf
wlsqtpfeqrhivdgfsfelskvvrpyirervvdqlahidltlaqavaknlgieltddqlnitpppdvnglkk
dpslslyaipdgdvkgrvvaillndevrsadllailkalkakgvhakllysrmgevtaddgtvlpiaatfaga
psltvdavivpcgniadiadngdanyylmeaykhlkpialagdarkfkatikiadqgeegiveadsadgsfmd
elltlmaahrvwsripkidkipa
>1B8G_A
mlsrnatssyflgwqeyeknpyhevhntngiiqmglaenqlcfdlleswlaknpeaaafkkngesifaelalf
qdyhglpafkkamvdfmaeirgnkvtfdpnhlvltagatsanetfifcladpgeavliptpyYpgfdrdlkwr
tgveivpihctssngfqitetaleeayqeaekrnlrvkgvlvtnpsnplgttmtrnelylllsfvedkgihli
sDeiysgtafsspsfisvmevlkdrncdensevwqrvhvvyslsKdlglpgfrvgaiysnddmvvaaatkmss
fglvssqtqhllsamlsdkkltknyiaenhkrlkqrqkklvsglqksgisclngnaglfcwvdmrhllrsntf
eaemelwkkivyevhlnispgsschctepgwfrvcfanlpertldlamqrlkafvg
>1QH5_A
mkvevlpaltdnymylviddetkeaaivdpvqpqkvvdaarkhgvklttvltthhhwDhaggneklvklesgl
kvyggddrigalthkithlstlqvgslnvkclatpchtsghicyfvskpggseppavftgdtlfvagcgkfye
gtademckallevlgrlppdtrvycgheytinnlkfarhvepgnaaireklawakekysigeptvpstlaeef
tynpfmrvrektvqqhagetdpvttmravrrekdqfkmprd
>3J36_1
mkiinlgilahvDagkttltesllytsgaiaelgsvdegttrtdtmnlerqrgitiqtavtsfqwedvkvnii
dtpghmdflaevyrslsvldgavllvsakdgiqaqtrilfhalqimkiptiffinkidqegidlpmvyremka
klsseiivkqkvgqhphinvtdnddmeqwdavimgndellekymsgkpfkmseleqeenrrfqngtlfpvyhg
saknnlgirqlieviaskfysstpegqselcgqvfkieysekrrrfvyvriysgtlhlrdvirisekekikit
emyvptngelyssdtacsgdivilpndvlqlnsilgneillpqrkfienplpmiqttiavkkseqreillgal
teisdcdpllkyyvdttheiilsflgnvqmevicaileekyhveaeikeptviymerplrkaeytihievpp
npfwasvglsieplpigsgvqyesrvslgylnqsfqnavmegvlygceqglygwkvtdckicfeyglyyspvs
tpadfrllspivleqalkkagtellepylhfeiyapqeylsrayhdaprycadivstqikndevilkgeipar
ciqeyrndltyftngqgvcltelkgyqpaigkficqprrpnsridkvrhmfhkla
>2YZC_E
tkvvlgqnqygkaevrlvkvtrntarheiqdlnvtsqlrgdfeaahtagdnahvvatdtqkntvyafardgfa
tteefllrlgkhftegfdwvtggrwaaqqffwdrindhdhafsrnksevrtavleisgseqaivagiegltvl
kstgsefhgfprdkyttlqettdrilatdvsarwryntvevdfdavyasvrglllkafaethslalQqtmyem
graviethpeideikmslpnkHhflvdlqpfgqdnpnevfyaadrpyglieatiqregsradhpiwsn
>1BYA_A
atsdsnmllnyvpvyvmlplgvvnvdnvfedpdglkeqllqlraagvdgvmvdvwwgiielkgpkqydwrayr
slfqlvqecgltlqaimsfhqcggnvgDivnipipqwvldigesnhdifytnrsgtrnkeyltvgvdnepifh
grtaieiysdymksfrenmsdflesgliidievglgpagElrypsypqsqgwefprigefqcydkylkadfka
avaraghpewelpddagkyndvpestgffksngtyvtekgkffltwysnkllnhgdqildeankaflgckvkl
aikvsgihwwykvenhaaeltagyynlndrdgyrpiarmlsrhhailnftclemrdseqpsdaksgpqelvqq
vlsggwredirvagenalprydataynqiilnakpqgvnnngppklsmfgvtylrlsddllqksnfnifkkfv
lkmhadqdycanpqkynhaitplkpsapkipievlleatkptlpfpwlpetdmkvdg
>2ALR_A
aascvllhtgqkmpliglgtwksepgqvkaavkyalsvgyrhidcaaiYgnepeigealkedvgpgkavpree
lfvtsKlwntkhhpedvepalrktladlqleyldlylmhwpyafergdnpfpknadgticydsthyketwkal
ealvakglvqalglsnfnsrqiddilsvasvrpavlqvechpylaqneliahcqarglevtaysplgssdraw
rdpdepvlleepvvlalaekygrspaqillrwqvqrkvicipksitpsrilqnikvfdftfspeemkqlnaln
knwryivpmltvdgkrvprdaghplypfndpy
>1CTT_A

```
mhprfqtafaqladnlqsalepiladkyfpalltgeqvsslksatgldedalafallplaaacartplsnfnv
gaiargvsgtwyfganmefigatmqqtvhaEqsaishawlsgekalaaitvnytpcghcrqfmnelnsgldlr
ihlpgreahalrdylpdafgpkdleiktllmdeqdhgyaltgdalsqaaiaaanrshmpyskspsgvaleckd
grifsgsyaenaafnptlpplqgalillnlkgydypdiqravlaekadapliqwdatsatlkalgchsidrvl
la
>1D8C_A
maqtitqsrlridanfkrfvdeevlpgtgldaaafwrnfdeivhdlapenrqllaerdriqaaldewhrsnpg
pvkdkaayksflrelgylvpqpervtvettgidseitsqagpqlvvpamnaryalnaanarwgslydalygsd
iipqegamvsgydpqrgeqviawvrrfldeslplengsyqdvvafkvvdkqlriqlkngkettlrtpaqfvgy
rgdaaaptcillknnglhielqidangrigkddpahindviveaaistilDcEdsvaavdaedkillyrnllg
lmqgtlqekmekngrqivrklnddrhytaadgseislhgrsllfiRnvghlmtipviwdsegneipegildgv
mtgaialydlkvqknsrtgsvyivkpkmhgpqevafanklftrietmlgmapntlkmgimdeerrtslnlrsc
iaqarnrvafintgfldrtgdemhsvmeagpmlrknqmkstpwikayernnvlsglfcglrgkaqigkgmwam
pdlmadmysqkgdqlragantawvpsptaatlhalhyhqtnvqsvqaniaqtefnaefeplllddlltipvaen
anwsaqeiqqeldnnvqgilgyvvrwveqgigcskvpdihnvalmeDratlrissqhianwlrhgiltkeqvq
aslenmakvvdqqnagdpayrpmagnfanscafkaasdliflgvkqpngytepllhawrlrekesh
>3Q14_A
amhprkdwyeltratnwtpsyvteeqlfpermsghmgiplekwesydepyktsypeyvsiqrekdagaysvka
alerakiyensdpgwistlkshygaiavgeyaavtgegrmarfskapgnrnmatfgmmdelrhgqlQlffphe
yckkdrqfdwawrayhsnewaaiaakhffddiitgrdaisvaimltfsfetgfTnmqflglaadaaeagdytf
anlissiqtdesrhaqqggpalqlliengkreeaqkkvdmaiwrawrlfavltgpvmdyytpledrsqsfkef
myewiigqferslidlgldkpwywdlflkdidelhhsyhmgvwywrttawwnpaagvtpeerdwleekypgwn
krwgrcwdvitenvlndrmdlvspetlpsvcnmsqiplvgvpgddwnievfslehngrlyhfgsevdrwvfqq
dpvqyqnhmnivdrflagqiqpmtlegalkymgfqsieemgkdahdfawadkc
>1XA8_A
ghmvdtsgvkihpavdngikpaqpgfaggtlhckcstnpvrvavraqtahnhvcgCtkcwkpegaifsqvavv
grdalevlegaekleivnaeapiqrhrcrdcgvhmygrienrdhpfygldfvhtelsdedgwsapefaafvss
iiesgvdpsrmeairarlrelglepydalspplmdaiathiakrsgalaa
>2ADM_A
mglppllslpsnsaprslgrvetppevvdfmvslaeaprggrvlepacahgpflrafreahgtgyrfvgveid
pkaldlppwaegiladfllwepgeafdlilgNPpYgivgeaskypihvfkavkdlykkafstwkgkynlygaf
lekavrllkpggvlvfvvpatwlvledfallreflaregktsvyylgevfpqkkvsavvirfqksgkglslwd
tqesesgftpilwaeyphwegeiirfeteetrkleisgmplgdlfhirfaarspefkkhpavrkepgpglvpv
ltgrnlkpgwvdyeknhsglwmpkerakelrdfyatphlvvahtkgtrvvaawderaypwreefhllpkegvr
ldpstlvqwlnseamqkhvrtlyrdfvphltlrmlerlpvrreygfhtspesarnf
>1MVN_A
mengkrdrqdmevnttprkprvllaasgsvaaikfgnlchcftewaevravvtksslhfldklslpqevtlyt
dedewsswnkigdpvlHielrrwadvlviaplsantlgkiagglcdnlltciirawdytkplfvapamntlmw
nnpfterhllsldelgitlippikkrlaSgdygngamaepsliystvrlfwesqahqqtggts
>1JNR_B
mpsfvnpekcdgckalertaceyicpndlmtldkekmkaynrepdmcWecyscvkmcpqgaidvrgyvdyspl
ggacvpmrgtsdimwtvkyrngkvlrfkfairttpwgsiqpfegfpepteealksellagepeiigtsefpqv
kkka
>3NC6_A
iklfsvlsdqfqnnpyayfsqlreedpvhyeesidsyfisryhdvryilqhpdifttkslveraakrrivvrs
figdaldhlsplikqnaenllapylergksdlvndfgktfavcvtmdmlgldkrdhekisewhsgvadfitsi
sqSpearahslwcseqlsqylmpvikerrvnpgsdlisilctlsdkdilalilnvllaatEPAdktlalmiyh
llnnpeqmndvladrslvpraiaetlrykppVqliprqlsqdtvvggmeikkdtivfcmigaanrdpeafeqp
dvfnihredlgiksafsgaarhlaFgsgihnCVGtafaknEieivanivldkmrnirleedfcyaesglytrg
pvsllvafd
>1JNR_A
mvyypkkyelykadevptevvetdiliigggfsgcgaayeaaywaklgglkvtlvekaaversgavaqglsai
NtyidltgrserqntledyvryvtldmmglaredlvadyarhvdgtvhlfekwglpiwktpdgkyvrEgqwqi
mihgesykpiiaeaakmavgeeniyervfifellkdndpnavagavgfsvrepkfyvfkakavilatggatl
```

lfrprstgeaagrtWyaifdtgsgyymglkagamltqfehrfipfRfkdgygpvgawflffkckaknaygeey
iktraaelekykpygaaqpiptplrnhqvmleimdgnqpiymhteealaelaggdkkklkhiyeeafeDfldm
tvsqallwacqnidpqeqpseaapaepyimgsHsgeagfwvcgpedlmpeeyaklfplkynrmttvkglfaig
dcaganphkfSsgsftegriaakaavrfileqkpnpeiddavveelkkkayapmerfmqykdlstaddvnpey
ilpwqglvrlqkimdeyaagiatiyktnekmlqralellaflkedleklaardlhelmrawelvhrvwtaeah
vrhmlfrketrwpgyyyrtdypelndeewkcfvcskydaekdewtfekvpyvqviewsf
>1LDM_A
atlkdklighlatsqeprsynkitvvgvgavgmacaisilmkdladevalvdvmedklkgemmdlqhgslflh
takivsgkdysvsagsklvvitagarqqegesrlnlvqrnvnifkfiipnivkhspdciilvvsnpvdvltyv
awklsglpmhriigsgcnlDsaRfrylmgerlgvhscschgwvigeHgdsvpsvwsgmnvasiklhpldgtnk
dkqdwkklhkdvvdsayeviklkgytswaiglsvadlaetimknlcrvhpvstmvkdfygikdnvflslpcvl
ndhgisnivkmklkpneeqqlqksattlwdiqkdlkf
>1EX1_A
dyvlykdatkpvedrvadllgrmtlaekigqmtqierlvatpdvlrdnfigsllsgggsvprkgatakewqdm
vdgfqkacmstrlgipmiygidavhgqnnvygatifphnvglgatrdpylvkrigeatalevratgiqyafap
ciavcrdprwgrcyesysedrrivqsmtelipglqgdvpkdftsgmpfvagknkvaacakhfvgdggtvdgin
enntiinreglmnihmpayknamdkgvstvmisysswngvkmhanqdlvtgylkdtlkfkgfvisDwegidri
ttpagsdysysvkasilagldmimvpnkyqqfisiltghvnggvipmsriddavtrilrvkftmglfenpyad
pamaeqlgkqehrdlareaarkslvllkngktstdapllplpkkapkilvagshadnlgyqcggwtiewqgdt
grttvgttileavkaavdpstvvvfaenpdaefvksggfsyaivavgehpytEtkgdnlnltipepglstvqa
vcggvrcatvlisgrpvvvqpllaasdalvaawlpgsegqgvtdalfgdfgftgrlprtwfksvdqlpmnvgd
ahydplfrlgyglttnatkky
>1OZT_M
atkavcvlkgdgpvqgiinfeqkesngpvkvwgsikglteglhgfrvhefgdntagctsagpHfnplsrkhgg
pkdeerhvgdlgnvtadkdgvadvsiedsvislsgdhciigrtlvvhekaddlgkggneestktgnagsRlac
gvigiaq
>1G8P_A
mttavarlqpsasgaktrpvfpfsaivgqedmklallltavdpgiggvlvfgdrgtgkstavralaallpeie
avegcpvsspnvemipdwatvlstnvirkptpvvdlplgvsedrvvgaldieraiskgekafepgllaranrg
ylyidecnlledhivdllldvaqsgenvverdglsirhparfvlvgsgnpeegdlrpqlldrfglsvevlspr
dvetrvevirrrdtydadpkafleewrpkdmdirnqilearerlpkveapntalydcaalcialgsdglRgel
tllrsaralaalegatavgrdhlkrvatmalshrlrrdpldeagstarvartveetlp
>1AYX_A
aypsfeaysnykvdrtdletfldkqkevslyyllqniaypegqfnngvpgtviaspstsnpdyyyqwtrdsai
tfltvlselednnfnttlakaveyyintsynlqrtsnpsgsfddenhkglgepkfntdgsaytgawgrpqndg
palrayaisrylndvnslnegklvltdsgdinfsstediykniikpdleyvigywdstgfdlwEenqgrhfft
slvqqkalayavdiaksfddgdfantlsstastlesylsgsdggfvntdvnhivenpdllqqnsrqgldsaty
igpllthdigessstpfdvdneyvlqsyylllednkdrysvnsaysagaaigrypedvyngdgssegnpwfla
tayaaqvpyklaydaksasnditinkinydffnkyivdlstinsayqssdsvtiksgsdefntvadnlvtfgd
sflqvildhinddgslnEqlnrytgystgaysltwssgalleairlrnkvkala
>1NBA_A
mtetsgtfndiearlaavleeafeagtsiynergfkrrigygnrpavihiDlanawtqpghpfscpgmetiip
nvqrineaarakgvpvfyttnvyrnrdassgtndmglwyskiptetlpadsywaqiddriapadgevvieKnr
asafpgtnlelfltsnridtlivtgATaagCvrhtvedaiakgfrpiipretigdrvpgvvqwnlydidnkfg
dvestdsvvqyldalpqfedtvpktlsdpqpeveapadpvfaeqh
>1FGJ_A
distvpdetydalkldrgkatpketyealvkrykdpahgagkgtmgdywepiaisiymdpntfykppvspkev
aerkdcvechsdetpvwvrawkrsthanldkirnlksddplyykkgkleevennlrsmgklgeketlkevgci
dchvdvnkkdkadhtkdirmptadtcgtchlrefaereserdtmvwpngqwpagrpshaldytaniettvwat
mpqrevaegctmchtnqnkcdnchtrhefsaaesrkpeacatchsgvDHnnweaytmskhgklaemnrdkwnw
evrlkdafskggqnaptcaachmeyegeythnitrktrwanYpfvpgiaenitsdwsearldswvltctqchs
erfarsyldlmdkgtleglakyqeanaivhkmyedgtltgqktnrpnppepekpgfgiftqlfwskgnnpasl
elkvlemgennlakmhvglahvnpggwtYtegwgpmnrayveiqdeytkmqelsalqarvnklegkqtslldl
kgtgekislgglgggmllagalaligwrkrkqtra

```
>1BS4_A
svlqvlhipderlrkvakpveevnaeiqrivddmfetmyaeegiGlaatQvdihqriividvsenrderlvli
npelleksgetgieegcLsipeqralvpraekvkiraldrdgkpfeleadgllaiciqhEmdhlvgklfmdyl
splkqqrirqkvekldrlkara
>1QRS_A
tnfirqiidedlasgkhttvhtrfppEpngylhighaksiclnfgiaqdykgqcnlrfddtnpvkedieyves
ikndvewlgfhwsgnvryssdyfdqlhayaielinkglayvdeltpeqireyrgtltqpgknspyrdrsveen
lalfekmraggfeegkaclrakidmaspfivmrdpvlyrikfaehhqtgnkwciypmydfthcisdalegith
slctlefqnnrrlydwvldnitipvhprqyefsRlnleytvmsKrklnllvtdkhvegwddprmptisglrrr
gytaasirefckrigvtkqdntiemaslescirednlenapramavidpvklvienyqgegemvtmpnhpnkp
emgsrqvpfsgeiwidradfreeankqykrlvlgkevrlrnayvikaervekdaegnittifctydadtlgvi
hwvsaahalpveirlydrlfsvpnpgaaddflsvinpeslvikqgfaepslkdavagkafqferegyfcldsr
hstaekpvfnrtvglrdt
>1DBF_A
mmirgirgattverdteeeilqktkqllekiieenhtkpedvvqmllsatpdlhavfpakavrelsgwqyvpv
tcmqemdvtgglkkciRvmmtvqtdvpqdqirhvylekavvlrpdlsltkntel
>1FGH_A
rakvamshfepheyirydlleknidivrkrlnrpltlsekivyghlddpanqeiergktylrlrpdrvamqda
taqmamlqfissglpkvavpstihcDHlieaqlggekdlrrakdinqevynflatagakygvgfwrpgsgiih
qiilenyaypgvlligtDshtpngggLggicigvggadavdvmagipwelkcpkvigvkltgslsgwtspkdv
ilkvagiltvkggtgaiveyhgpgvdsisctgmaticnmgaeigattsvfpynhrmkkylsktgradianlad
efkdhlvpdsgchydqlieinlselkphingpftpdlahpvaevgsvaekegwpldirvgligsctnssyedm
grsaavakqalahglkcksqftitpgseqiratierdgyaqvlrdvggivlanacgpcigqwdrkdikkgekn
tivtsynRnftgrndanpethafvtspeivtalaiagtlkfnpetdfltgkdgkkfkleapdadelpraefdp
gqdtyqhppkdssgqrvdvsptsqrlqllepfdkwdgkdledlqilikvkgkcttdhisaagpwlkfrghldn
isnnlligainsenrkansvrnavtqefgpvpdtaryykqhgirwvvigdenygegSsRehsaleprflggra
iitksfarihetnlkkqgllpltfadpadynkihpvdkltiqglkdfapgkpltciikhpngtqetillnhtf
netqiewfragsalnrmkelqqk
>1DOZ_A
srkkmgllvmaygtpykeedieryythirrgrkpepemlqdlkdryeaiggisplaqiteqqahnleqhlnei
qdeitfkayiglkhiepfiedavaemhkdgiteavsivlaphfstfsvqsynkrakeeaeklggltitsvesw
ydepkfvtywvdrvketyasmpederenamlivsaHslpekikefgdpypdqlhesakliaegagvseyavgw
qsegntpdpwlgpdvqdltrdlfeqkgyqafvyvpvgfvadHlEvlydndyeckvvtddigasyyrpempnak
pefidalatvvlkklgr
>1O88_A
atdtggyaataggnvtgavsktatsmqdivniidaarldangkkvkggayplvitytgnedslinaaaanicg
qwskdprgveikeftkgitiigangssanfgiwikkssdvvvqnmrigylpggakdgDmirvddspnvwvdhn
elfaanhecdgtpdndttfesavdikgasntvtvsynyihgvkkvgldgssssdtgrnityhhnyyndvnaRl
plqrgglvhaynnlytnitgsglnvrqngqaliennwfekainpvtsrydgknfgtwvlkgnnitkpadfsty
sitwtadtkpyvnadswtstgtfptvaynyspvsaqcvkdklpgyagvgknlatltstack
>1J7N_A
ernktqeehlkeimkhivkievkgeeavkkeaaekllekvpsdvlemykaiggkiyivdgditkhislealse
dkkkikdiygkdallhehyvyakegyepvlviqssedyventekalnvyyeigkilsrdilskinqpyqkfld
vlntiknasdsdgqdllftnqlkehptdfsvefleqnsnevqevfakafayyiepqhrdvlqlyapeafnymd
kfneqeinlsleelkdqrmlsryekwekikqhyqhwsdslseegrgllkklqipiepkkddiihslsqeekel
lkriqidssdflsteekeflkklqidilsekekeflkklkldiqpydinqrlqdtgglidspsinldvrkqyk
rdiqnidallhqsigstlynkiylyenmninnltatlgadlvdstdntkinrgifnefkknfkysissnymiv
dinerpaldnerlkwriqlspdtragylengklilqrnigleikdvqiikqsekeyiridakvvpkskidtki
qeaqlninqewnkalglpkytklitfnvhnryasnivesaylilnewknniqsdlikkvtnylvdgngrfvft
ditlpniaeqythqdeiyeqvhskglyvpesrsillhgpskgvelrndsegfihEfghavddyagylldknqs
dlvtnskkfidifkeegsnltsYgrtneaeffaeafrlmhstdhaerlkvqknapktfqfindqikfi
>2TMD_A
ardpkhdilfepiqigpktlrnrfyqvphcigagsdkpgfqsahrsvkaeggwaalnteycsinpesddthrl
sariwdegdvrnlkamtdevhkygalagvelwyggahapnmesratprgpsqyasefetlsyckemdlsdiaq
```

```
vqqfyvdaakrsrdagfdivyvYgaHsylplqflnpyynkrtdkyggslenrarfwletlekvkhavgsdcai
atrfgvdtvygpgqieaevdgqkfvemadslvdmwditigdiaewgeDagpsrfyqqghtipwvklvkqvskk
pvlgvgrytdpekmieivtkgyadiigcarpsiadpflpqkveqgryddirvcigcnvcisrweiggppmict
qnatageeyrrgwhpekfrqtknkdsvlivgagpsgseaarvlmesgytvhltdtaekigghlnqvaalpglg
ewsyhrdyretqitkllkknkesqlalgqkpmtaddvlqygadkviiatgarwntdgtnclthdpipgadasl
pdqltpeqvmdgkkkigkrvvilnadtyfmapslaeklataghevtivsgvhlanymhftleypnmmrrlhel
hveelgdhfcsriepgrmeiyniwgdgskrtyrgpgvsprdantshrwiefdslvlvtgrhsectlwnelkar
esewaendikgiyligdaeaprliadatftghrvareieeanpqiaipykretiawgtphmpggnfkieykv
>3CLA_A
mnytkfdvknwvRrehfefyrhrlpcgfsltskidittlkksddsaykfypvmiyliaqavnqfdelrmaik
ddelivwdsvdpqftvfhqetetfsalscpyssdidqfmvnylsvmeryksdtklfpqgvtpenhlnisalpw
vnfdsfnlnvanftdyfapiitmakyqqegdrlllplsvqvhHavcDgfhvarfinrlqelcnsklk
>1AH7_A
wsaedkhkegvnshlwivnraidimsrnttlvkqdrvaqlnewrtelengiyaaDyenpyydnstfashfydp
dngktyipfakqaketgakyfklagesyknkdmkqaffylglslhylgdvnqpmhaanftnlsypqgfhskye
nfvdtikdnykvtdgngywnwkgtnpeewihgaavvakqdysgivndntkdwfvkaavsqeyadkwraevtpm
tgkrlmdaqrvtagyiqlwfdtygdr
>1OFD_A
Cgvgfianlrgkpdhtlveqalkalgcmehrggcsadndsgdgagvmtaiprellaqwfntrnlpmpdgdrlg
vgmvflpqepsarevarayveevvrlekltvlgwrevpvnsdvlgiqaknnqphieqilvtcpegcagdeldr
rlyiarsiigkklaedfyvcsfscrtivykgmvrsiilgefyldlknpgytsnfavyhrrfstntmpkwplaq
pmrllghngeintllgninwmaarekelevsgwtkaelealtpivnqansdsynldsalellvrtgrspleaa
milvpeayknqpalkdypeisdfhdyysglqepwdgpallvfsdgkivgagldrnglrparycitkddyivlg
seagvvdlpevdivekgrlapgqmiavdlaeqkilknyqikqqaaqkypygewikiqrqtvasdsfaektlfn
daqtvlqqqaafgytaedvemvvvpmasqgkeptfcmgddtplavlshkprllydyfkqrfaqvtnppidplr
enlvmslamflgkrgnllepkaesartiklrsplvnevelqaiktgqlqvaevstlydldgvnsledaltnlv
ktaiatvqagaeilvltdrpngailtenqsfippllavgavhhhliraglrlkaslivdtaqcwsthhfaclv
gygasaicpylalesvrqwwldektqklmengrldridlptalknyrqsveaglfkilskmgisllasyhgaq
ifeaiglgaelveyafagttsrvggltiadvagevmvfhgmafpemakklenfgfvnyrpggeyhmnspemsk
slhkavaaykvggngnngeaydhyelyrqylkdrpvtalrdlldfnadqpaisleevesvesivkrfctggms
lgalsreahetlaiamnrlgaksnsgEggedvvryltlddvdsegnsptlphlhglqngdtansaikqiasgr
fgvtpeylmsgkqleikmaqgaKpgeggqlpgkkvseyiamlrrskpgvtlispphhdiysiedlaqliydl
hqinpeaqvsvklvaeigigtiaagvakanadiiqisghdggtgasplssikhagspwelgvtevhrvlmenq
lrdrvllradgglktgwdvvmaalmgaeeygfgsiamiaegcimarvchtnncpvgvatqqerlrqrfkgvpg
qvvnffyfiaeevrsllahlgyrslddiigrtdllkvrsdvqlsktqnltldcllnlpdtkqnrqwlnhepvh
sngpvldddiladpdiqeainhqttatktyrlvntdrtvgtrlsgaiakkygnngfegnitlnfqgaagqsfg
afnldgmtlhlqgeandyvgkgmnggeivivphpqasfapednviigntclygatggnlyangragerfavrn
svgkaviegagdhcceymtggvivvlgpvgrnvgagmtgglayfldevgdlpekinpeiitlqritaskgeeq
lkslitahvehtgspkgkailanwsdylgkfwqavppsekdspeanndvsltgektltsv
>1DIO_A
mrskrfealakrpvnqdgfvkewieegfiamespndpkpsikivngavteldgkpvsdfdlidhfiaryginl
nraeevmamdsvklanmlcdpnvkrseivplttamtpakivevvshmnvvemmmamqkmrarrtpsqqaHvtn
vkdnpvqiaadaaegawrgfdeqEttvavaryapfnaiallvgsqvgrpgvltqcsleeatelklgmlghtcy
aetisvygtepvftdgddtpwskgflassyasrglkmrftsgsgsevqmgyaegksmlylearciyitkaagv
qglQngsvscigvpsavpsgiravlaenlicssldlecassnDqtfthsdmrrtarllmqflpgtdfissgys
avpnydnmfagsnedaedfddynviqrdlkvdgglrpvreedviairnkaaralqavfagmglppitdeevea
atyahgskdmpernivedikfaqeiinknrnglevvkalaqggftdvaqdmlniqkakltgdylhtsaiivgd
gqvlsavndvndyagpatgyrlqgerweeiknipgaldpneid
>1HTI_A
apsrkffvggNwKmngrkqslgeligtlnaakvpadtevvcapptayidfarqkldpkiavaaqncykvtnga
ftgeispgmikdcgatwvvlgHserrhvfgesdeligqkvahalaeglgviacigekldereagitekvvfeq
tkviadnvkdwskvvlayEpvwaiGtgktatpqqaqevheklrgwlksnvsdavaqstriiyggsvtgatcke
lasqpdvdgflvggaslkpefvdiinakq
>1NDH_A
```

stpaitlenpdikyplrlidkevvnhdtrrfrfalpspehilglpvgqhiylsaridgnlvirpYtpvssddd
kgfvdlvikvyfkdthpkfpaggkmsqylesmkigdtiefrgpngllvyqgkgkfairpdkksspviktvksv
gmiaggtgitpmlqviraimkdpddhtvchllfanqtekdillrpeleelrnehsarfklwytvdrapeawdy
sqgfvneemirdhlpppeeeplvlmcgpppmiqyaclpnlervghpkercfaf
>1GE7_B
tyngcssseqsalaaaasaaqsyvaeslsylqthtaatpryttwfgsyissrhstvlqhytdmnsndfssysf
dctctaagtfayvypnrfgtvylcgafwkapttgtdsqagtlvhEsshftrnggtkdyaYgqaaakslatmdp
dkavmnadnheyfsennpaqs
>1J2U_A
msksvfvgeltwkeyearvaagdcvlmlpvgaleqhghhmcmnvdvllptavckrvaerigalvmpglqygyk
sqqksgggnhfpgttsldgatltgtvqdiirelarhgarrlvlmnghyEnsmfivegidlalrelryagiqdf
kvvvlsywdfvkdpaviqqlypegflgwdieHggvfetslmlalypdlvdldrvvdhppatfppydvfpvdpa
rtpapgtlssaktasrekgelilevcvqgiadaireefppt
>2UDP_B
mrvlvtggsgyigshtcvqllqnghdviildnlcnskrsvlpvierlggkhptfvegdirnealmteilhdha
idtvihfaglkavgesvqkpleyydnnvngtlrlisamraanvknfifssSATvygdqpkipyvesfptgtpq
spYgksKlmveqiltdlqkaqpdwsiallryfnpvgahpsgdMgedpQgipnnlmpyiaqvavgrrdslaifg
ndyptedgtgvrdyihvmdladghvvameklankpgvhiynlgagvgnsvldvvnafskacgkpvnyhfaprr
egdlpaywadaskadrelnwrvtrtldemaqdtwhwqsrhpqgypd
>1CD5_A
mrlipltaeqvgkwaarhivnrinafkptadrpfvlglptggtpmttykalvemhkagqvsfkhvvtfnmDe
yvglpkehpesyysfmhrnffdhvdipaeninllngnapdidaecrqyeekirsygkihlfmggvgnDgHiaf
nEpasslasrtriktlthdtrvansrffdndvnqvpkyaltvgvgtlldaeevmilvlgsqkalalqaavegc
vnhmwtisclqlhpkaimvcdepstmelkvktlryfneleaenikgl
>2CVY_A
tkqfskvvedlyryvnaatgkpapmisddvynivmenkdklnsaivydrdfqysyfgfktlersyllringqv
aerpqhlimrvalgihgrdieaaletynlmslkyfthasptlfnagtpkpqmssCflvamkedsiegiydtlk
ecalisktaggiglhihnirstgsyiagtngtsnglipmirvfnntaryvafalylepwhadifdfidirknh
gkeeirardlfpalwipdlfmkrveengtwtlfsptsapglsdcygdefealytryekegrgktikaqklwys
ileaqtetgtpfvvykdacnrksnqknlgvikssNlCcEiveysapdetavCnlasvalpafietsedgktst
ynfkklheiakvvtrnlnrvidrnyypveearksnmrhrpialgvqgladtfmllrlpfdseearllniqife
tiyhasmeascelaqkdgpyetfqgspasqgilqfdmwdqkpygmwdwdtlrkdimkhgvrnsltmapmptas
tsqilgynecfepvtsnmysfqvvnpyllrdlvdlgiwdegmkqylitqngsiqglpnvpqelkdlyktvwei
sqktiinmaadrsvyidqshslnlflraptmgkltsmhfygwkkglktgmyylrtq
>2FAH_C
lstslsalpaaardfveeavrlcrprevllcdgseeegkellrglqddgvlhplpkydncwlartdprdvarv
esktvlvtpeqsdavpppppsggppqlgnwmspnafqaavqerfpgcmagrplyvipfsmgpptsplaklgvq
vtdspyvvlsmrimtrvgpavlqrldddfvrclhsvgrplplteplvsswpcdpsrvlvahipserrivsfgs
gyggnsllgkkcfalriasrmaqqqgwlaeHmlilgvtspsgekrymaaafpsacgktnlammtpslpgwrih
cvgddiawmkfddegrlrainpergffgvapgtssrtnpnamatiarntiftnvglrsdggvywdgldeptep
gvtytswlgkpwkhgdpepcahpnsRfcapadqcpimdprwddpegvpidaiifggrrprgvplvveafgwrh
gvfmgsamrseataaaehkggrlmhdpfamrpffgynagrylehwlstglrsnarlprlfhvnwflrdnegrf
vwpgfghnarvlawifgriqgrdtarptpigwvpkegdldlgglpgvdysqlfpmekgfweeecrqlreyyge
nfgadlprdvmaelegleervrkm
>1Y7T_B
mkapvrvavtgaagqigysllfriaagemlgkdqpvilqlleipqamkalegvvmeledcafpllagleatdd
pkvafkdadyallvgaaprkagmerrdllqvngkifteqgralaevakkdvkvlvvgnpantnaliayknapg
lnprnftamtrlDhnrakaqlakktgtgvdrirrmtvwgNHsstmfpdlfhaevdgrpalelvdmewyekvfi
ptvaqrgaaiiqargassaasaanaaiehirdwalgtpegdwvsmavpsqgeygipegivysfpvtakdgayr
vvegleinefarkrmeitaqelldemeqvkalgli
>1QTW_A
mkyigahvsaagglanaairaaeidatafalftknqrqwraaplttqtidefkaacekyhytsaqilphdsyl
inlghpvtealeksrdafidemqrceqlglsllnfhpgshlmqiseedclariaesinialdktqgvtavien

tagqgsnlgfkfehlaaiidgvedksrvgvcidtchafaagydlrtpaecektfadfartvgfkylrgmhlnd
akstfgsrvdrhhslgegnighdafrwimqddrfdgiplilEtinpdiwaeeiawlkaqqtekava
>1CB8_A
qqtgtaelimkrvmldlkkplrnmdkvaeknlntlqpdgswkdvpykddamtnwlpnnhllqletiiqayiek
dshyygddkvfdqiskafkywydsdpksrnwwhneiatpqalgemlilmrygkkpldealvhkltermkrgep
ekktgaNktdialhyfyralltsdeallsfavkelfypvqfvhyeeglqydysylqHgpqlqissYgavfitg
vlklanyvrdtpyalsteklaifskyyrdsylkairgsymdfnvegRgvsrpdilnkkaekkrllvakmidlk
hteewadaiartdstvaagykiepyhhqfwngdyvqhlrpaysfnvrmvskrtrrsesgnkenllgrylsdga
tniqlrgpeyynimpvwewdkipgitsrdyltdrpltklwgeqgsndfaggvsdgvygasayaldydslqakk
awfffdkeivclgaginsnapenitttlnqswlngpvistagktgrgkittfkaqgqfwllhdaigyyfpega
nlslstqsqkgnwfhinnshskdevsgdvfklwinhgarpenaqyayivlpginkpeeikkyngtapkvlant
nqlqavyhqqldmvqaifytagklsvagieietdkpcavlikhingkqviwaadplqkektavlsirdlktgk
tnrvkidfpqqefagatvelk
>1QJ4_A
mafahfvlihtichgawiwhklkpllealghkvtaldlaasgvdprqieeigsfdeyseplltflealppgek
vilvgeScgglniaiaadkycekiaaavfhnsvlpdtehcpsyvvdklmevfpdwkdttyftytkdgkeitgl
klgftllrenlytlcgpeeyelakmltrkgslfqnilakrpfftkegygsikkiyvwtdqDeiflpefqlwqi
enykpdkvykveggdHklqltktkeiaeilqevadtyn
>3NKV_B
eydylfklllligdsgvgksclllrfaddtytesyistigvdfkirtieldgktiklqiwdtagQerfrtitss
yyrgahgiivvydvtdqesyanvkqwlqeidryasnvnkllvgnksdlttkkvvdnttakefadslgipflet
saknatnveqafmtmaaeikkrm
>3GDP_B
lattsdhdfsylsfaydatdlelegsydyvivgggtsgcplaatlsekykvlvlergslptaypnvltadgfv
ynlqqeddgktpverfvsedgidnvrgrvlggtsiinagvyarantsiysasgvdwdmdlvnqtyewvedtiv
ykpnsqswqsvtktafleagvhpnhgfsldheegtritgstfdnkgtrhaadellnkgnsnnlrvgvhasvek
iifsnapgltatgviyrdsngtphqafvrskgevivsagtigtpqllllsgvgpesylsslnipvvlshpyvg
qflhdnprnfinilppnpieptivtvlgisndfyqcsfsslpfttppfgffpsssyplpnstfahfaskvagp
lsygsltlksssnvrvspnvkfnyysnltdlsHcvsgmkkigellstdalkpykvedlpgvegfnilgiplpk
dqtddaafetfcresvasywHyHggclvgkvldgdfrvtginalrvvdgstfpytpasHpqgfylmlgryvgi
kilqersasd
>1UWQ_A
mysfpnsfrfgwsqagfqsemgtpgsedpntdwykwvhdpenmaaglvsgdlpengpgywgnyktfhdnaqkm
glkiaRlnvewsrifpnplprpqnfdeskqdvteveinenelkrldeyankdalnhyreifkdlksrglyfil
nmyhwplplwlhdpirvrrgdftgpsgwlstrtvyefarfsayiawkfddlvdeystmnEpnVvgglgyvgvk
sgfppgylsfelsrramyniiqaharaydgiksvskkpvgiiyanssfqpltdkdmeavemaendnrwwffda
iirgeitrekivrddlkgrldwigvNyYtrtvvkrtekgyvslggyghgcernsvslaglptsdfgweffpeg
lydvltkywnryhlymyvtEngiaddadyqrpyylvshvyqvhrainsgadvrgylhwsladnyewasgfsmr
fgllkvdyntkrlywrpsalvyreiatngaitdeiehlnsvppvkplrh
>1S3I_A
mkiavigqslfgqevycqlrkeghevvgvftipdkdgkadpdgleaekdgvpvfkfprwrargqalpevvaky
qalgaelnvlpfcsqfipmevinaprhgsiiyHpsllprhrgasainwtlihgdkkggftifwaddglDtgdl
llqkecevlpddtvstlynrflfpegikgmvqavrliaegtaprcpqseegatyegiqkketakinwdqpaea
ihnwirgndkvpgawteacgqkltffnstlntsglstqgealpipgahrpgvvtkaglilfgnddrmllvkni
qledgkmmpasqffkgsd
>1QRG_A
qeitvdefsnirenpvtpwnpepsapvidptayidpqasvigevtiganvmvspmasirsdEgmpifvgdrsn
vQdgvvlhalEtineegepiednivevdgkeyavyignnvslahqsqvhgpaavgddtfigmqafvfkskvgn
ncvleprsaaigvtipdgryipagmvvtsqaeadklpevtddyayshtneavvyvNvhlaegykets
>1BCR_B
tgsydpcterystayynrrdvqmalhanvtgamnytwatcsdtinthwhdaprsmlpiyreliaaglriwvfs
gdtDavvpltatrysigalglptttswypwyddqevggwsqvykgltlvsvrgagHevplhrprqalvlfqyf
lqgkpmpgqtknat
>1L0O_A

```
mrnemhlqfsarsenesfarvtvaafvaqldptmdelteiktvvsEavtnaiihgynndpngivsisviiedg
vvhltvrdegvgipdieearqplfttkpeleRsgmgftimenfmdevivesevnkgttvylkkhivkskryle
gssf
>1GPJ_A
medlvsvgithkeaeveelekarfesdeavrdivesfglsgsvllqtSnrvevyasgardraeelgdlihdda
wvkrgseavrHlfrvasglesmmvgeqeilrqvkkaydraarlgtldealkivfrrainlgkrareetriseg
avsigsaavelaerelgslhdktvlvvgagemgktvakslvdrgvravlvanrtyeravelardlggeavrfd
elvdhlarsdvvvsataaphpvihvddvrealrkrdrrspiliidianprdveegveniedvevrtiddlrvi
arenlerrrkeipkveklieeelstveeeleklkerrlvadvakslheikdreleralrrlktgdpenvlqdf
aeaytkrlinvltsaimelpdeyrraasralrraselng
>1DIN_A
mltegisiqsydghtfgalvgspakapapviviaqeIfgvnafmretvswlvdqgyaavcpdlyarqapgtal
dpqderqreqayklwqafdmeagvgdleaairyarhqpysngkvglvgyCLggalaflvaakgyvdravgyyg
vglekqlnkvpevkhpalfhmggqDhfvpapsrqlitegfganpllqvhwyeeagHsfartsssgyvasaaal
anertldflaplqs
>1V0Y_A
adsatphldaveqtlrqvspglegdvwertsgnkldgsaadpsdwllqtpgcwgddkcadrvgtkrllakmte
nignatrtvdistlapfpngafqdaivaglkesaakgnklkvrilvgaapvyhmnvipskyrdeltaklgkaa
enitlnvasmttsktafswnHskilvvdgqsaltgginswkddyldtthpvsDvdlaltgpaagsagryldtl
wtwtcqnksniasvwfaasgnagcmptmhkdtnpkaspatgnvpviavgglgvgikdvdpkstfrpdlptasd
tkcvvglhdntnadrdydtvnpeesalralvasakghieisqqdlnatcpplprydirlydalaakmaagvkv
rivvsdpanrgavgsggysqikslseisdtlrnrlanitggqqaaktamcsnlqlatfrsspngkwadghpya
qHhklvsvdsstfyigsknlypswlqDfgyivespeaakqldaklldpqwkysqetatvdyargicna
>1STD_A
mgsqvqksdeitfsdylglmtcvyewadsYDskdwdrlrkviaptlridYrsfldklweampaeefvgmvssk
qvlgdptlrtqHfiggtrwekvsedevigyhqlrvpHqrykdttmkevtmkghahsanlhwykkidgvwkfag
lkpdirwgefdfdrifedgretfgdk
>1MUG_A
mvedilapglrvvfcgiNpglssagtgfpfahpanrfwkviyqagftdrqlkpqeaqhlldyrcgvtklvdrp
tvqanevskqelhaggrkliekiedyqpqalailgkqayeqgfsqrgaqwgkqtltigstqiwvlpnpsglsr
vsleklveayreldqalvvrgr
>2THI_A
ahsdassditlkvaiypyvpdparfqaavldqwqrqepgvkleftdwdsysadppddldvfvldsiflshfvd
agyllpfgsqdidqaedvlpfalqgakrngevyglpqilCtnllfyrkgdlkigqvdniyelykkigtshseq
ipppqnkgllinmaggttkasmylealidvtgqyteydllppldplndkvirglrllinmagekpsqyvpedg
dayvraswfaqgsgrafigysEsmmrmgdyaeqvrfkpisssagqdiplfysdvvsvnsktahpelakklanv
masadtveqalrpqadgqypqyllparhqvyealmqdypiyselaqivnkpsnrvfrlgpevrtwlkdakqvl
pealgltdvsslas
>1ROZ_A
megslereapagalaavlkhsstlppestqvrgydfnrgvnyralleafgttgfqatnfgravqqvnamiekk
leplsqdedqhadltqsrrpltsctiflgytsnlissgiretirylvqhnmvdvlvttaggveEdlikclapt
ylgefslrgkelrenginrignllvpnenyckfedwlmpildqmvmeqntegvkwtpskmiarlgkeinnpes
vyywaqknhipvfspaltdgslgdmiffhsyknpglvldivedlrlintqaifakctgmiilgggvvkHhian
anlmrngadyavyintaqefdgsdsgarpdeavswgKirvdaqpvkvyadaslvfpllvaetfaqkmdafmhe
kned
>1CB7_B
melknkkwtdeefhkqreevlqqwptgkevdlqeavdylkkipaeknfaeklvlakkkgitmaqpragvalld
ehiellrylqdeggadflpstidaytrqnrydecengikesekagrsllngfpgvnfgvkgcrkvleavnlpl
qarhgtpdsrllaeiihaggwtsnEgggisynvpyaknvtiekslldwqycdrlvgfyeeqgvhinrepfgpl
tgtlvppsmsnavgiteallaaeqgvknitvgygecgnmiqdiaalrcleeqtneylkaygyndvfvttvfhq
wmggfpqdeskafgvivtattiaalagatkvivktpheaigiptkeanaagikatkmalnmlegqrmpmskel
etemavikaetkcildkmfelgkgdlaigtvkafetgvmdipfgpskynagkmmpvrdnlgcvrylefgnvpf
teeiknynrerlqerakfegrdvsfqmviddifavgkgrligrpe
>1DYP_A
```

smqppiakpgetwilqakrsdefnvkdatkwnfqtenygvwswknenatvskgklklttkreshqrtfwdgcn
qqqvanyplyytsgvaksratgnygyyearikgastfpgvspafwmystidrsltkegdvqysEiDvVeltqk
savresdhdlhnivvkngkptwmrpgsfpqtnhngyhlpfdprndfhtygvnvtkdkitwyvdgeivgekdnl
ywhrqmnltlsqglraphtqwkcnqfypsanksaegfptsmevdyvrtwvkv
>1LAM_A
tkglvlgiyskekeedepqftsagenfnklvsgklreilnisgpplkagktrtfyglhedfpsvvvvglgkkt
agideqenwhegkeniraavaagcrqiqdleipsvevdpcgdaqaaaegavlglyeyddlkqkrkvvvsaklh
gsedqeawqrgvlfasgqnlarrlmetpanemtptkfaeiveenlksasiktdvfirpkswieeqemgsflsv
akgseeppvfleihykgspnasepplvfvgkgitfDsggisiKaaanmdlmradmggaaticsaivsaakldl
pinivglaplcenmpsgkankpgdvvrarngktiqvdntdaegRliladalcyahtfnpkviinaatltgamd
ialgsgatgvftnsswlwnklfeasietgdrvwrmplfehytrqvidcqladvnnigkyrsagactaaaflke
fvthpkwahldiagvmtnkdevpylrkgmagrptrtlieflfrfsq
>2NAC_A
akvlcvlyddpvdgypktyarddlpkidhypggqtlptpkaidftpgqllgsvsgelglrkylesnghtlvvt
sdkdgpdsvferelvdadvvisqpfwpayltperiakaknlklaltagigsdhvdlqsaidrnvtvaevtycN
sisvaehvvmmilslvrnylpshewarkggwniadcvshaydleamhvgtvaagriglavlrrlapfdvhlhy
tdrhrlpesvekelnltwhatredmypvcdvvtlncplhpetehmindetlklfkrgayivntaRgklcdrda
varalesgrlagyagdvwfpQpapkdhpwrtmpyngmtpHisgttltaqaryaagtreilecffegrpirdey
livqggalagtgahsyskgnatggseea
>1E7Q_A
makqrvfiaghrgmvgsairrqleqrgdvelvlrtrdelnlldsravhdffaseridqvylaaakvggivann
typadfiyqnmmiesniihaahqndvnkllflgAsCiypklakqpmaesellqgtleptnepYaiaKiagikl
cesynrqygrdyrsvmptnlygphdnfhpsnsHvipallrrfheataqsapdvvvwgsgtpmreflhvddmaa
asihvmelahevwlentqpmlshinvgtgvdctirelaqtiakvvgykgrvvfdaskpdgtprklldvtrlhq
lgwyheisleaglastyqwflenqdrfrg
>1OG1_A
ltgplmldtapnafddqyegcvnkmeekaplllqedfnmnaklkvaweeakkrwnnikpsrsypkgfndfhgt
alvaytgsiavdfnravrefkenpgqfhykafhyyltralqllsngdchsvyrgtktrfhytgagsvrfgqft
SsslskkvaqsqEffsdhgtlfiiktclgvyikefsfRpdqeEvlipgyevyqkvrtqgyneifldspkrkks
nynclys
>1DE3_A
avtwtclndqknpktnkyetkrllynqnkaesnshhaplsdgktgssypHwftngydgdgklpkgrtpikfgk
sdcdrppkhskdgngktdhyllEfptfpdghdykfdskkpkenpgparviytypnkvfcgiiaHtkenqgelk
lcsh
>1CHM_A
qmpktlrirngdkvrstfsaqeyanrqarlrahlaaenidaaiftsyhninyysdflycsfgrpyalvvtedd
visisanidggqpwrrtvgtdnivytdwqrdnyfaaiqqalpkarrigiehdhlnlqnrdklaarypdaelvd
vaaacmrmrmiksaeehvmirhgariadiggaavvealgdqvpeyevalhatqamvraiadtfedvelmdtwt
wfqsgintdgaHnpvttrkvnkgdilslncfpmiagyytalErtlfldhcsddhlrlwqvnvevheaglklik
pgarcsdiarelneiflkhdvlqyrtfgyghsfgtlsHyygreaglelredidtvlepgmvvsmEpmimlpeg
lpgaggyrehdilivnengaenitkfpygpekniir
>1A50_B
ttllnpyfgefggmyvpqilmpalnqleeafvraqkdpefqaqfadllknyagrptaltkcqnitagtrttly
lkredllhggaHKtnqvlgqallakrmgkseiiaetgagqhgvasalasallglkcriymgakdverqspnvf
rmrlmgaevipvhsgsatlKdacnealrdwsgsyetahymlgtaagphpyptivrefqrmigeetkaqildke
grlpdaviacvgggsnaigmfadfindtsvgligvepgghgietgehgaplkhgrvgiyfgmkapmmqtadgq
ieesysisaglDfpsvgpqhaylnsigradyvsitddealeafktlcrhegiipalesshalahalkmmreqp
ekeqllvvnlSgrgdkdiftvhdilkargei
>1AQ2_A
mrvnngltpqeleaygisdvhdivynpsydllyqeeldpsltgyergvltnlgavavdtgiftgrspkdkyiv
rddttrdtfwwadkgkgkndnkplspetwqhlkglvtrqlsgkrlfvvdafcganpdtrlsvrfitevawqah
fvknmfirpsdeelagfkpdfivmngakctnpqwkeqglnsenfvafnltermqliggtwyggemkkgmfsmm
nyllplkgiasmHcsanvgekgdvavffglsgtgKttlstdpkrrligddehgwdddgvfnfeggcyaktikl
skeaepeiynairrdallenvtvredgtidfddgsktentRvsypiyhidnivkpvskaghatkvifltadaf

gvlppvsrltadqtqyhflsgftaklagtergiteptptfsacfgaaflslhptqyaevlvkrmqaagaqayl
vntgwngtgkrisikdtraiidailngsldnaetftlpmfnlaiptelpgvdtkildprntyaspeqwqekae
tlaklfidnfdkytdtpagaalvaagpkl
>6CHA_F
ivngeeavpgswpwqvslqdktgfhfcggslinenwvvtaaHcgvttsdvvvagefdqgsssekiqklkiakv
fknskynsltinnDitllklstaasfsqtvsavclpsasddfaagttcvttgwgltry
>1UJN_A
mqrlevrepvpypilvgegvlkevpplagpaallfdrrvegfaqevakalgvrhllglpggeaakslevygkv
lswlaekglprnatllvvgggtltdlggfvaatylRgvaylafptttlaivdasvggKtginlpegknlvgaf
hfpqgvyaelralktlplptfkeglveafkhgliagdeallkvedltpqsprleaflaravavKvrvteedpl
EkgkRrllNlghtlgHaleaqtrhalphgmavaygllyaallgralggedllppvrrlllwlspplpplafe
dllpylslhwvvplapgrlvvrplpegllreafaawreelkglgll
>1PP4_A
ttvylagdStmaknggsgtngwgeylasylsatvvndavaGrsarsytregrfeniadvvtagdyvivefgh
Ndggslstdngrtdcsgtgaevcysvydgvnetiltfpaylenaaklftakgakvilssqtpnnpwetgtfvn
sptrfveyaelaaevagveyvdhwsyvdsiyetlgnatvnsyfpiDhtHtspagaevvaeaflkavvctgtsl
ksvltttsfegtcl
>3H6I_D
peqamrerselarkgiaraKsvvalayaggvlfvaenpsrslqKiselydrvgfaaaGkfnefdnlrrggiqf
adtrgyaydrrdvtgrqlanvyaqtlgtifteqakpyevelcvaevahygetkrpelyritydgsiadephfv
vmgGttepianalkesyaenasltdalriavaalragvaslevavldanrprrafrritgsalqall
>1QQ5_A
mikavvfDaygTlfdvqsvadateraypgrgeyitqvwRqkqleyswlralmgryadfwsvtrealaytlgtl
glepdesfladmaqaynrltpypdaaqclaelaplkrailsNgapdmlqalvanagltdsfdavisvdakrvf
KphpdsyalveevlgvtpaevlfvSsNgFDvggaknfgfsvarvarlsqealarelvsgtiapltmfkalrmr
eetyaeapdfvvpalgdlprlvrgmagahlapav
>1MLV_C
mtepslspavqtfwkwlqeegvitaktpvkasvvteglglvalkdisrndvilqvpkrlwinpdavaaseigr
vcselkpwlsvilflirersredsvwkhyfgilpqetdstiywseeelqelqgsqllkttvsvkeyvkneclk
leqeiilpnkrlfpdpvtlddffwafgilrsrafsrlrnenlvvvpmadlinhsagvttedhayevkgaaglf
swdylfslksplsvkageqvyiqYdlnksnaelaldygfiepnenrhaytltleisesdpffddkldvaesng
faqtayfdifynrtlppgllpylrlvalggtdafllesl frdtiwghlelsvsrdneellckavreacksala
gyhttieqdrelkegnldsrlaiavgiregekmvlqqidgifeqkeleldqleyyqerrlkdlglcgengdil
enlyfq
>2AAA_A
lsaaswrtqsiyflltdrfgrtdnsttatcntgneiycggswqgiidhldyiegmgftaiwispiteqlpqdt
adgeayhgywqqkiydvnsnfgtadnlkslsdalhargmylmvdvvpdHmgyagngndvdysvfdpfdsssyf
hpyclitdwdnltmvedcwegdtivslpdldttetavrtiwydwvadlvsnysvdglRiDsvlevqpdffpgy
nkasgvycvgEidnGnpasdcpyqKvldgvlnypiywqllyafesssgsisnlynmiksvasdcsdptllgnf
ienHDnprfakytsdysqaknvlsyiflsdgipivyageeqhyaggkvpynreatwlsgydtsaelytwiatt
nairklaiaadsayityandafytdsntiamakgtsgsqvitvlsnkgssgssytltlsgsgytsgtklieay
tctsvtvdssgdipvpmasglprvllpasvvdssslcg
>1I4N_A
rrlweiveakkkdileidgenlivqrrnhrflevlsgkervkiiaEfKkaspsagdinadasledfirmydel
adaisiltekhyfkgdpafvraarnltcrpilaKdfyidtvqvklassvgadailiiariltaeqikeiyeaa
eelgmdslvEvhsredlekvfsvirpkiigiNtrdldtfeikknvlwellplvpddtvvvaeSgikdprelkd
lrgkvnavlvgtsimkaenprrfleemrawse
>2IAG_A
rtrrpgeppldlgsipwlgyaldfgkdaasfltrmkekhgdiftilvggryvtvlldphsydavvweprtrld
fhayaiflmerifdvqlphyspsdekarmkltllhrelqalteamytnlhavllgdateagsgwhemglldfs
ysfllragyltlygiealprthesqaqdrvhsadvfhtfrqldrllpklargslsvgdkdhmcsvksrlwkll
sparlarrahrskwlesyllhleemgvseemqaralvlqlwatqGnmgpaafwlllfllknpealaavrgele
silwqttlpqkvldstpvldsvlseslrltaapfitrevvvdlampmadgrefnlrrgdrlllfpflspqrdp

```
eiytdpevfkynrflnpdgsekkdfykdgkrlknynmpWgaghnhClgrsyavnsikqfvflvlvhldlelin
adveipefdlsrygfglmqpehdvpvryrirph
>2LIP_A
adnyaatrypiilvhgLtgtdkyagvleywygiqedlqqrgatvyvanlsgfqsddgpngrgeqllayvktvl
aatgatkvnlvghSQggltsryvaavapdlvasvttigtphrgsefadfvqgvlaydptglsstviaafvnvf
giltsssnntnqdalaalktlttaqaatynqnypsaglgapgscqtgaptetvggnthllyswagtaiqptis
vfgvtgatdtstiplvdpanaldpstlalfgtgtvmvnrgsgqnDgvvskcsalygqvlstsykwnHldeinq
llgvrganaedpvavirthanrlklagv
>1DUP_A
mmkkidvkildprvgkefplptyatsgsagldlraclndavelapgdttlvptglaihiadpslaammlprsg
lghkhgivlgnlvgliDsDyqgqlmisvwnrgqdsftiqpgeriaqmifvpvvqaefnlvedfdatdrgeggf
ghsgrq
>1GUF_B
mitaqavlytqhgepkdvlftqsfeidddnlapnevivktlgspvnpSdiNqiqgvYpskpakttgfgttepa
apcgneglfevikvgsnvssleagdwvipshvnfgtwrthalgndddfiklpnpaqskangkpngltinqgat
isvnpltaylmlthyvkltpgkdwfiqnggtsavgkyasqigkllnfnsisvirdrpnldevvaslkelgatq
vitedqnnsrefgptikewikqsggeaklalncvggksstgiarklnnnglmltyggmsfqpvtiptslyifk
nftsagfwvtellknnkelktstlnqiiawyeegkltdaksietlydgtkplhelyqdgvanskdgkqlity
>1J49_A
mtkifayairedekpflkewedahkdveveytdklltpetvalakgadgvvvyqqldyiaetlqaladngitk
mslrnvgvdnidmakakelgfqitnvpvyspnaiaehaaiqaarilrqdkamdekvarhdlrwaptigrevrd
qvvgvvgtghigqvfmqimegfgakvitydifrnpelekkgyyvdslddlykqadvislhvpDvpanvhmind
esiakmkqdvvivnvsRgplvdtdavirgldsgkifgyamDvyegEvgifnedwegkefpdarladliarpnv
lvtpHtafytthavrnmvvkafdnnlelvegkeaetpvkvg
>2QO3_A
esdpiaivsmacrlpggvntpqrlwellreggetlsgfptdrgwdlarlhhpdpdnpgtsyvdkggflddaag
fdaeffgvspreaaamdpqqrllletswelvenagidphslrgtatgvflgvakfgygedtadvegysvtgva
pavasgrisytmglegpsisvdtacssslvalhlaveslrkgessmavvggaavmatpgvfvdfsrqralaad
grskafgagadgfgfsegvtlvllerlsearrnghevlavvrgsalnqdgasnglsapsgpaqrrvirqales
cglepgdvdaveahgtgtalgdpieanalldtygrdrdadrplwlgsvksnightqaaagvtgllkvvlalrn
gelpatlhveeptphvdwssggvallagnqpwrrgertrraavsafgisgtnahviveeapehdgrpvplvvs
arstaalraqaaqiaellerpdadlagvglglattrarhehraavvastreeavrglreiaagaatadavveg
vtevdgrnvvflfpgQgsqwagmgaellssspvfagkiracdesmapmqdwkvsdvlrqapgapgldrvdvvq
pvlfavmvslaelwrsygvepaavvghSQgeiaaahvagaltledaaklvvgRsrlmrslsgeggmaavalge
aavrerlrpwqdrlsvaavngprsvvvsgepgalrafsedcaaegirvrdidvdyasHspqiervreellett
gdiaprparvtfhstvesrsmdgteldarywyrNlretvrfadavtrlaesgydafievsphpvvvqaveeav
eeadgaedavvvgslhrdggdlsaflrsmatahvsgvdirwdvalpgaapfalptypfqrkrywlq
>2AMG_A
dqagkspnavryhggdeiilqgfhwnvvreapndwynilrqqaatiaadgfsaiwmpvpwrdfsswsdgsksg
ggegyfwhdfnkngrygsdaqlrqaasalggagvkvlydvvpnhmnrgypdkeinlpagqgfwrndcadpgny
pndcddgdrfiggdadlntghpqvygmfrdeftnlrsqygaggfrfDfvrgyapervnswmtdsadnsfcvgE
lwkgpseypnwdwrntaswqqiikdwsdrakcpvfdfalkermqngsiadwkhglngnpdprwrevavtfvdn
hDtgyspgqnggqhhwalqdglirqayayiltspgtpvvywdhmydwgygdfirqliqvrraagvradsaisf
hsgysglvatvsgsqqtlvvalnsdlgnpgqvasgsfseavnasngqvrvwrs
>1Q3N_A
mkqkvvsigdinvandlpfvlfggmnvlesrdlamricehyvtvtqklgipyvfkasfdkanrssihsyrgpg
leegmkifqelkqtfgvkiitdvhepsqaqpvadvvdviqlpaflarqtdlveamaktgavinvkkpqfvspg
qmgnivdkfkeggnekvilcdrganfgydnlvvdmlgfsimkkvsgnspvifdvtHalqcrdpfgaasggrra
qvaelaragmavglaglfieahpdpehakcdgpsalplaklepflkqmkaiddlvkgfeeldtsk
>1WAO_4
galkraeelktqandyfkakdyenaikfysqaielnpsnaiyygnrslaylrtecygyalgdatraieldkky
ikgyyrraasnmalgkfraalrdyetvvkvkphdkdakmkyqecnkivkqkaferakrsvvdsldiesmtied
eysgpkledgkvtisfmkelmqwykdqkklhrkcayqilvqvkevlsklstlvettlketekitvcgdthgqf
ydllnifelnglpsetnpyifngdfvDRgsfsveviltlfgfkllypdhfhllrgNHetdnmnqiygfegevk
```

akytaqmyelfsevfewlplaqcingkvlimhgglfsedgvtlddirkiernrqppdsgpmcdllwsdpqpqn
grsiskRgvscqfgpdvtkafleennldyiirsHevkaegyevahggrcvtvfsapnycdqmgnkasyihlqg
sdlrpqfhqftavphpnvkpmayantll
>1NID_A
aagaapvdistlprvkvdlvkppfvhahdqvaktgprvveftmtieekklvidregteihamtfngsvpgplm
vvhendyvelrlinpdtntllhniDfhaatgalgggaltqvnpgeettlrfkatkpgvfvyhcapegmvpwhv
tsgmngaimvlprdglkdekgqpltydkiyyvgeqdfyvpkdeagnykkyetpgeayedavkamrtltpthiv
fngavgaltgdhaltaavgervlvvhsqanrdtrpHligghgdyvwatgkfrnppdldqetwlipggtagaaf
ytfrqpgvyayvnhnlieafelgaaghfkvtgewnddlmtsvvkpasm
>2QLB_C
tyqynmnfeklgkciiinnknfdkvtgmGvrngtdkdaealfkcfrslgfdvivyndcscakmqdllkkasee
dhtnaacfacillsHGeenviygkdgvtpikdltahfrgdrcktllEkpklffiqaCRgtelddgiq
>8PCH_A
yppsmdwrkkgnfvspvknQgscgsCwtfsttgalesavaiatgkmlslaeqqlvdcaqnfnnhgcqgglpsq
afeyirynkgimgedtypykgqddhckfqpdkaiafvkdvanitmndeeamveavalynpvsfafevtndflm
yrkgiysstschktpdkvnHavlavgygeengipywivkNswgpqwgmngyfliergknmcglaacasypipl
v
>3N3B_B
risainwnkisddkdlevwnrltsnfwlpekvplsndipawqtltvveqqltmrvftgltlldtlqnvigaps
lmpdaltpheeavlsnisfmeavharsYssifstlcqtkdvdaayawseenaplqrkaqiiqqhyrgddplkk
kiasvflesflfysgfwlpmyfssrgkltntadlirliirdeavhgyyigykyqknmekislgqreelksfaf
dlllelydnelqytdelyaetpwaddvkaflcynankalmnlgyeplfpaemaevnpailaalsp
>1ABR_A
edrpikfstegatsqsykqfiealrerlrgglihdipvlpdpttlqernryitvelsnsdtesievgidvtna
yvvayragtqsyflrdapssasdylftgtdqhslpfygtygdlerwahqsrqqiplglqalthgisffrsggn
dneekartlivi iqmvaEaaRfryisnrvrvsiqtgtafqpdaamislennwdnlsrgvqesvqdtfpnqvtl
tnirnepvivdslshptvavlalmlfvcnppn
>1QK2_B
tatysgnpfvgvtpwanayyasevsslaipsltgamataaaavakvpsfmwldtldktplmeqtladirtank
nggnyagqfvvYdlpdRDcaalasngeysiadggvakyknyidtirqivveysdirtllviepDslanlvtnl
gtpkcanaqsayleci nyavtqlnlpnvamyldaghagwlgwpanqdpaaqlfanvyknasspralrglatnv
anyngwnitsppsytqgnavyneklyihaigpllanhgwsnaffitdqgrsgkqptgqqqwgdwcnvigtgfg
irpsantgdslldsfvwvkpggecDgtsdssaprfdshcalpdalqpapqagawfqayfvqlltnanpsfl
>1APY_A
ssplplvvntwpfknateaawralasggsaldavesgcamcereqcdgSvgfggspdelgettldamimdgtt
mdvgavgdlrriknaigvarkvlehtthtllvgesattfaqsmgfinedlstsasqalhsdwlarncqpnywr
nvipdpskycgpykpp
>1I9A_A
mqtehvillnaqgvptgtlekyaahtadtrlhlafsswlfnakgqllvtrralskkawpgvwtnsvCghpqlg
esnedavirrcryElgveitppesiypdfryratdpsgivenEvcpvfaarttsalqinddevmdyqwcdlad
vlhgidatpwafspWmvmqatnrearkrlsaftqlk
>1B9H_A
mnarkapefpawpqyddaernglvraleqgqwwrmggdevnsferefaahhgaahalavtngthalelalqvm
gvgpgtevivpaftFisssqaaqrlgavtvpvdvdaatynldpeavaaavtprtkvimpvhmaglmadmdala
kisadtgvpllqDaahahgarwqgkrvgeldsiatfsfqngKlmtageggavvfpdgetekyetaflrhscgr
prddrryfhkiagsnmrlnefsasvlraqlarldeqiavrderwtllsrllgaidgvvpqggdvradrnshym
amfripglteerrnalvdrlveaglpafaafraiyrtdafwelgapdesvdaiarrcpntdaissdcvwlhhr
vllagepelhataeiiadavara
>1K4L_A
mpstdsipksnfdaipdviqafkngefvvvlddpsreneaDliiaaesvtteqmafmvrhssgliCapltper
ttaldlpqmvthnadprgtaYtvsvDaehpstttgisahdralacrmlaapdaqpshfrrpgHvfplravagg
vrarrghteagvelcrlagkrpvavisEivddgqevegravraapgmlrgdecvafarrwglkvctiedmiah
vektegkletngsg
>1CJY_A

msfidpyqhiivehqyshkftvvvlratkvtkgafgdmldtpdpyvelfisttpdsrkrtrhfnndinpvwne
tfefildpnqenvleitlmdanyvmdetlgtatftvssmkvgekkevpfifnqvtemvlemslevcscpdlrf
smalcdqektfrqqrkehiresmkkllgpknseglhsardvpvvailgsgGGframvgfsgvmkalyesgild
catyvaglSgstwymstlyshpdfpekgpeeineelmknvshnpllltpqkvkryveslwkkkssgqpvtft
difgmligetlihnrmnttlsslkekvntaqcplplftclhvkpdvselmfadwvefspyeigmakygtfmap
dlfgskffmgtvvkkyeenplhflmgvwgsafsilfnrvlgvsgsqsrgstmeeelenittkhivsndssdsd
deshepkgtenedagsdyqsdnqaswihrmimalvsdsalfntregragkvhnfmlglnlntsyplsplsdfa
tqdsfdddeldaavadpdeferiyepldvkskkihvvDsgltfnlpyplilrpqrgvdliisfdfsarpsdss
ppfkelllaekwakmnklpfpkidpyvfdreglkecyvfkpknpdmekdcptiihfvlaninfrkykapgvpr
eteeekeiadfdifddpespfstfnfqypnqafkrlhdlmhfntlnnidvikeamvesieyrrqnpsrcsvsl
snvearrffnkeflskpka
>1HR9_A
artdnfklsslanglkvatsntpghfsalglyidagsrfegrnlkgcthilDrlafkstehvegramaetlel
lggnyqctssrenlmyqasvfnqdvgkmlqlmsetvrfpkiteqelqeqklsaeyeidevwmkpelvlpellh
taaysgetlgsplicprglipsiskyylldyrnkfytpentvaafvgvphekaleltgkylgdwqsthppitk
kvaqytggescippapvfgnlpelfhiqigfeglpidhpdiyalatlqtllgggggpgkgmysrlythvlnqy
yfvencvafnhsysdsgifgislscipqaapqaveviaqqmyntfankdlrltedevsraknqlksslln mnle
sklveledmgrqvlmhgrkipvnemiskiedlkpddisrvaemiftgnvnnagngkgratvvmqgdrgsfgdv
envlkayglgnsss
>1OZH_A
mdkqypvrqwahgadlvvsqleaqgvrqvfgipgakidkvfdslldssiriipvrheanaafmaaavgritgk
agvalvtsgpgcsnlitgmatansegdpvvalggavkradkakqvhqsmdtvamfspvtkyaievtapdalae
vvsnafraaeqgrpgsafvslpqdvvdgpvsgkvlpasgapqmgaapddaidqvakliaqaknpifllglmas
qpenskalrrlletshipvtstyqaagavnqdnfsrfagrvglfnnqagdrllqladlvicigyspveyepam
wnsgnatlvhidvlpayeernytpdvelvgdiagtlnklaqnidhrlvlspqaaeilrdrqhqrelldrrgaq
lnqfalhplrivramqdivnsdvtltvdMgsfhiwiarylytfrarqvmisngqqtMgvalpwaigawlvnpe
rkvvsvsgdggflqssmeletavrlkanvlhliwvdngynmvaiqeekkyqrlsgvefgpmdfkayaesfgak
gfavesaealeptlraamdvdgpavvaipvdyrdnpllmgqlhlsqilehhhhhh
>1I8T_A
mydyiivgsglfgavcanelkklnkkvlviekrnhiggnaytedcegiqihkygahifhtndkyiwdyvndlv
efnrftnsplaiykdklfnlpfnmntfhqmwgvkdpqeaqniinaqkkkygdkvpenleeqaislvgedlyqa
likgytekqwgrsakelpafiikRipvrftfdnnyfsdryqgipvggytkliekmlegvdvklgidflkdkds
laskahriiytgpidqyfdyrfgaleyrslkfeterhefpnfqgnavinftdanvpytRiiehkhfdyvetkh
tvvtkEyplewkvgdepyypvndnknmelfkkyrelasredkvifggrlaeykyyDmhqvisaalyqvknims
td
>1F3L_A
dlqededgvyfssyghygiheemlkdkvrtesyrdfiyqnphifkdkvvldvgcgtgilsmfaakagakkvia
vdqseilyqamdiirlnkledtivlikgkieevslpvekvdviisEwmgyfllfEsmldsvlyakskylakgg
svypdictislvavsdvskhadriafwddvygfnmscmkkavipeavvevvdhktlisdpcdikhidchttsi
sdlefssdftlrttktamctavagyfdiyfeknchnrvvfstgpqstkthwkqtifllekpfpvkagealkgk
itvhknkkdprslivtltlnsstqtyslq
>1DHF_A
vgslncivavsqnmgigkngdLpwpplrnEfryfqrmtttssvegkqnlvimgkktwfsipeknrplkgrinl
vlsrelkeppqgahflsrslddalklteqpelankvdmvwivggssvykeamnhpghlklfvtrimqdfesdt
ffpeidlekykllpeypgvlsdvqeekgikykfevyeknd
>1AGI_A
aqddyryihfltqHydakpkgrndeycfnmmknrrltrpcKdrntfihgnkndikaicedrngqpyrgdlris
ksefqitickhkggssrppcrygatedsrvivvgcenglpvHFdesfitprh
>1GCI_A
aqsvpwgisrvqapaahnrgltgsgvkvavlDtgisthpdlnirggasfvpgepstqdgngHgthvagtiaal
nnsigvlgvapsaelyavkvlgasgsgsvssiaqglewagnngmhvanlslgspspsatleqavnsatsrgvl
vvaasgNsgagsisyparyanamavgatdqnnnrasfsqygagldivapgvnvqstypgstyaslngtSmatp
hvagaaalvkqknpswsnvqirnhlkntatslgstnlygsglvnaeaatr
>1BO1_A

mssnctsttavavaplsasktktkkkhfvcqkvklfrasepilsvlmwgvnhtinelsnvpvpvmlmpddfka
yskikvdnhlfnkenlpsrfkfkeycpmvfrnlrerfgiddqdyqnsvtrsapinsdsqgrcgtrflttydrr
fviKtvssedvaemhnilkkyhqfivechgntllpqflgmyrltvdgvetymvvtrnvfshrltvhrkydlkg
stvareasdkekakdlptfkdndflnegqklhvgeeskknfleklkrdveflaqlkimDysllvgihdvdrae
qeemeveeraedeecendgvggnllcsygtppdspgnllsfprffgpgefdpsvdvyamkshesspkkevyfm
aiidiltpydtkkaahaaktvkhgagaeistvnpeqyskrfnefmsnilt
>1A26_A
altvsagtksklakpiqdlikmifdvesmkkamvefeidlqkmplgklskrqiqsaysilnevqqavsdggse
sqildlsnrfytliphdfgmkkppllsnleyiqakvqmldnlldievaysllrggnedgdkdpidinyeklrt
dikvvdkdseeakiikqyvknthaathnaydlkvveifrieregesqrykpfkqlhnrqllwhgsrttnfagi
lsqglriappeapvtgymfgkgiyfadmvsksanYchtsqadpiglillgevalgnmyelknashitklpkgk
hsvkglgktapdptatttldgvevplgngistgindtcllynEyivydvaqvnlkyllklkfnyktslw
>1PFK_A
mikkigvltsgGdapgmnaairgvvrsalteglevmgiydgylglyedrmvqldrysvsdminrggtflgsaR
fpefrdeniravaienlkkrgidalvviggdgsymgamrltemgfpciglpgTiDndikgtdytigfftalst
vveaidrlrdtssshqrisvvevmgRycgdltlaaaiaggcefvvvpevefsredlvneikagiakgkkhaiv
aitehmcdvdelahfieketgretratvlghiqrggspvpydrilasrmgayaidlllagyggrcvgiqneql
vhhdiidaienmkrpfkgdwldcakkly
>1EEJ_A
ddaaiqqtlakmgikssdiqpapvagmktvltnsgvlyitddgkhiiqgpmydvsgtapvnvtnkmllkqlna
lekemivykapqekhvitvftditCgYChklheqmadynalgitvrylafpRqgldsdaekemkaiwcakdkn
kafddvmagksvapascdvdiadhyalgvqlgvsgtpavvlsngtlvpgyqppkemkefldehqkmtsgk
>1F8M_A
asvvgtpksaeqiqqewdtnprwkdvtrtysaedvvalqgsvveehtlarrgaevlweqlhdlewvnalgalt
gnmavqqvraglkaiylsgwqvagdanlsghtypdqslypansvpqvvrrinnalqradqiakiegdtsvenw
lapivadgeagfggalnvyelqkaliaagvagsHwedqlasekkCgHlggkvliptqqhirtltsarlaadva
dvptvviaRtdaeaatlitsdvderdqpfitgertregfyrtkngiepciarakayapfadliwmetgtpdle
aarqfseavkaeypdqmlayncspsfnwkkhlddatiakfqkelaamgfkfqfitlagfhalnysmfdlaygy
aqnqmsayvelqerefaaeergytatkhqrevgagyfdriattvdpnssttaltgsteegqf
>1HXQ_A
mtqfnpvdhphrrynpltgqwilvsphrakrpwqgaqetpakqvlpahdpdcflcagnvrvtgdknpdytgty
vftndfaalmsdtpdapeshdplmrcqsargtsrvicfspdhsktlpelsvaalteivktwqeqtaelgktyp
wvqvfenkgaamgCsnpHpHgQiwansflpneaeredrlqkeyfaeqkspmlvdyvqreladgsrtvvetehw
lavvpywaawpfetlllpkahvlritdltdaqrsdlalalkkltsrydnlfqcsfpysmgwhgapfngeenqh
wqlhahfypplirsatvrkfmvgyemlaetqrdltaeqaaerlravsdihfresgv
>1PJ5_A
mastpriviigagivgtnladelvtrgwnnitvldqgplnmpggstshapglvfqtnpsktmasfakytvekl
lsltedgvscfnqvgglevattetrladlkrklgyaaawgiegrllspaecqelyplldgenilgglhvpsdg
lasaaravqllikrtesagvtyrgsttvtgieqsggrvtgvqtadgvipadivvscagfwgakigamigmavp
llplaHqyvkttpvpaqqgrndqpngarlpilrhqdqdlYyrehgdrygigsyahrpmpvdvdtlgayapetv
sehhmpsrldftledflpaweatkqllpaladseiedgfngifsftpdggpllgeskeldgfyvaeavwvths
agvakamaellttgrsetdlgecditrfedvqltpeyvsetsqqnfveiydvlhplqprlsprnlrvspfhar
hkelgaffleaggwerpywfeanaallkempaewlppardawsgmfsspiaaaeawktrtavamydmtplkrl
evsgpgalkllqelttadlakkpgavtytllldhaggvrsDitvarlsedtfqlgangnidtayferaarhqt
qsgsatdwvqvrdttggtcciglwgplardlvskvsdddftndglkyfraknvviggipvtamrlsyvgelgw
elytsadngqrlwdalwqagqpfgviaagraafsslrlekgyrswgtdmttehdpfeaglgfavkmakesfig
kgalegrteeasarrlrcltiddgrsivlgkepvfykeqavgyvtsaaygytvakpiaysylpgtvsvgdsvd
ieyfgrritatvtedplydpkmtrlrg
>1BGL_A
tmitdslavvlqrrdwenpgvtqlnrlaahppfaswrnseeartdrpsqqlrslngewrfawfpapeavpesw
lecdlpeadtvvvpsnwqmhgydapiytnvtypitvnppfvptenptgcysltfnvdeswlqegqtriifdgv
nsafhlwcngrwvgygqdsrlpsefdlsaflragenrlavmvlrwsdgsyledqdmwrmsgifrdvsllhkpt
tqisdfhvatrfnddfsravleaevqmcgelrdylrvtvslwqgetqvasgtapfggeiiderggyadrvtlr
lnvenpklwsaeipnlyravvelhtadgtlieaeacdvgfrevrienglllllngkpllirgvnrhehhplhgq

vmdeqtmvqdillmkqnnfnavrcshypnhplwytlcdryglyvvdeaniethgmvpmnrltddprwlpamse
rvtrmvqrdrnhpsviiwslgnEsghganhdalyrwiksvdpsrpvqyegggadttatdiicpmyarvdedqp
fpavpkwsikkwlslpgetrplilcEyahamgnslggfakywqafrqyprlqggfvwdwvdqslikydengnp
wsayggdfgdtpndrqfcmnglvfadrtphpalteakhqqqffqfrlsgqtievtseylfrhsdnellhwmva
ldgkplasgevpldvapqgkqlielpelpqpesagqlwltvrvvqpnatawseaghisawqqwrlaenlsvtl
paashaiphlttsemdfcielgnkrwqfnrqsgflsqmwigdkkqlltplrdqftrapldndigvseatridp
nawverwkaaghyqaeaallqctadtladavlittahawqhqgktlfisrktyridgsgqmaitvdvevasdt
phparliglncqlaqvaervnwlglgpqenypdrltaacfdrwdlplsdmytpyvfpsenglrcgtrelnygph
qwrgdfqfnisrysqqqlmetshrhllhaeegtwlnidgfhmgiggddswspsvsaefqlsagryhyqlvwcq
k
>3IOF_A
agmsltqvsgpvyvvednyyvqensmvyfgakgvtvvgatwtpdtarelhklikrvsrkpvlevintnyhtdr
aggnaywksigakvvstrqtrdlmksdwaeivaftrkglpeypdlplvlpnvvhdgdftlqegkvrafyagpa
htpdgifvyfpdeqvlyggcilkeklgNlsfadvkaypqtlerlkamklpiktvigghdsplhgpelidhyea
likaapqs
>1S4D_L
aglpalekgsvwlvgagpgdpglltlhaanalrqadvivhDalvnedclklarpgavlefagKggkpskqrdi
slrlvelaragnrvlrlkggdpfvfgrggeealtlvehqvpfrivpgitagigglayagipvthrevnhavtf
ltghvpdrinwqgiasgspvivmyMamkhigaitanliaggrspdepvafvcnaatpqqavlettlaraeadv
aaagleppaivvvgevvrlraaldwiga
>1UA7_A
psiksgtilhawnwsfntlkhnmkdihdagytaiqtspinqvkegnqgdksmsnwywlyqptsyqignrylgt
eqefkemcaaaeeygikvivdavinHttfdyaaisnevksipnwthgntqiknwsdrwdvtqnsllglydwnt
qntqvqsylkrfleralndgadgfRfDaakhielpddgsygsqfwpnitNtsaefqygEiLqdsasrdaayan
ymdvtasnyghsirsalknrnlgvsnishyasdvsadklvtwvesHDtyanddeestwmsdddirlgwavias
rsgstplffsrpegggngvrfpgksqigdrgsalfedqaitavnrfhnvmagqpeelsnpqgnnqifmnqrgs
hgvvlanagsssvsintatklpdgrydnkagagsfqvndgkltgtinarsvavlypd
>1IT4_A
apadkpqvlasftqtsassqnawlaanrnqsawaayefdwstdlctqapdnpfgfpfntacarHdfgyrnyka
agsfdanksriDsafyedmkrvctgytgekntacnstawtyyqavkifg
>1AUO_A
mteplilqpakpadacviwlhgLgadrydfmpvaealqeslltrfvlpqaptrpvtinggyempswydikam
sparsisleelevsakmvtdlieaqkrtgidasriflagfSQggavvfhtafinwqgplggvialstyaptfg
delelsasqqripalclhgqyDdvvqnamgrsafehlksrgvtvtwqeypmgHevlpqeihdigawlaarlg
>3BIF_A
cptlivmvglpargktyiskkltrylnfigvptrefnvgqyrrdmvktyksfefflpdneeglkirkqcalaa
lndvrkflseegghvavfdatnttrerramifnfgeqngykt ffvesicvdpeviaanivqvklgspdyvnrd
sdeatedfmrriecyensyesldeeqdrdlsyikimdvgqsyvvnrvadhiqsrivyylmnihvtprsiylcR
HgeselNlkgriggdpglsprgrefskhlaqfisdqnikdlkvftsqmkRtiqtaealsvpyeqfkvlnEida
gvceemtyeeiqdhyplefalrdqdkyryrypkgesyedlvqrlepvimelerqenvlvicHqavmrcllayf
ldkaaeelpylkcplhtvlkltpvaygckvesiflnvaavnthrdrpqnvdisrpseealvtvpahq
>1ZIO_A
mnlvlmglpgagKgtqaekivaaygiphistgdmfraamkegtplglqakqymdrgdlvpdevtigivrerls
kddcqngflldgfprtvaqaealetmladigrkldyvihidvrqdvlmerltgRricrncgatyhlifhppak
pgvcdkcggelyqRaDDneatvanRlevnmkqmkplvdfyeqkgylrningeqdmekvfadirellgglar
>3OZU_A
mltqktkdivkatapvlaehgydiikcfyqrmfeahpelknvfnmahqeqgqqqqalaravyayaeniedpns
lmavlknianhkhaslgvkpeqypivgehllaaikevlgnaatddiisawaqaygnladvlmgmeselyersae
qpggwkgwrtfvirekrpesdvitsfilepadggpvvnfepgqYtsvaidvpalglqqirqYSlsdmpngrsy
risvkregggpqppgyvsnllhdhvnvgdqvklaapygsfhidvdaktpivlisggvgltpmvsmlkvalqap
prqvvfvhgarnsavhamrdrlreaaktyenldlfvfydqplpedvqgrdydypglvdvkqieksillpdady
yiCgpipfmrmqhdalknlgihearihyEvfgpdlfae
>2FGZ_A

```
dvvvrlpddafraafgvaladahwvdkttllwpggenkpivrlyyshsskvaadsngefsdkyvkltpttvnq
qvsmrfphlasypafklpddvnvdellqgetvaiaaesdgilssatqvqtagvlddtyaaaaealsygaqltd
sgvtfrvwaptaqqvelviysadkkviashpmtrdsasgawswqggsdlkgafyryamtvyhpqsrkveqyev
tdpyahslstnseysqvvdlndsalkpegwdgltmphaqktkadlakmtiheshirdlsawdqtvpaelrgky
laltaqesnmvqhlkqlsasgvthiellpvfdlatvnefsdkvadiqqpfsrlcevnsavkssefagycdsgs
tveevltqlkqndskdnpqvqalntlvaqtdsynwgydpfhytvpegsyatdpegtarikefrtmiqaikqdl
gmnvimdvvynhtnaagptdrtsvldkivpwyyqrlnettgsvesatccsdsapehrmfakliadslavwttd
ykidgfrfDlmlyhpkaqilsawerikalnpdiyffgegwdsnqsdrfeiasqinlkgtgigtfsDrlrdavr
gggpfdsgdalrqnqgvgsgagvlpnelttlsddqarhladltrlgmagnladfvlidkdgavkrgseidyng
apggyaadptevvnyvskhdnqtlwdmisykaaqeadldtrvrmqavslatvmlgqgiafdqqgsellrsksf
trdsydsgdwfnrvdyslqdnnynvgmprssddgsnydiiarvkdavatpgetelkqmtafyqeltalrkssp
lftlgdgatvmkrvdfrntgadqqtgllvmtiddgmqagasldsrvdgivvainaapesrtlqdfagtslqls
aiqqaagdrslasgvqvaadgsvtlpawsvavlelpqgesqgaglpvssk
>1KAN_B
mngpiimtreermkivheikerildkygddvkaigvygslgrqtdgpysdiemmcvmsteeaefshewttgew
kvevnfyseeilldyasqvesdwplthgqffsilpiydsggylekvyqtaksveaqkfhdaicaliveelfEy
agKwrnirvqgpttflpsltvqvamagamliglhhricyttsasvlteavkqsdlpsgydhlcqfvmsgqlsd
seklleslenfwngiqewterhgyivdvskripf
>1CT9_A
Asifgvfdiktdavelrkkalelsrlmrhrgpdwsgiyasdnailaherlsivdvnagaqplynqqkthvlav
NGeiynhqalraeygdryqfqtgsdcevilalyqekgpefldlqgmfafalydsekdayligrdhlgiiply
mgydehgqlyvasemkalvpvcrtikefpagsylwsqdgeirsyyhrdwfdydavkdnvtdknelrqaledsv
kshlmsdvpygvllsggldssiisaitkkyaarrvedqerseawwpqlhsfavglpgspdlkaaqevanhlgt
vhheihftvqegldairdviyhietydvttirastpmylmsrkikamgikmvlsgegsdevfggylyfhkapn
akelheetvrkllalhmydcarankamsawgvearvpfldkkfldvamrinpqdkmcgngkmekhilrecfea
ylpasvawrqkeqfsdgvgyswidtlkevaaqqvsdqqletarfrfpyntptskeaylyreifeelfplpsaa
ecvpggpsvacssakaiewdeafkkmddpsgravgvhqsayk
>1I7D_A
mrlfiaEKpslaraiadvlpkphrkgdgfiecgngqvvtwcighlleqaqpdaydsryarwnladlpivpekw
qlqprpsvtkqlnvikrflheaseivhagdpdregqllvdevldylqlapekrqqvqrclindlnpqaverai
drlrsnsefvplcvsalararadwlyginmtraytilgrnagyqgvlsvgrvqtpvlglvvrrdeeienfvak
dffevkahivtpaderftaiwqpseacepyqdeegrllhrplaehvvnrisgqpaivtsyndkresesaplpf
slsalqieaakrfglsaqnvldicqklyethklitFpRsdcrylpeehfagrhavmnaisvhapdllpqpvvd
pdirnrcwddkkvdahhaiiptarssainlteneakvynliarqylmqfcpdavfrkcvieldiakgkfvaka
rflaeagwrtllgskerdeendgtplpvvakgdellcekgevverqtqpprhftdatllsamtgiarfvqdkd
lkkilratdglgteatragiiellfkrgfltkkgryihstdagkalfhslpematrpdmtahwesvltqisek
qcryqdfmqplvgtlyqlidqakrtpvrqfrgivapgsggsadkkkaaprkrsakksppadevgsgaiahhhh
hh
>1IT8_B
kmlkfeikardgagrigklevngkkietpaimpvvnpkqmvvepkelekmgfeiiitnsyiiykdeelrrkal
elgihrmldyngiievDsgsfqlmkygsievsnreiiefqhrigvdigtfldiptppdapreqavkeleitls
rareaeeikeipmnatiqgstytdlrryaarrlssmnfeihpiggvvpllesyrfrdvvdivisskmalrpdr
pvhlfgaghpivfalavamgvdlfdsasyalyakddrymtpegtkrldeldyfpcscpvcskytpqelrempk
eertrllalhnlwvikeeikrvkqaikegelwrlvderarshpklysaykrllehytfleefepitkksalfk
isneslrwpvvrrakeraksinerfgelvehpifgrvsrylsltypfaqseaeddfkiekptkedaikyvmai
aeyqfgegasrafddakvelsktgmprqvkvngkrlatvraddglltlgiegakrlhrvlpyprmrvvvnkea
epfarkgkdvfakfvifadpgirpydevlvvnendellatgqallsgremivfqygravkvrkgve
>2ACY_A
aegdtlisvdyeifgkvqgvffRkytqaegkklglvgwvqNtdqgtvqgqlqgpaskvrhmqewletkgspks
hidrasfhnekvivkldytdfqivk
>1ZJ8_B
rnegqwalghreplnaneelkkagnpldvrerieniyakqgfdsidktdlrgrfrwwglytqreqgydgtwtg
ddnidkleakyfmmRvrcdggalsaaalrtlgqistefardtadisdrqnvqyhwievenvpeiwrrlddvgl
qttelacgdcpRvvlgsplagesldevldptwaieeivrryigkpdfadlprKyKtaisglqdvaheindvafi
```

gvnhpehgpgldlwvggglstnpmlaqrvgawvplgevpevwaavtsvfrdygyrrlrakarlkflikdwgia
kfrevleteylkrplidgpapepvkhpidhvgvqrlknglnavgvapiagrvsgtiltavadlmaragsdrir
ftpyqklvildipdallddliagldalglqsrpshwrrnlmacsgiefcklsfaetrvraqhlvpelerrled
insqldvpitvningcpnsCariqiadigfkgqmiddghggsvegfqvhlgghlgldagfgrklrqhkvtsde
lgdyidrvvrnfvkhrsegerfaqwviraeeddlr
>1MBB_A
mnhslkpwntfgidhnaqhivcaedeqqllnawqyataegqpvlilgegsnvlfledyrgtviinrikgieih
depdawylhvgagenwhrlvkytlqegmpglenlalipgcvgsspiqnigaygvelqrvcayvdsvelatgkq
vrltakecrfgyRdsifkheyqdrfaivavglrlpkewqpvltygdltrldpttvtpqqvfnavchmrttklp
dpkvngnagSffknpvvsaetakallsqfptapnypqadgsvklaagwlidqcqlkgmqiggaavhrqqalvl
inednaksedvvqlahhvrqkvgekfnvwlepEvrfigasgevsavetis
>1GET_A
mtkhydyiaigggsggiasinraamygqkcalieakelggtCvnvgCvpkkvmwhaaqireaihmygpdygfd
ttinkfnwetliasrtayidrihtsyenvlgknnvdvikgfarfvdaktlevngetitadhiliatggrpshp
dipgveygidsdgffalpalpervavvgagyiavelagvinglgakthlfvrkhaplrsfdpmisetlvevmn
aegpqlhtnaipkavvkntdgsltleledgrsetvdcliwaigrepandninleaagvktnekgyivvdkyqn
tniegiyavgdntgaveltpvavaagrrlserlfnnkpdehldysniptvvfshppigtvgltepqareqygd
dqvkvykssftamytavtthrqpcrmklvcvgseekivgihgigfgmdemlqgfavalkmgatkkdfdntvai
HptaaEefvtmr
>1JCH_A
msggdgrghntgahstsgninggptglgvgggasdgsgwssennpwgggsgsgihwgggsghgngggngnsgg
gsgtggnlsavaapvafgfpalstpgagglavsisagalsaaiadimaalkgpfkfglwgvalygvlpsqiak
ddpnmmskivtslpadditespvsslpldkatvnvnvrvvddvkderqnisvvsgvpmsvpvvdakpterpgv
ftasipgapvlnisvnnstpavqtlspgvtnntdkdvrpafgtqggntrdavirfpkdsghnavyvsvsdvls
pdqvkqrqdeenrrqqewdathpveaaernyeraraelnqanedvarnqerqakavqvynsrkseldaanktl
adaiaeikqfnrfahdpmagghrmwqmaglkaqraqtdvnnkqaafdaaakeksdadaalssamesrkkkedk
krsaennlndeknkprkgfkdyghdyhpapktenikglgdlkpgipktpkqngggkrkrwtgdkgrkiyewDs
qHgelEgyrasdgqhlgsfdpktgnqlkgpdpkRnikkyl
>1L9X_D
glvprakkpiigilmqkcrnkvmknygryyiaasyvkylesagarvvpvrldltekdyeilfksingilfpgg
svdlrrsdyakvakifynlsiqsfddgdyfpvwgtClgfeelsllisgeclltatdtvdvamplnftggqlhs
rmfqnfptelllslavepltanfhkwslsvknftmneklkkffnvlttntdgkiefistmegykypvygvqwH
pekapyewknldgishapnavktafylaeffvnearknnhhfkseseeekaliyqfspiytgnissfqqcyif
d
>1KNP_A
mntlpehscdvliigsgaaglslalrladqhqvivlskgpvtegstfyaqggiaavfdetdsidshvedtlia
gagicdrhavefvasnarscvqwlidqgvlfdthiqpngeesyhltregghshrrilhaadatgrevettlvs
kalnhpnirvlertnavdlivsdkiglpgtrrvvgawvwnrnketvetchakavvlatggaskvyqyttnpdi
ssgdgiamawragcrvanlefnqfhptalyhpqarnflltealrgegaylkrpdgtrfmpdfdergelapRdi
varaidhemkrlgadcmfldishkpadfirqhfpmiyekllglgidltqepvpivpaaHytcggvmvddhgrt
dveglyaigevsytglhganLmasnslleclvygwsaaeditrrmpyahdistlppwdesrvenpdervviqh
nwhelrlfmwdyvgivrttkrleralrritmlqqeideyyahfrvsnnllelrnlvqvaelivrcammrkesr
glhftldypellthsgpsilspgnhyinr
>1GA8_A
mdivfaaddnyaaylcvaaksveaahpdteirfhvldagiseanraavaanlrggggnirfidvnpedfagfp
lnirhisittyarlklgeyiadcdkvlyldidvlvrdsltplwdtdlgdnwlgasiDlfverqegykqkigma
dgeyyfNagvllinlkkwrrhdifkmssewveqykdvmqyqdQdilnglfkggvcyansrfnfmptnyafman
wfasrhtdplyrdrtntvmpvavshycgpakpwhrdctawgaerftelagslttvpeewrgklavphrmfstk
rmlqrwrrklsarflrkiy
>1DHK_A
yapqtqsgrtsivhlfewrwvdialecerylgpkgfggvqvsppnenvvvtnpsrpwweryqpvsyklctrsg
nenefrdmvtrcnnvgvriyvdavinHmcgsgaaagtgttcgsycnpgsrefpavpysawdfndgkcktasgg
iesyndpyqvrdcqlvglldlalekdyvrsmiadylnklidigvagfRiDaskhmwpgdikavldklhnlntn
wfpagsrpFifQEvidlggeaiqsseyfgngrvtefkygaklgtvvrkwsgekmsylknwgegwgfmpsdral

vfvdnHDnqrghgaggasiltfwdarlykvavgfmlahpygftrvmssyrwarnfvngedvndwigppnnngv
ikevtinadttcgndwvcehrwreirnmvwfrnvvdgqpfanwwdngsnqvafgrgnrgfivfnnddwqlsst
lqtglpggtycdvisgdkvgnsctgikvyvssdgtaqfsisnsaedpfiaihaeskl
>1KIM_A
mptllrvyidgphgmgKttttqlldivyvpEpmtywrvlgasetianiyttqhrldqgeisagdaavvmtsaq
itmgmpyavtdavlaphiggeappaltlifdRhpiaallcypaarylmgsmtpqavlafvalipptlpgtniv
lgalpedrhidrlakRqRpgErldlamlaairrvygllantvrylqcggswredwgqlsprphigdtlftlfr
apellapngdlynvfawaldvlakrlrsmhvfildydqspagcrdallqltsgmvqthvttpgsipticdlar
tfaremge
>5EAT_A
masaavanyeeeivrpvadfspslwgdqflsfsidnqvaekyaqeiealkeqtrsmllatgrkladtlnlidi
ierlgisyhfekeideildqiynqnsncndlctsalqfrllrqhgfnispeifskfqdengkfkeslasdvlg
llnlyeashvrthaddiledalafstihlesaaphlksplreqvthaleqclhkgvprvetrffissiydkeq
sknnvllrfakldfnllqmlhkqelaqvsrwwkdldfvttlpyaRdrvvecyfWalgvyfepqysqarvmlvk
tismisivddtfdaygtvkeleaytdaiqrwdineidrlpdymkisykaildlykdyekelssagrshivcha
iermkevvrnynvestwfiegytppvseylsnalaTTTyyylattsylgmksateqdfewlsknpkileasvi
icRviDdtatyeveksrgqiatgieccmrdygistkeamakfqnmaetawkdinegllrptpvstefltpiln
larivevtYihnlDgYthpeevlkphiinllvdsiki
>1ODT_C
mqlfdlpldqlqtykpektapkdfsefwklsleelakvqaepdlqpvdypadgvkvyrltyksfgnaritgwy
avpdkegphpaivkyhgynasydgeihemvnwalhgyatfgmlvrgqqssedtsisphghalgwmtkgildkd
tyyyrgvyldavralevissfdevdetrigvtggAQgggltiaaaalsdipkaavadypylsnferaidvale
epyleinsffrrngspetevqamktlsyfdimnladrvkvpvlmsigliDkvtppstvfaaynhletkkelkv
yryfgHeyipafqteklaffkqhlk
>1I6P_A
mkdidtlisnnalwskmlveedpgffeklaqaqkprflwigcsDsRvpaerltglepgelfvhrnvanlviht
dlnclsvvqyavdvlevehiiicghygcggvqaavenpelglinnwllhirdiwfkhssllgempqerrldtl
celnvmeqvynlghstimqsawkrgqkvtihgwaygihdgllrdldvtatnretleqryrhgisnlklkhanh
k
>1AX4_A
makrivepfrikmvekirvpsreereaalkeagynpfllpssavyidlltdsgtnamsdhqwaamitgdeaya
gsrnyydlkdkakelfnydyiipahqgrgaenilfpvllkykqkegkaknpvfisnfhFdttaahvelngcka
inivtekafdsetyddwkgdfdikklkeniaqhgadnivaivstvtcnsaggqpvsmsnlkevyeiakqhgif
vvmDsarfcenayfikardpkyknatikevifdmykyadaltmsakkdpllnigglvairdneeiftlarqrc
vpmegfvtygglagrdmaamvqgleegteeeylhyrigqvkylgdrlreagipiqyptgghavfvdckklvpq
ipgdqfpaqavinalylesgvraveigsfllgrdpatgeqkhadmefmrltiarrvytndhmdyiadaliglk
ekfatlkglefeyeppvlrhftarlkpie
>1A8H_A
mekvfyvttpiyyvnaephlghayttvvadflarwhrldgyrtffltgtdehgetvyraaqaagedpkafvdr
vsgrfkrawdllgiayddfirtteerhkkvvqlvlkkvyeagdiyygeyeglycvscerfytekelveglcpi
hgrpverrkegnyffrmekyrpwlqeyiqenpdlirpegyrnevlamlaepigdlsisrpksrvpwgiplpwd
enhvtyvwfdallnyvsaldypegeayrtfwphawhligkdilkphavfwptmlkaagipmyrhlnvggfllg
pdgrKmsKtlgnvvdpfallekygrdalryyllreipygqdtpvseealrtryeadladdlgnlvqrtramlf
rfaegripepvageelaegtglagrlrplvrelkfhvaleeamayvkalnryinekkpwelfkkepeearavl
yrvveglriasilltpampdkmaelrralglkeevrleeaerwglaeprpipeeapvlfpkk
>3K5D_C
sfvemvdnlrgksgqgyyvemtvgsppqtlnilvDTgSsnfavgaaphpflhryyqrqlsstyrdlrkgvyvp
ytqgkwegelgtdlvsiphgpnvtvraniaaitesdkffingsnwegilglayaeiarpddslepffdslvkq
thvpnlfslqlcgaggsmiiggidhslytgslwytpirrewyyeviivrveingqdlkmdckeynydksivDs
gTtnlrlpkkvfeaavksikaasstekfpdgfwlgeqlvcwqagttpwnifpvislylmgevtnqsfritilp
qqylrpvedvatsqddcykfaisqsstgtvmgavimegfyvvfdrarkrigfavsachvhdefrtaavegpfv
tldmedcgyni
>1MW3_A

spnsqylktrildiytpeqragieksedwrqfsrrmdthfpklmneldsvygnneallpmlemllaqawqsys
qrnsslkdidiarennpdwilsnkqvggvcyvdlfagdlkglkdkipyfqelgltylhlmplfkcpegksdgg
yavssyrdvnpalgtigdlreviaalheagisavvdfifnHtsnehewaqrcaagdplfdnfyyifpdrrmpd
qydrtlreifpdqhpggfsqledgrwvwttfnsfqwdlnysnpwvfRamagemlflanlgvdilRmDavaFiw
kqmgtscenlpqahalirafnavmriaapavffkSEaiVhpdqvvQyigqdecqigynplqmallwntlatre
vnllhqaltyrhnlpehtawvnyVrsHDdigwtfadedaaylgisgyDhrqflnrffvnrfdgsfargvpfqy
npstgdcrvsgtaaalvglaqddphavdrikllysialstgglpliylgdevgtlndddwsqdsnksddsrwa
hrprynealyaqrndpstaagqiyqdlrhmiavrqsnprfdggrlvtfntnnkhiigyirnnallafgnfsey
pqtvtahtlqampfkahdliggktvslnqdltlqpyqvmwleia
>1S9D_A
mrilmvgldaagkttilyklkTipTigfnvetveyknisftvwdvggQdkirplwrhyfqntqglifvvdsnd
rervneareelmrmlaedelrdavllvfankqdlpnamnaaeitdklglhslrhrnwyiqatcatsgdglyeg
ldwlsnqlr
>1D2R_A
mktifsgiqpsgvitignyigalrqfvelqheyncyfcivdqhaitvwqdphelrqnirrlaalylavgidpt
qatlfiqsevpahaqaawmlqcivyigelermtqfkeksagaaaaaaglltypplmaadillyntdivpvged
qkqhieltrdlaerfnkrygelftipearipkvgarimslvdptkKmsKsdpnpkayitllddaktiekkiks
avtdsegtirydkeakpgisnllniystlsgqsieelerqyegkgygvfkadlaqvvietlrpiqeryhhwme
seeldrvldegaekanrvasemvrkmeqamglgr
>1QRR_A
mkrvmviggdgycgwatalhlskknyevcivdnlvrrlfdhqlglesltpiasihdrisrwkaltgksielyv
gdicdfeflaesfksfepdsvvhfgeqrsapysmidrsravytqhnnvigtlnvlfaikefgeechlvklgTm
geygtpnidieegyitithngrtdtlpypkqassfYHlsKvhdshniaftckawgiratdlnqgvvygvktde
temheelrnrldydavfgtalnrfcvqaavghpltvygkggqtrgyldirdtvqcveiaianpakagefrvfn
qfteqfsvnelaslvtkagsklgldvkkmtvpnprveaeehyynakhtklmelglephylsdslldsllnfav
qfkdrvdtkqimpsvswkkigvktksmtt
>3Q82_A
qsitdynykkplhndyqildkskifgsnsgsfvmysmaadayyiynekesrkryspnStyKiylamfgldrhi
indensrmswnhkhypfdawnkeqdlntamqnSvnwyFerisdqipknytatqlkqlnygnknlgsyksyWme
dslkisnleqvivfknmmeqnnhfskkaknqlsssllikknekyelygktgTgivngkynngwfvgyvitnhd
kyyfathlsdgkpsgknaelisekilkemgvln
>1QWN_A
rsshhhhhhgefddpirpplkvarsprpgqcqdvvqdvpnvdvqmlelydrmsfkdidggvwkqgwnikydpl
kynahhklkvfvvphshndpgwiqtfeeyyqhdtkhilsnalrhlhdnpemkfiwaeisyfarfyhdlgenkk
lqmksivkngqlefvtggwvmpdeanshwrnvllqltegqtwlkqfmnvtptaswaiDpfghsptmpyilqks
gfknmliqrthysvkkelaqqrqleflwrqiwdnkgdtalfthmmpfysydiphtcgpdpkvccqfdfkrmgs
fglscpwkvpprtisdqnvaarsdllvdqwkkkaelyrtnvlliplgdDfrfkqntewdvqrvnyerlfehin
sqahfnvqaqfgtlqeyfdavhqaeragqaefptlsgdfftyadrsdnywsgyytsrpyhkrmdrvlmhyvra
aemlsawhswdgmarieerleqarrelslfqhhdgitgtakthvvvdyeqrmqealkacqmvmqqsvyrlltk
psiyspdfsfsyftlddsrwpgsgvedsrttiilgedilpskhvvmhntlphwreqlvdfyvsspfvsvtdla
nnpveaqvspvwswhhdtltktihpqgsttkyriifkarvppmglatyvltisdskpehtsyasnlllrknpt
slplgqypedvkfgdpreislrvgngptlafseqgllksiqltqdsphvpvhfkflkygvrshgdrsgaylfl
pngpaspvelgqpvvlvtkgklessvsvglpsvvhqtimrggapeirnlvdigsldnteivmrlethidsgdi
fytdlnglqfikrrrldklplqanyypipsgmfiedantrltlltgqplggsslasgeleimqdrrlasdder
glgqgvldnkpvlhiyrlvlekvnncvrpsklhpagyltsaahkasqslldpldkfifaenewigaqgqfggd
hpsaredldvsvmrrltkssaktqrvgyvlhrtnlmqcgtpeehtqkldvchllpnvarcerttltflqnleh
ldgmvapevcpmetaayvsshss
>1FO6_A
mtrqmilavgqqgpiaraetreqvvgrlldmltnaasrgvnfivfpElalttffprwhftdeaeldsfyetem
pgpvvrplfetaaelgigfnlgyaelvveggvkrrfNtsilvdksgkivgkyrKihlpghkeyeayrpfqhlE
kryfepgdlgfpvydvdaakmgmfiCndrrwpetwrvmglkgaeiicggyNtpthnppvpqhdhltsfhhlls
mqagsyqngawsaaagkvgmeegcmllghscivaptgeivalttttledevitaaldldrcrelrehifnfkah
rqpqhygliaef
>1ZM7_B

tkyaegtqpftvliegnigsgkttylnhfekykndiclltEpvekwrnvngvdllelmykdpkkwampfqsyv
tltmlqshtaptnkklkimeRsifsarycfvenmrrngsleqgmyntleewykfieesihvqadliiylrtsp
evayerirqrarseescvplkylqelhelhedwlihqrrpqsckvlvldadl
>2PDA_A
gkkmmttdgntatahvayamsevaaiypiTpsstmgeeaddwaaqgrknifgqtltiremqsEagaagavhga
laagalttftasqglllmipnmykisgellpgvfhvtaRaiaahalsifgdhqdiyaarqtgfamlasssvq
eahdmalvahlaaiesnvpfmhffdgfrtsheiqkievldyadmaslvnqkalaefraksmnpehphvrgtaq
npdiyfqgreaanpyylkvpgivaeymqkvasltgrsyklfdyvgapdaervivsmgsscetieevinhlaak
gekiglikvrlyrpfvseaffaalpasakvitvldrtkepgapgdplyldvcsafvergeampkilagryglg
skefspamvksvydnmsgakknhftvgieddvtgtslpvdnafadttpkgtiqcqfwglgadgtvgankqaik
iigdntdlfaqgyfsydskksggitishlrfgekpiqstylvnradyvachnpayvgiydilegikdggtfvl
nspwssledmdkhlpsgikrtiankklkfynidavkiatdvglggrinmimqtaffklagvlpfekavdllkk
sihkaygkkgekivkmntdavdqavtslqefkypdswkdapaetkaepmtneffknvvkpiltqqgdklpvsa
feadgrfplgtsqfekrgvainvpqwvpenciqcnqcafvcphsailpvlakeeelvgapanftaleakgkel
kgykfriqintldcMgcgncadicppkekalvmqpldtqrdaqvpnleyaaripvksevlprdslkgsqfqep
lmefsgacsgcgetpyvrvitqlfgermfianatgcssiwgasapsmpyktnrlgqgpawgnslfedaaeygf
gmnmsmfarrthladlaakalesdasgdvkealqgwlagkndpikskeygdklkkllagqkdgllgqiaamsd
lytkksvwifggdgwaydigyggldhvlasgedvnvfvmdtevysNtggqsskatptgavakfaaagkrtgkk
dlarmvmtygyvyvatvsmgyskqqflkvlkeaesfpgpslviayatcinqglrkgmgksqdvmntavksgyw
plfrydprlaaqgknpfqldskapdgsveeflmaqnrfavldrsfpedakrlraqvaheldvrfkelehmaat
nifesfapaggkadgsvdfgegaefctrddtpmmarpdsgeacdqnragtseqqgdlskrtkk
>1BVZ_A
mlleaifheakgsyaypisetqlrvrlrakkgdvvrcevlyadryaspeeelahalagkagsderfdyfeall
ecstkrvkyvflltgpqgeavyfgetgfsaerskagvfqyayihrsevfttpewakeaviyqifperfangdp
sndppgteqwakdarprhdsfyggdlkgvidrlpyleelgvtalyftpifaspshhkydtadylaidpqfgdl
ptfrrlvdeahrrgikiildavfnhagdqffafrdvlqkgeqsrykdwffiedfpvsktsrtnyetfavqvpa
mpklrtenpevkeylfdvarfwmeqgidgwrlDvanevdhafwrefrrlvkslnpdalivgEiWhdasgwlmg
dqfdsvmnylfresvirffatgeihaerfdaeltrarmlypeqaaqglwnlldshDterfltscggneakfrl
avlfqmtylgtpliyygdeigmagatdpdcrrpmiweekeqnrglfefykelirlrhrlasltrgnvrswhad
kqanlyafvrtvqdqhvgvvlnnrgekqtvllqvpesggktwldcltgeevhgkqgqlkltlrpyqgmilwng
r
>1TRK_A
mtqftdidklavstirilavdtvskansgHpgaplgmapaahvlwsqmrmnptnpdwinrdrfvlsnghaval
lysmlhltgydlsiedlkqfrqlgsrtpghpefelpgvevttgplgqgisnavgmamaqanlaatynkpgftl
sdnytyvflgdgclqegisseasslaghlklgnliaiyddnkitidgatsisfdedvakryeaygwevlyven
gnedlagiakaiaqaklskdkptlikmtttigygslhagshsvHgaplkaddvkqlkskfgfnpdksfvvpqe
vydhyqktilkpgveannkwnklfseyqkkfpelgaelarrlsgqlpanwesklptytakdsavatrklsetv
ledvynqlpeliggsadltpsnltrwkealdfqppssgsgnysgryirygirehamgaimngisafganykpy
ggtflnfvsyaagavrlsalsghpviwvathdsigvgedgpthqpietlahfrslpniqvwrpadgnevsaay
knsleskhtpsiialsrqnlpqlegssiesaskggyvlqdvanpdiilvatgsevslsveaaktlaaknikar
vvslpdfftfdkqpleyrlsvlpdnvpimsvevlattcwgkyahqsfgidrfgasgkapevfkffgftpegva
eraqktiafykgdklisplkkaf
>1NVT_A
gplgsminaktkviglighpvehsfspimhnaafkdkglnyvyvafdvlpenlkyvidgakalgivgfnvtip
hkieimkyldeidkdaqligavntikieDgkaigyntdgigarmaleeeigrvkdkniviygaggaaravafe
lakdnniiianrtvekaealakeiaeklnkkfgeevkfsgldvdldgvdiiinatpigmypnidvepivkaek
lredmvvmdliynpletvllkeakkvnaktinglgmliyqgavafkiwtgvepnievmknaiidkitk
>1BQC_A
atglhvkngrlyeangqefiirgvshphnwypqhtqafadikshgantvrvvlsngvrwskngpsdvanvisl
ckqnrlicmlevhdttgygeqsgastldqavdywielksvlqgeedyvlinigNEpygndsatvaawatdtsa
aiqrlraagfehtlvvdapnwgqdwtntmrnnadqvyasdptgntvfsiHmYgvysqastitsylehfvnagl
pliigEfghdhsdgnpdedtimaeaerlklgyigwswsgngggveyldmvynfdgdnlspwgerifygpngia
stakeavifg
>1RQL_A

mdrmkieavifDwagttvdygcfaplevfmeifhkrgvaitaeeArkpMgllKidHvraltempriasewnrv
frqlpteadiqemyeefeeilfailpryaspinavkeviaslrergikigsttgytremmdivakeaalqgyk
pdflvtpddvpagrpypwmcyknamelgvypmnhmikvgdtvsdmkegrnagmwtvgvilgsselglteeeve
nmdsvelrekievvrnrfvengahftietmqelesvmehiekqeliis
>1BG0_A
vdqatldkleagfkklqeasdcksllkkhltkdvfdsiknkktgmgatlldviqsgvenldsgvgiyapdaes
yrtfgplfdpiiddyhggfkltdkhppkqwgdintlvgldpagqfiistrvRcgrslqgypfnpcltaeqyke
meekvsstlssmedelkgtyypltgmskatqqliddhflfkegdrflqtanacrywptgrgifhndaktflv
wvneEdhlRiismqkggdlktvykrlvtavdniesklpfshddrfgfltfcptnlgttmRasvhiqlpklakd
rkvlediaskfnlqvRgtrgehteseggvydisnkrrlglteyqavremqdgilemikmekaaa
>1CF2_R
mkavaingygtvgkrvadaiaqqddmkvigvsktrpdfearmalkkgydlyvaipervklfekagievagtvd
dmldeadividctpegigaknlkmykekgikaifqggekhediglsfnslsnyeesygkdytrvvsCnttglc
rtlkplhdsfgikkvravivrrgadpaqvskgpinaiipnppklpshhgpdvktvldinidtmavivpttlmH
qhnvmveveetptvddiidvfedtprvilisaedgltstaeimeyakelgrsrndlfeipvwresitvvdnei
yymqavhqesdivpenvdavrailemeedkyksinktnkamnil
>1EHY_A
mairrpedfkhyevqlpdvkihyvregagptllllhgWpgfwwewskvigplaehydvivpdlrgfgdsekpd
lndlskysldkaaddqaalldalgiekayvvghDFaaivlhkfirkysdrvikaaifdpiqpdfgpvyfglgh
vheswYsqfhqldmavevvgssrevckkyfkhffdhwsyrdellteeelevhvdncmkpdnihggfnyYrani
rpdaalwtdldhtmsdlpvtmiwglgDtcvpyapliefvpkyysnytmetiedcgHflmvekpeiaidrikta
fr
>2YPN_A
mldnvlriatrqsplalwqahyvkdklmashpglvvelvpmvtrgdvildtplakvggkglfvkelevallen
radiavhsmkDvpvefpqglglvticeredprdafvsnnydsldalpagsivgtsslRRqcqlaerrpdliir
slRgnvgtRlskldngeydaiilavaglkrlglesriraalppeislpavgqgavgiecrlddsrtrellaal
nhhetalrvtaeramntrleggCqvpigsyaelidgeiwlralvgapdgsqiirgerrgapqdaeqmgislae
ellnngareilaevyngdapa
>1KKT_B
snqakadavkeafqhawngymkyafphdeltpvsnghadsrngwgasavdalstavimgkadvvnailehvad
idfsktsdtvslfEttiRylagmlsgydllqgpaknlvdnqdlidglldqsrnladvlkfafdtpsgvpynni
nitshgndgattnglavtgtlvlewtrlsdltgdeeyaklsqkaesyllkpqpsssepfpglvgssinindgq
fadsrvswnggdDsfyeylikmyvydpkrfetykdrwvlaaestikhlkshpksrpdltflssysnrnydlss
qhltcfdggsfllggtvldrqdfidfglelvdgceatynstltkigpdswgwdpkkvpsdqkefyekagfyis
sgsyvlrpeviesfyyahrvtgkeiyrdwvwnafvainstcrtdsgfaavsdvnkanggskydnqesflfaev
mkysylahsedaawqvqkggkntfvynteahpisvar
>1JEN_B
meaahffegtEkllevwfsrqqpdanqgsgdlrtiprsewdillkdvqcsiisvtktdkqeayvlsE
>1PL7_C
aaaakpnnlslvvhgpgdlrlenypipepgpnevllrmhsvgicGSdvHyweygrignfivkkpmvlgheasg
tvekvgssvkhlkpgdrvaiepgaprendefckmgrynlspsiffcatppddgnlcrfykhnaafcyklpdnv
tfeegalieplsvgihacrrggvtlghkvlvcgagpigmvtllvakamgaaqvvvtdlsatrlskakeigadl
vlqiskespqeiarkvegqlgckpevtiectgaeasiqagiyatrsggtlvlvglgsemttvpllhaairevd
ikgvfrycntwpvaismlasksvnvkplvthrfplekaleafetfkkglglkimlkcdpsdqnp
>1BZC_A
memekefeqidksgswaaiyqdirheasdfpcrvaklpknknrnryrdvspfdhsriklhqedndyinaslik
meeaqrsyiltqgplpntcghfwemvweqksrgvvmlnrvmekgslkcaqywpqkeekemifedtnlkltlis
ediksyytvrqlelenlttqetreilhfhyttwpDfgvpespasflnflfkvresgslspehgpvvvhCsagi
gRSgtfcladtclllmdkrkdpssvdikkvlldmrkfrmgliqtaeqlrfsylaviegakfimgdssvqdqwk
elshedlepppehipppprppkrilephn
>1OFG_A
atlpagasqvpttpagrpmpyairpmpedrrfgyaivglgkyalnqilpgfagcqhsriealvsgnaekakiv
aaeygvdprkiydysnfdkiakdpkidavyiilpnslhaefairafkagkhvmceKpmatsvadcqrmidaak
aankklmigyrchydpmnraavklirenqlgklgmvttdnsdvmdqndpaqqwrlrrelagggslmdigiYgl

ngtryllgeepievraytysdpnderfvevedriiwqmrfrsgalshgassystttsrfsvqgdkavllmdp
atgyyqnlisvqtpghanqsmmpqfimpannqfsaqldhlaeavinnkpvrspgeegmqdvrliqaiyeaart
grpvntdwgyvrqggy
>1L7Q_A
mvdgnysvasnvmvpmrdgvrlavdlyrpdadgpvpvllvrnpYdkfdvfawstqstnwlefvrdgyavviqd
trglfasegefvphvddeadaedtlswileqawcdgnvgmfgvAYlgvtqwqaavsgvgglkaiapsmasadl
yrapwygpggalsveallgWsaligtglitsrsdarpedaadfvqlaailndvagaasvtplaeqpllgrlip
wvidqvvdhpdndeswqsislferlgglatpalitagwyDgfvgeslrtfvavkdnadarlvvgpwsHsnltg
rnadrkfgiaatypiqeattmhkaffdrhlrgetdalagvpkvrlfvmgidewrdetdwplpdtaytpfylgg
sgaantstgggtlstsisgtesadtylydpadpvpslggtllfhngdngpadqrpihdrddvlcystevltdp
vevtgtvsarlfvsssavdtdftaklvdvfpdgraialcdgivrmryretlvnptlieageiyevaidmlats
nvflpghrimvqvsssnfpkydrnsntggviareqleemctavnrihrgpehpshivlpiikr
>1EI9_A
dppaplplviwhgMgdsccnplsmgaikkmvekkipgihvlsleigktlredvensfflnvnsqvttvcqila
kdpklqqgynamgfSQggqflravaqrcpsppmvnlisvggqhqgvfglprcpgesshicdfirktlnagayn
kaiqerlvqaeywhdpirediyrnhsifladinqergvnesykknlmalkkfvmvkflnDtivdpvdsewfgf
yrsgqaketiplqestlytqdrlglkamdkagqlvflalegdHlqlseewfyahiipfle
>1HRD_A
skyvdrviaevekkyadepefvqtveevlsslgpvvdahpeyeevallermvipervviefrvpweddngkvhv
ntgyrvqfngaigpykgglrfapsvnlsimkflgfeqafkdslttlpmggaKggsdfdpngksdrevmrfcqa
fmtelyrhigpdidvpagDlgvgareigymygqyrkivggfyngvltgkarsfggslvrpeatgygsvyyvea
vmkhendtlvgktvalagfgnvawgaakklaelgakavtlsgpdgyiydpegitteekinymlemrasgrnkv
qdyadkfgvqffpgekpwgqkvdiimpcatqndvdleqakkivannvkyyievanmpttnealrflmqqpnmv
vapskavnaggvlvsgfemsqnserlswtaeevdsklhqvmtdihdgsaaaaeryglgynlvaganivgfqki
adammaqgiaw
>2BMI_A
aqksvkisddisitqlsdkvytyvslaeiegwgmvpsngmivinnhqaalldtpindaqtemlvnwvtdslha
kvttfipnhwhgDcigglgylqrkgvqsyanqmtidlakekglpvpehgftdsltvsldgmplqcyylgggha
tdnivvwlptenilfggcmlkdnqatsigNisdadvtawpktldkvkakfpsaryvvpghgdyggteliehtk
qivnqyiestskp
>1CG2_A
qkrdnvlfqaatdeqpaviktleklvnietgtgdaegiaaagnfleaelknlgftvtrsksaglvvgdnivgk
ikgrggknlllmsHmdtvylkgilakapfrvegdkaygpgiaDdkggnavilhtlkllkeygvrdygtitvlf
ntdEEkgsfgsrdliqeeakladyvlsfEptsagdeklslgtsgiayvqvnitgkashagaapelgvnalvea
sdlvlrtmniddkaknlrfnwtiakagnvsniipasatlnadvryarnedfdaamktleeraqqkklpeadvk
vivtrgrpafnageggkklvdkavayykeaggtlgveertgggtdaayaalsgkpvieslglpgfgyHsdkae
yvdisaiprrlymaarlimdlgag
>1B04_A
mdrqqaerraaelrellnrygyeyyvldrpsvpdaeydrlmqeliaieeqypelktsdsptqriggppleafr
kvahrvpmmslanafgegdlrdfdrrvrqevgeaayvcelAidglavsvryedgyfvqgatrgdgttgedite
nlktirslplrlkepvsleargeafmpkasflrlneerkargeelfanprnaaagslrqldpkvaasrqldlf
vygladaealgiashsealdylqalgfkvnperrrcanideviafvsewhdkrpqlpyeidgivikvdsfaqq
ralgatakspr waiaykfpaeevvtt
>1BWP_A
msgdenpaskptpvqdvqgdgrwmslhhrfvadskdkepevvfigdSavqlmhqceiwrelfsplhalnfgig
GdstqhvlwrlengelehirpkivvvwvgtNnhghtaeqvtggikaivqlvnerqpqarvvvlgllprgqhpn
plreknrrvnelvraalaghprahfldadpgfvhsdgtishhdmyDylHlsrlgytpvcralhslllrlltqd
qgqggaplpepspp
>1FUA_A
mernklarqiidtclemtrlglnqgtagnvsvryqdgmlitptgipyeklteshivfidgngkheegklpssE
wrfhmaayqsrpdanavvhnhavhctavsilnrsipaihYmiaaaggnsipcapyatfgtrelsehvalalkn
rkatllqhhgliacevnlekalwlahevevlaqlylttlaitdpvpvlsdeeiavvlekfktyglriee
>1B93_A

melttrtlparkhialvaHdhckqmlmswverhqplleqhvlyatgttgnlisratgmnvnamlsGpmggDqq
vgalisegkidvliffwDplnavpHdpDvkallrlatvwnipvatnvatadfiiqsphfndavdilipdyqry
ladrlk
>2BRW_A
vkdtytdrlddwngiiagnqyydskndqmaklnqelegkvadslssissqadriylwekfsnyktsanltaty
rkleemakqvtnpssryyqdetvvrtvrdsmewmhkhvynseksivgnwwdyeigtprainntlslmkeyfsd
eeikkytdviekfvpdpehfrkttdnpfkalggNlvdmgrvkviagllrkddqeisstirsieqvfklvdqge
gfyqdgsyidHtnvaytgaYgnvlidglsqllpviqktknpidkdkmqtmyhwidksfapllvngelmdmsrg
rsisranseghvaavevlRgihriadmsegetkqrlqslvktivqsdsyydvfknlktykdislmqsllsdag
vasvprtsylsafnkmdktamynaekgfgfglslfssrtlnyehmnkenkrgwytsdgmfylyngdlshysdg
ywptvnpykmpgttetdakradsdtgkvlpsafvgtsklddanatatmdftnwnqtltahkswfmlkdkiafl
gsniqntstdtaattidqrklessnpykvyvndkeaslteqekdypetqsvflessdskknigyfffkkssis
mskalqkgawkdinegqsdkeveneflt isqahkqngdsygymlipnvdratfnqmikelessliennetlqs
vydakqgvwgivkyddsvstisnqfqvlkrgvytirkegdeykiayynpetqesapdqevfkkl
>1GXS_A
qlqqqeddrilglpgqpngvafgmyggyvtiddnngralyywfqeadtadpaaaplvlwlnggpgcssiglga
mqelgafrvhtngeslllneyawnkaanilfaespagvgfsysntssdlsmgddkmaqdtytflvkwferfph
ynyrefyiageSghfipqlsqvvyrnrnnspfinfqgllvssgltndhedmigmfeswwhhglisdetrdsgl
kvcpgtsfmhptpectevwnkalaeqgninpytiytptcdrepspyqrrfW
>1R4F_A
saknvvldhDgnlddfvamvllasntekvrligalctdadcfvengfnvtgkimclmhnnmnlplfpigksaa
tavnpfpkeWrclaknmddmpilnipenvelwdkikaenekyegqqlladlvmnseekvticvtgplsnvawc
idkygekftskveecvimggavdvrgnvflpstdgtaewNiywdpasaktvfgcpglrrimfsldstntvpvr
spyvqrfgeqtnfllsilvgtmwamyaAdaltaayvvdqkvanvdpvpidvvvdkqpnegatvrtdaenyplt
fvarnpeaefflldmllrsarac
>1MUD_A
mqasqfsaqvldwydkygrktlpwqidktpykvwlsEvmlqqtqvatvipyferfmarfptvtdlanapldev
lhlwtglgyyararnlhkaaqqvatlhggkfpetfeevaalpgvgrstagailslslgkhfpilNgnvkrvla
rcyavsgwpgkkevenklwslseqvtpavgverfnqammdlgamictrskpkcslcplqngciaaannswaly
pgkkpk
>1MQW_A
maehdfetissetlhtgaifalrrdqvrmpgggivtrevvehfgavaivamddngnipmvyqyRhtygrrlwe
lpaglldvageppbltaarelreevglqastwqvlvdldtapgfsdesvrvylatglrevgrpeahheEadmt
mgwypiaeaarrvlrgeivnsiaiagvlavhavttgfaqprpldtewidrptafaarraer
>2FN6_B
kefaysepcldkedkkavlevlnskqltqgkrsllfeealceflgvkhalvfnsatsalltlyrnfsefsadr
neiittpisFvatanmllesgytpvfagikndgnidelalekli nertkaivsvdyagksvevesvqklckkh
slsflsDsshalgseyqnkkvggfalasvfsfhaiKpittaeggavvtndselhekmklfrshgmlkkdffeg
evksighnfrlneiqsalglsqlkkapflmqkreeaaltydrifkdnpyftplhpllkdkssnhlypilmhqk
fftckklileslhkrgilaqvhykpiyqyqlyqqlfntaplksaedfyhaeislpchanlnlesvqniahsvl
ktfesf
>2SQC_B
apayartldraveyllscqkdegywwgpllsnvtmeaEyvllchildrvdrdrmekirryllheqredgtwal
ypggppdldttiEayvalkyigmsrdeepmqkalrfiqsqggiessRvftrmwlalvgeypwekvpmvppeim
flgkrmplniyefgsWaratvvalsivmsrqpvfplperarvpelyetdvpprrrgakggggwifdaldralh
gyqklsvhpfrraaeiraldwllerqagdgswggiQppwfyalialkildmtqhpafikgweglelygveldy
ggwmfqasispvWdtglavlalraaglpadhdrlvkagewlldrqitvpgdwavkrpnlkpggfaFqfdnvyy
pDvCDtavvvwalntlrlpderrrrdamtkgfrwivgmqssnggwgaydvdntsdlpnhipfsdfgevtdpps
edvtaHvlecfgsfgyddawkvirraveylkreqkpdgswfgrWgvnylYgtgavvsalkavgidtrepyiqk
aldwveqhqnpdggwgedcrsyedpayagkgastpsqtawalmaliaggraeseaarrgvqylvetqrpdggw
depyytgtgFpgdFylgytmyrhvfptlalgrykqaier
>1WD8_A
gplgspqmaqgtlirvtpeqpthavcvlgtltqldicssapedctsfsinaspgvvvdiahsppakkkstgss
twpldpgvevtltmkaasgstgdqkvqisyygpktppvkallyltaveislcaditrtgkvkptravkdqrtw

```
twgpcgqgaillvncdrdnlessamdceddevldsedlqdmslmtlstktpkdfftnhtlvlhvarsemdkvr
vfqatrgklsskcsvvlgpkwpshylmvpggkhnmdfyvealafpdtdfpglitltislldtsnlelpeavvf
qdsvvfrvapwimtpntqppqevyacsifenedflksvttlamkakcklticpeeenmddqwmqDemeigyiq
aphktlpvvfdsprnrglkefpikrvmgpdfgyvtrgpqtggisgldsfgnlevsppvtvrgkeyplgrilfg
dscypsndsrqmhqalqdflsaqqvqapvklysdwlsvgHvDeflsfvpapdrkgfrlllasprscyklfqeq
qneghgeallfegikkkkqqkiknilsnktlrehnsfvercidwnrellkrelglaesdiidipqlfklkefs
kaeaffpnmvnmlvlgkhlgipkpfgpvingrccleekvcslleplglqctfindfftyhirhgevhCgtnvr
rkpfsfkwwnmvp
>1LCB_A
mleqpyldlakkvldeghfkpdrthtgtysifghqmrfdlskgfplltTkkvpfgliksEllwflhgdtnirf
llqhrnhiwdewafekwvksdeyhgpdmtdfghrsqkdpefaavyheemakfddrvlhddafaakygdlglvy
gsqwrawhtskgdtidqlgdvieqikthpysrrlivsawnpedvptmalppChtlyqfyvndgklslqlyqrS
aDiflgvpfniasyallthlvahecglevgefihtfgDaHlyvnhldqikeqlsrtprpaptlqlnpdkhdif
dfdmkdikllnydpypaikapvav
>1L8T_A
akmrispelkkliekyrcvkdtegmspakvyklvgenenlylKmtdsrykgttydverekdmmlwlegklpvp
kvlhferhdgwsnllmseadgvlcseeyedeqspekiielyaecirlfhsidisdcpytnsldsrlaeldyll
nndladvdcenweedtpfkdprelydflktekpeeelvfshgDlgdsnifvkdgkvsgfidlgrsgradkwyd
iafcvrsiredigeeqyvelffdllgikpdwekikyyilldelf
>1VID_A
mgdtkeqrilryvqqnakpgdpqsvleaidtyctqkewamnvgdakgqimdavireyspslvlelgaycgysa
vrmarllqpgarlltmemnpdyaaitqqmlnfaglqdkvtilngasqdlipqlkkkydvdtldmvfldhwKdr
ylpdtlllekcgllrkgtvlladnvivpgtpdflayvrgsssfecthyssylEymkvvdglekaiyqgpsspd
ks
>1UN1_B
vafgrnyvptwafdhikyfnggneiqlhldkytgtgfqskgsylfghfsmqmklvpgdsagtvtafylssqns
ehdEiDfEflgnrtgqpyilqtnvftggkgdreqriylwfdptkefhyysvlwnmymivflvddvpirvfknc
kdlgvkfpfnqpmkiysslwnaddwatrgglektdwskapfiasyrsfhidgceasveakfcatqgarwwdqk
efqdldafqyrrlswvrqkytiynyctdrsrypsmppeckrdrdi
>1MHL_C
vncetscvqqppcfplkippndpriknqadcipffrscpacpgsnitirnqinaltsfvdasmvygseeplar
nlrnmsnqlgllavnqrfqdngrallpfdnlhddpclltnrsaripcflagdtRssempeltsmhtlllrehn
rlatelkslnprwdgerlyqearkivgamvqiityrdylplvlgptamrkylptyrsyndsvdprianvftna
fryghtliqpfmfrldnryqpmepnprvplsrvffaswrvvleggidpilrglmatpaklnrqnqiavdeire
rlfeqvmrigldlpalnmqrsrdhglpgynawrrfcglpqpetvgqlgtvlrnlklarklmeqygtpnnidiw
mggvseplkrkgrvgpllaciigtqfrklrdgdrfwwenegvfsmqqrqalaqislpriicdntgittvsknn
ifmsnsyprdfvncstlpalnlaswrea
>3BHK_A
sthfdvivvgagsmgmaagyqlakqgvktllvdafdpphtngshhgdTkiirhaygegreyvplalrsqelwy
eleketHhkiftktgvlvfgpkgesafvaetmeaakehsltvdllegdeinkrwpgitvpenynaifepnsgv
lfsencirayrelaeargakvlthtrvedfdispdsvkietangsytadklivsmgawnskllsklnldiplq
pyrqvvgffesdeskysndidfpgfmvevpngiyygfpsfggcglKlgyHtfgqkidpdtinrefgvypedes
nlrafleeympgangelkrgavCmytktldehfiidlhpehsnvviaagfsghgfKfssgvgevlsqlaltgk
tehdisifsinrpalkeslq
>1VOM_A
mnpihdrtsdyhkylkvkqgdsdlfkltvsdkryiwynpdpderdsyecgeivsetsdsftfktvdgqdrqvk
kddanqrnpikfdgvedmselsylnepavfhnlrvrynqdliytysglflvavnpfkripiytqemvdifkgr
rrnevaphifaisdvayrsmlddrqnqsllitgesGagktentkkviqylasvagrnqangsgvleqqilqan
pileafgnakttrNnnssrfgkfieiqfnnagfisgasiqsylleksrvvfqseternyhifyqllagataee
kkalhlagpesfnylnqsgcvdikgvsdedefkitrqamdivgfsqeeqmsifkiiagilhlgnikfekgage
gavlkdktalnaastvfgvnpsvlekalmeprilagrdlvaqhlnvekssssrdalvkalygrlflwlvkkin
nvlcseraayfigvldisGfEifkvnsfeqlcinytneklqqffnhhmfkveqeeylkekinwtfidfgldsq
atidlidgrqppgilalldeqsvfpnatdntlitklhshfskknakyeeprfsktefgvthyagqvmyeiqdw
leknkdplqqdlelcfkdssdnvvtklfndpniasrakkganfitvaaqykeqlaslmatlettnphfvrcii
```

pnnkqlpakledkvvldqlrcngvlegiritrkgfpnriiyadfvkryyllapnvprdaedsqkatdavlkhl
nidpeqyrfgitkiffragqlarieearelpn
>1DQW_A
mhkatykeraathpspvaaklfnimhekqtnlcasldvrttkellelvealgpkicllKthvdiltdfsmegt
vkplkalsakynfllfeDrKfaDigntvklqysagvyriaewaditnahgvvgpgivsglkqaaeevtkeprg
llmlaelsckgslstgeytkgtvdiaksdkdfvigfiaqrdmggrdegydwlimtpgvglddkgdalgqqyrt
vddvvstgsdiiivgrglfakgrdakvegeryrkagweaylrrcgqqd
>1OBA_A
mvkkndlfvDvsshngyditgileqmgttntiikisesttylnrclsaqveqsnpigfyhfarfggdvaeaer
eaqffldnvpmqvkylvlDyEddpsgdaqantnaclrfmqmiadagykpiyysykpfthdnvdyqqilaqfpn
slwiagyglndgtanfeyfpsmdgirwwqyssnpfDknivllddeeddkpktagtwkqdskgwwfrrnngsfp
ynkwekiggvwyyfdskgycltsewlkdnekwyylkdngamatgwvlvgsewyymddsgamvtgwvkyknnwy
ymtnergnmvsnefiksgkgwyfmntngeladnpsftkepdglitva
>1I0D_A
drintvrgpitiseagftlthehicgssagflrawpeffgsrkalaekavrglrraraagvrtivdvstfdig
rdvsllaevsraadvhivaatglwfdpplsmrlrsveeltqfflreiqygiedtgiragiikvattgkatpfq
elvlkaaaraslatgvpvtthtaasqrdgeqqaaifeseglspsrvcighsdDtddlsyltalaargyligld
HiphsaigledndsasallgirswqtrallikalidqgymkqilvsnDwlfgfssyvtnimdvmdrvnpdgma
fiplrvipflrekgvpqetlagitvtnparflsptlras
>1RPX_B
srvdkfsksdiivspsilsanfsklgeqvkaieqagcdwiHvDvmdgrfvpnitigplvvdslrpitdlpldv
Hlmivepdqrvpdfikagadivsvhceqsstihlhrtinqikslgakagvvlnpgtpltaieyvldavdlvli
msvnpgfggqsfiesqvkkisdlrkicaerglnpwievDggvgpknaykvieaganalvagsavfgapdyaea
ikgiktskrpe
>1KAE_A
msfntiidwnsctaeqqrqllmrpaisasesitrtvndildnvkargdealreysakfdkttvtalkvsaeei
aaaserlsdelkqamavavknietfhtaqklppvdvetqpgvrcqqvtrpvasvglyipggsaplfstvlmla
tpasiagckkvvlcspppiadeilyaaqlcgvqdvfnvggaqaiaalafgtesvpkvdkifgpgnafvteakr
qvsqrldgaaidmpagpsevlviadsgatpdfvasdllsqaehgpdsqvilltpaadmarrvaeaverqlael
praetarqalnasrlivtkdlaqcveisnqygpEHliiqtrnarelvdsitsagsvflgdwspesagdyasgt
nhvlptygytatcsslgladfqkrmtvqelskegfsalastietlaaaerltahknavtlrvnalkeqa
>1BU7_A
tikempqpktfgelknlpllntdkpvqalmkiadelgeifkfeapgrvtrylssqrlikeacdesrfdknlsq
alkfvrdfagdglftswtheknwkkahnillpsfsqqamkgyhammvdiavqlvqkwerlnadehievpedmt
rltldtiglcgfnyrfnsfyrdqphpfitsmvraldeamnklqranpddpaydenkrqfqedikvmndlvdki
iadrkasgeqsddllthmlngkdpetgeplddeniryqiitfliagheTtsgllsfalyflvknphvlqkaae
eaarvlvdpvpsykqvkqlkyvgmvlnealrlwptapafslyakedtvlggeyplekgdelmvlipqlhrdkt
iwgddveefrperfenpsaipqhafkpFgngqraCigqqfalheatlvlgmmlkhfdfedhtnyeldiketlt
lkpegfvvkakskkipl
>1OE8_A
mtgdhikviYfngrgraesirmtlvaagvnyederisfqdwpkikptipggrlpavkitdnhghvkwmvesla
iarymakkhhmmggteeeyynvekligqaedleheyyktlmkpeeekqkiikeilngkvpvlldiiceslkas
tgklavgdkvtladlvliavidhvtdldkefltgkypeihkhrenllassprlakylsdraatpf
>1D7R_A
mslnddatfwrnarqhlvryggtfepmiierakgsfvydadgraildftsgqmsavlghchpeivsvigeyag
kldhlfsgmlsrpvvdlatrlanitppgldralllstgaesneaairmaklvtgkyeivgfaqsWhgmtgaaa
satysagrkgvgpaavgsfaipapftyrprferngaydylaeldyafdlidrqssgnlaafiaepilssggii
elpdgymaalkrkceargmllilDeaqtgvgrtgtmfacqrdgvtpdiltlsKtlgaglplaaivtsaaieer
ahelgylfytthvsdplpaavglrvldvvqrdglvaranvmgdrlrrglldlmerfdcigdvrgrglllgvei
vkdrrtkepadglgakitrecmnlglsmnivqlpgmggvfriappltvsedeidlglsllgqaieral
>1DQA_A
gamassvlvtqepeielpreprpneeclqilgnaekgakflsdaeiiqlvnakhipaykletliethergvsi
rrqllskklsepsslqylpyrdynyslvmgaccenvigympipvgvagplcldekefqvpmattEgclvastn
rgcraiglgggassrvladgmtrgpvvrlpracdsaevkawletsegfavikeafdstsrfarlqklhtsiag

```
rnlyirfqsrsgdamgmnmiskgtekalsklheyfpemqilavsgnyctdKkpaainwiegrgksvvceavip
akvvrevlkttteamievninknlvgsamagsiggynahaanivtaiyiacgqDaaqnvgssncitlmeasgp
tnedlyisctmpsieigtvgggtnllpqqaclqmlgvqgackdnpgenarqlarivcgtvmagelslmaalaa
ghlvksHmihnrskinlqdlqgactkkta
>1XQW_A
mdqecienyakvngiyiyyklckapeekaklmtmhgGpgmshdyllslrdmtkegitvlfydqfgcgrseepd
qskftidygveeaealrsklfgnekvflmgsAYggalalayavkyqdhlkglivsgglssvpltvkemnrlid
elpakyrdaikkygssgsyenpeyqeavnyfyhqhllrsedwppevlksleyaerrnvyrimngpneftitgt
ikdwditdkisaikiptlitvgeyDevtpnvarvihekiagselhvfrdcsHltmwedregynkllsdfilkh
l
>2P2F_B
hkhaipaniadrclinpeqyetkykqsindpdtfwgeqgkildwitpyqkvkntsfapgnvsikwyedgtlnl
aancldrhlqengdrtaiiwegddtsqskhisyrelhrdvcrfantlldlgikkgdvvaiympmvpeaavaml
acarigavhsvifggfspeavagriidsssrlvitadegvragrsiplkknvddalknpnvtsvehvivlkrt
gsdidwqegrdlwwrdliekaspehqpeamnaedplfilytsgstgkpkgvlhttggylvyaattfkyvfdyh
pgdiywctadvgwvtghsyllygplacgattlmfegvpnwptparmcqvvdkhqvnilytaptairalmaegd
kaiegtdrsslrilgsvgepinpeawewywkkigkekcpvvdtwwqTetggfmitplpgaielkagsatrpff
gvqpalvdneghpqegategnlvitdswpgqartlfgdherfeqtyfstfknmyfsgdgarrdedgyywitgr
vddvlNvsghRlgtaeiesalvahpkiaeaavvgiphaikgqaiyayvtlnhgeepspelyaevrnwvrkeig
platpdvlhwtdslpktrsgKimrrilrkiaagddpgvveklleekqa
>1A65_A
qivnsvdtmtltnanvspdgftragilvngvhgplirggkndnfelnvvndldnptmlrptsihwhglfqrgt
nwadgadgvnqcpispghaflykftpaghagtfwyhshfgtqycdglrgpmviyddndphaalydeddentii
tladwyhipapsiqgaaqpdatlingkgryvggpaaelsivnveqgkkyrmrlislscdpnwqfsidghelti
ievdgeltephtvdrlqiftgqrysfvldanqpvdnywiraqpnkgrnglagtfangvnsailryagaanadp
ttsanpnpaqlneadlhalidpaapgiptpgaadvnlrfqlgfsggrftingtayespsvptllqimsgaqsa
ndllpagsvyelprnqvvelvvpagvlggphpfhlhghafsvvrsagsstynfvnpvkrdvvslgvtgdevti
rfvtdnpgpwffHCHiefhlmnglaivfaedmantvdannppvewaqlceiyddlppeatsiqtvv
>1EUG_A
maneltwhdvlaeekqqpyflntlqtvaserqsgvtiyppqkdvfnafrftelgdvkvvilgqDpyhgpgqah
glafsvrpgiaippsllnmykelentipgftrpnhgyleswarqgvlllntvltvragqahshaslgwetftd
kvislinqhregvvfllwgshaqkkgaiidkqrhhvlkapHpsplsahrgffgcnhfvlanqwleqhgetpid
wmpvlpaese
>3EP6_A
smfvskrrfilktCgttlllkalvpllklardysgfdsiqsffysrknfmkpshqgyphrnfqeeieflnaif
pngaaycmgrmnsdcwylytldfnqtleilmseldpavmdqfymkdgvtakdvtresgirdlipgsvidatmf
npcgysmngmksdgtywtihitpepefsyvsfetnlsqtsyddlirkvvevfkpgkfvttlfvnqpqkiegfk
rldcqsamfndynfvftsfak
>2DPH_B
agnksvvyhgtrdlrvetvpypklehnnrklehavilkvvstnicGSdqHiyrgrfivpkghvlgheitgevv
ekgsdvelmdigdlvsvpfnvacgrcrnckearsdvcennlvnpdadlgafgfdlkgwsggqaeyvlvpyady
mllkfgdkeqamekikdltlisdilptgfhgcvsagvkpgshvyiagagpvgrcaaagarllgaacvivgdqn
perlkllsdagfetidlrnsaplrdqidqilgkpevdcgvdavgfeahglgdeantetpngalnslfdvvrag
gaigipgiyvgsdpdpvnkdagsgrlhldfgkmwtksirimtgmapvtnynrhlteailwdqmpylskvmnie
vitldqapdgyakfdkgspakfvidphgmlknk
>1Q3Q_A
maqlsgqpvvilpegtqryvgrdaqrlnilaariiaetvrttlgpkgmdkmlvdslgdivvtnDcatildkid
lqhpaakmmvevaktqdkeagdgTTtavviagellrkaeelldqnihpsiitkgyalaaekaqeildeiairv
dpddeetllkiaatsitgknaeshkellaklaveavkqvaekkdgkyvvdldnikfekkagegveeselvrgv
vidkevvhprmpkrvenakialinealevkktetdakinitspdqlmsfleqeekmlkdmvdhiaqtganvvf
vqkgiddlaqhylakygimavrrvkksdmeklakatgakivtnvkdltpedlgyaevveerklagenmifveg
cknpkavtilirggtehvideveraleDavkvvkdvmedgavlpaggapeielairldeyakqvggkealaie
nfadalkiipktlaenagldtvemlvkvisehknrglgigidvfegkpadmlekgiieplrvkkqaiksasea
aimilriddviaakatkpeggqgggmpggmggmdmgm
```

>1X9H_A
msqllqdylnwenyilrrvdfptsyvvegevvrieamprlyisgmggsgvvadlirdfsltwnweveviavkd
yflkardglliavsysgntietlytveyakrrripavaittggrlaqmgvptvivpkasapRaalpqlltaal
hvvakvygidvkipegleppnealihklveefqkrptiiaaesmrgvayrvknefnEnakiepsveilpeahH
nwiegseravvaltsphipkehqervkatveivggsiyavemhpkgvlsflrdvgiasvklaeirgvnplatp
ridalKrrlq
>1EIX_A
mtltassssravtnspvvvaldyhnrddalafvdkidprdcrlKvgkemftlfgpqfvrelqqrgfdiflDlK
fhDipntaahavaaaadlgvwmvnvhasggarmmtaarealvpfgkdaplliavtvltsmeasdlvdlgmtls
padyaerlaaltqkcgldgvvcsaqeavrfkqvfgqefklvtpgirpqgseagdqrrimtpeqalsagvdymv
igrpvtqsvdpaqtlkainaslqrsa
>1QST_A
ldfdiltndgthrnmkllidlknifsrqlpkmpkeyivklvfdrhhesmvilknkqkviggicfrqykpqrfa
Evaflavtaneqvrgygtrlmnkfkdhmqkqnieylltyadnfaigyfkkqgftkehrmpqekwkgyikdydg
gtlmecyihpyvdy
>2B3I_A
asvqikmgtdkyaplyepkalsisagdtvefvmnkvgphnvifdkvpagesapalsntklaiapgsfysvtlg
tpgtysfyctpHrgagmvgtitve
>3E5R_B
gkikigingfgrigrlvarvalqsedvelvavndpfittdymtymfkydtvhgqwkhsdikikdsktlllgek
pvtvfgirnpdeipwaeagaeyvvestgvftdkekaaahlkggakkvvisapskdapmfvcgvnedkytsdid
ivsnasCttnclaplakvihdnfgiieglmttvHaitatqktvdgpsskdwrggraasfniipsstgaakavg
kvlpdlngkltgmsfrvptvdvsvvdltvriekaasydaiksaiksasegklkgiigyveedlvstdfvgdsr
ssifdakagialndnfvklvawydnewgysnrvidlirhmaktq
>1EHK_B
mvdehkahkailayekgwlafslamlfvfialiaytlathtagvipagklervdpttvrqegpwadpaqavvq
tgpnqytvyvlaFaFgyqpnpievpqgaeivfkitspdvihgfhvegtninvevlpgevstvrytfkrpgeyr
iicnqycglghqnmfgtivvke
>1EHK_A
mavraseisrvyeaypekkatlyflvlgflalivgslfgpfqalnygnvdaypllkrllpfvqsyyqgltlhg
vlnaivftqlfaqaimvylparelnmrpnmglmwlswwmafiglvvaalpllaneatvlytfypplkghwafy
lgasvfvlstwvsiyivldlwrrwkaanpgkvtplvtymavvfwlmwflaslglvleavlfllpwsfglvegv
dplvartlfwwtgHpivYfwllpayaiiytilpkqaggklvsdpmarlaflllflllstpvgfhhqfadpgidp
twkmihsvltlfvavpslmtaftvaaslefagrlrggrglfgwiralpwdnpafvapvlgllgfipggaggiv
nasftldyvvhntawvpgHFHlqvaslvtltamgslywllpnltgkpisdaqrrlglavvwlwflgmmimavg
lhwagllnvpRRayiaqvpdayphaavpmvfnvlagivllvalllfiyglfsvllsrerkpelaeaplpfaev
isgpedrrlvlamdrigfwfavaailvvlaygptlvqlfghlnpvpgwrlw
>1OGO_X
hgttanthcgadfctwwhdsgeintqtpvqpgnvrqshkysvqvslagtnnfhdsfvyesiprngngriyapt
dppnsntldssvddgisiepsiglnmawsqfeyshdvdvkilatdgsslgspsdvvirpvsisyaisqsddgg
ivirvpadangrkfsvefktdlytflsdgneyvtsggsvvgveptnalvifaspflpsgmiphmtpdntqtmt
pgpinngdwgaksilyfppgvywmnqdqsgnsgklgsnhirlnsntywvylapgayvkgaieyftkqnfyatg
hgilsgenyvyqanagdnyiavksdstslrmwwhnnlgggqtwycvgptinappfntmdfngnsgissqisdy
kqvgafffqtDgpeiypnsvvhdvfwhvnDdaikiyysgasvsratiwkchndpiiqmgwtsrdisgvtidtl
nvihtryiksetvvpsaiigaspfyasgmspdsrksismtvsnvvceglcpslfritplqnyknfvvknvafp
dglqtnsigtgesiipaasgltmglaisawtigggqkvtmenfqanslgqfnidgsywgewqis
>3ZQ6_B
afkdlfkfnkgkttfvfiggkgGvgKttisaatalwmarsgkktlvistdpahslsdslereightptkiten
lyaveidpevameeyqakdmlqdqmdmasmspgideaaafdqflrymttdeydivifdtaptghtlrllsfpe
imdswvgkmikirrqigsalqdmeatkkqinaarevmsdpertsfkmvvipeemsiyeseramkalekysiha
dgvivnqvlpeesdcefcnarrklqqerlkqirekfsdkvvaevpllkkeakgietlekiaeqlygepe
>2I4M_A
gshmrlsrfflpilkenpkeaeivshrlmlragmlrqeaagiyawlplghrvlkkieqivreeqnragaiell
mptlqladlwresgrydaygpemlriadrhkrellygptneemiteifrayiksykslplnlyhiqwkfRdeq

rprfgvmrgreflmkdaysfdvdeagarksynkmfvaylrtfarmglkaipmraetgpiggdlshefivlaet
gesgvyidrdvlnlpvpdenvdydgdltpiikqwtsvyaatedvheparyesevpeanrlntrgievgqifyf
gtkysdsmkanvtgpdgtdapihggsygvgvsrllgaiieachddngiiwpeavapfrvtilnlkqgdaatda
acdqlyrelsakgvdvlyddtdqragakfatadligipwqihvgprglaegkvelkrrsdgarenlaladvva
r
>1AMP_A
mppitqqatvtawlpqvdasqitgtisslesftnrfytttsgaqasdwiasewqalsaslpnasvkqvshsgy
nqksvvmtitgseapdewivigghldstigshtneqsvapgadddasgiaavtevirvlsennfqpkrsiafm
ayaaEevglrgsqdlanqyksegknvvsalqldmtnykgsaqdvvfitdytdsnftqyltqlmdeylpsltyg
fdtcgyacsdhaswhnagypaampfeskfndynprihttqdtlansdptgshakkftqlglayaiemgsatg
>2PTH_A
tiklivglaNpgaeyaatrHnagawfvdllaerlraplreeakffgytsrvtlggedvrllvpttfmnlsgka
vaamasffrinpdeilvahDeldlppgvakfklggghgghnglkdiisklgnnpnfhrlrigighpgdknkvv
gfvlgkppvseqklideaideaarctemwftdgltkatnrlhafkaq
>1GOJ_A
msssansikvvarfrpqnrveiesggqpivtfqgpdtctvdskeaqgsftfdrvfdmsckqsdifdfsikptv
ddilngyngtvfaygqtgagksytmmgtsiddpdgrgviprivеqiftsilssaanieytvrvsymeiymeri
rdllapqndnlpvheeknrgvyvkglleiyvssvqevyevmrrggnaravaatnmnqessrshsifvititqk
nvetgsaksgqlflvdlaGsekvgktgasgqtleeakkinkslsalgmvinaltdgksshvpyrdskltrilq
eslggnsrttliincspssyndaetlstlrfgmraksiknkakvnaelspaelkqmlakaktq
>1MUC_A
mtsalieridaiivdlptirphklamhtmqqqtlvvlrvrcsdgvegigeattigglaygyespegikanida
hlapaliglaadninaamlkldklakgntfaksgiesalldaqgkrlglpvsellggrvrdslevawtlasgd
tardiaearhmleirrhrvfKlKiganpveqdlkhvvtikrelgdsasvrvdvnqywdesqairacqvlgdng
idlieqpisrinrggqvrlnqrtpapimadesiesvedafslaadgaasifalkiaknggpravlrtaqiaea
agiglyggtmlegsigtlasahafltlrqltwgtElfgplllteeivneppqyrdfqlhiprtpglgltldeq
rlarfarr
>1EC9_A
mssqfttpvvtemqvipvaghdsmlmnlsgahapfftrniviikdnsghtgvgeipggekirktledaiplvv
gktlgeyknvltlvrntfadrdaggrglqtfdlrttihvvtgieaamldllgqhlgvnvasllgdgqqrseve
mlgylffvgnrkatplpyqsqpddscdwyrlrheeamtpdavvrlaeaayekygfndfKlKggvlageeeaes
ivalaqrfpqaritldpngawslneaikigkylkgslayaedpcgaeqgfsgrevmaefrratglptatnmia
tdwrqmghtlslqsvdiplaDphfwtmqgsvrvaqmchefgltwgsHsnnhfdislamfthvaaaapgkitai
Dthwiwqegnqrltkepfeikgglvqvpekpglgveidmdqvmkahelyqkhglgarddamgmqylipgwtfd
nkrpcmvr
>1G0D_A
masykglivdvngrshennlahrtreidrerlivrrgqpfsitlqcsdslppkhhlelvlhlgkrdevvikvq
kehgardkwwfnqqgaqdeilltlhspanavighyrlavlvmspdghiveradkisfhmlfnpwcrddmvylp
desklqeyvmnedgviymgtwdyirsipwnygqfedyvmdicfevldnspaalknsemdiehrsdpvyvgrti
tamvnsngdrgvltgrweepytdgvapyrwtgsvpilqqwskagvrpvkygqCwvfaavactvlrclgiptrp
itnfasahdvdgnlsvdfllnerlesldsrqrsdsswnfHcwveswmsredlpegndgwqvlDptpqelsdge
fccgpcpvaaikegnlgvkydapfvfaevnadtiywivqkdgqrrkitedhasvgknistksvygnhredvtl
hykypegsqkerevykkagrrvtepsneiaeqgrlqlsikhaqpvfgtdfdvivevkneggrdahaqltmlam
avtYnslrrgecqrktisvtvpahkahkevmrlhyddyvrcvsehhlirvkalldapgengpimtvaniplst
pellvqvpgkavvwepltayvsftnplpvplkggvftlegagllsatqihvngavapsgkvsvklsfspmrtg
vrkllvdfdsdrlkdvkgvttvvvhkkyrslitglhtd
>1C2T_A
mnivvlisgngsnlqaiidacktnkikgtvravfsnkadafglerarqagiathtliasafdsreaydrelih
eidmyapdvvvlagfmrilspafvshyagrllNiHpsllpkypglhthrqalengdeehgtSvhfvtdelDgg
pvilqakvpvfagdsedditarvqtqehaiyplviswfadgrlkmhenaawldgqrlppqgyaade
>3PVA_A
Csslsirttddkslfartmdftmepdskviivprnygirllekenvvinnsyafvgmgstditspvlydgvne
kglmgamlYyatfatyadepkkgttginpvyvisqvlgncvtvddviekltsytllneaniilgfapplhytf
tdasgesiviepdktgitihrktigvmtNspgyewhqtnlrayigvtpnppqdimmgdldltpfgqgaglgl

pgdftpsaRflrvaywkkytekaknetegvtnlfhilssvnipkgvvltnegktdytiytsamcaqsknyyfk
lydnsrisavslmaenlnsqdlitfewdrkqdikqlnqvnvms
>1HV9_A
mlnnamsvvilaagkgtRmysdlpkvlhtlagkamvqhvidaanelgaahvhlvyghggdllkqalkddnlnw
vlqaeqlgtghamqqaapffaddedilmlygdvplisvetlqrlrdakpqggigllltvklddptgygritren
gkvtgivehkdatdeqrqiqeintgiliangadmkrwlakltnnnaqgeyyitdiialayqegreivavhpqr
lsevegvnnrlqlsrlervyqseqaeklllagvmlrdparfdlrgtlthgrdveidtnviiegnvtlghrvki
gtgcviknsvigddceispytvvedanlaaactigpfarlrpgaellegahvgnfvemkkarlgkgskaghlt
ylgdaeigdnvnigagtitcnydgankfktiigddvfvgsdtqlvapvtvgkgatiaagttvtrnvgenalai
srvpqtqkegwrrpvkkk
>1DVE_A
merpqldsmsqdlsealkeatkevHiraensefmrnfqkgqvsregfklvmaslyhiYtaleeeiernkqnpv
yaplyfpeelhrraaleqdmafwygphwqeaipytpatqhyvkrlhevggthpellvahayTRylGDlsGgqv
lkkiaqkamalpssgeglafftfpsidnptkfkqlyrarmntlemtpevkhrvteeaktafllnielfeelqa
llteehkdqspsqteflrqrpaslvqdttsaetprgksqistsssqtp
>1I1H_A
peydyirdgnaiyersfaiiraeadlsrfseeeadlavrmvHacgsveatrqfvfspdfvssaraalkagapi
lcdaemvahgvtrarlpagnevictlrdprtpalaaeigntrsaaalklwserlagsvvaignaptalfflle
mlrdgapkpaailgmpvgfvgaaeskdalaensygvpfaivrgrlggsamtaaalnslarpgl
>1EXP_A
attlkeaadgagrdfgfaldpnrlseaqykaiadsefnlvvaenamkwdatepsqnsfsfgagdrvasyaadt
gkelyghtlvwhsqlpdwaknlngsafesamvnhvtkvadhfegkvaswdvvnEafadgggrrqdsafqqklg
ngyietafraaraadptaklcindynveginaksnslydlvkdfkargvpldcvgfqsHlivgqvpgdfrqnl
qrfadlgvdvritElDirmrtpsdatklatqaadykkvvqacmqvtrcqgvtvwgitdkyswvpdvfpgegaa
lvwdasyakkpayaavmeaf
>1QGX_A
malerellvatqavrkaslltkriqsevishkdsttitkndnspvttgDyaaqtiiinaiksnfpddkvvgee
sssglsdafvsgilneikandevynknykkddflftndqfplksledvrqiidfgnyeggrkgrfwcldpidg
Tkgflrgeqfavclalivdgvvqlgcigcpnlvlssygaqdlkghesfgyifravrglgafyspssdaeswtk
ihvrhlkdtkdmitlegvekghsshdeqtaiknklnisksl hldsqakycllalgladvylrlpiklsyqeki
wDhaagnvivheaggihtdamedvpldfgngrtlatkgviassgprelhdlvvstscdviqsrna
>1MNF_A
aakdvkfgndarvkmlrgvnvladavkvtlgpkgrnvvldksfgaptitkDgvsvareieledkfenmgaqmv
kevaskandaagdgTTtatvlaqaiiteglkavaagmnpmdlkrgidkavtaaveelkalsvpcsdskaiaqv
gtisansdetvgkliaeamdkvgkegvitvedgtglqdeldvvegmqfdrgylspyfinkpetgavelespfi
lladkkisniremlpvleavakagkplliiaedvegealatlvvntmrgivkvaavkapgfgdrrkamlqdia
tltggtviseeigmelekatledlgqakrvvinkdtttiidgvgeeaaiqgrvaqirqqieeatsdydreklq
ervaklaggvavikvgaatevemkekkarveDalhatraaveegvvagggvalirvaskladlrgqnedqnvg
ikvalrameaplrqivlncgeepsvvantvkggdgnygynaateeygnmidmgildptkvtrsalqyaasvag
lmittecmvtdlpk
>1RVV_2
mniiqgnlvgtglkigivvgrfndfitskllsgaedallrhgvdtndidvawvpgafeipfaakkmaetkkyd
aiitlgtvirgattHydyvcneaakgiaqaanttgvpvifgivttenieqaieragtkagnkgvdcavsaiem
anlnrsfe
>1BTL_A
hpetlvkvkdaedqlgarvgyieldlnsgkilesfrpeerfpmmStfKvllcgavlsridagqeqlgrrihys
qndlveyspvtekhltdgmtvrelcsaaitmSdntaanlllttiggpkeltaflhnmgdhvtrldrwEpelne
aipnderdttmpvamattlrklltgelltlasrqqlidwmeadkvagpllrsalpagwfiadksgagergsrg
iiaalgpdgkpsrivviyttgsqatmdernrqiaeigaslikhw
>3E7B_A
slnldsiigrllevqgsrpgknvqlteneirglclksreiflsqpilleleaplkicgdihgqyydllrlfey
ggfppesnylflgdyvDRgkqsleticlllaykikypenffllrgNHecasinriygfydeckrryniklwkt
ftdcfnclpiaaivdekifcchgglspdlqsmeqirrimrptdvpdqgllcdllwsdpdkdvqgwgendRgvs

ftfgaevvakflhkhdldlicraHqvvedgyeffakrqlvtlfsapnycgefdnagammsvdetlmcsfqilk
pa
>1LZ1_A
kvfercelartlkrlgmdgyrgislanwmclakwEsgyntratnynagdrstDygifqinsrywcndgktpga
vnachlscsallqdniadavacakrvvrdpqgirawvawrnrcqnrdvrqyvqgcgv
>1MAS_A
akkiildcDpglddavaillahgnpeiellaittvvgnqtlakvtrnaqlvadiagitgvpiaagcdkplvrk
imtaghihgesgmgtvaypaefknkvderhavnliidlvmshepktitlvptggltniamaarleprivdrvk
evvlmgggyhegnatsvaefNiiidpeaahivfneswqvtmvgldlthqalatppilqrvkevdtnparfmle
imdyytkiyqsnrymaaaavHdpcavayvidpsvmttervpvdieltgkltlgmtvadfrnprpehchtqvav
kldfekfwglvldalerigdpq
>2C61_A
gplifvektepvgyneivnikmgdgtvrrgqvldssadivvvqvfftgetlklpasvdllgrilsgsgeprdg
gprivpdqlldingaamnpyarlppkdfiqtgistidgtntlvrgqklpifsasglphneialqiarqasvpg
sesafavvfaamgitneeaqyfmsdfektgaleravvflnladdpaverivtprmaltaaeylayehgmhvlv
iltditnyaealrqmgaarnevpgrrgypgymytdlatlyeragivkgakgsvtqipilsmpgddithpipdl
sgyitegqivvarelhrkgiyppinvlpslsRlmnsgigagktredhkavsdqmyagyaegrdlrglvaivgk
ealserdtkflefadlfedkfvrqgrnenrtiedtleigwqilthlpenqlgridnkyiqkyhpah
>1DHP_A
mftgsivaivtpmdekgnvcraslkklidyhvasgtsaivsvgttgesatlnhdehadvvmmtldladgripv
iagtganataeaisltqrfndsgivgcltvtpyynrpsqeglyqhfkaiaehtdlpqilYnvpsRtgcdllpe
tvgrlakvkniigiKeatgnltrvnqikelvsddfvllsgddasaldfmqlgghgvisvtanvaardmaqmck
laaeghfaearvinqrlmplhnklfvepnpipvkwackelglvatdtlrlpmtpitdsgretvraalkhagll
>1BFD_A
masvhgttyellrrqgidtvfgnpgsnElpflkdfpedfryilalqeacvvgiadgyaqasrkpafinlHsaa
gtgnamgalsnawnshsplivtagqqtramigveallltnvdaanlprplvkwsyepasaaevphamsraihma
smapqgpvylsvpyddwdkdadpqshhlfdrhvsssvrlndqdldilvkalnsasnpaivlgpdvdaananad
cvmlaerlkapvwvapsaprcpfptrhpcfrglmpagiaaisqllleghdvvlvigapvfryHqydpgqylkpg
trlisvtcdpleaarapmgdaivadigamasalanlveessrqlptaapepakvdqdagrlhpetvfdtlndm
apenaiylneststtaqmwqrlnmrnpgsyyfcaagglgfalpaaigvqlaeperqviavigdgsanysisal
wtaaqyniptifvimnngtygalrwfagvleaenvpgldvpgidfralakgygvqalkadnleqlkgslqeal
sakgpvlievstvspvk
>1I29_A
mifsvdkvradfpvlsrevnglplayldsaasaqkpsqvidaeaefyrhgyaavhrgihtlsaqatekmenvr
kraslfinarsaeelvfvrgtteginlvanswgnsnvragdniiisqmehhanivpwqmlcarvgaelrvipl
npdgtlqletlptlfdektrllaithvsnvlgtenplaemitlahqhgakvlvdgaqavmhhpvdvqaldcdf
yvfsghKlygptgigilyvkeallqemppwegggsmiatvslsegttwtkapwrfeagtpntggiiglgaale
yvsalglnniaeyeqnlmhyalsqlesvpdltlygpqnrlgviafnlgkhhaydvgsfldnygiavrtghhCa
mplmayynvpamcraslamyntheevdrlvtglqrihrllg
>1CMX_A
msgenravvpiesnpevftnfahklglknewayfdiysltepellaflprpvkaivllfpinedrksstsqqi
tssydviwfkQsvknaCglyailhslsnnqsllepgsdldnflksqsdtsssknrfddvttdqfvlnvikenv
qtfstgqseapeatadtnlHyityveenggifelDgrnlsgplylgksdptatdlieqelvrvrvasymenan
eedvlnfamlglgpnw
>2PJR_G
gdavmlmtlhaakglefpvvfligmeegifphnrsledddemeeerrlayvgitRaeeelvltsaqmrtlfgn
iqmdppsrflneipahlletas
>1HZF_A
spggvasllrlprgCgeQtiylaptlaasryldkteqwstlppetkdhavdliqkgymriqqfrkadgsyaaw
lsrdsstwltafvlkvlslaqeqvggspeklqetsnwllsqqqadgsfqdpcpvldrsqgglvgndetvalta
fvtialhhglavfqdegaeplkqrveasiskassflgekasagllgahaaaitayaltltkapadlrgvahnn
laaqetgdnlywgqapalwiettayallhlllhegkaeadqasawltrqgsfqggfrstqdtvialdalsayw
iasht
>1QD6_D

ftlypydtnyliytqtsdlnkeaiasydwaenarkdevkfqlslafplwrgilgpnsvlgasytqkswwqlsn
seesspfretnyepqlflgfatdyrfagwtlrdvemgynHdSnGrsdptsrswnrlytrlmaengnwlvevkp
wyvvgntddnpditkymgyyqlkigyhlgdavlsakgqynwntgyggaelglsypitkhvrlytqvysgyges
lidynfnqtrvgvgvmlndlf
>1DXE_A
mnndvfpnkfkaalaakqvqigcwsalsnpistevlglagfdwlvldgeHapndistfipqlmalkgsasapv
vRvptnepviikrlldigfynflipfvetkeeaelavastryppegirgvsvshranmfgtvadyfaqsnkni
tilvqiesqqgvdnvdaiaategvdgifvgpsdlaaalghlgnashpdvqkaiqhifnrasahgkpsgilapv
eadarrylewgatfvavgsdlgvfrsatqkladtfkk
>1AJ0_A
mklfaqgtsldlshphvmgilNvtpdsfsdggthnslidavkhanlminagatiidvggestRpgaaevsvee
elqrvipvveaiaqrfevwisvdtskpeviresakvgahiindirslsepgaleaaaetglpvclmhmqgnpk
tmqeapkyddvfaevnryfieqiarceqagiakeklllldpgfgfgknlshnysllarlaefhhfnlpllvgms
rksmigqllnvgpserlsgslacaviaamqgahiiRvhdvketveamrvveatlsakenkrye
>1GTP_S
pslskeaalvhealvargletplrppvhemdnetrksliaghmteimqllnldladdslmetphriakmyvde
ifsgldyanfpkitlienkmkvdemvtvrditltstceHhfvtidgkatvayipkdsviglskinrivqffaq
rpqvqerltqqilialqtllgtnnvavsidavHycvkargirdatsattttslgglfkssqntrheflravrh
hn
>3PCA_N
paqdnsrfvirdrnwhpkaltpdyktsiarsprqalvsipqsisettgpnfshlgfgahdhdlllnfglpige
riivagrvvdqygkpvpntlvemwqanaggryrhkndrylapldpnfggvgrcltdsdgyysfrtikpgpYpw
rngpndwRpahihfgisgpsiatklitqlyfegdplipmcpivksianpeavqqliakldmnnanpmdclayr
fdivlrgqrkthfe
>1CG6_A
masgttttavkigiiggtglddpeilegrtekyvdtpfgkpsdalilgkiknvdcvllarhgrqhtimpskvn
yqaniwalkeegcthvivttacgslreeiqpgdiviidqfidrttmrpqsfydgshscargvchipmaepfcp
ktrevlietakklglrchskgtmvtiegprfssraesfmfrtwgadvinmttvpevvlakeagicyasiamat
DyDcwkeheeavsvdrvlktlkenankakslllttipqigstewsetlhnlknmaqfsvllprh
>1K0W_A
mledlkrqvleanlalpkhnlvtltwgnvsavdrergvfvikpsgvdysimtaddmvvvsietgevvegakkp
ssdtpthrllyqafpsiggivhthsrhatiwaqagqsipatgtthaNyfygtipctrkmtdaeingeyewetg
nvivetfekqgidaaqmpgvlvhshgpfawgknaedavhnaivleevaymgifcrqlapqlpdmqqtllnkhy
lrkhgakayygq
>1I40_A
sllnvpagkdlpediyvvieipanadpikyeidkesgalfvdrfmstamfypcnygyinhtlsldgDpvdvlv
ptpyplqpgsvtrcrpvgvlkmtdeagedaklvavphsklskeydhikdvndlpellkaqiahffehykdlek
gkwvkvegwenaeaakaeivasferaknk
>1I1E_A
pvtinnfnyndpidnnniimmeppfargtgryykafkitdriwiiperytfgykpedfnkssgifnrdvceyy
dpdylntndkkniflqtmiklfnrikskplgekllemiingipylgdrrvpleefntniasvtvnklisnpge
verkkgifanliifgpgpvlnenetidigiqnhfasregfggimqmkfcpeyvsvfnnvqenkgasifnrrgy
fsdpalilmhelihvlhglygikvddlpivpnekkffmqstdaiqaeElytfggqdpsiitpstdksiydkvl
qnfrgivdrlnkvlvcisdpnininiyknkfkdykfvedsegkysidvesfdklykslmfgftetniaenyk
iktRasYfsdslppvkiknlldneiytieegfnisdkdmekeyrgqnkainkqayeeiskehlavykiqmcks
vkapgicidvdnedlffiadknsfsddlsknerieyntqsnyiendfpinelildtdliskielpsenteslt
dfnvdvpvyekqpaikkiftdentifqylysqtfpldirdisltssfddallfsnkvysffsmdyiktankvv
eaglfagwvkqivndfvieanksntmdkiadislivpyiglalnvgnetakgnfenafeiagasillefipel
lipvvgafllesyidnknkiiktidnaltkrnekwsdmyglivaqwlstvntqfytikegmykalnyqaqale
eiikyryniysekeksninidfndinsklneginqaidninnfingcsvsylmkkmiplaveklldfdntlkk
nllnyidenklyligsaeyekskvnkylktimpfdlsiytndtiliemfnkynseilnniilnlrykdnnlid
lsgygakvevydgvelndknqfkltssanskirvtqnqniifnsvfldfsvsfwiripkykndgiqnyihney
tiincmknnsgwkisirgnriiwtlidingktksvffeynirediseyinrwffvtitnnlnnakiyingkle
sntdikdireviangeiifkldgdidrtqfiwmkyfsifntelsqsnieerykiqsyseylkdfwgnplmynk

eyymfnagnknsyiklkkdspvgeiltrskynqnskyinyrdlyigekfiirrksnsqsinddivrkedyiyl
dffnlnqewrvytykyfkkeeeklflapisdsdefyntiqikeydeqptyscqllfkkdeestdeigligihr
fyesgivfeeykdyfciskwylkevkrkpynlklgcnwqfipkdegwte
>1KL7_A
mpnasqvyrstrssspktisfeeaiiqglatdgglfipptipqvdqatlfndwsklsfqdlafaimrlyiaqe
eipdadlkdlikrsystfrsdevtplvqnvtgdkenlhilelfhgptyafKdvalqfvgnlfeyflqrtnanl
pegekkqitvvgatsgdtgsaaiyglrgkkdvsvfilyptgrispiqeeqmttvpdenvqtlsvtgtfdncqd
ivkaifgdkefnskhnvgavnsinwarilaqmtyyfysffqatngkdskkvkfvvpsgnfgdilagyfakkmg
lpieklaiatnendildrflksglyersdkvaatlspamdilissnferllwylareylangddlkageivnn
wfqelktngkfqvdksiiegaskdftservsneetsetikkiyessvnpkhyildphtavgvcaterliakdn
dksiqyislstahpakfadavnnalsgfsnysfekdvlpeelkklstlkkklkfieradvelvknaieeelak
mkl
>1MDR_A
msevlitglrtravnvplaypvhtavgtvgtaplvlidlatsagvvghsylfaytpvalkslkqllddmaami
vneplapvsleamlakrfclagytglirmaaagidmaawdalgkvhetplvkllganarpvqaydshsldgvk
lateravtaaelgfravktKigypaldqdlavvrsirqavgddfgimvdynqsldvpaaikrsqalqqegvtw
ieeptlqhdyeghqriqsklnvpvqmgenwlgpeemfkalsigacrlampDamkiggvtgwirasalaqqfgi
pmssHlfqeisahllaatptahwlErldlagsvieptltfeggnavipdlpgvgiiwrekeigkylv
>1LNH_A
mlggllhrghkikgtvvlmrknvldvnsvtsvggiigqgldlvgstldtltaflgrsvslqlisatkadangk
gklgkatflegiitslptlgagqsafkinfewddgsgipgafyiknfmqtefflvsltledipnhgsihfvcn
swiynaklfksdriffanqtylpsetpaplvkyreeelhnlrgdgtgerkeweriydydvyndlgdpdkgenh
arpvlggndtfpyprrgrtgrkptrkdpnsesrsndvylprdeafghlkssdfltyglksvsqnvlpllqsaf
dlnftprefdsfdevhglysggiklptdiiskisplpvlkeifrtdgeqalkfpppkviqvsksawmtdeefa
remlagvnpnlirclkdfpprskldsqvygdhtsqitkehlepnlegltvdeaiqnkrlflldhhdpimpylr
rinatstkayatrtilflkndgtlrplaielslphpqgdqsgafsqvflpadegvessiwllakayvvvndsc
yhqlvshwlnthavvepfiiatnrhlsvvhpiykllhphyrdtmninglarlslvndggvieqtflwgrysve
msavvykdwvftdqalpadlikrgmaiedpscphgirlviedypytvdgleiwdaiktwvheyvflyyksddt
lredpelqacwkelvevghgdkknepwwpkmqtreelveacaiiiwtasalhaavNfgqypygglilnrptls
rrfmpekgsaeyeelrknpqkaylktitpkfqtlidlsvieilsrhasdevylgerdnpnwtsdtraleafkr
fgnklaqienklsernndeklrnrcgpvqmpytlllpsskegltfrgipnsisi
>1MUW_A
syqptpedrftfglwtvgwqgrdpfgdatrpaldpvetvqrlaelgahgvtfHddDlipfgssdtereshikr
frqaldatgmtvpmattnlfthpvfkdggftandrdvrryalrktirnidlavelgaktyvawggregaesga
akdvrvaldrmkeafdllgeyvtsqgydirfaiEpKpneprgdillptvghalafierlerpelygvnpevgH
eqmaglnfphgiaqalwagklfhidlngqsgikyDqdlrfgagdlraafwlvdllesagyegprhfdfkpprt
edidgvwasaagcmrnylilkeraaafradpevqealrasrldelaqptaadgvqelladrtafedfdvdaaa
argmaferldqlamdhllgar
>1JQN_A
mneqysalrsnvsmlgkvlgetikdalgehilervetirklskssragndanrqellttlqnlsndellpvar
afsqflnlantaeqyhsispkgeaasnpeviartlrklknqpelsedtikkaveslslelvltaHpteitrrt
lihkmvevnaclkqldnkdiadyehnqlmrrlrqliaqswhtdeirklrpspvdeakwgfavvenslwqgvpn
ylrelneqleenlgyklpvefvpvrftswmggdrdgnpnvtaditrhvlllsrwkatdlflkdiqvlvselsm
veatpellalvgeegaaepyrylmknlrsrlmatqawlearlkgeelpkpeglltqneelweplyacyqslqa
cgmgiiangdlldtlrrvkcfgvplvridiRqestrhtealgeltrylgigdyeswseadkqaflirelnskr
pllprnwqpsaetrevldtcqviaeapqgsiaayvismaktpsdvlavhlllkeagigfampvaplfetlddl
nnandvmtqllnidwyrgliqgkqmvmigysdsakdagvmaaswaqyqaqdaliktcekagieltlfhgRggs
igrggapahaallsqppgslkgglrvteqgemirfkyglpeitvsslslytgaileanllpppepkeswrrim
delsviscdvyrgyvrenkdfvpyfrsatpeqelgklplgsRpakrrptggveslRaipwifawtqnrlmlpa
wlgagtalqkvvedgkqseleamcrdwpffstrlgmlemvfakadlwlaeyydqrlvdkalwplgkelrnlqe
edikvvlaiandshlmadlpwiaesiqlrniytdplnvlqaellhrsrqaekegqepdprveqalmvtiagia
agmrntg
>1MKA_A

vdkresytkedllasgrgelfgakgpqlpapnmlmmdrvvkmtetggnfdkgyveaeldinpdlwffgcHfig
dpVmpGClglDamwqlvgfylgwlggegkgralgvgevkftgqvlptakkvtyrihfkrivnrrlimgladge
vlvdgrliytasdlkvglfqdtsaf
>1NSJ_A
mvrvkiCgitnledalfsvesgadavgfvfypkskryispedarrisvelppfvfrvgvfvneepekildvas
yvqlnavqlhgeepielcrkiaerilvikavgvsnerdmeralnyrefpillDtktpeyggsgktfdwslilp
yrdrfrylvlsgglnpenvrsaidvvrpfavdvssgveafpgkkdhdsikmfiknakgl
>1B5D_A
misdsmtveeirlhlglalkekdfvvdktgvktieiigasfvadepfifgalndeyiqrElewykskslfvkd
ipgetpkiwqqvasskgeinsnygwaiwsednyaqydmclaelgqnpdsrrgimiytrpsmqfdynkdgmsdf
mCtntvqylirdkkinavvnmrsndvvfgfrnDyawqkyvldklvsdlnagdstrqykagsiiwnvgslhvys
rhfylvdhwwktgethiskkdyvgkya
>2R11_C
elsdngiryyqtyneslslwpvrcksfyistrfgqthviasgpedapplvllhgAlfsstwypniadwsskyr
tyavdiigdknksipenvsgtrtdyanwlldvfdnlgiekshiglSLgglhtnfllrpervksaailsPaetf
lpfhhdfykyalgltasngvetflnwndqnvlhpifvkqfkagvwqdgsrnpnpnadgfpyvftdeelrsarv
pillllgeHEviydphsalhrassfvpdieaeviknagHvlseqptyvnervrffna
>3DAY_A
lqwghqevpakfnfasdvldhwadmekagkrppspalwwvngkgkelmwnfrelsensqqaanvlsgacglqr
gdrvavvlprvpewwlvilgciraglifmpgtiqmkstdilyrlqmskakaivagdeviqevdtvasecpslr
ikllvsekscdgwlnfkkllneastthhcvetgsqeasaiyftsgtsglpkmaehsysslglkakmdagwtgl
qasdimwtisdtgwilnilcslmepwalgactfvhllpkfdplvilktlssypiksmmgapivyrmllqqdls
sykfphlqncvtvgesllpetlenwraqtgldiresygqTetgltcmvsktmkikpgymgtaascydvqiidd
kgnvlppgtegdigirvkpirpigifsgyvdnpdktaanirgdfwllgdrgikdedgyfqfmgraddiiNssg
yRigpsevenalmehpavvetavisspdrgevvkafvvlasqflshdpeqltkelqqhvksvtapykyprkie
fvlnlpktvtgKiqraklrdkewk
>1EI5_A
mskfdtsaleafvrhipqnykgpggvvavvkdgevvlqhawgfadlrtrtpmtldtrmpicSvsKqftcavll
davgepellddaleayldkfederpavrdlcnnqsglrdywalsvlcgadpegvflpaqaqsllrrlktthfe
pgshysYcNgnfriladlieahtgrtlvdilserifapagmkraelisdtalfdectgyegdtvrgflpatnr
iqwmgdagicaslndmiaweqfidatrddesglyrrlsgpqtfkdgvaapygfglnlhetggkrltgHggalr
gwrcqrwhcaderlstiamfnfeggasevafklmnialgvsssevsrveadsawfgswlddetglvlsledag
hgrmkarfgtspemmdvvsanearsavttirrdgetielvrasenlrlsmkrvkgeakhdiigryhsdeldad
lllvseggaiygafegflgksdmyplysvgsdvwllpvqrsmdapspgewklvfrrddkgeitglsvgcwlar
gveyrrvqp
>1QBA_A
dqqlvdqlsqlklnvkmldnragengvdcaalgadwascnrvlftlsndgqaidgkdwviyfhsprqtlrvdn
dqfkiahltgdlykleptakfsgfpagkaveipvvaeywqlfrndflprwyatsgdakpkmlantdtenldqf
vapftgdqwkrtkddknilmtpasrfvsnadlqtlpagalrgkivptpmqvkvhaqdadlrkgvaldlstlvk
paadvvsqrfallgvpvqtngypiktdiqpgkfkgamavsgayelkigkkeaqvigfdqagvfyglqsilslv
psdgsgkiatldasdaprfpyrgifldvarnfhkkdavlrlldqmaayklnkfhfhlsddegwrieipglpel
tevggqrchdlsettcllpqygqgpdvyggffsrqdyidiikyaqarqievipeidmpaharaavvsmearyk
klhaagkeqeanefrlvdptdtsnttsvqffnrqsylnpcldssqrfvdkvigeiaqmhkeagqpiktwhfgg
DEaknirlgagytdkakpepgkgiidqgnedkpwaksqvcqtmikegkvadmehlpsyfgqevsklvkahgid
rmqawqdglkdaesskafatsrvgvnfwdtlywggfdsvndwankgyevvvsnpdyvymdfpyevnpdergyy
wgtrfsderkvfsfapdnmpqnaetsvdrdgnhfnaksdkpwpgayglsaqlwsetqrtdpqmeymifprals
vaerswhragweqdyragreykggethfvdtqalekdwlrfanilgqrelakldkggvayrlpvpgarvaggk
leanialpglgieystdggkqwqrydakakpavsgevqvrsvspdgkrysraekv
>1BT1_A
apiqapeiskcvvppadlppgavvdnccppvasnivdyklpavttmkvrpaahtmdkdaiakfakavelmkal
paddprnfyqqalvhcaycnggydqvnfpdqeiqvhnswlffpfhrwylyfyerilgkligdpsfglpfwnwd
npggmvlpdflndstsslydsnrnqshlppvvvdlgyngadtdvtdqqritdnlalmykqmvtnagtaelflg
kayragdapspgagsiEtsphipihrwvgdprntnnedmgnfysagrdiafychhsnvdrmwtiwqqlagkpr
krdytdsdwlnatflfydengqavkvrigdsldnqkmgykyaktplpwldskp

```
>1PT0_B
mirtmlqgklhrvkvthadlhyegsacaidqdfldaagileneaidiwnvtngkrfstYaiaaergsriisvn
gaaahcasvgdiviiasfvtmpdeeartwrpnvayfegdnemk
>1XIK_A
ayttfsqtkndqlkepmffgqpvnvarydqqkydifekliekqlsffwrpeevdvsrdridyqalpehekhif
isnlkyqtlldsiqgrspnvallplisipeletwvetwafsetihsrsYthiirnivndpsvvfddivtneqi
qkraegissyydeliemtsywhllgegthtvngktvtvslrelkkklylclmsvnaleairfyvsfacsfafa
erelmegnakiirliardealhltgtqhmlnllrsgaddpemaeiaeeckqecydlfvqaaqqekdwadylfr
dgsmiglnkdilcqyveyitnirmqavgldlpfqtrsnpipwintwlvsdnvqvapqevevssylvgqidsev
dtddlsnfql

//Dtestset200//
>1K32_D
mpnlllnpdihgdriifvccddlwehdlksgstrkivsnlgvinnarffpdgrkiairvmrgsslntadlyfy
ngengeikrityfsgkstgrrmftdvagfdpdgnliistdamqpfssmtclyrvendginfvplnlgpathil
fadgrrvigrntfelphwkgyrggtrgkiwievnsgafkkivdmsthvsspvivghriyfitdidgfgqiyst
dldgkdlrkhtsftdyyprhlntdgrrilfskggsiyifnpdtekiekieigdlespedriisipskfaedfs
pldgdliafvsrgqafiqdvsgtyvlkvpeplriryvrrggdtkvafihgtregdflgiydyrtgkaekfeen
lgnvfamgvdrngkfavvandrfeimtvdletgkptviersreamitdftisdnsrfiaygfplkhgetdgyv
mqaihvydmegrkifaattenshdyapafdadsknlyylsyrsldpspdrvvlnfsfevvskpfviplipgsp
nptklvprsmtseageydlndmykrsspinvdpgdyrmiiplessiliysvpvhgefaayyqgapekgvllky
dvktrkvtevknnltdlrlsadrktvmvrkddgkiytfplekpedertvetdkrplvssiheeflqmydeawk
lardnywneavakeiseriyekyrnlvplcktrydlsnvivemqgeyrtsHsyemggtftdkdpfrsgriacd
fkldgdhyvvakayagdysnegekspifeygidptgyliedidgetvgagsniyrvlsekagtsarirlsgkg
gdkrdlmidilddrfiryrswveanrryvherskgtigyihipdmgmmglnefyrlfinessyqglivdvrf
nggGfvsqliieklmnkrigydnprrgtlspyptnsvrgkiiaitneyagSDgdifsfsfkklglgkligtrt
wggvvgitpkrrlidgtvltqpefafwfrdagfgvenygvdpdveieyaphdylsgkdpqidyaidalieelr
n
>1A4L_A
tpafnkpkvelhvhldgaikpetilyfgkkrgialpadtveelrniigmdkplslpgflakfdyympviagcr
eaikriayefvemkakegvvyvevrysphllanskvdpmpwnqtegdvtpddvvdlvnqglqegeqafgikvr
silccmrhqpswslevlelckkynqktvvamdlagdetiegsslfpghveayegavkngihrtvhagEvgspe
vvreavdilktervgHgYhtiedealynrllkenmhfevcpwssyltgawdpktthavvrfkndkanyslntD
dplifkstldtdyqmtkkdmgfteeefkrlninaakssflpeeekkellerlyreyq
>2NLR_A
dtticepfgtttiqgryvvqnnrwgstapqcvtatdtgfrvtqadgsaptngapksypsvfngchytncspgt
dlpvrldtvsaapssisygfvdgavynasydiwldptartdgvnqtEimiwfnrvgpiqpigspvgtasvggr
twevwsggngsndvlsfvapsaisgwsfdvmdfvratvarglaendwyltsvqagfepwqngaglavnsfsst
vetgtpggtdpgdpg
>1INP_A
msdilqellrvsekaaniaracrqqetlfqllieekKegeknkkfavdfktladvlvqevikenmenkfpglg
kkifgeesneltndlgekiimrlgpteeetvallskvlngnklasealakvvhqdvffsdpaldsveinipqd
ilgiwvdpidsTyqyikgsaditpnqgifpsglqcvtvligvydiqtgvplmgvinqpfvsqdlhtrrwkgqc
ywglsylgtnihsllppvstrsnseaqsqgtqnpssegscrfsvvistseketikgalshvcgerifraagag
ykslcvilgladiyifsedttfkwdscaahailramgggmvdlkeclernpdtgldlpqlvyhvgnegaagvd
qwankggliayrsekqletflsrllqhlapvatht
>1YID_B
arprvltgdrptgalhlghlagslqnrvrlqdeaelfvlladvqaltdhfdrpeqvrenvlavaldylaagld
pqkttcvvqsavpelaeltvyflnlvtvshlrqnptvkaeiaqkgygervpagffvypvsqaadiaafgatlv
pvgddqlpmleqtreivrrfnalyapvlaepqaqlsrvprlpgldgqaKmsKslgnaialgdsadevarkvmg
mytdpghlrasdpgrvegnpvftfldafdpdparvqalkdqyragglgdvkvkkhlidvlngvlapirtrrae
yerdpdavlrfvtegtargrevaaqtlgqvrramrlfgh
>2C47_C
```

```
frvgkkielrlgknlytneyvaiklepiksrapqlhleyrfykqlsategvpqvyyfgpgkynamvlellgps
ledlfdlcdrtftlktvlmiaiqlitrmeyvhtksliyrDvKpeNflvgrpgqhaihiidfglakeyidpetk
khipyrehksltgTarymsinthlgkeqsrrddlealghmfmyflrgslpwqglkadtlkeryqkigdtkrat
pievlcenfpeematylryvrrldffekpdydylrklftdlfdrsgfvfdyeydwagkplptp
>1J7G_A
mialiqrvsqakvdvkgetigkigkgllvllgvekednrekadklaekvlnyrifsdendkmnlnvqqaqgel
livsQFTlaadtqkglrpsfskgaspalanelyeyfiqkcaeklpvstgqfaadmqvsltndgpvtfwlnv
>1SGR_E
isggdaiysstgrcslgfnvrsgstyyfltagHctdgattwwansarttvlgttsgssfpnnDygivrytntt
ipkdgtvggqditsaanatvgmavtrrgsttgthsgsvtalnatvnygggdvvygmirtnvcaepGdSggply
sgtraigltSggsgncssggttffqpvtealvaygvsvy
>2DW7_O
svrivdvreitkpisspirnayidftkmttslvavvtdvvregkrvvgygfnsNgrygqgglirerfasrile
adpkkllneagdnldpdkvwaamminekpgghgersvavgtidmavwdavakiagkplfrllaerhgvkanpr
vfvyaaggyyypgkglsmlrgemrgyldrgynvvKmKiggapieedrmrieavleeigkdaqlavdangrfnl
etgiayakmlrdyplfwyeevgdpldyalqaalaefypgpmatgenlfshqdarnllryggmrpdrdwlqfDc
alsyglceyqrtlevlkthgwspsrcipHgghqmslniaaglglggnEsypdlfqpyggfpdgvrvenghitm
pdlpgigfegksdlykemkalae
>1DCI_C
ayesiqvtsaqkhvlhvqlnrpekrnamnrafwrelvecfqkiskdsdcravvvsgagkmftsgidlmdmasd
ilqppgddvariawylrdlisryqktftviekcpkpviaaihggcigGgvDlisacdiryctqdaffqvkEvd
vglaaDvgtlqrlpkvignrslvneltftarkmmadealdsglvsrvfpdkdvmlnaafalaadissksp vav
qgskinliysrdhsvdesldymatwnmsmlqtqdiiksvqaamekkdsksitfskl
>3O8O_G
kkkiavmtsgGdspgmnaavravvrtgihfgcdvfavyegyegllrggkylkkmawedvrgwlseggtligta
RSmefrkregrrqaagnlisqgidalvvcggdgsltgadlfrhewpslvdelvaegrftkeevapyknlsivg
lvGSiDndmsgtdstigaysalericemvdyidatakshsrafvvevmgRhcgwlalmagiatgadyifiper
avphgkwqdelkevcqrhrskgrrnntiivaegalddqlnpvtandvkdalielgldtkvtilghvqrggtav
ahdrwlatlqgvdavkavleftpetpspligilenkiirmplvesvkltksvataienkdfdkaislrdtefi
elyenflsttvkddgsellpvsdrlnigivhvgapsaalnaatraatlyclshghkpyaimngfsgliqtgev
kelswidvenwhnlggseigtnrsvasedlgtiayyfqknkldgliilggfegfrslkqlrdgrtqhpifnip
mclipatvsnnvpgteyslgvdtclnalvnytddikqsasatrrrvfvcevqgghsgyiasftglitgavsvy
tpekkidlasireditllkenfrhdkgenrngkllvrneqassvystqlladiiseaskgkfgvrtaipghvq
qggvpsskdrvtasrfavkcikfieqwnkkneeddsaavicvngshvsfkpianlwenetnvelrkgfevhwa
eynkigdilsgrlklr
>1OTG_A
PhfivecsdnireeadlpglfakvnptlaatgiFplagiRsrvhwvdtwqmadgqhdyafvhmtlkigagrsl
esrqqagemlfelikthfaalmesrllalsfeieelhptlnfkqnnvhalfk
>3PRJ_B
mfeikkiccigagyvggptcsviahmcpeirvtvvdvnesrinawnsptlpiyepglkevvescrgknlffst
niddaikeadlvfisvntptktygmgkgraadlkyieacarrivqnsngykivteksTvpvraaesirrifda
ntkpnlnlqvlsnpeflaEgtaikdlknpdrvliggdetpegqravqalcavyehwvprekilttntwssels
KlaaNaflaqrissinsisalceatgadveevataigmdqrignkflkasvgfggsCfqkDvlnlvylcealn
lpevarywqqvidmndyqrrrfasriidslfntvtdkkiailgfafkkdtgdtresssiyiskylmdegahlh
iydpkvpreqivvdlsdqvsrlvtiskdpyeacdgahavvictewdmfkeldyerihkkmlkpafifdgrrvl
dglhnelqtigfqietigkkv
>1E2A_A
mnreemtllgfeivayagdarskllealkaaengdfakadslvveagsciaeahssqtgmlareasgeelpys
vtmmHgQDHlmttillkdvihhlielykrgak
>1JS4_B
epafnyaealqksmffyeaqrsgklpennrvswrgdsglndgadvgldltggwyDagDhvkfgfpmaftatml
awgaiespegyirsgqmpylkdnlrwvndyfikahpspnvlyvqvgdgdadhkwwgpaevmpmerpsfkvdps
cpgsdvaaetaaamaassivfadddpayaatlvqhakqlytfadtyrgvysdcvpagafynswsgyqdelvwg
aywlykatgddsylakaeyeydflsteqqtdlrsyrwtiawddksygtyvllaketgkqkyiddanrwldywt
```

```
vgvngqrvpyspggmavldtwgalryaantafvalvyakviddpvrkqryhdfavrqinyalgdnprnssyvv
gfgnnpprnphhrtahgswtdsiaspaenrhvlygalvggpgspndaytddrqdyvanEvatdynagfssala
mlveeyggtpladfppteepdgpeifveaqintpgttfteikamirnqsgwparmldkgtfrywftldegvdp
aditvssaynqcatpedvhhvsgdlyyveidctgekifpggqsehrrevqfriaggpgwdpsndwsfqgigne
lapapyivlyddgvpvwgtap
>2EBN_A
avtgttkaniklfsftevndtnplnnlnftlknsgkplvdmvvlfsaninydaandkvfvsnnpnvqhlltnr
akylkplqdkgikvilsilgnhdrsgianlstarakafaqelkntcdlynldgvffDdEysayqtpppsgfvt
psnnaaarlayetkqampnklvtvyvysrtssfptavdgvnagsyvdyaihdyggsydlatnypglaksgmvm
ssqefnqgryataqalrnivtkgygghmifamdpnrsnftsgqlpalkliakelygdelvysntpyskdw
>2JJD_E
kyfpipvehleeeirirckqfreefnslpsghiqgtfelankeenreknrypnilpndhsrvilsqlgipcsd
yinasyidgykeknkfiaaqgpkqetvndfwrmvweqksativmltnlkerkeekchqywpdqgcwtygnirv
cvedcvvlvdytirkfciqpkaprlvsqlhftswpDfgvpftpigmlkflkkvktlnpvhagpivvhCsagvg
RTgtfividammammhaeqkvdvfefvsrirnqrpqmvqtdmqytfiyqalleyylygdteldvssgleeefr
kltnvrimkenmrtgnlpanmkkarviqiipydfnrvilsmytdyinasfidgyrqkdyfiatqgplahtved
fwrmiwewkshtivmltevqEreqdkcyqywptegsvthgeitieikndtlseaisirdflvtlngeqvrvvr
qfhfhgwpEigipaegkgmidliaavqkqqqtgnhpitvHCsagagRTgtfialsnilervkaeglldvfqa
vkslrlqrphmvqtleqyefcykvvqdfid
>1LBU_A
dgcytwsgtlsegssgeavrqlqirvagypgtgaqlaidgqfgpatkaavqrfqsayglaadgiagpatfnki
yqlqdddctpvnftyaelnrcnsdwsggkvsaataranalvtmwklqamrhamgdkpitvnggfrsvtcnsnv
ggasnsrhmyghaadlgagsqgfcalaqaarnhgfteilgpgypgHndhthvaggdgrfwsapscgi
>3NNE_G
mhidnienlsdrefdyivvgggsagaavaarlsedpavsvalveagpddrgvpevlqldrwmellesgydwdy
piepqengnsfmrharakvmggcsshnaciafwapredldeweakygatgwnaeaawplykrletnedagpda
phhgdsgpvhlmnvppkdptgvalldaceqagiprakfntgttvvnganffqinrradgtrssssvsyihpiv
eqenftlltglrarqlvfdadrrctgvdivdsafghthrltarnevvlstgaidtpkllmlsgigpaahlaeh
gievlvdspgvgehlqdhpegvvqfeakqpmvaestqwweigiftptedgldrpdlmmhygsvpfdmntlrhg
ypttengfsltpNvtharsrgtvrlrsrdfrdkpmvdpryftdpeghdmrvmvagirkareiaaqpamaewtg
relspgveaqtdeelqdyirkthntvyHpvgtvrmgavedemspldpelrvkgvtglrvadasvmpehvtvNp
nitvmmigercadlirsa
>1B66_A
vglrrrarlsrlvsfsashrlhspslsaeenlkvfgkCnnpnghghnykvvvtihgeidpvtgmvmnltdlke
ymeeaimkplDHknldldvpyfadvvsttenvavyiwenlqrllpvgalykvkvyEtdnnivvykge
>1L1R_A
mtmsvadahaliktipdfptkgiafkdlsdilstpaaldavrkevtahykdvpitkvvgiesRgfilggivan
slgvgfvalrkagklpgdvckctfdmEyqkgvtievqkrqlgphdvvllhddvlatggtllaaielcetagvk
peniyinvlyeiealkgrekvgqkctrlfsvirehhhhhh
>1SLM_A
ypldgaargedtsmnlvqkylenyydlkkdvkqfvrrkdsgpvvkkiremqkflglevtgkldsdtlevmrkp
rcgvpdvghfrtfpgipkwrkthltyrivnytpdlpkdavdsavekalkvweevtpltfsrlyegeadimisf
avrehgdfypfdgpgnvlahayapgpgingdahfdddeqwtkdttgtnlflvaahEighslglfhsantealm
yplyhsltdltrfrlsqddingiqslygpppdspet
>1Q6X_A
mpilekapqkmpvkassweeldlpklpvpplqqtlatylqcmqhlvpeeqfrksqaivkrfgapgglgetlqe
kllerqektanwvseywlndmYlnnrlalpvnssPavifarqhfqdtndqlrfaaclisgvlsyktlldshsl
ptdwakgqlsgqplcmkqyyrlfssyrlpghtqdtlvaqkssimpepehvivaccnqffvldvvinfrrlseg
dlftqlrkivkmasnederlppiglltsdgrsewakartvllkdstnrdsldmierciclvcldgpgtgelsd
thralqllhgggcslnganrwydkslqfvvgrdgtcgvvceHspfdgivlvqctehllkhmmtsnkklvrads
vselpaprrlrwkcspetqghlassaeklqrivknldfivykfdnygktfikkqkyspdgfiqvalqlayyrl
yqrlvptyesasirrfqegrvdnirsatpealafvqamtdhkaampaseklqllqtamqaqteytvmaitgma
idnhllalrelardlckeppemfmdetylmsnrfvlstSqvpttmemfccygpvvpngygacynpqpeaitfc
issfhscketssvefaeavgaslvdmrdlcssrqpadskppapkekargptkpsnldssh
```

```
>1U8V_C
mlmtaeqyieslrklntrvymfgekienwvdhpmirpsincvrmtyelaqdpqyadlmttksnligktinrfa
nlhqstddlrkkvkmqrllgqktascfqrcvgmdafnavfsttyeidqkygtnyhknfteylkyiqendlivd
gamtdpkgdrglapsaqkdpdlflrivekredgivvrgakahqtgsinshehiimptiamteadkdyavsfac
psdadglfmiygrqscdtrkmeegadidlgnkqfggqealvvfdnvfipndriflcqeydfagmmverfagyH
rqsyggckvgvgdvvigaaalaadyngaqkashvkdkliemthlnetlyccgiacsaegyptaagnyqidlll
anvckqnitrfpyeivrlaediagglmvtmpseadfksetvvgrdgetigdfcnkffaaaptcteermrvlr
fleniclgasavgyrtesmhgagspqaqrimiarqgninakkelakaiagik
>1EH6_A
mdkdcemkrttldsplgklelsgceqglheikllgkgtsaadavevpapaavlggpeplmqctawlnayfhqp
eaieefpvpalhhpvfqqesftrqvlwkllkvvkfgevisyqqlaalagnpkaaravggamrgNpvpilipCH
rvvcssgavgnysgglavkewllahEghrlgkpglggssglagawlkgagatsgsppagrn
>1UOK_A
mekqwwkesvvyqiyprsfmdsngdgigdlrgiiskldylkelgidviwlspvyespnddngydisdyckimn
efgtmedwdellhemhernmklmmdlvvnhtsdehnwfiesrkskdnkyrdyyiwrpgkegkepnnwgaafsg
sawqydemtdeyylhlfskkqpdlnwdnekvrqdvyemmkfwlekgidgfrmDvinfiskeeglptveteeeg
yvsghkhfmngpnihkylhemneevlshydimtvgEmpgvtteeaklytgeerkelqmvfqfehmdldsgegg
kwdvkpcslltlkenltkwqkalehtgwnslywnnhDqprvvsrfgndgmyriesakmlatvlhmmkgtpyiy
qgeeigmtnvrfesideyrdietlnmykekvmergediekvmqsiyikgrdnartpmqwddqnhagfttgepw
itvnpnykeinvkqaiqnkdsifyyykklielrknneivvygsydlilennpsifayvrtygvekllvianft
aeecifelpedisysevellihnydvengpienitlrpyeamvfklk
>1TRP_A
acdytcgsncysssdvstaqaagyklhedgetvgsnsYpHkynnwegfdfsvsspyyEypilssgdvysggsp
gadRvvfnennqlagvitHtgasgnnFvect
>1KEI_A
itgtstvgvgrgvlgdqkninttystyyylqdntrgngiftydakyrttlpgslwadadnqffasydapavda
hyyagvtydyyknvhnrlsydgnnaairssvhysqgynnafwngsqmvygdgdgqtfiplsggidvvahElth
avtdytagliyqnesgaineaisdifgtlvefyanknpdweigedvytpgisgdslrsmsdpakygdpdhysk
rytgtqdnggvHinsgiinkaaylisqggthygvsvvgigrdklgkifyraltqyltptsnfsqlraaavqsa
tdlygstsqevasvkqafdavgvk
>1AKD_A
ttetiqsnanlaplpphvpehlvfdfdmynpsnlsagvqeawavlqesnvpdlvwtrcngghwiatrgqlire
ayedyrhfssecpfipreageaydfiptsmdppeqrqfralanqvvgmpvvdklenriqelacslieslrpqg
qcnftedyaepfpirifmllaglpeediphlkyltdqmtrpdgsmtfaeakealydylipiieqrrqkpgtda
isivangqvngrpitsdeakrmcglllvgglDTvvnflsfsmeflakspehrqeliqrperipaaceellrrf
slvadgriltsdyefhgvqlkkgdqillpqmlsglderenacpmhvdfsrqkvshttfghgshlclgqhlarr
eiivtlkewltripdfsiapgaqiqhksgivsgvqalplvwdpattkav
>1NU3_A
maskieqprwaskdsaagaastpdekivlefmdaltsndaaklieyfaedtmyqnmplppaygrdaveqtlag
lftvmsidavetfhigssnglvyteRvDvlralptgksynlsilgvfqltegkitgwrDyfdlrefeeavdlp
lrg
>1QPR_C
glsdwelaaaraaiargldedlrygpdvttlatvpasattaslvtreagvvagldvalltlnevlgtngyrv
ldrvedgarvppgealmtleaqtrglltaeRtmlnlvghlsgiatataawvdavrgtkakirdtrKtlpglra
lqkyavrtgggvnhrlglgdaalikdnhvaaagsvvdalravrnaapdlpcevEvdsleqldavlpekpelil
lDnfavwqtqtavqrrdsraptvmlessgglslqtaatyaetgvdylavgalthsvrvldigldm
>1VZZ_A
nlptaqevqglmarYielvdvgdieaivqmfaddatveDpfgqppihgreqiaafyrqglgkvracltgpvra
shngcgampfrvemvpcaldvilvmrfdehgriqtmqaywsevnlsv
>1EXN_B
skswgkfieeeeaemasrrnlmivdgtnlgfrfkhnnskkpfassyvstiqslaksysarttivlgdkgksvf
rlehlpeyKgnrdekyaqrteeekaldeqffeylkdafelckttfptftirgveaddmaayivklighlydhv
wlistdgdwdtlltdkvsrfsfttrreyhlrdmyehhnvddveqfislkaimgdlgdnirgvegigakrgyni
irefgnvldiidqlplpgkqkyiqnlnaseellfrnlilvdlptycvdaiaavgqdvldkftkdileiaeq
```

>1UAS_A
fenglgrtpqmgwnswnhfycgineqiiretadalvntglaklgyqyvniddcwaeysrdsqgnfvpnrqtfp
sgikaladyvhakglklgiysdagsqtcsnkmpgsldheeqdvktfaswgvdylkyDncndagrsvmerytrm
snamktygkniffslcewgkenpatwagrmgnswrttgDiadnwgsmtsradendqwaayagpggwndpdmle
vgnggmseaeyrshfsiwalakaplligcdvrsmsqqtknilsnseviavnqdslgvqgkkvqsdnglevwag
plsnnrkavvlwnrqsyqatitahwsniglagsvavtardlwahssfaaqgqisasvaphdckmyvltpn
>1JRP_D
svgkplphdsarahvtgqarylddlpcpantlhlafglsteasaaitgldlepvrespgviavftaadlphdn
daspapspepvlatgevhfvgqpiflvaatshraariaarkarityaprpailtldqalaadsrfeggpviwa
rgdvetalagaahlaegcfeiggqehfylegqaalalpaeggvvihcssqhpseiqhkvahalglafhdvrve
mrrmgggfggkesqgnhlaiacavaaratgrpckmrydrdddmvitgkrhdfriryrigadasgkllgadfvh
larcgwsadlslpvcdRamlhadgsyfvpalrieshrlrtntqsntafrgfggpqgalgmeraiehlargmgr
dpaelralnfydppekktqtthygqevadcvlgelvtrlqksanfttrraeiaawnstnrtlargialspvkf
gisftlthlnqagalvqiytdgsvalnhggtemgqglhakmvqvaaavlgidpvqvritatdtskvpntsata
assgadmngmavkdacetlrgrlagfvaaregcaardvifdagqvqasgkswrfaeivaaaymarislsatgf
yatpklswdrlrgqgrpflyfaygaaitevvidrltgenrilrtdilhdagaslnpaldigqiegayvqgagw
ltteelvwdhcgrlmthapstykipafsdrprifnvalwdqpnreetifrskavgEppfllgisaflalhdac
aacgphwpdlqapatpeavlaavrraegra
>1ECX_A
mrvyfdnnattrvddrvleemivfyrekygnpnsahgmgieanlhmekarekvakvlgvspseifftscates
inwilktvaetfekrkrtiittpieHkavletmkylsmkgfkvkyvpvdsrgvvkleeleklvdedtflvsim
aannevgtiqpvedvtrivkkknketlvhvDavqtigkipfsleklevdyasfsahKfhgpkgvgityirkgv
pirplihgggqerglrsgtqnvpgivgaarameiaveelseaakhmeklrsklvsglmnlgahiitpleislp
ntlsvsfpnirgstlqnllsgygiyvstssactskderlrhvldamgvdrriaqgairislckynteeevdyf
lkkieeilsfldltgnnrr
>1HQC_A
medlalrpktldeyigqerlkqklrvyleaakarkeplehlllfgppglgkttlahviahelgvnlrvtsgpa
iekpgdlaailansleegdilfideihrlsrqaeehlypamedfvmdivigqgpaartirlelprftligatT
rpglitapllsrfgivehleyytpeelaqgvmrdarllgvriteeaaleigrrsrgtmRvakrlfrrvrdfaq
vageevitreralealaalgldelglekrdreilevlilrfgggpvglatlatalsedpgtleevhepylirq
gllkrtprgrvptelayrhlgypppvgpllep
>2Q6N_G
gklppgpsplpvlgnllqmdrkgllrsflrlrekygdvftvylgsrpvvvlcgtdairealvdqaeafsgrgk
iavvdpifqgygvifangerwralrrfslatmrdfgmgkrsveeriqeearclveelrkskgalldntllfhs
itsniicsivfgkrfdykdpvflrlldlffqsfslissfssqvfelfsgflkyfpgthrqiyrnlqeintfig
qsvekhratldpsnprdfidvyllrmekdksdpssefhhqnliltvlslffagteTTsttlrygfllmlkyph
vtervqkeieqvigshrppalddrakmpytdaviheiqrlgdlIpfgvphtvtkdtqfrgyvipkntevfpvl
ssalhdpryfetpntfnpghfldangalkrnegfmpFslgkriClgegiartElflffttilqnfsiaspvpp
edidltprEsgvgnvppsyqirflarh
>1X7D_A
mtyfidvptmsdlvhdigvapfigelaaalrddfkrwqafdksarvashsevgviElmpvadksryafkyvng
hpantarnlhtvmafgvladvdsgypvllseltiatalrtaatslmaaqalarpnarkmalignqaqsefqal
afhkhlgieeivaydtdplataklianlkeysgltirrassvaeavkgvdiittvtadkayatiitpdmlepg
mhlnavggDcpgktelhadvlrnarvfveyepqtriegeiqqlpadfpvvdlwrvlrgetegrqsdsqvtvfd
svgfaledytvlryvlqqaekrgmgtkidlvpwveddpkdlfshtrgragkrrirrva
>1TKK_A
mkiirietsriavpltkpfktalrtvytaesvivritydsgavgwgeapptlvitgdsmdsiesaihhvlkpa
llgkslagyeailhdiqhlltgnmsakaavemalydgwaqmcglplyqmlggyrdtletdytvsvnspeemaa
daenylkqgfqtlKiKvgkddiatdiariqeirkrvgsavklrldanqgwrpkeavtairkmedaglgielve
qpvhkddlaglkkvtdatdtpimadesvftprqafevlqtrsadliniKlmkaggisgaekinamaeacgvec
mvGsmietklgitaaahfaaskrnITrfDfdaplmlktdvfnggitysgstismpgkpglgiigaal
>1FA0_A
mssqkvfgitgpvstvgataaenklndsliqelkkegsfeteqetanrvqvlkilqelaqrfvyevskkknms
dgmardaggkiftygsyrlgvhgpgsdidtlvvvpkhvtredfftvfdsllrerkeldeiapvpdafvpiiki

kfsgisidlicarldqpqvplsltlsdknllrnldekdlralngtrvtdeilelvpkpnvfrialraiKlwaq
rravyanifgfpggvawamlvaricqlypnacsavilnrffiilsewnwpqpvilkpiedgplqvrvwnpkiy
aqdrshrmpvitpaypsmcathnitestkkvilqefvrgvqitndifsnkkswanlfekndfffrykfyleit
aytrgsdeqhlkwsglveskvrllvmklevlagikiahpftkpfessyccpteddyemiqdkygshktetaln
alklvtdenkeeesikdapkaylstmyigldfnienkkekvdihipctefvnlcrsfnedygdhkvfnlalrf
vkgydlpdevfdenekrpskkskrkn
>1D8H_A
hmyrnvpiwaqkwkptikalqsinvkdlkidpsflniipdddltksvqdwvyatiysiapelrsfielemkfg
viidakgpdrvnppvssqcvfteldahltpnidaslfkelskyirgisevtentgkfsiiesqtrdsvyrvgl
stqrprflRmstdiktgrvgqfiekrhvaqlllyspkdsydvkislnlElpvpdndppekyksqspisertKd
Rvsyihndsctriditkvenhnqnsksrqsettheveleintpallnafdnitndskeyaslirtflnngtii
rrklsslsyeifegskkvm
>1D4C_A
aapevladfhgemggcdschvsdkggvtndnlthengqcvschgdlkelaaaapkdkvsphkshligeiacts
chkgheksvaycdachsfgfdmpfggkwerkfvpvdadkaaqdkaiaagvkettdvviigsggaglaaavsar
dagakvillekepipggntklaaggmnaaetkpqaklgiedkkqimiddtmkggrnindpelvkvlannssds
idwltsmgadmtdvgrmggasvnrshrptggagvgahvaqvlwdnavkrgtdirlnsrvvriledasgkvtgv
lvkgeytgyyvikadavviaaggfaknnervskydpklkgfkatnhpgatgdgldvalqagaatrdleyiqaH
ptyspaggvmiteavrgngaivvnregnrfmneittRdkasaailqqkgesaylvfddsirkslkaiegyvhl
nivkegktieelakqidvpaaelaktvtayngfvksgkdaqferpdlprelvvapfyaleiapavHhtmgglv
idtkaevksektgkpitglyaagevtggvhganRlggnaisdivtygriagasaakfakdn
>1KCZ_A
mkivdvlctpgltgfyfddqraikkgaghdgftytgstvtegftqvrqkgesisvllvledgqvahgdcaavq
ysgaggrdplflakdfipviekeiapkligreitnfkpmaeefdkmtvngnrlhtairygitqaildavaktr
kvtmaevirdeynpgaeinavpvfaqsgddrydnvdkmiikeadvlpHalinnveeklglkgeklleyvkwlr
driiklrvredyapifhidvygtigaafdvdikamadyiqtlaeaakpfhlriegpmdvedrqkqmeamrdlr
aeldgrgvdaelvadewcntvedvkfftdnkaghmvqiKtpdlggvnniadaimyckangmgaycggtcnetn
rsaevttnigmacgarqvlakpgmgvdegmmivknemnrvlalvgrrk
>3S4J_A
dvyaqekqdfvqhfsqivrvltedehpeigdaiarlkevleynaiggkynrgltvvvafrelveprkqdadsl
qrawtvgwcvellqafflvaddimdssltRrgqicwyqkpgvgldaindanlleaciyrllklycreqpyyln
lielflqssyqteigqtldlltapqgnvdlvrftekryksivkyktafysfylpiaaamymagidgekehana
kkillemgefFqiqddyldlfgdpsvtgkigtdiqdnkcswlvvqclqratpeqyqilkenygqkeaekvarv
kalyeeldlpavflqyeedsyshimalieqyaaplppavflglarkiy
>1BJP_A
PiaqihilegrsdeqketlirevseaisrsldapltsvRviitemakghFgiggelaskvrr
>1YEY_B
rtiialethdvrfptsreldgsdanpdpdysaayvvlrtdgaedlagyglvftigrgndvqtaavaalaehvv
glsvdkviadlgafarrltndsqlrwlgpekgvhaigavinaawdlaaraankplwrfiaeltpeqlvdtidf
rylsdaltrdealailrdaqpqraartatlieqgypayttspgwlgysdeklvrlakeavadgfrtiKlKvga
nvqddirrcrlaraaigpdiaavdanqrwdvgpaidwrqlaefdiawieeptspddvlghaairqgitpvpvs
tgehtqnrvvfkqllqagavdliqiDaarvggvnenlailllaakfgvrvfpHaggvglcelvqhlaadfvai
tgkedRaiEfvdhlhqhfldpvriqhgrylapevpgfsaehpasiaefsypdgrfwve
>2GLX_D
nrwgligastiarewvigairatggevvsmmstsaergaayatengigksvtsveelvgdpdvdavyvsttne
lhreqtlaairagkhvlceKplamtledaremvvaareagvvlgtnhhlrnaaahramrdaiaegrigrpiaa
rvfhavylpphlqgwrlerpeagggvilditvHdadtlrfvlnddpaeavaishsagmgkegvedgvmgvlrf
qsgviaqfhdafttkfaetgfevhgtegsligrnvmtqkpvgtvtlrnaegesqlpldpanlyetalaafhsa
ieghgqpsatgedgvwslatglavvkaaatgqaaeietgl
>1ECM_A
mtsenpllalRekisaldekllallaeRrelavevgkakllshrpvrdidrerdllerlitlgkahhldahyi
trlfqliiedsvltqqallqqhlnkinphsariafl
>1QV0_A

```
msskyavklktdfdnprwikrHkhmfdfldingngkitldeivskasddicakleatpeqtkrhqvcveaffr
gcgmeygkeiafpqfldgWkqlatselkkwarneptlirewgdavfdifdkdgsgtitldewkaYgkisgisp
sqedceatfrhcdldnagdldvdemtrqHlgfwytldpeadglYgngvp
>1JM6_B
mrwfrallknaslagapkyiehfskfspsplsmkqfldfgssnacektsftflrqelpvrlanimkeinllpd
rvlstpsvqlvqswyvqslldimefldkdpedhrtlsqftdalvtirnrhndvvptmaqgvleykdtygddpv
snqniqyfldrfylsrisirmlinqhtlifdgstnpahpkhigsidpncsvsdvvkdaydmakllcdkyymas
pdleiqevnatnatqpihmvyvpshlyHmlfElfknamratveshessltlppikimvalgeedlsikmsdrg
ggvplrkierlfsymystaptpqpgtggtplagfgyglpisrlyakyfqgdlqlfsmegfgtdaviylkalst
dsverlpvynksawrhyqtiqeagdwcvpstepkntstyrvs
>1A16_A
seisrqefqrrrqalveqmqpgsaalifaapevtrsadseypyrqnsdfwyftgfnepeavlvliksddthnh
svlfnrvrdltaeiwfgrrlgqdaapeklgvdralafseinqqlyqllngldvvyhaqgeyayadvivnsale
klrkgsrqnltapatmidwrpvvhemrlfkspeeiavlrrageitamahtramekcrpgmfeyhlegeihhef
nrhgarypsyntivgsgengcilhytenecemrdgdlvlidagceykgyagditrtfpvngkftqaqreiydi
vlesletslrlyrpgtsilevtgevvrimvsglvklgilkgdvdeliaqnahrpffmhglshwlgldvHdvgv
ygqdrsrilepgmvltvEpglyiapdaevpeqyrgigirieddivitetgnenltasvvkkpeeiealmvaar
kq
>2ENG_A
adgrstrywDcckpscgwakkapvnqpvfscnanfqritdfdaksgcepggvayscadqtpwavnddfalgfa
atsiagsneagwccacyeltftsgpvagkkmvvqststggdlgsnhfDlnipgggvgifdgctpqfgglpgqr
yggissrnecdrfpdalkpgcywrfdwfknadnpsfsfrqvqcpaelvartgcrrnddgnfpav
>1TI6_A
mgevvrltnsstggpvfvyvkdgkiirmtpmdfddavdapswkieargktftpprktsiapytagfksmiysd
lripypmkrksfdpngernpqlrgaglskqdpwsdyeriswdeatdivvaeinrikhaygpsailstpssHhm
wgnvgyrhstyfrfmnmmgftyadhnpDswegwhwggmhmwgfswrlgnpeqydlledglkhaemivfwssdp
etnsgiyagfesnirrqwlkdlgvdfvfidphmnhtarlvadkwfspkigtdhalsfaiaytwlkedsydkey
vaanahgfeewadyvlgktdgtpktcewaeeesgvpaceiralarqwakkntylaagglggwggacrashgie
wargmialatmqgmgkpgsnmwsttqgvpldyefyfpgYaeggisgdcensaagfkfawrmfdgkttfpspsn
lntsagqhiprlkipecimggkfqwsgkgfaggdishqlhqyeypapgyskikmfwkyggphlgtmtatnrya
kmythdslefvvsqsiwfegevpfadiilpactnferwdisefancsgyipdnyqlcnhrvislqakciepvg
esmsdyeiyrlfakklnieemfsegkdelawceqyfnatdmpkymtwdeffkkgyfvvpdnpnrkktvalrwf
aegrekdtpdwgprlnnqvcrkglqtttgkvefiatslknfeeqgyidehrpsmhtyvpawesqkhsplavky
plgmlsphprfsmhtmgdgknsymnyikdhrvevdgykywimrvnsidaeargikngdlirayndrgsvilaa
qvteclqpgtvhsyescavydplgtagksadrggciniltpdryiskyacgmanntalveiekwdgdkyeiy
>1IDJ_A
vgvsgsaegfaegvtgggdatpvypdtidelvsylgddearvivltktfdftdsegtttgtgcapwgtasacq
vaidqddwcenyepdapsvsveyynagvlgitvtsnksligegssgaikgkglrivsgaeniiiqniavtdin
pkyvwggdaitlddcdlvwidhvttarigRqhyvlgtsadnrvsltnnyidgvsdysatcdgyhywgiyldgd
adlvtmkgnyiyhtsgRspkvqdntllhcvnnyfydisghafeigeggyvlaegnvfqnvdtvletyegaaft
vpsttagevcstylgrdcvingfgcsgtfsedstsflsdfegkniasasaytsvasrvvanagqgnl
>2AG0_A
mamitggelvvrtlikagvehlfglhgaHidtifqacldhdvpiidtrhEaaaghaaegyaragaklgvalvt
agggftnavtpianawldrtpvlfltgsgalrddetntlQagidqvamaapitkwahrvmatehiprlvmqai
raalsaprgpvlldlpwdilmnqidedsviipdlvlsahgarpdpadldqalallrkaerpvivlgseasrta
rktalsafvaatgvpvfadyeglsmlsglpdamrgglvqnlysfakadaapdlvlmlgarfglntghgsgqli
phsaqviqvdpdacelgrlqgialgivadvggtiealaqataqdaawpdrgdwcakvtdlaqeryasiaakss
sehalhpfhasqviakhvdagvtvvadgaltylwlsevmsrvkpggflchgylGsmgvgfgtalgaqvadlea
grrtilvtgdgsvgysigefdtlvrkqlpliviimnnqswgatlhfqqlavgpnrvtgtrlengsyhgvaaaf
gadgyhvdsvesfsaalaqalahnrpacinvavaldpippeeliligmdpfa
>1E1A_A
meipvieplftkvtedipgaegpvfdkngdfyivapEvevngkpageilridlktgkktvickpevngyggip
agcqcdrdanqlfvadmrlgllvvqtdgtfeeiakkdsegrrmqgcndcafdyegnlwitapagevapadytr
smqekfgsiycfttdgqmiqvdtafqfpngiavrhmndgrpyqlivaetptkklwsydikgpakienkkvwgh
```

ipgtheggadgmdfdednnllvanwgsshievfgpdggqpkmrircpfekpsnlhfkpqtktifvteHennav
wkfewqrngkkqycetlkfgif
>2ACE_A
ddhsellvntksgkvmgtrvpvlsshisaflgipfaeppvgnmrfrrpepkkpwsgvwnastypnncqqyvde
qfpgfsgsemwnpnremsedclylniwvpsprpksttvmvwiygggfysgsstldvyngkylayteevvlvsl
syrvgafgflalhgsqeapgnvglldqrmalqwvhdniqffggdpktvtifgeSaggasvgmhilspgsrdlf
rrailqsgspncpwasvsvaegrrravelgrnlncnlnsdeelihclrekkpqelidvewnvlpfdsifrfsf
vpvidgeffptslesmlnsgnfkktqillgvnkdEgsffllygapgfskdseskisredfmsgvklsvphand
lgldavtlqytdwmddnngiknrdglddivgdhnvicplmhfvnkytkfgngtylyffnhrasnlvwpewmgv
iHgyeiefvfglplvkelnytaeeealsrrimhywatfaktgnpnephsqeskwplfttkeqkfidlntepmk
vhqrlrvqmcvfwnqflpkllnatac
>1B57_A
skifdfvkpgvitgddvqkvfqvakennfalpavncvgtdsinavletaakvkapvivqfsnggasfiagkgv
ksdvpqgaailgaisgahhvhqmaehygvpvilhtDhcakkllpwidglldagekhfaatgkplfsshmidls
eeslqenieicskylermskigmtleielgctggeEdgvdnshmdasalytqpedvdyaytelskisprftia
asfgnvhgvykpgnvvltptilrdsqeyvskkhnlphnslnfvfhggsgstaqeikdsvsygvvkmNidtdtq
watwegvlnyykaneaylqgqlgnpkgedqpnkkyydprvwlragqtsmiarlekafqelnaidvl
>3DDB_A
mpfvnkqfnykdpvngvdiayikipnagqmqpvkafkihnkiwviperdtftnpeegdlnpppeakqvpvsyy
dstylstdnekdnylkgvtklferiystdlgrmlltsivrgipfwggstidtelkvidtncinviqpdgsyrs
eelnlviigpsadiiqfecksfghevlnltrngygstqyirfspdftfgfeeslevdtnpllgagkfatdpav
tlahelihaghrlygiainpnrvfkvntnayyemsglevsfeElrtfgghdakfidslqenefrlyyynkfkd
iastlnkaksivgttaslqymknvfkekyllsedtsgkfsvdklkfdklykmlteiytednfvkffkvlnRkt
Ylnfdkavfkinivpkvnytiydgfnlrntnlaanfngqnteinnmnftklknftglf
>1QHG_A
mnflseqllahlnkeqqeavrttegpllimagagsgKtrvlthriaylmaekhvapwnilaitftnkaaremr
ervqsllggaaedvwistfhsmcvrilrrdidriginrnfsildptdqlsvmktilkeknidpkkfeprtilg
tisaaknellppeqfakrastyyekvvsdvyqeyqqrllrnhsldfddlimttiqlfdrvpdvlhyyqykfqy
ihidEyqdtnraqytlvkklaerfqnicavgdadqsiyrwrgadiqnilsferdypnakvilleqnyrstkri
lqaaneviehnvnrkpkriwtenpegkpilyyeamneadeaqfvagrireavergerryrdfavlyrtnaqsr
vmeemllkanipyqivgglkfydrkeikdilaylrvianpdddlsllriinvpkrgigastidklvryaadhe
lslfealgelemiglgakaagalaafrsqleqwtqlqeyvsvtelveevldksgyremlkaertieaqsrlen
ldeflsvtkhfenvsddksliafltdlalisdldeldgteqaaegdavmlmtlhaakglefpvvfligmeegi
fphnrsledddemeeerrlayvgitRaeeelvltsaqmrtlfgniqmdppsrflneipahlletasrrqagas
rpavsrpqasgavgswkvgdranhrkwgigtvvsvrgggddqeldiafpspigikrllakfapiekv
>1MHL_A
vtcpeqdkyrtitgmcnnrrsptlgasnrafvrwlpaeyedgfslpygwtpgvkrngfpvalaravsneivrf
ptdqltpdqerslmfmqwgQlldHdldftpepaar
>1O4S_A
vsrriseipisktmeldakakalikkgedvinltagepdfptpepvveeavrflqkgevkytdprgiyelreg
iakrigerykkdispdqvvvtngakqalfnafmalldpgdevivfspvwvsyipqiilaggtvnvvetfmskn
fqpsleevegllvgktkavlinspnnptgvvyrrefleglvrlakkrnfyiisDevydslvytdeftsildvs
egfdrivyingfsKshsmtgwrvgylissekvatavskiqshttscintvaqyaalkalevdnsymvqtfker
knfvverlkkmgvkfvepegafylffkvrgddvkfcerlleekkvalvpgsaflkpgfvrlsfatsierltea
ldriedflns
>1KYW_A
mgstgetqitpthisdeeanlfamqlasasvlpmilksaleldlleiiakagpgaqispieiasqlpttnpda
pvmldrmlrllacyiiltcsvrtqqdgkvqrlyglatvakylvknedgvsisalnlmnqdkvlmeswyhlkda
vldggipfnkaygmtafeyhgtdprfnkvfnkgmsdhstitmkkiletytgfeglkslvdvgggtgavintiv
skyptikginfdlphviedapsypgvehvggdmfvsipkadavfmkwicHdwsdehclkflkncyealpdngk
vivaecilpvapdsslatkgvvhidvimlahnpggkertqkefedlakgagfqgfkvhcnafntyimeflkkv
>2X7J_A
tvnpithyigsfidefalsgitdavvcpgSrStplavlcaahpdisvhvqidErsagffalglakakqrpvll
icTsgtaaanfypavveahysrvpiivltadrphelrevgapqainqhflfgnfvkfftdsalpeespqmlry

irtlasraageaqkrpmgpvhvnvplreplmpdlsdepfgrmrtgrhvsvktgtqsvdreslsdvaemlaeae
kgmivcgelhsdadkeniialskalqypiladplsnlrngvhdkstvidaydsflkddelkrklrpdvvirfg
pmpvskpvflwlkddptiqqividedggwrdptqasahmihcnasvfaeeimagltaatrssewlekwqfvng
rfrehlqtissedvsfegnlyrilqhlvpensslfvgnsmpirdvdtffekqdrpfriysnrGaNgidgvvss
amgvcegtkapvtlvigdlsfyhdlngllaakklgipltvilvnndgggifsflpqasekthfedlfgtptgl
dfkhaaalyggtyscpaswdefktayapqadkpglhlieiktdrqsrvqlhrdmlneavrevkkqwel
>1MPX_A
tsptpditgkpfvaadasndyikreviprdgvklhtvivlpkgaknapivltrtpYdasgrterlasphkdll
sagddvfveggyirvfqdvrgkygsegdyvtrplrgplnpsevdhatdawdtidwlvknvsesngkvgigsSY
egftvvaltnphpalkvavpespidgwgddwfnygafrqvnfdyftgqlskrgkgagiarqghddysnflqag
sagdfakaagleqlpwwhkltehaaydafwqeqaldkvartplkvptwlqglwDqedwgaihsyaaeprdkrn
tlnylvgpwrHsqvnydgsalgalnfegdtarqfrhdvlrpffdqylvdgapkadtppvfiyntgenhwdrlk
awprscdkgcaatskplylqaggklsfqppvagqagfeeyvsdpakpvpfvprpvdfadrawttwlvhdqrfv
dgrpdvltfvteplteplqiagapdvhlqastsgsdsdwvvklidvypeeasnpkggyelpvslaifrgryre
sfstpkpltsnqplafqfglptanhtfqpghrvvqvqsslfplydrnpqtyvpniffakpgdyqkatqrvyvs
peqpsyislpvr
>1NWS_D
yklvcyytswsqyregdgscfpdaldrflcthiiysfanisndhidtwewndvtlygmlntlknrnpnlktll
svggwnfgsqrfskiasntqsrrtfiksvppflrthgfdglDlawLypgrrdkqhfttlikemkaefikeaqp
gkkqlllsaalsagkvtidssydiakisqhldfisimtydfhgawrgttghhsplfrgqedaspdrfsntdya
vgymlrlgapasklvmgiptfgrsftlassetgvgapisgpgipgrftkeagtlayyeicdflrgatvhrilg
qqvpyatkgnqwvgyddqesvkskvqylkdrqlagamvwaldlddfqgsfcgqdlrfpltnaikdalaat
>1LBA_A
akqrestdaifvhcsatkpsqnvgvreirqwhkeqgwldvgYhfiikrdgtveagrdemavgshakgynhnsi
gvclvggiddkgkfdanftpaqmqslrsllvtllakyegavlrahhevapKacpsfdlkrwweknelvtsdrg
>1KYQ_A
mvkslqlahqlkdkrilligggevgltrlyklmptgckltlvspdlhksiipkfgkfiqnkdqpdyredakrf
inpnwdptkneiyeyirsdfkdeyldlenendawyiimtcipdhpesariyhlckerfgkqqlvnvaDkpdlc
dfyfganleigdrlqilistnglsprfgalvrdeirnlftqmgdlaledavvklgelrrgirllapddkdvky
rmdwarrctdlfgiqhchnidvkrlldlfkvmfqeqncslqfpprerllseycss
>1MYR_A
deeitcqennpftcgntdglnsssfeadfifgvassayqiegtigrglniwdgfthrypdksgpdhgngdttc
dsfsywqkdidvldelnatgyRfsiawsriiprgkrsrgvnqkgidyyhglidglikkgitpfvtlfhwdlpq
tlqdeyegfldpqiiddfkdyadlcfeefgdsvkywltinQlySvptrgygsaldapgrcsptvdpscyagns
stepyivahhqllahakvvdlyrknythqggkigptmitrwflpyndtdrhsiaatermkqfflgwfmgpltn
gtypqimidtvgarlptfspeetnlvkgsydflglNyYftqyaqpspnpvnatnhtammdagakltyinasgh
yigplfesdggdgssniyyypkgiysvmdyfknkyynpliyvtEngistpgsenrkesmldytridylcshlc
flnkvikekdvnvkgylawalgdnyefnngftvrfglsyinwnnvtdrdlkksgqwyqkfisp
>1B85_A
iekratcsngktvgdasccawfdvlddiqqnlfhggqcgaeahesiRlvfHdsiaispameaqgkfggggadg
simifddietafhpNigldeivklqkpfvqkhgvtpgdfiafagavalsncpgapqmnfftgrapatqpapdg
lvpepfhtvdqiinrvndagefdelelvfmlsahsvaavndvdptvqglpFdstpgifdsqffvetqlrgtaf
pgsggnqgevesplpgeiriqsdhtiardsrtacewqsfvnnqsklvddfqfiflaltqlgqdpnamtdcsdv
ipqskpipgnlpfsffpagktikdveqacaetpfptlttlpgpetsvqrippppga
>9ICY_A
etlnggitdmltelanfeknvsqaihkynayrkaasviakyphkiksgaeakklpgvgtkiaekideflatgk
lrklekirqddtsssinfltrvsgigpsaarkfvdegiktledlrknedklnhhqriglkyfgdfekripree
mlqmqdivlnevkkvdseyiatvcgsfrrgaessgdmdvllthpsftsestkqpkllhqvveqlqkvhfitdt
lskgetkfmgvcqlpskndekeyphrriDirlipkdqyycgvlyftgsdifnknmrahalekgftineytirp
lgvtgvageplpvdsekdifdyiqwkyrepkdrse
>2ABK_A
mnkakrleiltrlrennphpttelnfsspfelliavllsaqatdvsvnkataklypvantpaamlelgvegvk
tyiktiglynskaeniiktcrilleqhngevpedraalealpgvgrKtanvvlntafgwptiavDthifrvcn
rtqfapgknveqveekllkvvpaefkvdchhwlilhgrytciarkprcgsciiedlceykekvdi

```
>1WGI_A
tyttrqigakntleykvyiekdgkpvsafhdiplyadkennifnmvveiprwtnakleitkeetlnpiiqdtk
kgklrfvrncfphhgyihnygafpqtwedpnvshpetkavgdnDpidvleigetiaytgqvkqvkalgimall
degetdwkviaidindplapklndiedvekyfpgllratnewfriykipdgkpenqfafsgeaknkkyaldii
kethdswkqliagkssdskgidltnvtlpdtptyskaasdaippaslkadapidksidkwffisgsv
>2X0B_A
ssviltnymdtqyygeigigtppqtfkvvfDTgSsnvwvpsskcsrlytacvyhklfdasdsssykhngtelt
lrystgtvsgflsqdiitvggitvtqmfgevtempalpfmlaefdgvvgmgfieqaigrvtpifdniisqgvl
kedvfsfyynrdselggqivlggsdpqhyegnfhyinliktgvwqiqmkgvsvgsstllcedgclalvDtgAs
yisgstssieklmealgakkrlfdyvvkcnegptlpdisfhlggkeytltsadyvfqesysskklctlaiham
dippptgptwalgatfirkfytefdrrnnrigfalar
>5FIT_A
msfrfgqhlikpsvvflktelsfalvnrkpvvpghvlvcplrpverfhdlrpdevadlfqttqrvgtvvekhf
hgtsltfsmQdgpeagqtvkHvHvhvlprkagdfhrndsiyeelqkhdkedfpaswrseeemaaeaaalrvyf
q
>1BH2_A
revkllllgagEsgkstivkqmkiiheagyseeeckqykavvysntiqsiiaiiramgrlkidfgdaaradda
rqlfvlagaaeegfmtaelagvikrlwkdsgvqacfnrsreyqlndsaayylndldriaqpnyiptqqdvlrt
RvkTtgivethftfkdlhfkmfdvggQrserkkwihcfegvtaiifcvalsdydlvlaedeemnrmhesmklf
dsicnnkwftdtsiilflnkkdlfeekikkspltìcypeyagsntyeeaaayiqcqfedlnkrkdtkeiythf
tcstdtknvqfvfdavtdviikn
>1XRH_D
sskisretlhqlienklcqaglkrehaatvaevlvyadargihsHgavrveyyaeriskggtnrepefrleet
gpcsailhadnaagqvaakgehaiktaqqngvavvgisrghsgaisyfvqqaaragfigiscqsdpvvpfgga
eiyygtnplafaapgegdeiltfdattvqawgkvldarsrnsipdtwavdkngvpttdpfavhallpaagpkg
yglidvlsgvllglpfgrqvssyddlhagrnlgqlhivinpnffssselfrqhlsqtrelnaitpapgfnqvy
ypgqdqdikqrkaavegieivddiyqylisdalyntsy
>3BG9_D
qnraqkllhylghvmvngpttpipvkaspsptdpvvpavpigpppagfrdillregpegfaravrnhpglllm
dttfrDahqsllatrvrthdlkkiapyvahnfsklfsmenwggatfdvamrflyecpwrrlqelrelipnipf
qmllrganavgytnypdnvvfkfcevakengmdvfrvfDslnylpnmllgmeaagsaggvveaaisytgdvad
psrtkyslqyymglaeelvragthilciKdmagllkptactmlvsslrdrfpdlplhihthdtsgagvaamla
caqagadvvdvaadsmsgmtsqpsmgalvactrgtpldtevpmervfdyseywegarglyaafdctatmksgn
sdvyeneipggqytnlhfqahsmglgskfkevkkayveanqmlgdlikvtpsskivgdlaqfmvqnglsraea
eaqaeelsfprsvveflqgyigvphggfpepfrskvlkdlprvegrpgaslppldlqalekelvdrhgeevtp
edvlsaamypdvfahfkdftatfgpldslntrlflqgpkiaeefevelergktlhikalavsdlnragqrqva
felngqlrsilvkdtqa
>1DEW_A
gpalyedppdhktspsgkpatlkicswNvdglrawikkkgldwvkeeapdilclqetkcsenklpaelqelpg
lshqywsapsdkegysgvgllsrqcplkvsygigdeehdqegrvivaefdsfvlvtaYvpnagrglvrleyrq
rwdeafrkflkglasrkplvlcgDlNvaheeidlrnpkgnkknagftpqerqgfgellqavpladsfrhlypn
tpyaytfwtymmnarsknvgwrlDyfllshsllpalcdskirskalgsdHcpitlylal
>1HLG_A
spevtmnisqmitywgypneeyevvtedgyilevnripygkknsgntgqrpvvflqhgllasatnwisnlpnn
slafiladagydvwlgnsrgntwarrnlyyspdsvefwafsfdemakydlpatidfivkktgqkqlhyvghSq
gttigfiafstnpslakriktfyalapvatvkytkslinklrfvpqslfkfifgdkifyphnffdqflatevc
sremlnllcsnalfiicgfdsknfntsrldvylshnpagtsvqnmfhwtqavksgkfqaydwgspvqnrmhyd
qsqppyynvtamnvpiavwnggkDlladpqdvglllpklpnliyhkeipfynHldfiwamdapqevyndivsm
isedkk
>4E36_B
vatngerfpwqelrlpsvviplhydlfvhpnltsldfvasekievlvsnatqfiilhskdleitnatlqsepg
kelkvlsypaheqiallvpekltphlkyyvamdfqaklgdgfegfykstyrtlggetrilavtdfeptqarma
fpcfdeplfkanfsikirresrhialsnmpkvktielegglledhfettvkmstylvayivcdfhslsgftss
gvkvsiyaspdkrnqthyalqaslklldfyekyfdiyyplskldliaipdfapgamEnwglityretsllfdp
```

```
ktssasdklwvtrviahelahqwfgnlvtmewwndiwlkegfakymeliavnatypelqfddyflnvcfevit
kdslnssrpiskpaetptqiqemfdevsynkgacilnmlkdflgeekfqkgiiqylkkfsyrnaknddlwssl
snsaevkemmttwtlqkgipllvvkqdgcslrlqqerflqgvrylwhipltystsssnvihrhilksktdtld
lpektswvkfnvdsngyyivhyeghgwdqlitqlnqnhtllrpkdrvglihdvfqlvgagrltldkaldmtyy
lqhetsspalleglsylesfyhmmdrrnisdisenlkryllqyfkpvidrqswsdkgsvwdrmlrsallklac
dlnhapciqkaaelfsqwmessgklniptdvlkivysvgaqttagwnylleqyelsmssaeqnkilyalstsk
hqekllklielgmegkviktqnlaallhaiarrpkgqqlawdfvrenwthllkkfdlgsydirmiisgttahf
sskdklqevklffesleaqgshldifqtvletitknikwleknlptlrtwlmvntr
>1DZR_A
mmiviktaipdvlilepkvfgdergfffesynqqtfeeligrkvtfvqdnhskskknvlrglHfqrgenaqgk
lvrcavgevfdvavdirkesptfgqwvgvnlsaenkrqlwipegfahgfvtlseyaeflykatnyyspssegs
ilwndeaigiewpfsqlpelsakDaaaplldqallte
>1UAM_A
mgsshhhhhhssglvprgshmwigvislfpemfkaitefgvtgravkhnllkvecwnprdftfdkhktvddrp
ygggpgmlmmvqplrdaihtakaaagegakviylspqgrkldqggvtelaqnqklilvcgryegiderliqte
ideewsigdyvltggelpamtlidavarfipgvlgkqasaeeDsfadglldcphytrpevlegltvppvlmsg
hheeirkwrlkqslqrtwlrrpelleglaltdeqrkllkeaqaehnslehhhhhh
>1JXH_B
mghhhhhhhhhhssgyhiqgrhmqrinaltiagtdpsggagiqadlktfsalgaygcsvitalvaentcgvqs
vyriepdfvaaqldsvfsdvridttkigmlaetdiveavaerlqrhhvrnvvldtvmlaksgdpllspsaiet
lrvrllpqvslitpnlpeaaalldapharteqemlaqgrallamgceavlmKgghledaqspdwlftregeqr
fsaprvntknthGtgctlsaalaalrprhrswgetvneakawlsaalaqadtlevgkgigpvhhfhaww
>1ZE1_D
mkhgilvaykpkgptshdvvdevrkklktrkvghggtlDpfacgvliigvnqgtrilefykdlkkvYwvkmrl
glitetfditgevveerecnvteeeireaifsfvgeydqvppapkrvkifkiwdvniegrdvsfrvevspgty
irslcmdigyklgcgatavelvresvgphtieeslnvfeaapeeienriiplekclewlprvvvhqestkmil
ngsqihlemlkewdgfkkgevvrvfneegrllalaeaernssfletlrkherqrrvltlrkvfqt
>4JR0_B
nadnsykmdypemglciiinnknfhkstgmTsrsgtdvdaanlretfrnlkyevrnkndltreeivelmrdvs
kedhskrssfvcvllsHGeegiifgtngpvdlkkitnffrgdrcrsltGkpklfiiqaCRgtipveadflyay
stapgyyswrnskdgswfiqslcamlkqyadklefmhiltrvnrkvatefesfsfdatfhakkqipcivsmlt
kelyfyh
>3QN0_C
sttlfkdftfeaahrlphvpeghkCgrlhghsfmvrleitgevdphtgwiidfaelkaafkptyerldhhyln
dipglenptsevlakwiwdqvkpvvpllsavmvkEtctagciyr
>1CC1_S
mtegakkapviwvqgqgcTgcsvsllnavhprikeilldvislefhptvmasegemalahmyeiaekfngnff
llvegaiptakegrycivgetldakahhhevtmmelirdlapkslatvavgtcsayggipaaegnvtgsksvr
dffadekiekllvnvpgcpphpdwmvgtlvaawshvlnptehplpeldddgrpllffgdnihencpyldkydn
sefaetftkpgckaelgckgpstyadcakrrwnnginwcvenavcigcvepdfpdgkspfyvae
>1B73_A
mkigifDSgvggltvlkairnryrkvdivylgdtarvpygirskdtiiryslecagflkdkgvdiivvaCnta
sayalerlkkeinvpvfgviepgvkealkksrnkkigvigtpatvksgayqrkleeggadvfakacplfapla
eegllegeitrkvvehylkefkgkidtlilgCthypllkkeikkflgdaevvdssealslslhnfikddgsss
lelfftdlspnlqfliklilgrdypvklaegvfth
>1IG8_A
mvhlgpkkpqarkgsmadvpkelmqqienfekiftvptetlqavtkhfiselekglskkggnipmipgwvmdf
ptgkesgdflaidlggtnlrvvlvklggdrtfdttqskyrlpdamrttqnpdelwefiadslkafideqfpqg
isepiplgftfSfpasqnkinegilqRwtkgfdipnienhdvvpmlqkqitkrnipievvalinDttgtlvas
yytdpetkmgvifgtgvngayydvcsdieklqgklsddippsapmainceygsfdnehvvlprtkyditidee
sprpgqqtfekmssgyylgeilrlalmdmykqgfifknqdlskfdkpfvmdtsyparieedpfenledtddlf
qnefginttvqerklirrlseligaraarlsvcgiaaicqkrgyktghiaadgsvynrypgfkekaanalkdi
ygwtqtslddypikivpaedgsgagaaviaalaqkriaegksvgiiga
>1AHJ_A
```

```
msvtidhttenaapaqaavsdrawalfraldgkglvpdgyvegwkktfeedfsprrgaelvarawtdpefrql
lltdgtaavaqygylgpqgeyivavedtptlknvivcslCSCtawpilglpptwyksfeyrarvvreprkvls
emgteiasdieirvydttaetrymvlpqrpagtegwsqeqlqeivtkdcligvaipqvptv
>1H3I_B
fffdgstlegyyvddalqgqgvytyedggvlqgtyvdgelngpaqeydtdgrlifkgqykdnirhgvcwiyyp
dggslvgevnedgemtgekiayvypdertalygkfidgemiegklatlmsteegrphfelmpgnsvyhfdkst
sscistnallpdpyeservyvaeslissageglfskvavgpntvmsfyngvrithqevdsrdwalngntlsld
eetvidvpepynhvskycaslgHkanhsftpnciydmfvhprfgpikcirtlraveadeeltvaYgydhsppg
k
>1PTD_A
assvnelenwskwmqpipdsiplarisipgtHdsgtfklqnpikqvwgmtqeydfryqmdhgarifdiRgrlt
ddntivlhHgplylyvtlhefineakqflkdnpsetiimslkkeyedmkgaedsfsstfekkyfvdpiflkte
gniklgdargkivllkrysgsnepggynnfywpdnetftttvnqnanvtvqdkykvsydekvksikdtmdetm
nnsedlnhlyinftslssggtawnspyyyasyinpeianyikqknparvgwviqDyinekwspllyqeviran
kslike
>1O8A_A
deaeaskfveeydrtsqvvwneyaeanwnyntnittetskillqknmqianhtlkygtqarkfdvnqlqntti
kriikkvqdleraalpaqeleeynkilldmettysvatvchpngsclqlepdltnvmatsrkyedllwawegw
rdkagrailqfypkyvelinqaarlngyvdagdswrsmyetpsleqdlerlfqelqplylnlhayvrralhrh
ygaqhinlegpipahllgnmwaqtwsniydlvvpfpsapsmdtteamlkqgwtprrmfkeaddfftslgllpv
ppefwnksmlekptdgrevvcHAsawdfyngkdfrikqcttvnledlvvahhEmghiqyfmqykdlpvalreg
anpgfheaigdvlalsvstpkhlhslnllssdehdinflmkmaldkiafipfsylvdqwrwrvfdgsitkeny
nqewwslrlkyqglcppvprtqgdfdpgakfHipssvpyirYfvsfiiqfqfhealcqaaghtgplhkcdiyq
skeagqrlatamklgfsrpwpeamqlitgqpnmsasamlsyfkplldwlrtenelhgeklgwp
>1RYP_1
qfnpygdngGtilgiagedfavlagdtrnitdysinsryepKvfdcgdnivmsanGfaadgdalvkrfknsvk
wyhfdhndkklsinsaarniqhllygkrffpyyvhtiiagldedgkgavysfdpvgsyereqcraggAaasli
mpfldnqvnfknqyepgtngkvkkplkylsveeviklvrdsftsaterhiqvgdgleilivtkdgvrkefyel
krd
>1QOL_H
meltlyngekktfysrpnnhdnAwlnailqlfryveepffdwvysspenltleaikqledltglelheggppa
lviwnikhllhtgigtasrpsevcvvdgtdmcladfhagiflkgqeHavfacvtsngwyaiDdedfypwtpdp
sdvlvfvpydqeplngewkakvqrklk
>1YCF_B
sqpvaitdgiywvgavdwniryfHgpafsthrgttynaylivddktalvdtvyepfkeeliaklkqikdpvkl
dylvvnhtesdhagafpaimelcpdahvlctqrafdslkahyshidfnytivktgtsvslgkrsltfieapml
hwpdsmftyvpeealllpndafgqhiatsvrfddqvdaglimdeaakYyanilmpfsnlitkkldeiqkinla
iktiapshgiiwrkdpgriieayarwaegqgkakaviaydtmwlstekmahalmdglvaggcevklfklsvsd
rndvikeildaravlvgsptinndilpvvsplldlvglrpknkvglafgaygwgggaqkileerlkaakiel
iaepgptvqwvprgedlqrcyelgrkiaariad
>1A95_A
msekyivtwdmlqiharklasrlmpseqwkgiiavsrgglvpgallarelgirhvdtvcissydhdnqrelkv
lkraegdgegfiviDDlvDtggtavairemypkahfvtifaKpagrplvddyvvdipqdtwieqpwdmgvvfv
ppisgr
>1FHL_A
altyrgadissllllledegysyknlngqtqaletiladaginsiRqrvwvnpsdgsydldynlelakrvkaag
mslyldlhlsdtwadpsdqttpsgwsttdlgtlkwqlynytlevcntfaendidieiisignEiragllwplg
etssysnigallhsgawgvkdsnlattpkimihlddgwswdqqnyfyetvlatgellstdfdyfgvsyypfys
asatlaslktslanlqstydkpvvvvEtnwpvscpnpayafpsdlssipfsvagqqefleklaavveattdgl
gvyywepawignaglgsscadnlmvdyttdevyesietlgel
>1SZD_A
masmsvstastemsvrkiaahmksnpnakvifmvgagistscgiPDFRspgtglyhnlarlklpypeavfdvd
ffqsdplpfytlakelypgnfrpskfhyllklfqdkdvlkrvytqNiDtlerqagvkddliieaHgsfahchc
```

igcgkvyppqvfksklaehpikdfvkcdvcgelvkpaivffgedlpdsfsetwlndsewlrekittqqplviv
vgtslavypfaslpeeiprkvkrvlcnletvgdfkankrptdlivhqysdefaeqlveelgwqedfekilta
>1PYL_B
aladvcrtklpsqaqdtlaliakngpypynrdgvvfenresrlpkkgngyyhEftvvtpgsndRgtrrvvtgg
ygeqywspdHyatfqeidprc
>1MRO_A
adklfinalkkkfeespeekkttfytlggwkqserktefvnagkevaakrgipqynpdigtplgqrvlmpyqv
sttdtyvegddlhfvnnaamqqmwddirrtvivglnhahaviekrlgkevtpetithyletvnhampgaavvq
ehmvethpalvadsyvkvftgndeiadeidpafvidinkqfpedqaetlkaevgdgiwqvvriptivsrtcdg
attsrwsamqigmsmisaykqaageaatgdfayaakaevihmgtylpvrargenepggvpfgyladicqssrv
nyedpvrvsldvvatgamlydqiwlgsymsggvgftqyataaytdnilddftyfgkeyvedkyglceapnnmd
tvldvatevtfygleqyeeypalledqfggsraavvaaaagcstafatgnaqtglsgwylsmylhkeqhsrlg
fyydlqdqgasnvfsirgdeGlplelrgpnypnyamnvghqgeyagisqaphaargDafvfnplvkiafaddn
lvfdftnvrgefakgalrefepageralitpa
>3D47_G
menimtlpkikhvrawfiggataekgagggdyhdqggnhwiddhiatpmskyrdyeqsrqsfginvlgtlive
veaenrqtgfavstAgemgcfivekhlnrfiegkcvsdiklihdqmlgatmyysgsgglvmntiscvdlalwd
lfgkvvglpvykllggavrdeiqfyatgarpdlakemgfiggKmPthwGpHdgdagirkdaamvadmrekcgp
dfwlmldcwmsqdvnyatklahacapfnlkwieeclppqqyegyrelkrnapagmmvtsgehhgtlqsfrtla
etgidimqpDvgwcggltltlveiaalaksrgqlvvpHgsSvyshhavitftntpfsEFlmtspdcstlrpqfd
pilldepvpvngrihksvldkpgfgvelnrdchlkrpysh
>1H16_A
selneklatawegftkgdwqnevnvrdfiqknytpyegdesflagateatttlwdkvmegvklenrthapvdf
dtavastitshdagyinkqlekivglqteaplkralipfggikmiegsckaynreldpmikkifteyrkthnq
gvfdvytpdilrcrksgvltglpdaygrgriigdyrrvalygidylmkdklaqftslqadlengvnleqtirl
reeiaeqhralgqmkemaakygydisgpatnaqeaiqwtyfgylaavksqngaamsfgrtstfldvyierdlk
agkiteqeaqemvdhlvmklrmvrflrtpeydelfsgdpiWatesiggmgldgrtlvtknsfrflntlytmgp
spepnmtilwseklplnfkkfaakvsidtsslqyenddlmrpdfnnddyaiaCCvspmivgkqmqffgaranl
aktmlyainggvdeklkmqvgpksepikgdvlnydevmermdhfmdwlakqyitalniihymhdkysyeaslm
alhdrdvirtmacgiaglsvaadslsaikyakvkpirdedglaidfeiegeypqfgnndprvddlavdlverf
mkkiqklhtyrdaiptqsvltitsnvvygkktgntpdgrragapfgpganpmhgrdqkgavasltsvaklpfa
yakdgisytfsivpnalgkddevrktnlaglmdgyfhheasieggqhlnvnvmnremlldamenpekypqlti
rvsGyavrfnsltkeqqqdvitrtftqsm
>1ALK_A
tpempvlenraaqgnitapggarrltgdqtaalrnslsdkpakniilligdgmgdseitaarnyaegaggffk
gidalpltgqythyalnkktgkpdyvtdSaasatawstgvktyngalgvdihekdhptilemakaaglatgnv
staelqdatpaalvahvtsRkcygpsatsqkcpgnalekggkgsiteqllnaradvtlgggaktfaetatage
wqgktlreeaeargyqlvsdaaslnsvteanqqkpllglfadgnmpvrwlgpkatyhgnidkpavtctpnpqr
ndsvptlaqmtdkaiellsknekgfflqvegasidkqdhaanpcgqigetvdldeavqralefakkegntlvi
vtadhahasqivapdtkapgltqalntkdgavmvmsygnseedsqehtgsqlriaaygphaanvvgltdqtdl
fytmkaalglk
>1P4N_A
pvlnlndpqaveryeefmrqspygqvtqdlgwakvKnnwepvdvyleddqgaiiaamsmllgdtptdkkfaya
skgpvmdvtdvdlldrlvdeavkaldgrayvlrfDpevaysdefnttlqdhgyvtrnrnvadagmhatiqprl
nmvldltkfpdakttldlypsktkskikrpfrdgvevhsgnsateldeffktyttmaerhgithRpieyfqrm
qaafdadtmrifvaeregkllstgialkygrkiwymyagsmdgntyyapyavqsemiqwaldtntdlydlggi
esestddslyvfkhvfvkdapreyigEidkvldpevyaelvkd
>4EBF_B
lpklvithrvheeilqllaphcelitnqtdstltreeilrrcrdaqaafpdrvdadflqacpelrvigcalkg
fdnfdvdactargvwltfvpdlLtvptaelaiglavglgrhlraadafvrsgqfrgwqprfygtgldnatvgf
lggaiglaadrlqgwgatlqyhaakaldtqteqrlglrqvacselfassdfillalplnadtlhlvnaellal
vrpgallvnpcRgsvvdeaavlaalergqlggyaaDvfeEdwarAdRpqqidpallahpntlftpHigsavra
vrleiercaaqnilqalagerpinavnrlpk
>1AZY_A

lflaqeiirkkrdghalsdeeirffingirdntisegqiaalamtiffhdmtmpervsltmamrdsgtvldwk
slhlngpivdkHstggvgdvtslmlgpmvaacggyipmisgrglghtggtldklesipgfdifpddnrfreii
kdvgvaiigqtsslapadkrfyatRditatvdsiplitasilaKklaegldalvmdvkvgsgafmptyelsea
laeaivgvangagvrttalltdmnqvlassagnavevreavqfltgeyrnprlfdvtmalcvemlisgklakd
daearaklqavldngkaaevfgrmvaaqkgptdfvenyakylptamltkavyadtegfvsemdtralgmavva
mgggrrqasdtidysvgftdmarlgdqvdgqrplavihakdennwqeaakavkaaikladkapestptvyrri
se
>1BWZ_A
mqfskmhglgndfvvvdgvtqnvfftpetirrlanrhcgigfdqlliveapydpeldfhyrifnadgsevsqC
gngarcfarfvtlkgltnkkdisvstqkgnmvltvkdmnqirvnmgepiwepakipftankfeknyilrtdiq
tvlcgavsmgnpHcvvqvddiqtanveqlgpllesherfpervnagfmqiinkehiklrvyErgagetqaCgs
gacaavavgimqgllnnnvqvdlpggslmiewngvghplymtgeathiydgfitl
>1B7Y_A
mleealaaiqnardleelkalkarylgkkglltqemkglsalpleerrkrgqelnaikaaleaalearekale
eaalkealerervdvslpgaslfsgglhpitlmerelveifralgyqavegpeveseffnfdalnipehhpar
dmwdtfwltgegfrlegplgeevegrlllrthtspmqvrymvahtppfrivvpgrvfRfeqtdatheavfhql
eglvvgegiamahlkgaiyelaqalfgpdskvrfqpvyfpfvepgaqfavwwpeggkwlelggagmvhpkvfq
avdayrerlglppayrgvtgfAfglgverlamlrygipdiryffggrlkfleqfkgvl
>2XLL_B
vaqispqypmftvplpippvkqprltvtnpvngqeiwyyeveikpfthqvypdlgsadlvgydgmspgptfqv
prgvetvvrfinnaeapnsvhlhgsfsraafdgwaeditepgsfkdyyypnrqsartlwyhdhamhitaenay
rgqaglymltdpaedalnlpsgygefdipmiltskqytangnlvttngelnsfwgdvihvngqpwpfknvepr
kyrfrfldaavsrsfglyfadtdaidtrlpfkviasdsgllehpadtsllyismaeryevvfdfsdyagktie
lrnlggsiggigtdtdydntdkvmrfvvaddttqpdtsvvpanlrdvpfpspttntprqfrfgrtgptwting
vafadvqnrllanvpvgtverwelinagngwthpihihlvdfkvisrtsgnnartvmpyesglkdvvwlgrre
tvvveahyapfpgvymfHChnlihedhdmmaafnatvlpdygynatvfvdpmeelwqarpyelgefqaqsgqf
svqavteriqtmaeyrpyaaad
>1O7A_A
palwplplsvkmtpnllhlapenfyishspnstagpsctlleeafrryhgyifgtqvqqllvsitlqsecdaf
pnissdesytllvkepvavlkanrvwgalrgletfsqlvyqdsygtftinestiidsprfshrgilidtsrhy
lpvkiilktldamafnkfnvlhwhivddqsfpyqsitfpelsnkgsyslshvytpndvrmvieyarlrgirvl
pefdtpghtlswgkgqkdlltpcyskldsfgpinptlnttysflttffkeisevfpdqfihlggDEvefkcwe
snpkiqdfmrqkgfgtdfkklesfyiqkvldiiatinkgsivwqevfddkaklapgtivevwkdsaypeelsr
vtasgfpvilsapwyldlisygqdwrkyykvepldfggtqkqkqlfiggeaclwgeyvdatnltprlwprasa
vgerlwsskdvrdmddaydrltrhrcrmvergiaaqplyagycnh
>2RSP_A
lamtmehkdrplvrviltntgshpvkqrsvyitallDSgaditiiseedwptdwpvmeaanpqihgigggipm
rksrdmielgvinrdgslerplllfpavamvrgsilgrdclqglglrltnl
>1ARZ_A
mhdanirvaiagaggrmgrqliqaalalegvqlgaaleregssllgsdagelagagktgvtvqssldavkddf
dvfidftrpegtlnhlafcrqhgkgmvigttgfdeagkqairdaaadiaivfaanfsvgvnvmlkllekaakv
mgdytdieiieaHhrhKvdapsgtalamgeaiahaldkdlkdcavysreghtgervpgtigfatvragdivge
htamfadigerleithkassrmtfangavrsalwlsgkesglfdmrdvldlnnl
>1DO6_A
misetirsgdwkgEKhvpvieyeregelvkvkvqvgkeiphpnttehhiryielyflpegenfvyqvgrveft
ahgesvngpntsdvytepiayfvlktkkkgklyalsycnihglwenevtle
>1QJE_A
mgsvskanvpkidvsplfgddqaakmrvaqqidaasrdtgffyavnhginvqrlsqktkefhmsitpeekwdl
airaynkehqdqvragyylsipgkkavesfcylnpnftpdhpriqaktpthevnvwpdetkhpgfqdfaeqyy
wdvfglssallkgyalalgkeenffarhfkpddtlasvvlirypyldpypeaaiktaadgtklsFewhedvsl
itvlyqsnvqnlqvetaagyqdieaddtgylincgsymahltnnyykapihrvkwvnaerqslpffvnlgyds
vidpfdprepngksdreplsygdylqnglvslinkngqt
>1DOD_A

```
mktqvaiigagpsglllgqllhkagidnvilerqtpdyvlgriragvleqgmvdllreagvdrrmardglvhe
gveiafagqrrridlkrlsggktvtvygqtevtrdlmeareacgattvyqaaevrlhdlqgerpyvtferdge
rlrldcdyiagcdgfhgisrqsipaerlkvfervypfgwlglladtppvsheliYanhprgfalcsqrsatrs
ryyvqvplsekvedwsderfwtelkarlpsevaeklvtgpsleksiaplrsfvvepmqhgrlflagdaahivp
ptgakglnlaasdvstlyrlllkayregrgellerysaiclrriwkaerfswwmtsvlhrfpdtdafsqriqq
teleyylgseaglatiaenYvglpyeeie
>1AZW_A
mrtlypeitpyqqgslkvddrhtlyfeqcgnphgkpvvmlhggpgggcndkmrrfhdpakyrivlfdqrgsgr
stphadlvdnttwdlvadierlrthlgvdrwqvfggSwgstlalayaqthpqqvtelvlrgifllrrfelewf
yqegasrlfpdawehylnaippveradlmsafhrrltsddeatrlaaakawsvwegatsflhvdedfvtghed
ahfalafarienhyfvnggffevedqllrdahriadipgvivhgryDvvcplqsawdlhkawpkaqlqispas
gHsafepenvdalvratdgfa
>1AF7_A
svllqmtqrlalsdahfrricqliyqragivladhkrdmvynrlvrrlralglddfgrylsmleanqnsaewq
afinalttnltaffReahhfpilaeharrrhgeyrvwsaaastgeepysiaitladalgmapgrwkvfasDid
tevlekarsgiyrlselktlspqqlqryfmrgtgpheglvrvrqelanyvefssvnllekqynvpgpfdaifc
rnvmiyfdkttqedilrrfvpllkpdgllfaghsenfsnlvrefslrgqtvyals
>4FIV_A
vgttttlekrpeilifvngypikfllDTgaditilnrrdfqvknsiengrqnmigvgggkrgtnyinvhleir
denyktqcifgnvcvlednsliqpllgrdnmikfnirlvm
>1GOG_A
asapigsaisrnnwavtcdsaqsgnecnkaidgnkdtfwhtfygangdpkpphtytidmkttqnvnglsmlpr
qdgnqngwigrhevylssdgtnwgspvasgswfadsttkysnfetrparyvrlvaiteangqpwtsiaeinvf
qassytapqpglgrwgptidlpivpaaaaieptsgrvlmwssyrndafggspggitltsswdpstgivsdrtv
tvtkhdmfCpgismdgngqivvtggndakktslydsssdswipgpdmqvargYqssatmsdgrvftiggsWsg
gvfekngevyspssktwtslpnakvnpmltadkqglyrsdnhawlfgwkkgsvfqagpstamnwyytsgsgdv
ksagkrqsnrgvapdamcgnavmydavkgkiltfggspdyqdsdattnahiitlgepgtspntvfasnglyfa
rtfhtsvvlpdgstfitggqrrgipfedstpvftpeiyvpeqdtfykqnpnsivrvYhsislllpdgrvfngg
gglcgdcttnhfdaqiftpnylynsngnlatrpkitrtstqsvkvggritistdssiskaslirygtathtvn
tdqrripltltnnggnsysfqvpsdsgvalpgywmlfvmnsagvpsvastirvtq
>1ZPU_C
ethtfnwttgwdyrnvdglksrpvitcngqfpwpditvnkgdrvqiyltngmnntntsmhfhglfqngtasmd
gvpfltqcpiapgstmlynftvdynvgtywyhshtdgqyedgmkglfiikddsfpydydeelslslsewyhdl
vtdltksfmsvynptgaepipqnlivnntmnltwevqpdttyllrivnvggfvsqyfwiedhemtvveidgit
teknvtdmlyitvaqrytvlvhtkndtdknfaimqkfddtmldvipsdlqlnatsymvynktaalptqnyvds
idnflddfylqpyekeaiygepdhvitvdvvmdnlkngvnyaffnnitytapkvptlmtvlssgdqannseiy
gsnthtfilekdeiveivlnnqdtgthpfhlhghafqtiqrdrtyddalgevphsfdpdnhpafpeypmrrdt
lyvrpqsnfvirfkadnpgvwffHChiewhllqglglvlvedpfgiqdahsqqlsenhlevcqscsvategna
aantldltdltgenvqha
>1B65_E
kprardlglpftgvtgpynaitdvdgvgvgfqtiieneprpgrkrparsgvtailphmqsetpvpvyagvhrf
ngngemtgthwiedggyflgpvvitnthgigmahhatvrwmvdryastyqtddflwimpvvaetYdgalndin
gfpvteadvrkaldnvasgpvqegncgggtgmitygfkggtgtasrvvefggrsftigalvqaNhgqrdwlti
agvpvgqhmrdgtpqsqlSiivvlatdlplmphqlkrlarrasigigrngtpggnnSGdifiafstanqrpmq
hrsapfldvemvndepldtvylaavdsveeavvnamiaaedmggtpfdrllvqaidherlravlrqygrla
>1URO_A
meanglgpqgfpelkndtflraawgeetdytpvwcmrqagrylpefretraaqdffstcrspeacceltlqpl
rrfpldaaiifsDilvvpqalgmevtmvpgkgpsfpeplreeqdlerlrdpevvaselgyvfqaitltrqrla
grvpligfagapwtlmtYmvegggsstmaqakrwlyqrpqashqllriltdalvpylvgqvvagaqalqlfes
haghlgpqlfnkfalpyirdvakqvkarlreaglapvpmiifakdghfaleelaqagyevvgldwtvapkkar
ecvgktvtlqgnldpcalyaseeeigqlvkqmlddfgphryianlghglypdmdpehvgafvdavhkhsrllr
qn
>1IMA_A
```

madpwqecmdyavtlarqagevvceaiknemnvmlksspvdlvtatdqkvekmlissikekypshsfigEesv
aageksiltdnptwiidpidgTtnfvhrfpfvavsigfavnkkiefgvvyscvegkmytarkgkgafcngqkl
qvsqqeditksllvtelgssrtpetvrmvlsnmeklfcipvhgirsvgtaavnmclvatggadayyemgihcw
dvagagiivteaggvlmdvtggpfdlmsrrviaannrilaeriakeiqviplqrdded
>1F7L_A
giygiglditeIkriasmagrqkrfaeriltrseldqyyelsekrkneflagrfaakeafsKafgtgigrqls
fqdieirkdqngkpyiictklspaavhvsitHtkeyaaaqvvierlss
>1P84_A
aevtqlsngivvatehnpsahtasvgvvfgsgaanenpynngvsnlwKniflskensavaakeglalssnisr
dfqsyivsslpgstdksldflnqsfiqqkanllsssnfeatkksvlkqvqdfedndhpnrvlehlhstafqnt
plslptrgtleslenlvvadlesfannhflnsnavvvgtgnikhedlvnsiesknlslqtgtkpvlkkkaafl
gsevrlrddtlpkawislavegepvnspnyfvaklaaqifgsynafepasrlqqgiklldniqeyqlcdnfnhf
slsykdsglwgfstatrnvtmiddlihftlkqwnrltisvtdteveraksllklqlgqlyesgnpvndanllg
aevlikgsklslgeafkkidaitvkdvkawagkrlwdqdiaiagtgqiegllldymrirsdmsmmrw
>1FUG_B
akhlftsesvsegHpdkiadqisdavldaileqdpkarvacetyvktgmvlvggeittsawvdieeitrntvr
eigyvhsdmgfdanscavlsaigkqspdinqgvdradpleqgagdqglmfgyatnetdvlmpapityahrlvq
rqaevrkngtlpwlrpdaKsqvtfqyddgkivgidavvlstqhseeidqkslqeavmeeiikpilpaewltsa
tkffinptgrfviggpmgdcgltgRKiivdtyggmarhgggafsgKdpsKvDrsaayaaryvaknivaaglad
rceiqvsyaigvaeptsimvetfgtekvpseqltllvreffdlrpygliqmldllhpiyketaayghfgrehf
pwektdkaqllrdaaglk
>1SML_A
aevplpqlraytvdaswlqpmaplqiadhtwqigtedltallvqtpdgavlldggmpqmashlldnmkargvt
prdlrlillshahadhagpvaelkrrtgakvaanaesavllarggsddlhfgdgityppanadrivmdgevit
vggivftahfmaghtpgstawtwtdtrngkpvriayadslsapgYqlqgnpryphliedyrrsfatvralpcd
vlltphpgasnwdyaagaragakaltckayadaaeqkfdgqlaketagar
>1APY_B
TigmvvihktghiaagtsTngikfkihgrvgdspipgagayaddtagaaaaTGngdilmrflpsyqaveymrr
gedptiacqkvisriqkhfpeffgavicanvtgsygaacnklstftqfsfmvynseknqpteekvdci
>1ORO_A
mkpyqrqfiefalskqvlkfgeftlksgrkspyffnaglfntgrdlallgrfyaealvdsgiefdllfgpayk
gipiatttavalaehhdldlpycfnrkeaKdhgeggnlvgsalqgrvmlvddvitagtairesmeiiqangat
lagvlisldrqergrgeisaiqeverdynckvisiitlkdliayleekpemaehlaavkayreefgv
>1PS1_A
mpqdvdfhiplpgrqspdharaeaeqlawprslglirsdaaaerhlrggyadlasrfyphatgadldlgvdlm
swfFlfddlfdgprgenpedtkqltdqvaaaldgplpdtappiahgfadiwrrtcegmtpawcarsarhwrny
fdgyvdeaesrfwnapcdsaaqylamrrhtigvqptvdlaeragrfevphrvfdsavmsamlqiavdvnllln
diaslekeeargeqnnmvmilrrehgwsksrsvshmqnevrarleqylllesclpkvgeiyqldtaerealer
yrtdavrtvirgsydwHrssgrydaefalaagaqgyleelgssah
>1CQJ_A
silidkntkvicqgftgsqgtfhseqaiaygtkmvggvtpgkggtthlglpvfntvreavaatgatasviyvp
apfckdsileaidagikliititegiptldmltvkvkldeagvrmigpncpgvitpgeckigiqpghihkpgk
vgivsrsgtltyeavkqttdygfgqstcvgiggdpipgsnfidilemfekdpqteaivmigEiggsaeeeaaa
yikehvtkpvvgyiagvtapkgkrmgHagaiiaggkgtadekfaaleaagvktvrsladigealktv
>1OS7_A
mserlsitplgpyigaqisgadltrplsdnqfeqlyhavlrhqvvflrdqaitpqqqralaqrfgelhihpvy
phaegvdeiivldthndnppdndnwhtdvtfietppagailaakelpstggdtlwtsgiaayealsvpfrqll
sglraehdfrksfpeykyrkteeehqrwreavaknppllhpvvrthpvsgkqalfvnegfttrivdvsekese
allsflfahitkpefqvrwrwqpndiaiwdnrvtqhyanadylpqrrimhRatilgdkpfyrag
>1S9C_C
gfagaigqklppfsyayteleaimyalgvgasikdpkdlkfiyegssdfsclptfgviigqksmglaeipgls
infakvlhgeqylelykplpragklkceavvadvldkgsgvviimdvysyselichnqfslflkrtsdkvkva
vaipnrppdavltdttslnqaalyrlsgDwnplHidpnfaslagfdkpIlhGlctfgfsarrvlqqfadndvs
rfkavkarfakpvypgqtlqtemwkegnrihfqtkvqetgdivisnayvdla

>1DIZ_A
mytlnwqppydwswmlgflaaravssvetvadsyyarslavgeyrgvvtaipdiarhtlhinlsaglepvaae
clakmsrlfdlqcnpqivngalgrlgaarpglrlpgcvdafeqgvrailgqlvsvamaakltarvaqlygerl
ddfpeyicfptpqrlaaadpqalkalgmplkraealihlanaalegtlpmtipgdveqamktlqtfpgigrwt
anYfalrgwqakdvflpdDylikqrfpgmtpaqirryaerwkpwrsyallhiWytegwqpdea
>4KBP_D
rdmpldsdvfrvppgynapqqvhitqgdlvgramiiswvtmdepgssavrywsekngrkriakgkmstyrffn
yssgfihhttirklkyntkyyyevglrnttrrfsfitppqtgldvpytfgligdlgqsfdsnttlshyelspk
kgqtvlfvgdlsyadrypnhdnvrwdtwgrftersvayqpwiwtagnHeiefapeinetepfkpfsyryhvpy
easqstspfwysikrasahiivlssysaygrgtpqytwlkkelrkvkrsetpwlivlmhsplynsynHHfmeg
eamrtkfeawfvkykvdvvfaghvhayerservsniaykitdglctpvkdqsapvyitigdagnygvidsnmi
qpqpeysafreasfghgmfdiknrthahfswnrnqdgvaveadsvwffnrhwypvddst
>2IUA_C
qstysleqladflkvefqgngatllsgveeieeaktahitfldnekyakhlksseagaiiisrtqfqkyrdln
knflitsespslvfqkclelfitpvdsgfpgihptavihptaiiedhvciepyavvcqhahvgsachigsgsv
igaystvgehsyihprvvirervsigkrviiqpgavigscgfgyvtsafgqhkhlkhlgkviieddveigant
tidrgrfkhsvvregskidnlvqiaHQvevgqhsmivaqagiaGstkignhviiggqagitghiciadhvimm
aqtgvtksitspgiyggaparpyqeihrqvakvrnlprleeriaaleklvqkle
>1A2O_A
mskirvlsvddsalmrqimteiinshsdmemvatapdplvardlikkfnpdvltldvemprmdgldfleklmr
lrpmpvvmvssltgkgsevtlralelgaidfvtkpqlgiregmlaysemiaekvrtaarariaahkpmaaptt
lkagpllssekliaigaSTggteairhvlqplplsspaviitqHmppgftrsfaerlnklcqisvkeaedger
vlpghayiapgdkhmelarsganyqikihdgppvnrhrpsvdvlfhsvakhagrnavgviltgMgnDgaagml
amyqagawtiaqneascvvfgmpreainmggvsevvdlsqvsqqmlakisagqai
>1DB3_A
skvalitgvtgqdgsylaefllekgyevhgikrrassfntervdhiyqdphtcnpkfhlhygdlsdtsnltri
lrevqpdevynlgamshvavsfespeytadvdamgtlrlleairflglekktrfyqasTsElyglvqeipqke
ttpfyprspYavaKlyaywitvnyresygmyacngilfnhesprrgetfvtrkitraianiaqglesclylgn
mdslrdwghakdyvkmqwmmlqqeqpedfviatgvqysvrqfvemaaaqlgiklrfegtgveekgivvsvtgh
dapgvkpgdviiavdpryfrpaevetllgdptkaheklgwkpeitlremvsemvandleaakkhsllkshgyd
vaiales
>1GIM_A
gnnvvvlgtqwgDegkgkivdllterakyvvryqgghnagHtlvingektvlhlipsgilrenvtsiigngvv
lspaalmkemkeledrgipvrerlllseacplildyhvaldnarekargakaigttgrgigpayedkvarrgl
rvgdlfdketfaeklkevmeyhnfqlvnyykaeavdyqkvlddtmavadiltsmvvdvsdlldqarqrgdfvm
fegaQgtlldidhgtypyvtssnttaggvatgsglgpryvdyvlgilkaystrvgagpfptelfdetgeflck
qgnefgattgrrrrtgwldtvavrravqlnslsgfcltkldvldglkevklcvayrmpdgrevtttplaaddw
kgvepiyetmpgwsestfgvkdrsglpqaalnyikrieeltgvpidiistgpdrtetmilrdpfda
>2OAT_A
mfsklahlqrfavlsrgvhssvasatsvatkktvqgpptsddifereykygahnyhplpvalergkgiylwdv
egrkyfdflssysavnqghchpkivnalksqvdkltltsrafynnvlgeyeeyitklfnyhkvlpmntgveag
etacklarkwgytvkgiqkykakivfaagnFwgrtlsaissstdptsydgfgpfmpgfdiipyndlpaleral
qdpnvaafmvepiqgeagvvvpdpgylmgvrelctrhqvlfiaDeiqtglartgrwlavdyenvrpdivllgK
alsgglypvsavlcdddimltikpgehgstyggnplgcrvaiaalevleeenlaenadklgiilrnelmklps
dvvtavrgkgllnaiviketkdwdawkvclrlrdngllakpthgdiirfapplvikedelresieiinktils
f
>2F9R_B
adnrrpiwnlaHmvnavaqipdfldlganaleaDvtfkgsvptytyHGtpcDfgrdcirweyfnvflktlrey
ttpgnakyrdgfilfvldlKtgslsndqvrpagenvakellqnywnngnnggrayvvlslpdighyefvrgfk
evlkkeghedllekvgydfsgpylpslptldatheaykkagvdghiwlsdgltnfsplgdmarlkeaiksrds
angfinkiyyWsvDkvsttkaaldvgvdgimtNypnvligvlkesgyndkyrlatyddnpwetfkn
>1AOP_A
leprhamllRcrlpggvittkqwqaidkfagentiygsirltnrqtfqfhgilkknvkpvhqmlhsvgldala
tandmnRnvlctsnpyesqlhaeayewakkisehllprtrayaeiwldqekvattdeepilgqtylprKfKtt

vvippqndidlhandmnfvaiaengklvgfnllvggglsiehgnkktyartasefgylplehtlavaeavvtt
qrdwgnrtdrknaktkytlervgvetfkaeverragikfepirpyeftgrgdrigwvkgiddnwhltlfieng
rildyparplktglleiakihkgdfritanqnliiagvpesekakiekiakesglmnavtpqrensmacvsfp
tcplamaeaerflpsfidnidnlmakhgvsdehivmrvtgcpngCgramlaevglvgkapgrynlhlggnrig
triprmykenitepeilasldeligrwakereagegfgdftvragiirpvldpardlwd
>1A0I_A
vniktnpfkavsfvesaikkaldnagyliaeiKydgvrgnicvdntansywlsrvsktipalehlngfdvrwk
rllnddrcfykdgfmldgelmvkgvdfntgsgllrtkwtdtknqefheelfvepirkkdkvpfklhtghlhik
lyailplhivesgedcdvmtllmqehvknmlpllqeyfpeiewqaaesyevydmvelqqlyeqkraeghegli
vkdpmciykrgkksgwwkmkpeneadgiiqglvwgtkglanegkvigfevllesgrlvnatnisralmdefte
tvkeatlsqwgffspygigdndactinpydgwacqisymeetpdgslrhpsfvmfr
>1BD3_A
qeesilqdiitrfpnvvlmkqtaqlrammtiirdketpkeefvfyadrlirllieealnelpfqkkevttpld
vsyhgvsfyskicgvsivragesmesglravcrgvrigkiliqRdetTaepkliyeklpadirerwvmlldpm
catagsvckaievllrlgvkeeriifvnilaapqgiervfkeypkvrmvtaavdiclnsryyivpgigDfgdr
yfgtm
>1JHF_A
mkaltarqqevfdlirdhisqtgmpptraeiaqrlgfrspnaaeehlkalarkgvieivsgasrgirllqeee
eglplvgrvaadepllaqqhieghyqvdpslfkpnadfllrvsgMSmkdigimDgdllavhktqdvrngqvvv
ariddEvtvKrlkkqgnkvellpensefkpivvdlrqqsftieglavgvirngdwl
>2TS1_A
mdllaelqwrglvnqttdedglrkllneervtlycgfdptadslhighlatiltmrrfqqaghrpialvggat
gligdpsgKkseRtlnaketveawsarikeqlgrfldfeadgnpakiknnydwigpldvitflrdvgkhfsvn
ymmakesvqsrietgisftefsymmlqaydflrlyetegcrlqiggsdqwgnitaglelirktkgearafglt
iplvtkadgtKfgKtesgtiwldkektspyefyqfwintddrdvirylkyftflskeeiealeqelreapekr
aaqktlaeevtklvhgeealrqairisealfsgdianltaaeieqgfkdvpsfvheggdvplvellvsagisp
skrqarediqngaiyvngerlqdvgailtaehrlegrftvirrgkkkyylirya
>1NSF_A
kpafgtnqedyasyimngiikwgdpvtrvlddgellvqqtknsdrtplvsvllegpphsgKtalaakiaeesn
fpfikicspdkmigfsetakcqamkkifddayksqlscvvvddierlldyvpigprfsnlvlqallvllKkap
pqgrklliigttsrkdvlqememlnafsttihvpniatgeqllealellgnfkdkerttiaqqvkgkkvwigi
Kkllmliemslqmdpeyrvrkflallre
>1DNP_B
tthlvwfrqdlrlhdnlalaaacrnssarvlalyiatprqwathnmsprqaelinaqlnglqialaekgipll
frevddfvasveivkqvcaensvthlfynyqyevnerardveveralrnvvcegfddsvilppgavmtgnhem
ykvftpfknawlkrlregmpecvaapkvrssgsiepspsitlnyprqsfdtahfpveekaaiaqlrqfcqnga
geyeqqrdfpavegtsrlsaslatgglsprqclhrllaeqpqaldggagsvwlneliwrefyrhlityhpslc
khrpfiawtdrvqWqsnpahlqawqegktgypivdaamrqlnstgwmhnrlrmitasflvkdllidWregery
fmsqlidgdlaannggWqwaastgtdaapyfrifnpttqgekfdhegefirqwlpelrdvpgkvvhepwkwaq
kagvtldypqpivehkearvqtlaayeaark
>1G99_A
mkvlvinagSsslkyqlidmtnesalavglcerigidnsiitqkkfdgkklekltdlpthkdaleevvkaltd
defgvikdmgeinavghRvvhggekfttsalydegvekaikdcfelaplhnppnmmgisacaeimpgtpmviv
fDtafhqtmppyaymyalpydlyekhgvrkygfhgtshkyvaeraalmlgkpaeetkiitchlgnGssitave
ggksvetsmgftpleglamgtRcgsidpaivpflmekeglttreidtlmnkksgvlgvsglsndfrdldeaas
kgnrkaelaleifaykvkkfigeysavlngadavvftagigensasirkriltgldgigikiddeknkirgqe
idistpdakvrvfviptneelaiaretkeivetevklrssipv
>1CZF_A
mhsfasllayglvagatfasaspieardsctfttaaaakagkakcstitlnnievpagttldltgltsgtkvi
fegtttfqyeewagplismsgehitvtgasghlincdgarwwdgkgtsgkkkpkffyahgldsssitglnikn
tplmafsvqanditftdvtinnadgdtqgghntDafdvgnsvgvniikpwvhnqDDclavnsgeniwftggtc
iggHglsigsvgdrsnnvvknvtiehstvsnsenavriktisgatgsvseitysnivmsgisdygvviqqdye
dgkptgkptngvtiqdvklesvtgsvdsgateiyllcgsgscsdwtwddvkvtggkkstacknfpsvasc
>1DNK_A

```
lkiaafnirtfgetkmsnatlasyivrivrrydivliqevrdshlvavgklldylnqddpntyhyvvseplgr
nsykErylflfrpnkvsvldtyqyddgcescgndsfsrepavvkfsshstkvkefaivalHsapsdavaeins
lydvyldvqqkwhlndvmlmgdfnadcsyvtssqwssirlrtsstfqwlipdsadttatstncayDrivvags
llqssvvpgsaapfdfqaayglsnemalaisdHypvevtlt
>1CHK_A
agaglddphkkeiamelvssaEnssldwkaqykyiedigDgrgytggiigfcsgtgdmlelvqhytdlepgni
lakylpalkkvngsashsglgtpftkdwataakdtvfqqaqnderdrvyfdpavsqakadglralgqfayyda
ivmhgpgndptsfggirktamkkartpaqggdettylngfldarkaamlteaahddtsrvdteqrvflkagnl
dlnpplkwktygdpyvins
>12AS_A
mktayiakqrqisfvkshfsrqleerlglievqapilsrvgdgtqDnlsgaekavqvkvkalpdaqfevvhsl
akwkrqtlgqhdfsageglythmkalRpdedrlsplhsvyvdQwdwervmgdgerqfstlkstveaiwagika
teaavseefglapflpdqihfvhsqellsrypdldakgreraiakdlgavflvgiggklsdghrhdvrapdyd
dwstpselghaglngdilvwnpvledafelssmgirvdadtlkhqlaltgdedrlelewhqallrgempqtig
ggigqsrltmlllqlphigqvqagvwpaavresvpsll
>1B6B_B
nefrcltpedaagvfeiereafdevqhfltlcpelslgwfvegrlvafiigSlwdeerltqeslaLhrprghs
ahlHaLavhrsfrqqgkgsvllwrylhhvgaqpavrravlmcedalvpfYqrfgfhpagpcaivvgsltftem
hcslrghaal
>1F8X_A
mpkktiYfgagwftdrqnkaykeamealkenptidlensyvpldnqykgirvdehpeylhdkvwatatynnDl
ngiktndimlgvyipdeeDvglgmElgyalsqgkyvllvipdedygkpinlmswgvsdnvikmsqlkdfnfnk
prfdfyegavy
>1FCB_A
epkldmnkqkispaevakhnkpddcwvvingyvydltrflpnhpggqdvikfnagkdvtaifeplhapnvidk
yiapekklgplqgsmppelvcppyapgetkediarkeqlksllppldniinlydfeylasqtltkqawaYyss
gandevthrenhnayhriffkpkilvdvrkvdistdmlgshvdvpfyvsatalcklgnplegekdvargcgqg
vtkvpqmistlascspeeiieaapsdkqiqwyqlYvnsdrkitddlvknveklgvkalfvtvDapslgqrekd
mklkfsntkagpkamkktnveesqgasralskfidpsltwkdieelkkktklpivikgvqrtedvikaaeigv
sgvvlsnHggRqldfsrapievlaetmpileqrnlkdklevfvdggvrrgtdvlkalclgakgvglgrpflya
nscygrngvekaieilrdeiemsmrllgvtsiaelkpdlldlstlkartvgvpndvlynevyegptltefeda
>1GCU_A
mdaepkrkfgvvvvgvgragsvrlrdlkdprsaaflnligfvsrrelgsldevrqisledalrsqeidvayic
sessshedyirqflqagkhvlvEYpmtlsfaaaqelwelaaqkgrvlheEhvEllmeefeflrrevlgkellk
gslrftaspleeerfgfpafsgiSRltwlvslfgelslisatleerkedqymkmtvqletqnkgllswieekg
pglkrnryvnfqftsgsleevpsvgvnkniflkdqdifvqklldqvsaedlaaekkrimhclglasdiqklch
qkk
>1CA2_A
hwgygkhngpehwhkdfpiakgerqspvdidthtakydpslkplsvsydqatslrilnngHafnvefddsqdk
avlkggpldgtyrliqfhfhwgsldgqgsEhtvdkkkyaaelhlvhwntkygdfgkavqqpdglavlgiflkv
gsakpglqkvvdvldsiktkgksadftnfdprgllpesldywtypgslTtppllecvtwivlkepisvsseqv
lkfrklnfngegepeelmvdnwrpaqplknrqikasf
>1GSA_A
miklgivmdpianinikkdssfamlleaqrrgyelhymemgdlylingearahtrtlnvkqnyeewfsfvgeq
dlpladldvilmrkdppfdtefiyatyileraeekgtlivnkpqslrdcneklftawfsdltpetlvtrnkaq
lkafwekhsdiilKpldgmggasifrvkegdpnlgviaetltehgtrycmaqnylpaikdgdkRvlvvdgepv
pyclaRipqggetrgnlaaggrgeprpltesdwkiarqigptlkekglifvgldiigdrlteinvtsptcire
ieaefpvsitgmlmdaiearlqqq
>1CGK_A
mvsvseirkaqraegpatilaigtanpancveqstypdfyfkitnsehktelkekfqrmcdksmikrrymylt
eeilkenpnvceymapsldarqdmvvvevprlgkeaavkaikewgqpkskithlivcttsgvdmpgadyqltk
llglrpyvkrymmyqqgCfaggtvlrlakdlaennkgarvlvvcsevtavtfrgpsdthldslvgqalFgdga
aalivgsdpvpeiekpifemvwtaqtiapdsegaidghlreagltfhllkdvpgivsknitkalveafeplgi
```

```
sdynsifwiaHpggpaildqveqklalkpekmnatrevlseygNmssacvlfildemrkkstqnglkttgegl
ewgvlfgfgpgltietvvlrsvai
>1KEZ_B
ssalrdgyrqagvsgrvrsyldllaglsdfrehfdgsdgfsldlvdmadgpgevtviccagtaaisgpheftr
lagalrgiapvravpqpgyeegeplpssmaavaavqadavirtqgdkpfvvaghSAgalmayalatelldrgh
pprgvvliDvyppghqdamnawleeltatlfdretvrmddtrltalgaydrltgqwrpretglptllvsagep
mgpwpddswkptwpfehdtvavpgdHftmvqehadaiarhidawlggg
>1EQ2_A
miivtggagfigsnivkalndkgitdilvvdnlkdgtkfvnlvdlniadymdkedfliqimageefgdveaif
hegacssttewdgkymmdnnyqyskellhyclereipflyasSaatyggrtsdfiesreyekplnvYgysKfl
fdeyvrqilpeansqivgfryfnvygpreghkgsmasvafhlntqlnngespklfegsenfkrdfvyvgdvad
vnlwflengvsgifnlgtgraesfqavadatlayhkkgqieyipfpdklkgryqaftqadltnlraagydkpf
ktvaegvteymawlnrda
>1DO8_A
ikekgkpllnprtnkgaftlqerqlglqgllppkietqdiqalrfhrnlkktsplekyiyigiqerneklfyr
ilqddieslpivYtptvglacsqyghifrrpkglfisisdrghvrsivdnwpenhvkavvvtdgerilglgdl
gvyggipvgKlclytacagirpdrclpvcidvgtdniallkdpfyglyqkrdrtqqyddlidefkaitdrygr
ntliqfedfgnhnafrflrkyrekyctfnDdiqgtaavalagllaaqkviskpisehkilflgageaalgian
livsvenglseqeaqkkiwfdkygllvkgrkakidsyqepfthsapesipdtfedavnilkpstiigvagagr
lftpdviraasinerpvifalsnptaqaectaeeaytltegrclfasgspfgpvkltdgrvftpgqgnnvyif
pgvalavilcntrhisdsvfleaakaltsqltdeelaqgrlypplaniqevsiniaikvteylyankafrype
pedkakyvkertwrseydsllpdvyewp
>1F6D_A
mkvltvfgtrpeaikmaplvhalakdpffeakvcvtaqhremldqvlklfsivpdydlnimqpgqglteitcr
ileglkpilaefkpdvvlvhgDttttlatslaafyqripvghvEaglrtgdlyspwpEeanrtltghlamyhf
sptetsrqnllrenvadsrifitgntvidallwvrdqvmssdklrselaanypfidpdkkmilvtgHrresfg
rgfeeichaladiatthqdiqivypvhlnpnvrepvnrilghvknvilidpqeylpfvwlmnhawliltdsgg
iqeeapslgkpvlvmrdtterpeavtagtvrlvgtdkqriveevtrllkdeneyqamsrahnpygdgqacsri
lealknnrisl
>1HTO_X
tektpddvfklakdekveyvdvrfcdlpgimqhftipasafdksvfddglafDgssirgfqsihesdmlllpd
petaridpfraaktlninffvhdpftlepysrdprniarkaenylistgiadtayfgaeaefyifdsvsfdsr
angsfyevdaisgwwntgaateadgspnrgykvrhkggyfpvapndqyvdlrdkmltnlinsgfilekghhev
gsggqaeinyqfnsllhaaddmqlykyiikntawqngktvtfmpkplfgdngsgmhchqslwkdgaplmydet
gyaglsdtarhyigglhhapsllaftnptvnsykrlvpgyEapinlvysqrnRsacvripitgsnpkakrle
frspdssgnpylafsamlmagldgiknkiepqapvdkdlyelppeeaasipqtptqlsdvidrleadheylte
ggvftndlietwisfkreneiepvnirphpyefalyydv
>1DLI_A
mkiavagsgyvglslgvllslqnevtivdilpskvdkinnglspiqdeyieyylkskqlsikatldskaayke
aelviiatptnynsrinyfdtqhvetvikevlsvnshatliiksTipigfitemrqkfqtdriifspeflrEs
kalydnlypsriivsceendspkvkadaekfalllksaakknnvpvlimgaseaeavKlfaNtylalrvayfn
eldtyaesrklnshmiiqgisyddrigmhynnpsfgyggyClpkDtkqllanynnipqtlieaivssnnvrks
yiakqiinvlkeqespvkvvgvyrlimksnsdnfresaikdvidilkskdikiiiyepmlnklesedqsvlvn
dlenfkkqaniivtnrydnelqdvknkvysrdifgrd
>1O98_A
mskkpvaliildgfalrdetygnavaqankpnfdrywneyphttlkacgeavglpegqmgnSevghlnigagr
ivyqsltriniairegefdrnetflaamnhvkqhgtslhlfgllsdggvhshihhlyallrlaakegvkrvyi
hgfldgrDvgpqtapqyikelqekikeygvgeiatlsgryysmdrdkrwdrvekayramvygegptyrdplec
iedsykhgiydefvlpsvivredgrpvatiqdndaiifynfRpdraiqisntftnedfrefdrgpkhpkhlff
vclthfsetvagyvafkptnldntigevlsqhglrqlriaetekyphvtffmsggreeefpgedrilinspkv
ptydlkpemsayevtdallkeieadkydaiilnyanpdmvghsgkleptikaveavdeclgkvvdailakggi
aiitadhgnadevltpdgkpqtahttnpvpvivtkkgiklrdggilgdlaptmldllglpqpkemtgkslivk
>1YTW_A
```

```
mrerphtsghhgagearatapstvspygpearaelssrlttlrntlapatndprylqacggeklnrfrdiqcr
rqtavradlnanyiqvgntrtiacqyplqsqleshfrmlaenrtpvlavlasssEianqrfgmpdyfrqsgty
gsitveskmtqqvglgdgimadmytltireagqktisvpvvhvgnwpDqtavssevtkalaslvdqtaetkrn
myeskgssavaddsklrpviHCragvgRTaqligamcmndsrnsqlsvedmvsqmrvqrngimvqkdeqldvl
iklaegqgrpllns
>1HRK_A
rkpktgilmlnmggpetlgdvhdfllrlfldrdlmtlpiqnklapfiakrltpkiqeqyrrigggspikiwts
kqgegmvklldelspntaphkyyigfRYvhplteeaieemerdgleraiaftqypqyscsttgsslnaiyryy
nqvgrkptmkwstidrwpthhlliqcfadhilkeldhfplekrsevvilfsaHslpmsvvnrgdpypqevsat
vqkvmerleycnpyrlvwqskvgpmpwlgpqtdesikglcergrknillvpiaftsDhiEtlyEldieysqvl
akecgvenirraeslngnplfskaladlvhshiqsnelcskqltlscplcvnpvcretksfftsqql
>2PGD_A
aqadialiglavmgqnlilnmndhgfvvcafnrtvskvddflaneakgtkvlgahsleemvsklkkprriill
vkagqavdnfieklvplldigdiiidggnseyrdtmrrcrdlkdkgilfvgsgvsgGedgarygpslmpggnk
eawphikaifqgiaakvgtgepccdwvgddgaghfvKmvhNgiEygdmqliceayhlmkdvlglghkemakaf
eewnkteldsflieitasilkfqdadgkhllpkirdsagqkgtgkwtaisaleygvpvtligeavfarclssl
kderiqaskklkgpqnipfegdkksfledirkalyaskiisyaqgfmllrqaatefgwtlnyggialmwrggc
iirsvflgkikdafdrnpglqnlllddffksavencqdswrraistgvqagipmpcfttalsfydgyrhamlp
anliqaqrdyfgahtyellakpgqfihtnwtghggsvssssyna
>1Z9H_A
lqltlyqYktCpFCskvrafldfhalpyqvvevnpvlraeikfssyrkvpilvaqegessqqlndssviisal
ktylvsgqpleeiityypamkavndqgkevtefgnkywlmlnekeaqqvysgkearteemkwrqwaddwlvhl
ispnvyrtptealasfdyivregkfgavegavakymgaaamyliskrlksrhrlqdnvredlyeaadkwvaav
gkdrpfmggqkpnladlavygvlrvmegldafddlmqhthiqpwylrveraitea
>1KAS_A
skrrvvvtglgmlspvgntvestwkallagqsgislidhfdtsayatkfaglvkdfncediisrkeqrkmdaf
iqygivagvqamqdsgleiteenatrigaaigsgigglglieenhtslmnggprkispffvpstivnmvaghl
timyglrgpsisiataCtsgvhnighaariiaygdadvmvaggaekastplgvggfgaaralstrndnpqaas
rpwdkerdgfvlgdgagmlvleeyehakkrgakiyaelvgfgmssdayhmtsppengagaalamanalrdagi
easqigyvnaHgtstpagdkaeaqavktifgeaasrvlvsstksmtgHllgaagavesiysilalrdqavppt
inldnpdegcdldfvphearqvsgmeytlcnsfgFggtngslifkki
>1N2C_C
msreevesliqevlevypekarkdrnkhlavndpavtqskkciisnkksqpglmtirgCayAgskgvvwgpik
dmihishgpvgcgqysragRrnyyigttgvnafvtmnftsdfqekdivfggdkklaklidevetlfplnkgis
vqseCpigLigddiesvskvkgaelsktivpvrcegfrgvsqslgHhiandavrdwvlgkrdedttfastpyd
vaiigdyniggdawssrilleemglrcvaqwsgdgsiseieltpkvklnlvhcyrsmnyisrhmeekygipwm
eynffgptktieslraiaakfdesiqkkceeviakykpeweavvakyrprlegkrvmlyigglrprhvigaye
dlgmevvgtgyefahnddydrtmkemgdstllyddvtgyefeefvkrikpdligsgikekfifqkmgipfrem
hswdysgpyhgfdgfaifardmdmtlnnpcwkklqapwea
>1H7A_A
srvfptqrdlmagivskhiaknmvpsfimkahesgiihvhdidyspalpftnCclvdlkgmlengfklgnaqi
etpksigvataimaqitaqvashqyggttfanvdkvlspyvkrtyakhiedaekwqiadalnyaqsktekdvy
dafqayeyevntlfssngqtpfvtitfgtgtdwtermiqkailknrikglgrdgitpifpklvmfveegvnly
kddpnydikqlalecaskrmypdiisaknnkaitgssvpvspmgCrsflsvwkdstgneildgrnNlgvvtln
lprialdsyigtqfneqkfvelfnermdlcfealmcrisslkgvkatvapilyqegafgvrlkpdddiielfk
ngrssvslgyigihelnilvgrdigreiltkmnahlkqwtertgfafslystpaEnlcyrfckldtekygsvk
dvtdkgwytnsfhvsveenitpfekisreapyhfiatgghisyvelpdmknnlkgleavwdyaaqhldyfgvn
mpvdkcftcgsthemtptengfvcsicgetdpkkmntirrtcAylgnpnerg
>1BIB_A
mkdntvplkliallangefhsgeqlgetlgmsraainkhiqtlrdwgvdvftvpgkgyslpepiqllnakqil
gqldggsvavlpvidstnqylldrigelksgdaciaeyqqagrgRrgrkwfspfganlylsmfwrleqgpaaa
iglslvigivmaevlrklgadkvrvkwpndlylqdrKlagilveltgktgdaaqivigaginmamrrveesvv
nqgwitlqeaginldrntlaamlirelraalelfeqeglapylsrwekldnfinrpvkliigdkeifgisrgi
dkqgallleqdgiikpwmggeislRsaek
```

```
>1PYM_B
vkkttqlkqlnskdlefieahnglsarivqeagfkgiwgsGLsvsaqlgvrDsneaswtqvvevlefsdasdv
pilldadtgygnfnnarrlvrkledrgvagacledklfpKtnslhdgraqpladieefalkikackdsqtdpd
fcivarveafiagwgldealkraeayrnagadailhskkadpsdieafkawnnqgpvvivptkyyktptdhfr
dgvsviwanhnlrasvsaiqqttkqiyddqslvnvedkivsvkeifrlqrddelvqaedkylp
>1M9C_A
mvnptvffdiavdgeplgrvsfelfadkvpktaenfralstgekgfgykgscfhRiipgFmcQggdftrhngt
ggksiygekfedenfilkhtgpgilsmaNagpntngsqfFictaktewLdgkhvvfgkvkegmniveamerfg
srngktskkitiadcgqle
>2DLN_A
mtdkiavllggtsaErevslnsgaavlaglreggidaypvdpkevdvtqlksmgfqkvfialhgrggedgtlq
gmlelmglpytgsgvmasalsmdklrskllwqgaglpvapwvaltraefekglsdkqlaeisalglpvivkps
regSsvgmskvvaenalqdalrlafqhdeevliekwlsgpeftvailgeeilpsiriqpsgtfydyeakYlsd
etqyfcpagleasqeanlqalvlkawttlgckgwgRidvmldsdgqfylleantspGmtshslvpmaarqagm
sfsqlvvrilelad
>2A86_B
aipafhpgelnvysapgdvadvsralrltgrrvmlvptMgalHegHlalvraakrvpgsvvvvsifvnpmqfr
tpdddlaqlraegveiaftpttaamypdglrttvqpgplaaeleggprpthfagvltvvlkllqivrpdrvff
geKdyqqlvlirqlvadfnldvavvgvptvreadglamSSRnryldpaqraaavalsaaltaaahaatagaqa
aldaaravldaapgvavdylelrdiglgpmplngsgrllvaarlgttrlldniaieig
>1SES_B
mvdlkrlrqepevfhrairekgvaldleallaldrevqelkkrlqevqternqvakrvpkappeekealiarg
kalgeeakrleealrekearleallqvplppwpgapvggeeanreikrvggppefsfppldhvalmekngww
eprisqvsgsrsyalkgdlalyelallrfamdfmarrgflpmtlpsyarekaflgtghfpayrdqvwaiaetd
lyltgtaevvlnalhsgeilpyealplryagyapafRsEagSfgkDvrglmRvhqfhkveqyvlteasleasd
rafqellenaeeilrllelpyrlvevatgdmgpgkwrqvdievylpsegryrethscsalldwqarranlryr
dpegrvryaytlnntalatprilamllenhqlqdgrvrvpqalipymgkevlepcg
>1OK4_J
nltekflrifarrgksiilayDhgiehgpadfmdnpdsadpeyilrlardagfdgvvfqrgiaekyydgsvpl
ilklngkttlyngepvsvancsveeavslgasavgytiypgsgfewkmfeelarikrdavkfdlplvvwsYpr
ggkvvnetapeivayaarialelgadamKikytgdpktfswavkvagkvpvlmsggpktkteedflkqvegvl
eagalgiavgrnvwqrrdalkfaralaelvy
>1NN4_A
hhhssgltprgsqmkkiafgcDHvgfilkheivahlvergvevidkgtwssertdyphyasqvalavaggevd
ggiliCgtgvgisiaankfagiravvcsepysaqlsrqhndtnvlafgsrvvglelakmivdawlgaqyeggr
hqqRveaitaieq
>1W1O_A
mavvyylllagliacshalaagtpalgddrgrpwpaslaalaldgklrtdsnataaastdfgnitsalpaavl
ypsstgdlvallsaanstpgwpytiafrgrgHslmgqafapggvvvnmaslgdaaapprinvsadgryvdagg
eqvwidvlraslargvaprswtDylyltvggtlsnagisgqafrhgpqisnvlemdvitghgemvtcskqlna
dlfdavlgglgqfgvitrariavepapararwvrfvytdfaafsadqerltaprpggggasfgpmsyvEgsvf
vnqslatdlantgfftdadvarivalagernattvysieatlnydnataaaaavdqelasvlgtlsyvegfaf
qrdvayaafldrvhgeevalnklglwrvphpwlnmfvprsriadfdrgvfkgilqgtdivgplivyplnksmw
ddgmsaatpsedvfyavsllfssvapndlarlqeqnrrilrfcdlagiqyktylarhtdrsdwvrhfgaakwn
rfvemknkydpkrllspgqdifn
>1ALD_A
pyqypaltpeqkkelsdiahrivapgkgilaaDestgsiakrlqsigtenteenrrfyrqllltaddrvnpci
ggvilfhetlyqkaddgrpfpqvikskggvvgikvdkgvvplagtngetttqgldglsercaqykkdgadfak
wrcvlkigehtpsalaimenanvlaryasicqqngivpivEpeilpdgdhdlkrcqyvtekvlaavykalsdh
hiylegtllKpnmvtpghactqkfsheeiamatvtalrrtvppavtgitflsggqseeeasinlnainkcpll
kpwaltfsygralqasalkawggkkenlkaaqeeyvkralanslacqgkytpsgqagaaaseslfvsnhay
>1Q6L_B
```

```
slpmlqvaldnqtmdsayettrliaeevdiievgTIlcvgegvravrdlkalyphkivladaKiaDAgkiLsr
mcfeanadwvtviccadintakgaldvakefngdvqiEltgywtweqaqqwrdagigqvvyHrsRdaqaagva
wgeaditaikrlsdmgfkvtvtgglaledlplfkgipihvfiagrsirdaaspveaarqfkrsiaelwg
>1DE6_A
tqleqawelakqrfaavgidveealrqldrlpvsmhcwqgddvsgfenpegsltggiqatgnypgkarnasel
radleqamrlipgpkrlnlhaiylesdtpvsrdqikpehfknwvewakanqlgldfnpscfshplsadgftls
haddsirqfwidhckasrrvsayfgeqlgtpsvmniwipdgmkditvdrlaprqrllaaldeviseklnpahh
idavesKlfgigaesytvgsnefymgyatsrqtalcldagHfhptevisdkisaamlyvpqlllhvsrpvrwD
sdhvvlldde tqaiaseivrhdlfdrvhigldffdasinriaawvigtrnmkkallralleptaelrkleapg
dytarlalleeqkslpwqavwemycqrhdtpagsewlesvrayekeilsrr
>1DI1_A
tppptqwsylchprvkevqdevdgyflenwkfpsfkavrtfldakfsevtclYfplalddrihfacrlltvlF
liddvlehmsfadgeaynnrlipisrgdvlpdrtkpeefilydlwesmrahdaelanevleptfvFmraqtdr
arlsihelghyleyrekdvgkallsalmrfsmglrlsadelqdmkaleancakqlsvvndiysydkeeeasrt
ghkegaflcsavkvlaeesklgipatkrvlwsmtrewetvhdeivaekiaspdgcseaakaymkgleyqmsgn
eqWskttr
>1L7N_B
ekkkklilfDFDstlvnnetideiareagveeevkkitkeaegklnfeqslrkrvsllkdlpiekvekaikri
tptegaeetikelknrgyvvavvsGgfdiavnkikeklgldyafanrlivkdgkltgdvegevlkenaKgeil
ekiakieginledtvavgdganDisfkkaglkiafcakpilkekadiciekrdlreilkyik
>1BD0_A
mndfhrdtwaevdldaiydnvenlrrllpddthimavvKanayghgdvqvartaleagasrlavafldealal
rekgieapilvlgasrpadaalaaqqrialtvfrsdwleeasalysgpfpihfhlkmdtgmgRlgvkdeeetk
rivalierhphfvleglythfatadevntdyfsyqytrflhmlewlpsrpplvhcansaaslrfpdrtfnmvr
fgiamyglapspgikpllpyplkeafslhsrlvhvkklqpgekvsYgatytaqteewigtipigyadgwlrrl
qhfhvlvdgqkapivgriCmdqcmirlpgplpvgtkvtligrqgdevisiddvarhletinyevpctisyrvp
riffrhkrimevrnaigagessa
>1B6T_A
mqkraiypgtfdpitngHidivtratqmfdhvilaiaaspskkpmftleervalaqqatahlgnvevvgfsdl
manfarnqhatvlirglRavadfeyemqlahmnrhlmpelesvflmpskewsfisSslvkevarhqgdvthfl
penvhqalmakla
>1DJL_B
pmeisgthteinldnaidmireansiiitpgYglcaakaqypiadlvkmlteqgkkvrfgihpvagRmpgqln
vllaeagvpydivlemdeinhdfpdtdlvlvigandtvnsaaqedpnsiiagmpvlevwkskqvivmkrslgv
gYaavdnpifykpntamllgdakktcdalqakvres
>1QFE_B
mktvtvknliigegmpkiivslmgrdinsvkaealayreatfdilewrvdhfmdiastqsvltaarvirdamp
dipllftfrsakEggeqtittqhyltlnraaidsglvdmidlelftgdadvkatvdyahahnvyvvmsnHdfh
qtpsaeemvsrlrkmqalgadipKiavmpqskhdvltlltatlemqqhyadrpvitmsmakegvisrlagevf
gsaatfgavkqasapgqiavndlrsvlmilhna
>1P7M_A
mercgwvsqdplyiaYhdnewgvpetdskklfemiclEgqqaglsWitvlkkrenyracfhqfdpvkvaamqe
edverlvqdagiirhrgkiqaiignaraylqmeqngepfadfvwsfvnhqpqmtqattlseiptstpasdals
kalkkrgfkfvgtticysfmqacglvndhvvgcccypgnkp
>1DQR_A
aaltrnpqfqklqqwhrehgselnlrhlfdtdkerfnhfsltlntnhghilldysknlvteevmhmlldlaks
rgveaaresmfngekinstedravlhvalrnrsntpivvdgkdvmpevnkvldkmkafcqrvrsgdwkgytgk
titdvinigiggsdlgplmvtealkpyssggprvwfvsnidgthiaktlaclnpesslfiiasKtfttqEtit
naktakdwfllsakdpstvakhfvalstntakvkefgidpqnmfefwdwvgGRyslwsaiglsialhvgfdnf
eqllsgahwmdqhfrttpleknapvllamlgiwyincfgcetqavlpydqylhrfaayfqqgdmEsngkyitk
sgarvdhqtgpivwgepgtngqHafyqlihqgtkmipcdflipvqtqhpirkglhhkillanflaqtealmkg
ksteearkelqaagkspedlmkllphkvfegnrptnsivftkltpfilgaliamyehkifvqgvvwdinsfdq
wgvelgKqlakkiepeldgsspvtshdsstnglinfikqqreakiq
>1FQ0_A
```

mknwktsaesilttgpvvpvivvkklehavpmakalvaggvrvlEvtlrtecavdairaiakevpeaivgagt
vlnpqqlaevteagaqfaispgltepllkaategtiplipgistvselmlgmdyglkefKffpaeanggvkal
qaiagpfsqvrfcptggispanyrdylalksvlciggswlvpadaleagdydritklareavegakl
>1CVR_A
ytpveekengrmivivakkyegdikdfvdwknqrglrtevkvaediaspvtanaiqqfvkqeyekegndltyv
llvgdhkdipakitpgiksdqvygqivgndhynevfigrfsceskedlktqidrtihyernittedkwlgqal
ciasaEggpsadngesdiqhenvianlltqygytkiikcydpgvtpkniidafnggislvnytgHGsetawgt
shfgtthvkqltnsnqlpfifdvaCvngdflfsmpcfaealmraqkdgkptgtvaiiastidqywappmrgqd
emneilcekhpnnikrtfggvtmngmfamvekykkdgenmldtwtvfgdpsllvrtlvptemqvtapanisas
aqtfevacdyngaiatlsddgdmvgtaivkdgkaiiklnesiadetnltltvvgynkvtvikdvkvegts
>2TDT_A
mqqlqnviesaferraditpanvdtvtreavnqviglldsgalrvaekidgqwvthqwlkkavllsfrindnk
vmdgaetryydkvpmkfadydearfqkegfrvvppatvrqgafiarntvlmpsyvnigayvdegtmvDtwatv
gscaqigknvhlsggvgigGvlEplqanptiiedncfigarsevvegviveegsvismgvylgqstriydret
geihygrvpagsvvvsgnlpskdgsyslycavivkkvdaktrgkvginellrtid
