# Supplementary material for: PINGU: PredIction of eNzyme catalytic residues usinG seqUence information
Source: PLoS One. 2015 Aug 11;10(8):e0135122. doi: 10.1371/journal.pone.0135122 (PMC4532418; doi:10.1371/journal.pone.0135122)
Supplement: S1 Table — (PDF) [file pone.0135122.s002.pdf]

S1 Table. PINGU predictions in real scenario on 60 diverse enzymes

PDB chain	True Positive	False Negative	False Positive	True Negative	Sensitivity	Precision	Specificity	Accuracy	MCC	F-measure
**12AS_A**	3	0	33	280	1.0000	0.0833	0.8946	0.8956	0.273	0.1538
**1A0I_A**	1	0	29	304	1.0000	0.0333	0.9129	0.9132	0.174	0.0645
**1ALD_A**	3	0	81	265	1.0000	0.0357	0.7659	0.7679	0.165	0.0689
**1AOP_A**	5	0	118	360	1.0000	0.0407	0.7531	0.7557	0.175	0.0782
**1B6B_B**	3	2	12	125	0.6000	0.2000	0.9124	0.9014	0.307	0.3000
**1B6T_A**	2	1	36	106	0.6667	0.0526	0.7465	0.7448	0.134	0.0975
**1BD0_A**	4	0	85	285	1.0000	0.0449	0.7703	0.7727	0.186	0.0859
**1BD3_A**	2	1	32	175	0.6667	0.0588	0.8454	0.8429	0.165	0.1081
**1BIB_A**	2	0	46	259	1.0000	0.0417	0.8492	0.8502	0.188	0.0801
**1CA2_A**	3	0	36	203	1.0000	0.0769	0.8494	0.8512	0.256	0.1428
**1CGK_A**	4	0	135	236	1.0000	0.0288	0.6361	0.6400	0.135	0.0560
**1CHK_A**	2	0	23	199	1.0000	0.0800	0.8964	0.8973	0.268	0.1481
**1CVR_A**	4	0	43	374	1.0000	0.0851	0.8969	0.8979	0.276	0.1569
**1CZF_A**	4	0	49	295	1.0000	0.0755	0.8576	0.8592	0.254	0.1404
**1DB3_A**	4	0	111	243	1.0000	0.0348	0.6864	0.6899	0.155	0.0673
**1DE6_A**	3	0	52	347	1.0000	0.0545	0.8697	0.8706	0.218	0.1034
**1DI1_A**	1	2	26	257	0.3333	0.0370	0.9081	0.9021	0.084	0.0666
**1DJL_B**	3	0	41	124	1.0000	0.0682	0.7515	0.7560	0.226	0.1277
**1DLI_A**	6	0	109	273	1.0000	0.0522	0.7147	0.7191	0.193	0.0992
**1DNK_A**	4	0	21	221	1.0000	0.1600	0.9132	0.9146	0.382	0.2759
**1DNP_B**	3	0	108	344	1.0000	0.0270	0.7611	0.7626	0.143	0.0526
**1DO8_A**	3	0	109	413	1.0000	0.0268	0.7912	0.7924	0.146	0.0522
**1DQR_A**	6	1	155	381	0.8571	0.0373	0.7108	0.7127	0.140	0.0715
**1EQ2_A**	3	0	59	234	1.0000	0.0484	0.7986	0.8007	0.197	0.0923
**1F6D_A**	4	0	113	245	1.0000	0.0342	0.6844	0.6878	0.153	0.0661
**1F8X_A**	2	1	17	123	0.6667	0.1053	0.8786	0.8741	0.230	0.1819
**1FCB_A**	5	0	80	412	1.0000	0.0588	0.8374	0.8390	0.222	0.1111
**1FQ0_A**	2	0	42	155	1.0000	0.0455	0.7868	0.7889	0.189	0.0870
**1G99_A**	5	0	102	287	1.0000	0.0467	0.7378	0.7411	0.186	0.0892
**1GCU_A**	2	4	21	254	0.3333	0.0870	0.9236	0.9110	0.136	0.1380
**1GIM_A**	3	0	142	272	1.0000	0.0207	0.6570	0.6595	0.117	0.0406

**1GSA_A**	3	0	65	234	1.0000	0.0441	0.7826	0.7848	0.186	0.0845
**1H7A_A**	5	0	132	412	1.0000	0.0365	0.7574	0.7596	0.166	0.0704
**1HRK_A**	6	0	53	286	1.0000	0.1017	0.8437	0.8464	0.293	0.1846
**1HTO_X**	3	0	131	329	1.0000	0.0224	0.7152	0.7171	0.127	0.0438
**1JHF_A**	4	1	22	161	0.8000	0.1538	0.8798	0.8777	0.317	0.2580
**1KAS_A**	4	0	114	280	1.0000	0.0339	0.7107	0.7136	0.155	0.0656
**1KEZ_B**	4	0	19	230	1.0000	0.1739	0.9237	0.9249	0.401	0.2963
**1L7N_B**	5	1	23	165	0.8333	0.1786	0.8777	0.8763	0.350	0.2942
**1M9C_A**	4	2	63	82	0.6667	0.0597	0.5655	0.5695	0.091	0.1096
**1N2C_C**	4	2	69	389	0.6667	0.0548	0.8493	0.8470	0.160	0.1013
**1NN4_A**	4	0	36	105	1.0000	0.1000	0.7447	0.7517	0.273	0.1818
**1NSF_A**	2	1	21	209	0.6667	0.0870	0.9087	0.9056	0.217	0.1539
**1O98_A**	3	0	141	353	1.0000	0.0208	0.7146	0.7163	0.122	0.0408
**1OK4_J**	2	1	25	208	0.6667	0.0741	0.8927	0.8898	0.197	0.1334
**1P7M_A**	2	1	33	137	0.6667	0.0571	0.8059	0.8035	0.154	0.1052
**1PYM_B**	3	1	39	225	0.7500	0.0714	0.8523	0.8507	0.201	0.1304
**1Q6L_B**	7	2	21	171	0.7778	0.2500	0.8906	0.8856	0.399	0.3784
**1QFE_B**	2	1	23	212	0.6667	0.0800	0.9021	0.8992	0.207	0.1429
**1SES_B**	4	1	73	329	0.8000	0.0519	0.8184	0.8182	0.174	0.0975
**1W1O_A**	1	2	63	454	0.3333	0.0156	0.8781	0.8750	0.049	0.0298
**1YTW_A**	5	1	44	242	0.8333	0.1020	0.8462	0.8459	0.258	0.1818
**1Z9H_A**	2	2	15	241	0.5000	0.1176	0.9414	0.9346	0.220	0.1904
**2A86_B**	7	0	57	199	1.0000	0.1094	0.7773	0.7833	0.292	0.1972
**2DLN_A**	5	0	72	215	1.0000	0.0649	0.7491	0.7534	0.221	0.1219
**2F9R_B**	8	1	26	236	0.8889	0.2353	0.9008	0.9004	0.427	0.3721
**2OAT_A**	3	0	96	326	1.0000	0.0303	0.7725	0.7741	0.153	0.0588
**2PGD_A**	4	0	121	343	1.0000	0.0320	0.7392	0.7415	0.154	0.0620
**2TDT_A**	2	1	74	183	0.6667	0.0263	0.7121	0.7115	0.089	0.0506
**2TS1_A**	2	2	85	316	0.5000	0.0230	0.7880	0.7852	0.069	0.0440
