# Supplementary material for: PINGU: PredIction of eNzyme catalytic residues usinG seqUence information
Source: PLoS One. 2015 Aug 11;10(8):e0135122. doi: 10.1371/journal.pone.0135122 (PMC4532418; doi:10.1371/journal.pone.0135122)
Supplement: S2 Table — (PDF) [file pone.0135122.s003.pdf]

S2 Table. Prediction performance upon application of post-processing filter in real scenario on 60 diverse enzymes

PDB chain	True Positive	False Negative	False Positive	True Negative	Sensitivity	Precision	Specificity	Accuracy	MCC	F-measure
**12AS_A**	3	0	2	311	1.000	0.600	0.994	0.994	0.772	0.750
**1A0I_A**	1	0	12	321	1.000	0.077	0.964	0.964	0.272	0.143
**1ALD_A**	3	0	17	329	1.000	0.150	0.951	0.951	0.378	0.261
**1AOP_A**	5	0	30	448	1.000	0.143	0.937	0.938	0.366	0.250
**1B6B_B**	2	3	2	135	0.400	0.500	0.985	0.965	0.429	0.444
**1B6T_A**	2	1	14	128	0.667	0.125	0.901	0.897	0.258	0.211
**1BD0_A**	2	2	13	357	0.500	0.133	0.965	0.960	0.244	0.210
**1BD3_A**	2	1	9	198	0.667	0.182	0.957	0.952	0.332	0.286
**1BIB_A**	2	0	19	286	1.000	0.095	0.938	0.938	0.299	0.174
**1CA2_A**	2	1	9	230	0.667	0.182	0.962	0.959	0.334	0.286
**1CGK_A**	4	0	16	355	1.000	0.200	0.957	0.957	0.437	0.333
**1CHK_A**	1	1	1	221	0.500	0.500	0.995	0.991	0.495	0.500
**1CVR_A**	4	0	4	413	1.000	0.500	0.990	0.990	0.704	0.667
**1CZF_A**	0	4	2	342	0.000	0.000	0.994	0.983	-0.008	NA
**1DB3_A**	3	1	15	339	0.750	0.167	0.958	0.955	0.340	0.273
**1DE6_A**	3	0	10	389	1.000	0.231	0.975	0.975	0.474	0.375
**1DI1_A**	1	2	7	276	0.333	0.125	0.975	0.969	0.191	0.182
**1DJL_B**	3	0	16	149	1.000	0.158	0.903	0.905	0.378	0.273
**1DLI_A**	2	4	15	367	0.333	0.118	0.961	0.951	0.177	0.174
**1DNK_A**	2	2	5	237	0.500	0.286	0.979	0.972	0.365	0.364
**1DNP_B**	1	2	20	432	0.333	0.048	0.956	0.952	0.112	0.084
**1DO8_A**	2	1	9	513	0.667	0.182	0.983	0.981	0.342	0.286
**1DQR_A**	4	3	10	526	0.571	0.286	0.981	0.976	0.393	0.381
**1EQ2_A**	3	0	14	279	1.000	0.176	0.952	0.953	0.410	0.299
**1F6D_A**	4	0	15	343	1.000	0.211	0.958	0.959	0.449	0.348
**1F8X_A**	2	1	4	136	0.667	0.333	0.971	0.965	0.456	0.444
**1FCB_A**	4	1	16	476	0.800	0.200	0.967	0.966	0.390	0.320
**1FQ0_A**	2	0	7	190	1.000	0.222	0.964	0.965	0.463	0.363
**1G99_A**	3	2	16	373	0.600	0.158	0.959	0.954	0.292	0.250

**1GCU_A**	2	4	9	266	0.333	0.182	0.967	0.954	0.224	0.235
**1GIM_A**	3	0	19	395	1.000	0.136	0.954	0.954	0.361	0.239
**1GSA_A**	2	1	6	293	0.667	0.250	0.980	0.977	0.399	0.364
**1H7A_A**	1	4	9	535	0.200	0.100	0.983	0.976	0.130	0.133
**1HRK_A**	2	4	17	322	0.333	0.105	0.950	0.939	0.162	0.160
**1HTO_X**	1	2	12	448	0.333	0.077	0.974	0.970	0.149	0.125
**1JHF_A**	0	5	2	181	0.000	0.000	0.989	0.963	-0.017	NA
**1KAS_A**	4	0	10	384	1.000	0.286	0.975	0.975	0.528	0.445
**1KEZ_B**	3	1	2	247	0.750	0.600	0.992	0.988	0.665	0.667
**1L7N_B**	5	1	3	185	0.833	0.625	0.984	0.979	0.712	0.714
**1M9C_A**	4	2	6	139	0.667	0.400	0.959	0.947	0.491	0.500
**1N2C_C**	2	4	9	449	0.333	0.182	0.980	0.972	0.233	0.235
**1NN4_A**	3	1	8	133	0.750	0.273	0.943	0.938	0.429	0.400
**1NSF_A**	1	2	6	224	0.333	0.143	0.974	0.966	0.203	0.200
**1O98_A**	1	2	3	491	0.333	0.250	0.994	0.990	0.284	0.286
**1OK4_J**	2	1	7	226	0.667	0.222	0.970	0.966	0.372	0.333
**1P7M_A**	2	1	3	167	0.667	0.400	0.982	0.977	0.506	0.500
**1PYM_B**	3	1	12	252	0.750	0.200	0.955	0.951	0.372	0.316
**1Q6L_B**	1	8	9	183	0.111	0.100	0.953	0.915	0.061	0.105
**1QFE_B**	2	1	8	227	0.667	0.200	0.966	0.962	0.352	0.308
**1SES_B**	3	2	18	384	0.600	0.143	0.955	0.951	0.276	0.231
**1W1O_A**	0	3	5	512	0.000	0.000	0.990	0.985	-0.008	NA
**1YTW_A**	3	3	10	276	0.500	0.231	0.965	0.955	0.320	0.316
**1Z9H_A**	1	3	2	254	0.250	0.333	0.992	0.981	0.279	0.286
**2A86_B**	7	0	12	244	1.000	0.368	0.953	0.954	0.593	0.538
**2DLN_A**	1	4	12	275	0.200	0.077	0.958	0.945	0.100	0.111
**2F9R_B**	4	5	2	260	0.444	0.667	0.992	0.974	0.532	0.533
**2OAT_A**	3	0	13	409	1.000	0.188	0.969	0.969	0.426	0.316
**2PGD_A**	4	0	11	453	1.000	0.267	0.976	0.976	0.510	0.421
**2TDT_A**	1	2	3	254	0.333	0.250	0.988	0.981	0.279	0.286
**2TS1_A**	0	4	17	384	0.000	0.000	0.958	0.948	-0.021	NA
